# Supplementary figures and images for: An expanded cell wall damage signaling network is comprised of the transcription factors Rlm1 and Sko1 in Candida albicans
Source: PLoS Genet. 2020 Jul 8;16(7):e1008908. doi: 10.1371/journal.pgen.1008908 (PMC7371209; doi:10.1371/journal.pgen.1008908)

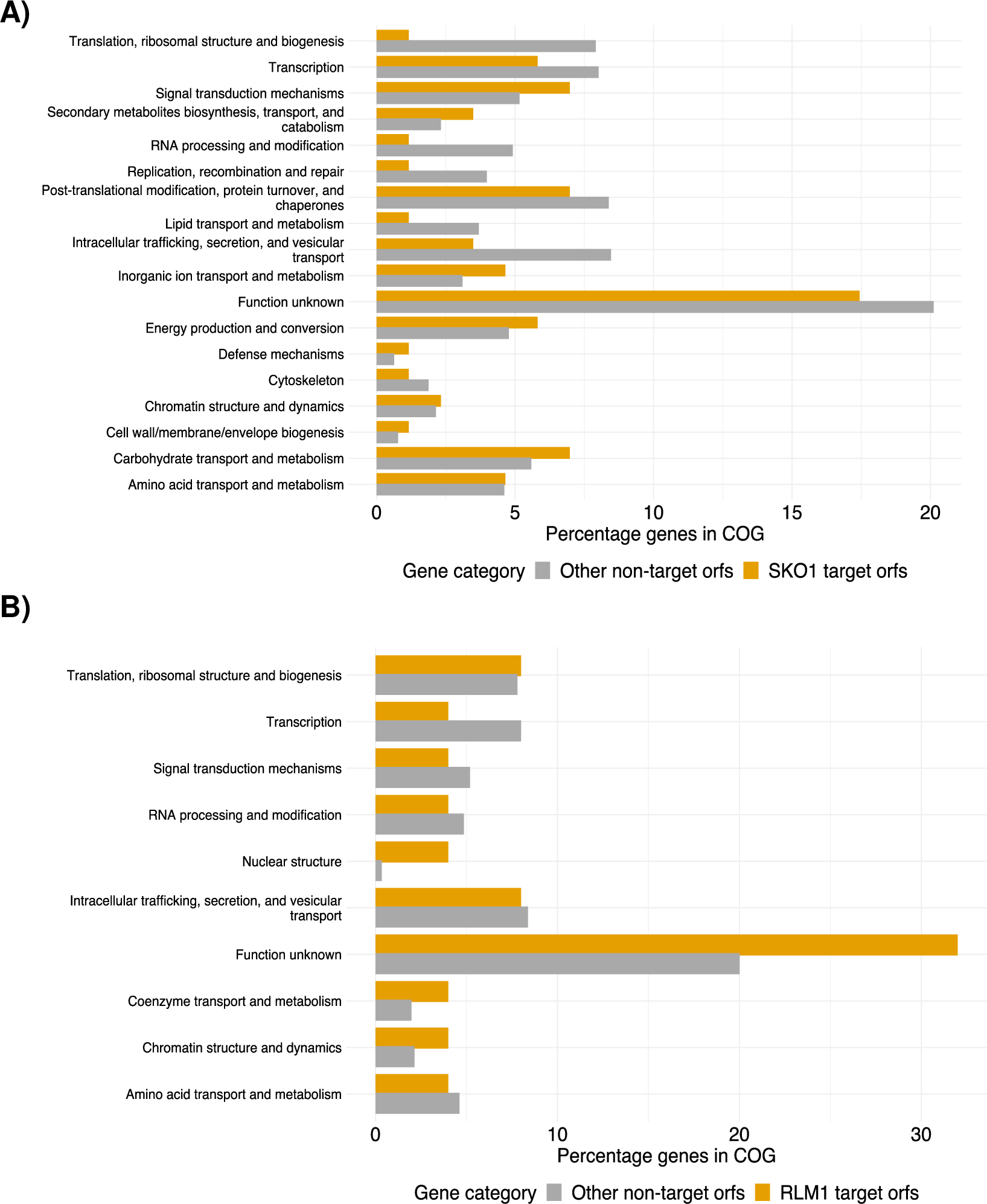

Supplement: S1 Fig — COG functional categories were determined using eggNOG-mapper for A) Sko1 target genes and B) Rlm1 target genes. (TIF) [file pgen.1008908.s003.tif]

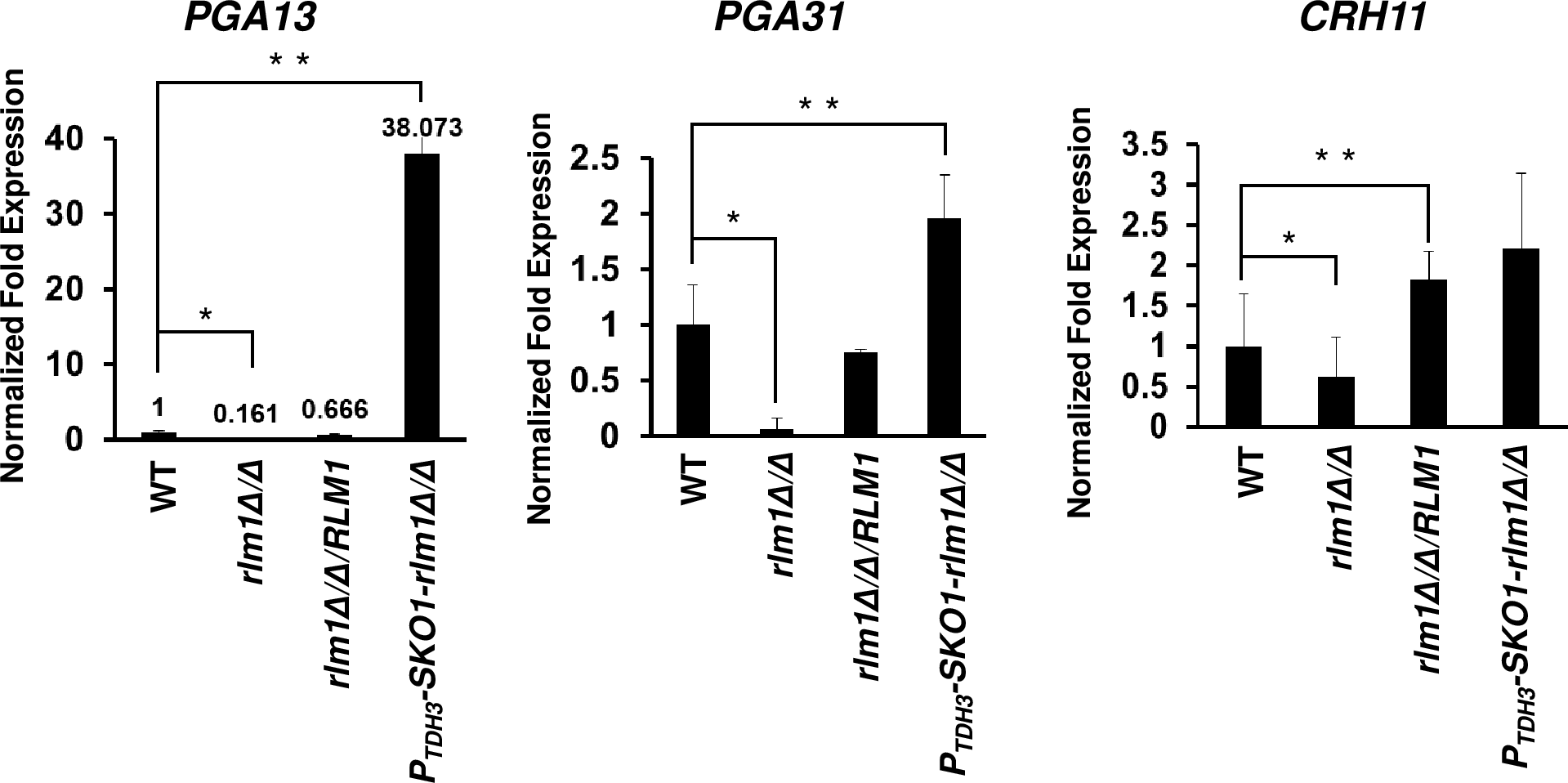

Supplement: S2 Fig — Expression of Sko1 and Rlm1 CWD response genes, PGA13, PGA31, and CRH11 was monitored via RT-qPCR analysis in the wild-type strain, rlm1Δ/Δ mutant strain and PTDH3SKO1-rlm1Δ/Δ overexpressing strain. Gene expression changes were normalized to the caspofungin-treated wild-type strain adjusted to the value of 1.0. Data presented represents the mean of three biological replicates. The single asterisk indicates a P-value of ≤ 0.05 between the wild-type and rlm1Δ/Δ mutant strains. The double asterisk indicates a P-value of ≤ 0.05 between the wild-type and PTDH3SKO1-rlm1Δ/Δ overexpressing strains. (TIF) [file pgen.1008908.s004.tif]

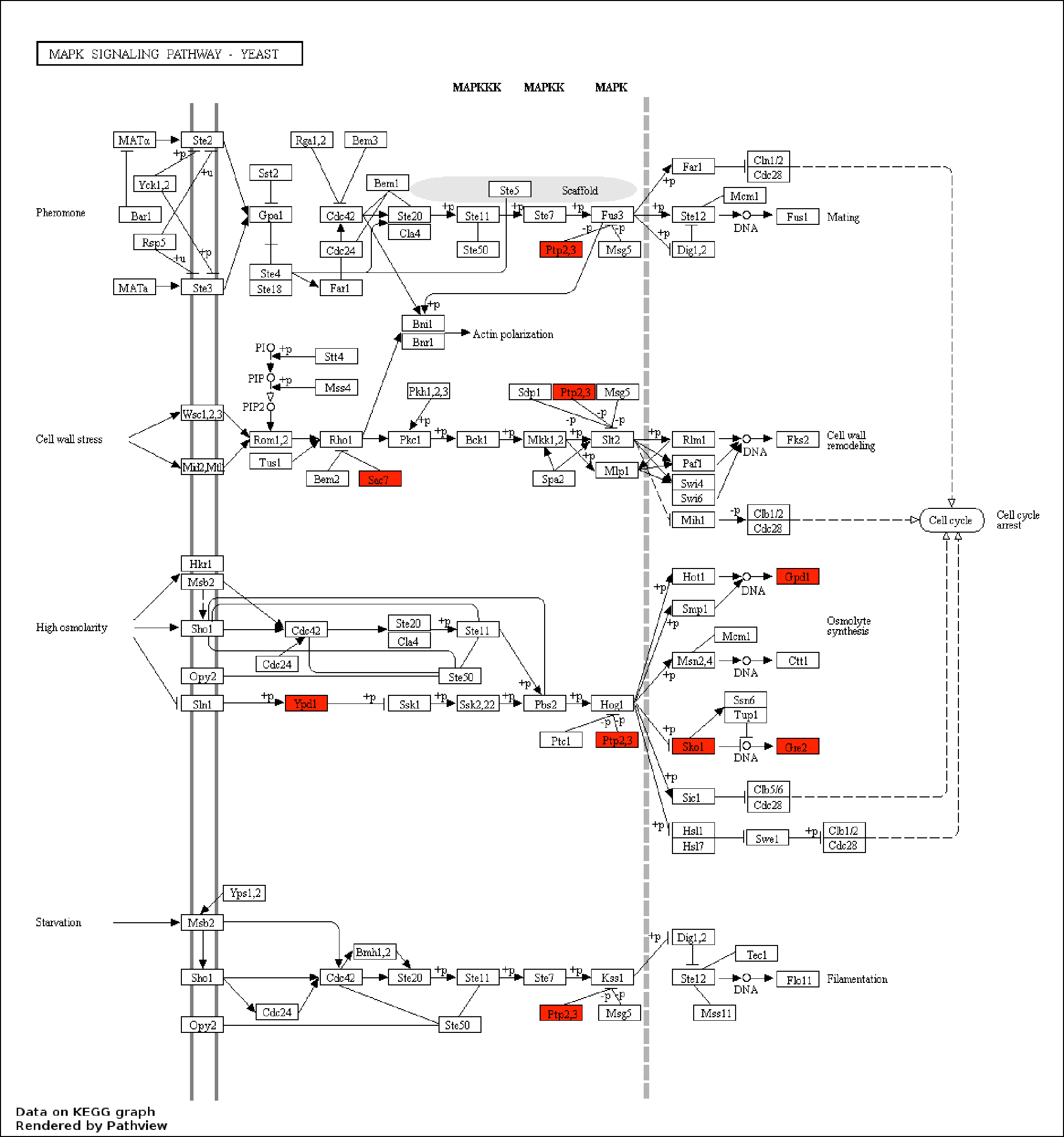

Supplement: S3 Fig — Sko1 direct target genes are highlighted in red rectangular boxes. (TIF) [file pgen.1008908.s005.tif]
