# Supplementary material for: An expanded cell wall damage signaling network is comprised of the transcription factors Rlm1 and Sko1 in Candida albicans
Source: PLoS Genet. 2020 Jul 8;16(7):e1008908. doi: 10.1371/journal.pgen.1008908 (PMC7371209; doi:10.1371/journal.pgen.1008908)
Supplement: S1 Appendix — Genes are listed in descending order for Rlm1 upstream intergenic region enrichment. (PDF) [file pgen.1008908.s006.pdf]

ORF Number	Gene	Page in S1 Appendix
orf19.2766	*ORF19.2766*	2,3
orf19.2765	*PGA62*	2,3
orf19.2767	*PGA59*	2,3
orf19.6081	*PHR2*	4
orf19.3697	*ORF19.3697*	5
orf19.3698	*ORF19.3698*	5
orf19.1297	*ORF19.1297*	6
orf19.1298	*NUP84*	6
orf19.1142	*AVT4*	7
orf19.3669	*SHA3*	8
orf19.2770	*ORF19.2770*	9
orf19.1742	*HEM3*	10
orf19.1105.2	*PGA56*	11
orf19.1105.3	*ORF19.1105.3*	11
orf19.3220	*ORF19.3220*	11
orf19.927	*ORF19.927*	12
orf19.5322	*ORF19.5322*	13
orf19.5326	*MIG2*	13
orf19.1302	*ORF19.1302*	14
orf19.1303	*ORF19.1303*	14
orf19.2638	*ORF19.2638*	15
orf19.2639	*ORF19.2639*	15
orf19.6654	*ORF19.6654*	16
orf19.7186	*CLB4*	17
orf19.5094	*BUL1*	18
orf19.3086	*SEC10*	19
orf19.3087	*UBI3*	19
orf19.3087.1	*ORF19.3087.1*	19
orf19.1991	*ORF19.1991*	20
orf19.1992	*SIR2*	20

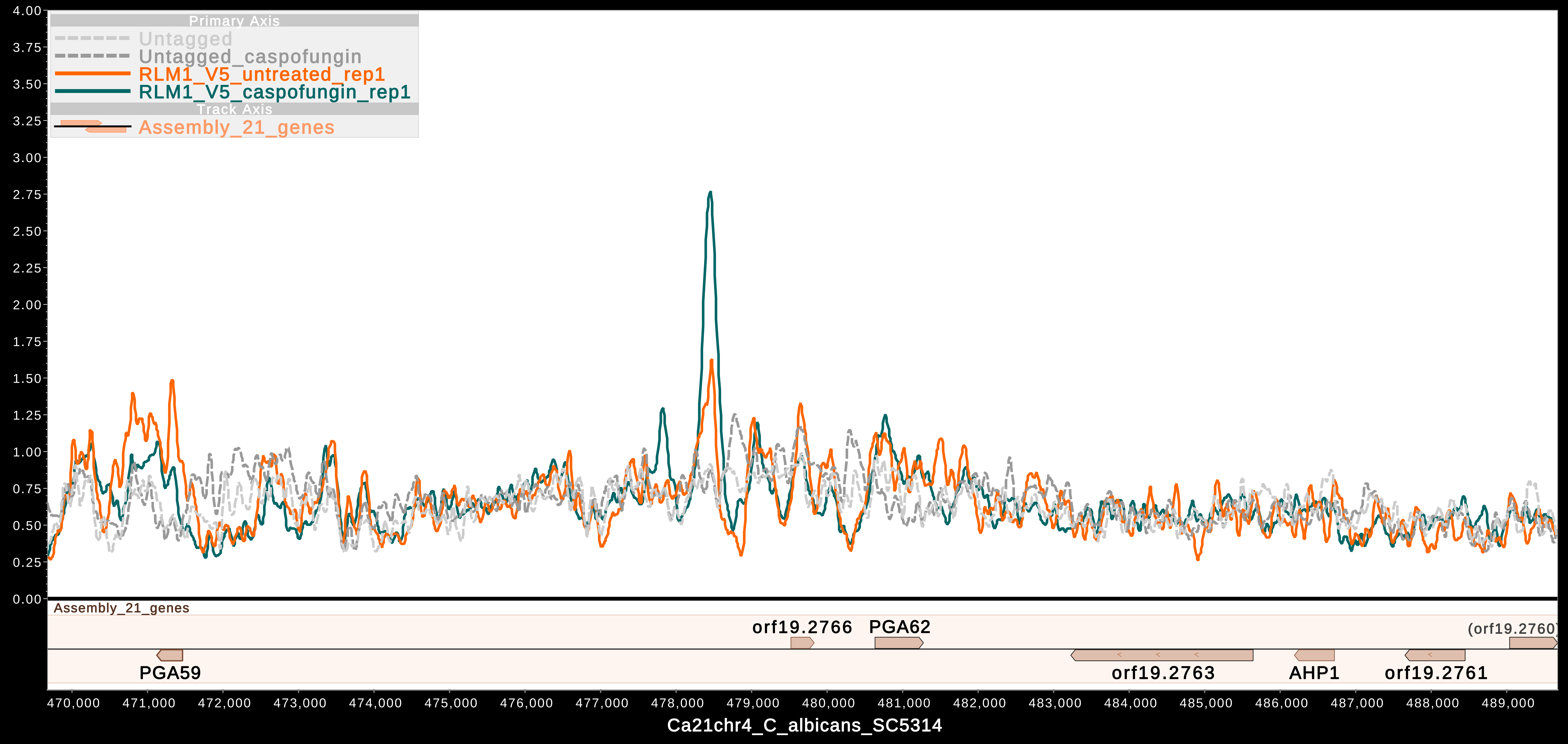

Primary Axis
Untagged
Untagged_caspofungin
RLM1_V5_untreated_rep1
RLM1_V5_caspofungin_rep1
Track Axis
Assembly_21_genes
4.00
3.75
3.50
3.25
3.00
2.75
2.50
2.25
2.00
1.75
1.50
1.25
1.00
0.75
0.50
0.25
0.00
Assembly_21_genes
orf19.2766
PGA62
(orf19.2760)
PGA59
orf19.2763
AHP1
orf19.2761
470,000
471,000
472,000
473,000
474,000
475,000
476,000
477,000
478,000
479,000
480,000
481,000
482,000
483,000
484,000
485,000
486,000
487,000
488,000
489,000
Ca21chr4_C_albicans_SC5314

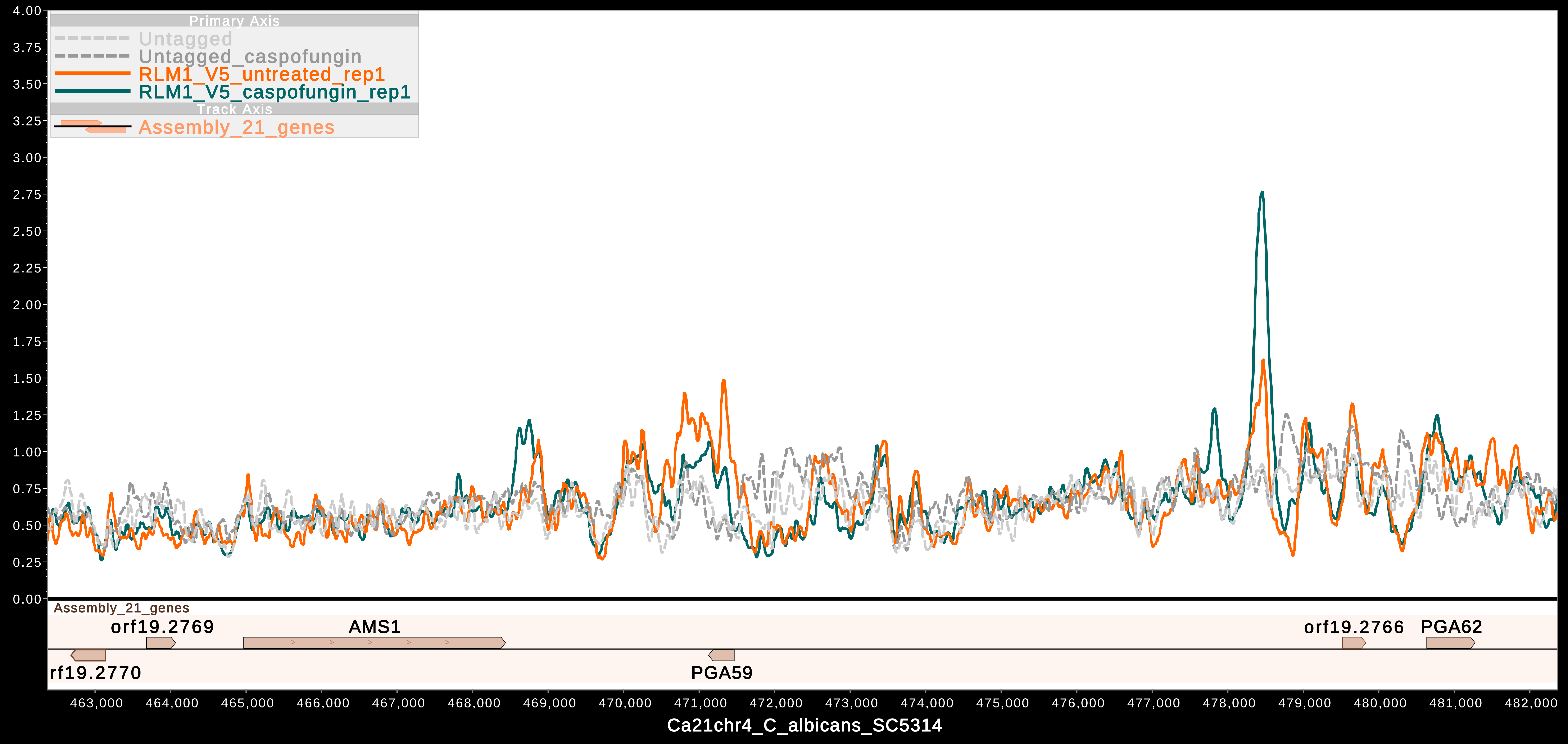

Primary Axis
Untagged
Untagged_caspofungin
RLM1_V5_untreated_rep1
RLM1_V5_caspofungin_rep1
Track Axis
Assembly_21_genes
4.00
3.75
3.50
3.25
3.00
2.75
2.50
2.25
2.00
1.75
1.50
1.25
1.00
0.75
0.50
0.25
0.00
Assembly_21_genes
orf19.2769
AMS1
orf19.2766
PGA62
rf19.2770
PGA59
463,000
464,000
465,000
466,000
467,000
468,000
469,000
470,000
471,000
472,000
473,000
474,000
475,000
476,000
477,000
478,000
479,000
480,000
481,000
482,000
Ca21chr4_C_albicans_SC5314

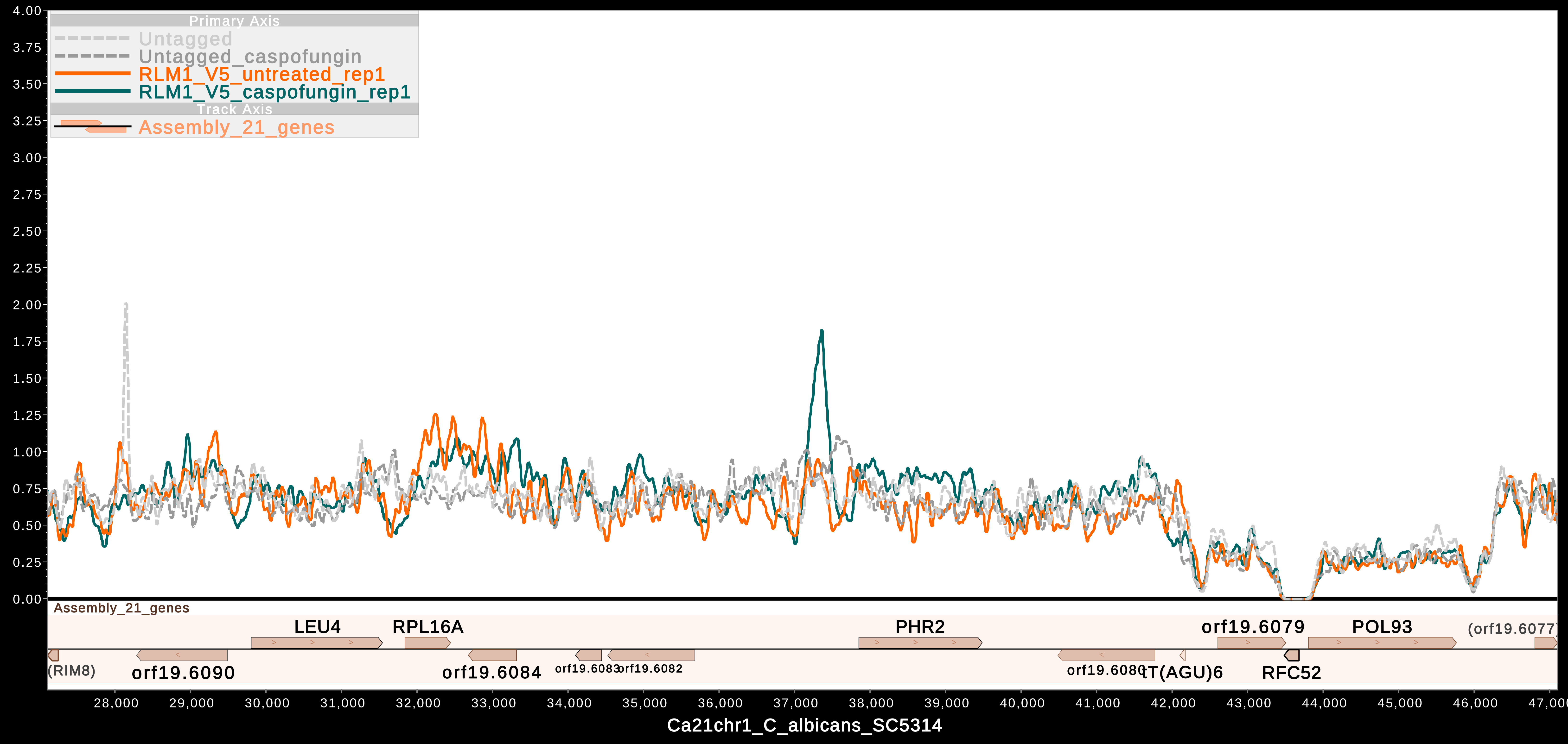

Primary Axis
Untagged
Untagged_caspofungin
RLM1_V5_untreated_rep1
RLM1_V5_caspofungin_rep1
Track Axis
Assembly_21_genes
4.00
3.75
3.50
3.25
3.00
2.75
2.50
2.25
2.00
1.75
1.50
1.25
1.00
0.75
0.50
0.25
0.00
Assembly_21_genes
LEU4
RPL16A
PHR2
orf19.6079
POL93
(orf19.6077)
(RIM8)
orf19.6090
orf19.6084
orf19.6083
orf19.6082
orf19.6080
tT(AGU)6
RFC52
28,000
29,000
30,000
31,000
32,000
33,000
34,000
35,000
36,000
37,000
38,000
39,000
40,000
41,000
42,000
43,000
44,000
45,000
46,000
47,00
Ca21chr1_C_albicans_SC5314

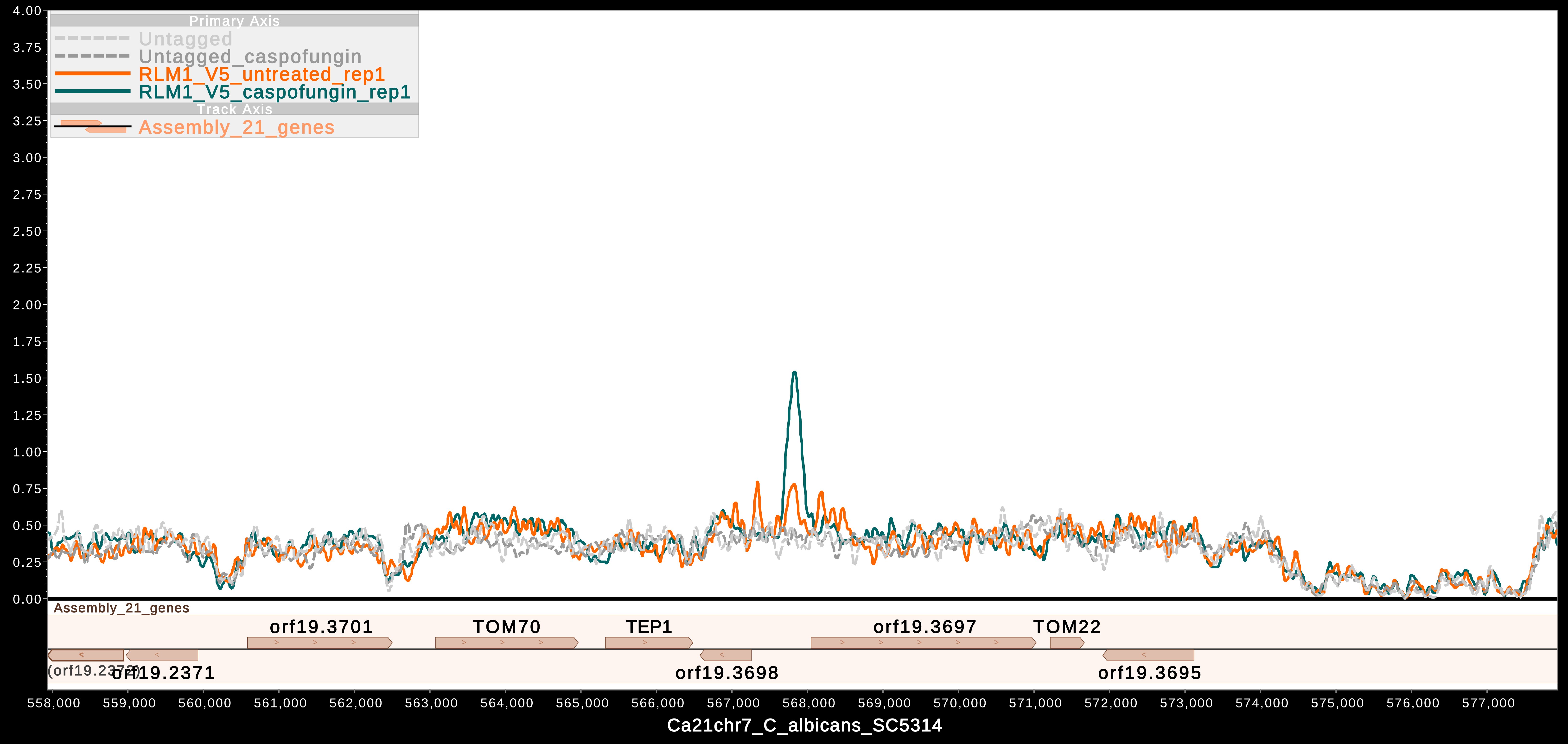

Primary Axis
Untagged
Untagged_caspofungin
RLM1_V5_untreated_rep1
RLM1_V5_caspofungin_rep1
Track Axis
Assembly_21_genes
4.00
3.75
3.50
3.25
3.00
2.75
2.50
2.25
2.00
1.75
1.50
1.25
1.00
0.75
0.50
0.25
0.00
Assembly_21_genes
orf19.3701
TOM70
TEP1
orf19.3697
TOM22
(orf19.2372)
orf19.2371
orf19.3698
orf19.3695
558,000
559,000
560,000
561,000
562,000
563,000
564,000
565,000
566,000
567,000
568,000
569,000
570,000
571,000
572,000
573,000
574,000
575,000
576,000
577,000
Ca21chr7_C_albicans_SC5314

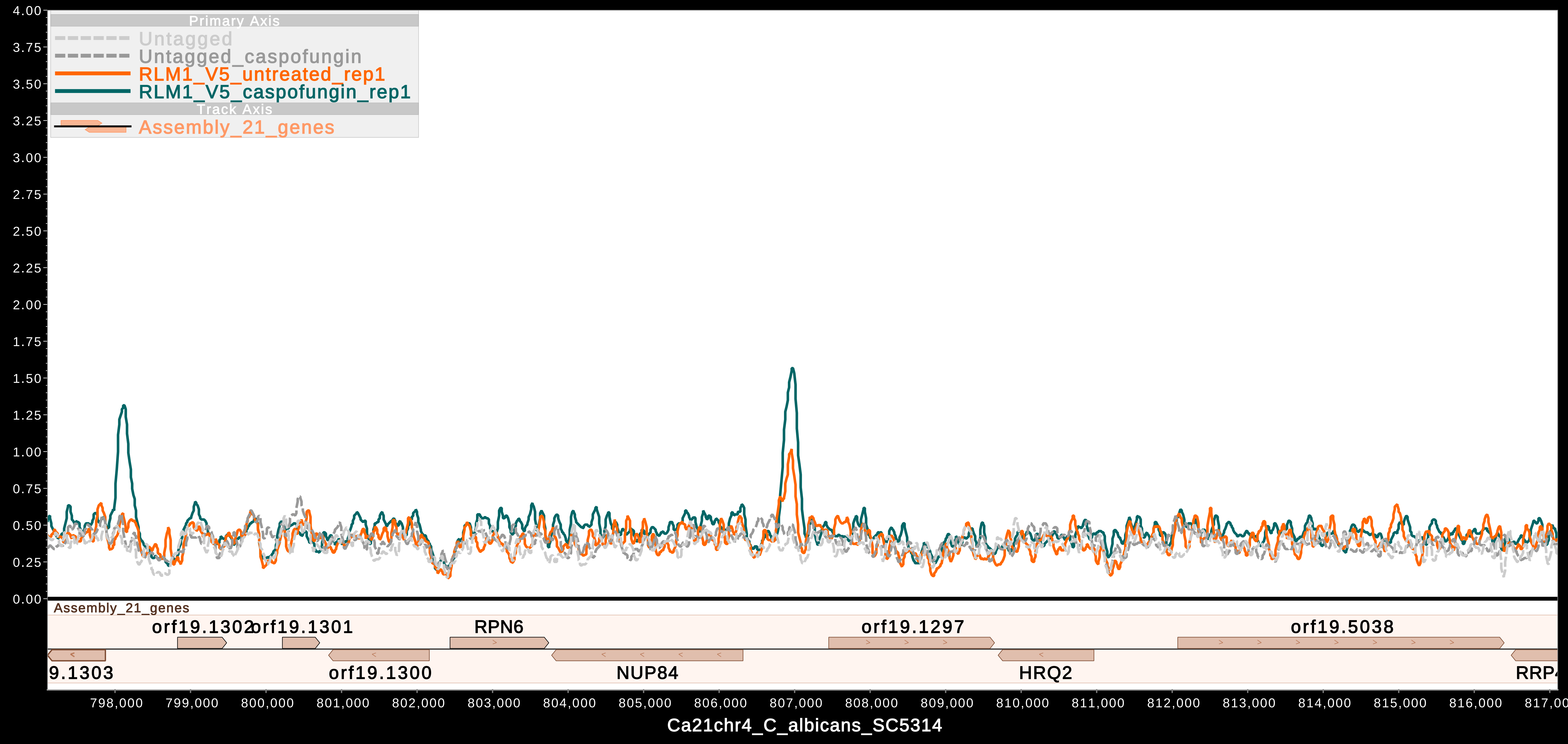

Primary Axis
Untagged
Untagged_caspofungin
RLM1_V5_untreated_rep1
RLM1_V5_caspofungin_rep1
Track Axis
Assembly_21_genes
4.00
3.75
3.50
3.25
3.00
2.75
2.50
2.25
2.00
1.75
1.50
1.25
1.00
0.75
0.50
0.25
0.00
Assembly_21_genes
orf19.1302
orf19.1301
RPN6
orf19.1297
orf19.5038
9.1303
orf19.1300
NUP84
HRQ2
RRP
798,000
799,000
800,000
801,000
802,000
803,000
804,000
805,000
806,000
807,000
808,000
809,000
810,000
811,000
812,000
813,000
814,000
815,000
816,000
817,00
Ca21chr4_C_albicans_SC5314

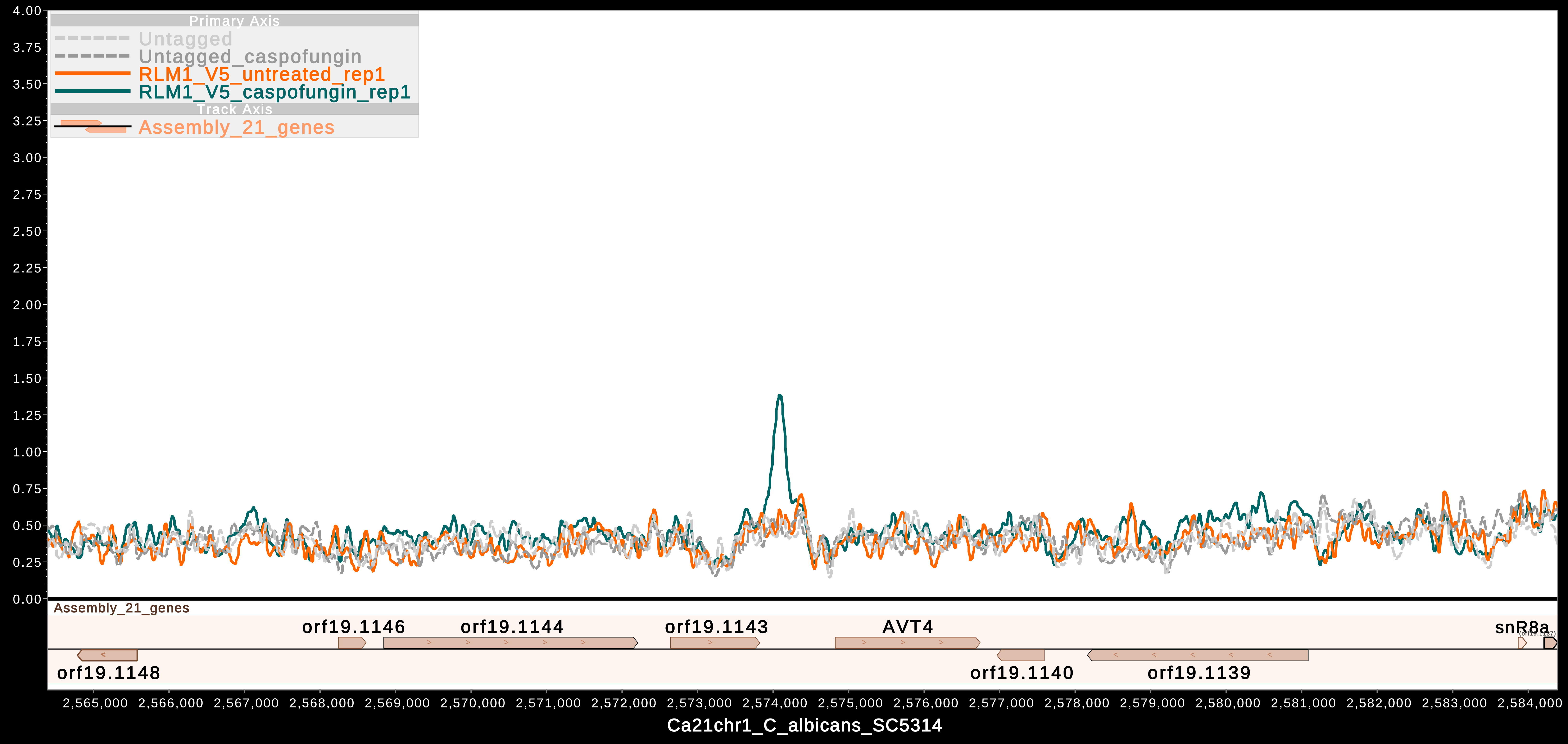

Primary Axis
Untagged
Untagged_caspofungin
RLM1_V5_untreated_rep1
RLM1_V5_caspofungin_rep1
Track Axis
Assembly_21_genes
4.00
3.75
3.50
3.25
3.00
2.75
2.50
2.25
2.00
1.75
1.50
1.25
1.00
0.75
0.50
0.25
0.00
Assembly_21_genes
orf19.1146
orf19.1144
orf19.1143
AVT4
snR8a
orf19.1148
orf19.1140
orf19.1139
2,565,000
2,566,000
2,567,000
2,568,000
2,569,000
2,570,000
2,571,000
2,572,000
2,573,000
2,574,000
2,575,000
2,576,000
2,577,000
2,578,000
2,579,000
2,580,000
2,581,000
2,582,000
2,583,000
2,584,000
Ca21chr1_C_albicans_SC5314

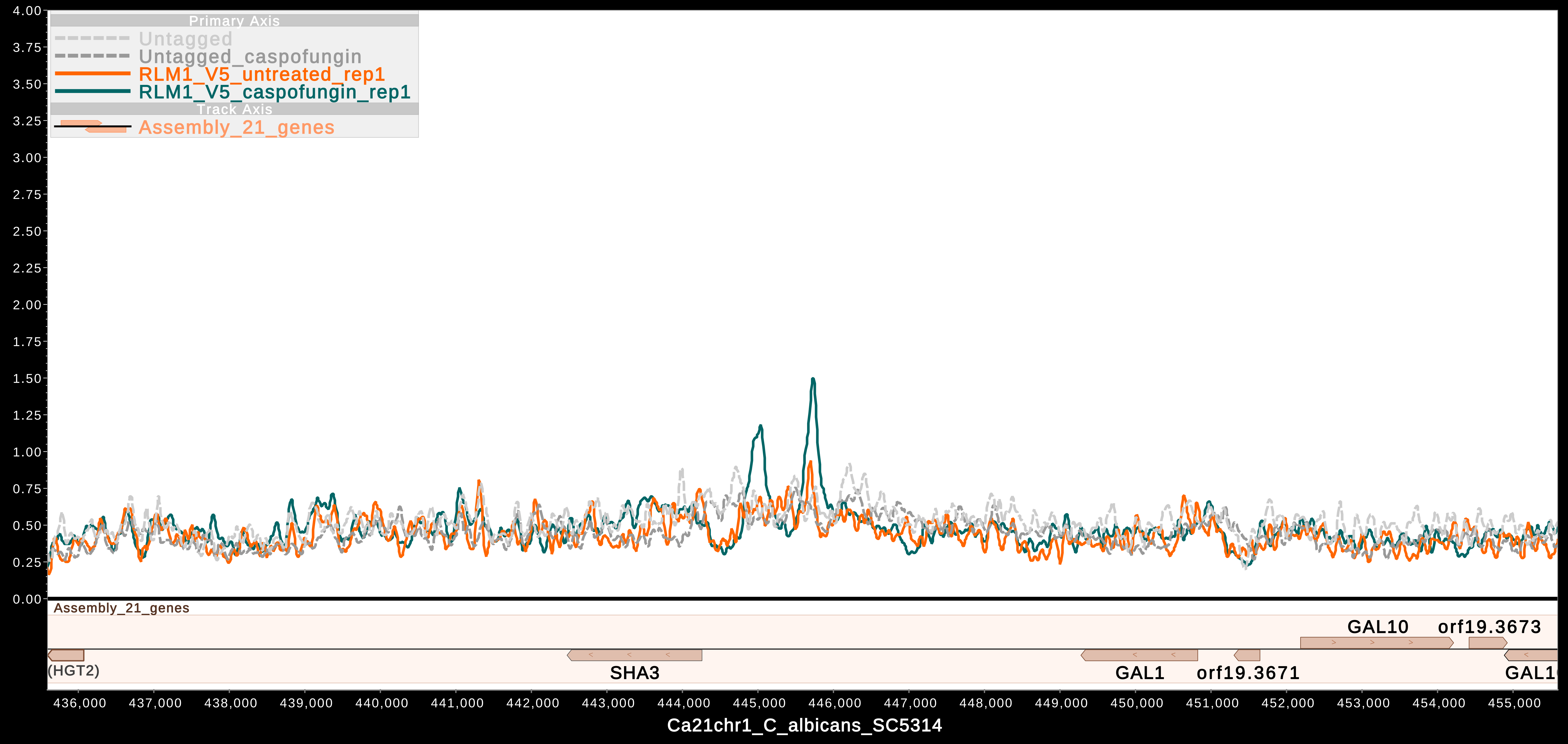

Primary Axis
Untagged
Untagged_caspofungin
RLM1_V5_untreated_rep1
RLM1_V5_caspofungin_rep1
Track Axis
Assembly_21_genes
4.00
3.75
3.50
3.25
3.00
2.75
2.50
2.25
2.00
1.75
1.50
1.25
1.00
0.75
0.50
0.25
0.00
Assembly_21_genes
GAL10
orf19.3673
(HGT2)
SHA3
GAL1
orf19.3671
436,000
437,000
438,000
439,000
440,000
441,000
442,000
443,000
444,000
445,000
446,000
447,000
448,000
449,000
450,000
451,000
452,000
453,000
454,000
455,000
Ca21chr1_C_albicans_SC5314

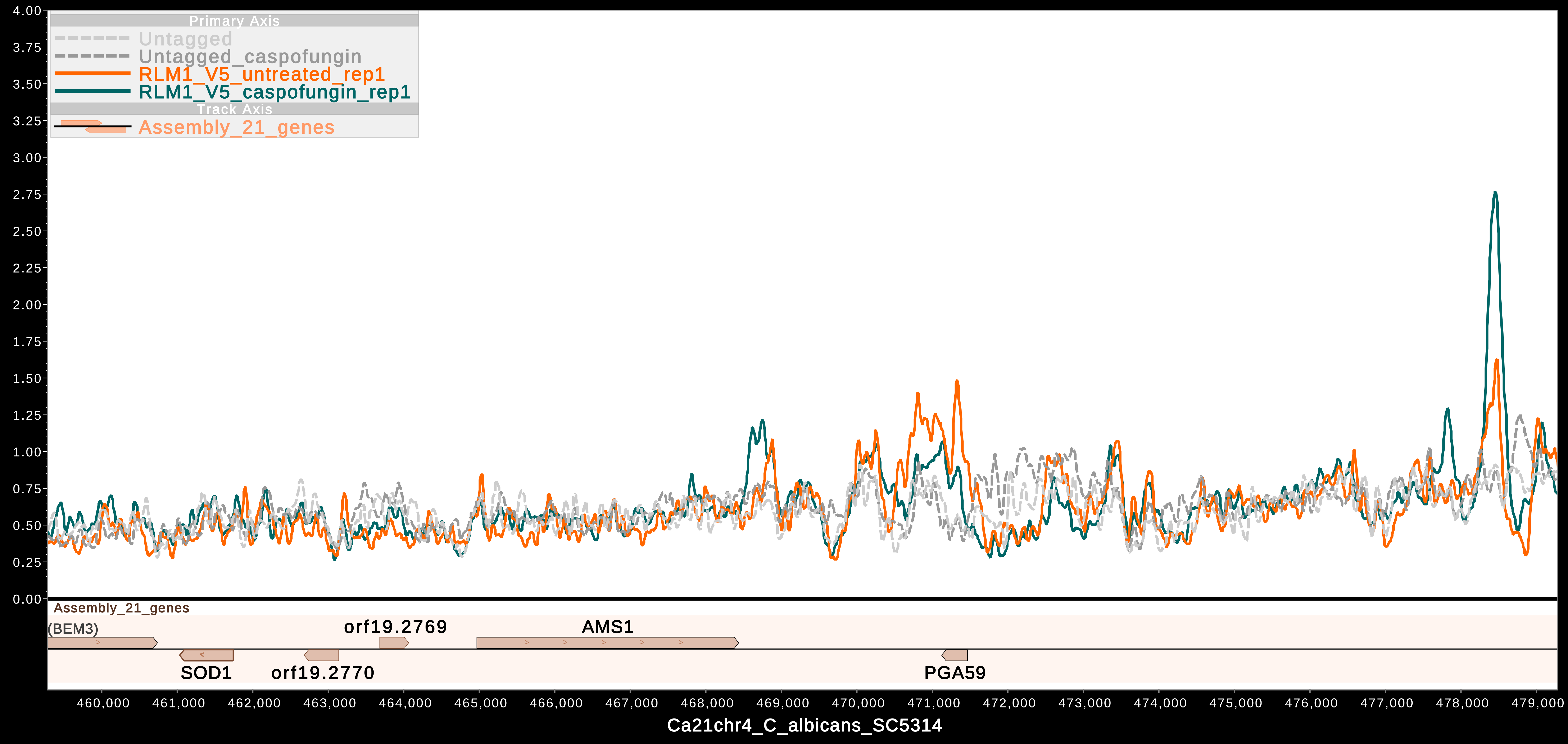
Primary Axis
Untagged
Untagged_caspofungin
RLM1_V5_untreated_rep1
RLM1_V5_caspofungin_rep1
Track Axis
Assembly_21_genes
4.00
3.75
3.50
3.25
3.00
2.75
2.50
2.25
2.00
1.75
1.50
1.25
1.00
0.75
0.50
0.25
0.00
Assembly_21_genes
(BEM3)
orf19.2769
AMS1
SOD1
orf19.2770
PGA59
460,000
461,000
462,000
463,000
464,000
465,000
466,000
467,000
468,000
469,000
470,000
471,000
472,000
473,000
474,000
475,000
476,000
477,000
478,000
479,000
Ca21chr4_C_albicans_SC5314

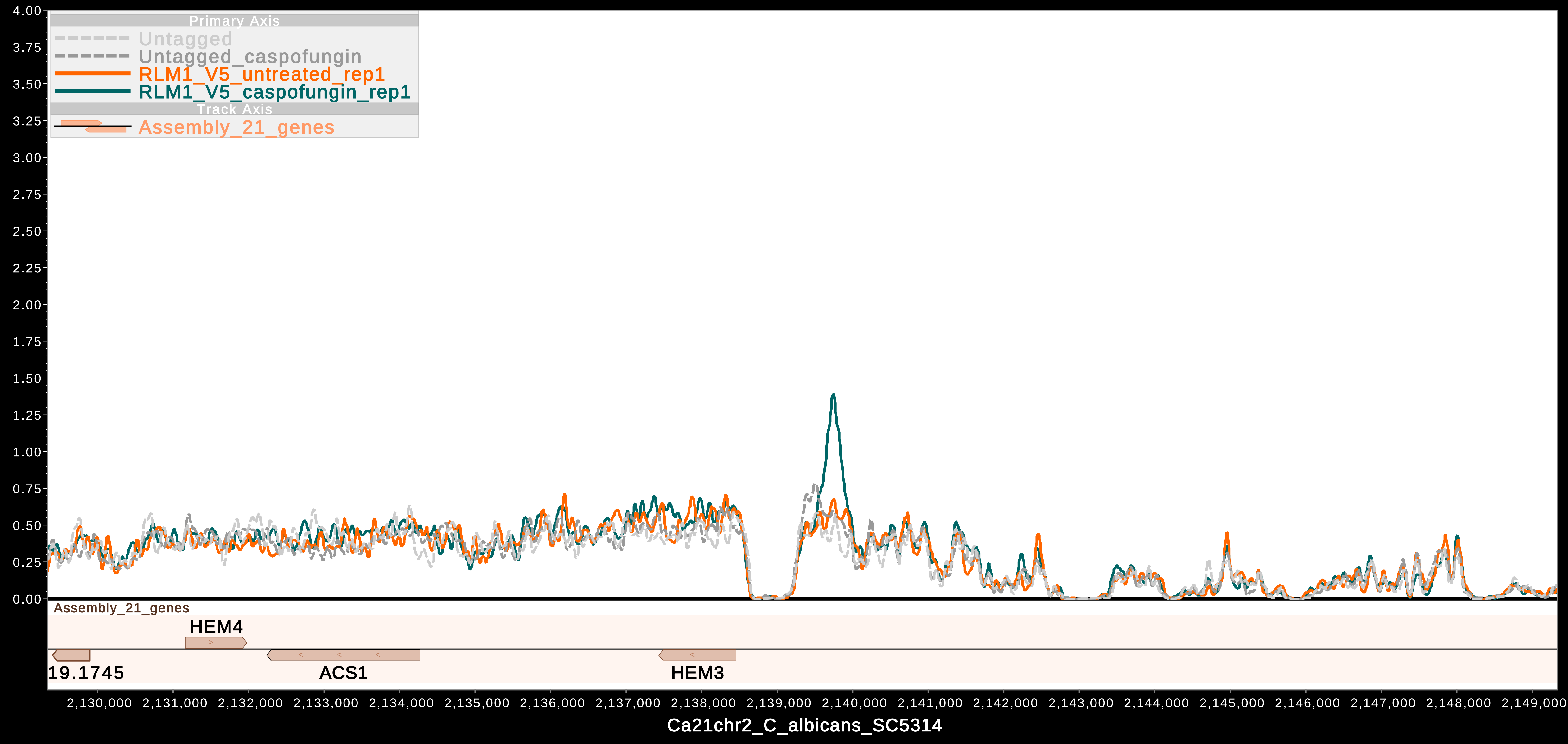

Primary Axis
Untagged
Untagged_caspofungin
RLM1_V5_untreated_rep1
RLM1_V5_caspofungin_rep1
Track Axis
Assembly_21_genes
4.00
3.75
3.50
3.25
3.00
2.75
2.50
2.25
2.00
1.75
1.50
1.25
1.00
0.75
0.50
0.25
0.00
Assembly_21_genes
HEM4
19.1745
ACS1
HEM3
2,130,000
2,131,000
2,132,000
2,133,000
2,134,000
2,135,000
2,136,000
2,137,000
2,138,000
2,139,000
2,140,000
2,141,000
2,142,000
2,143,000
2,144,000
2,145,000
2,146,000
2,147,000
2,148,000
2,149,000
Ca21chr2_C_albicans_SC5314

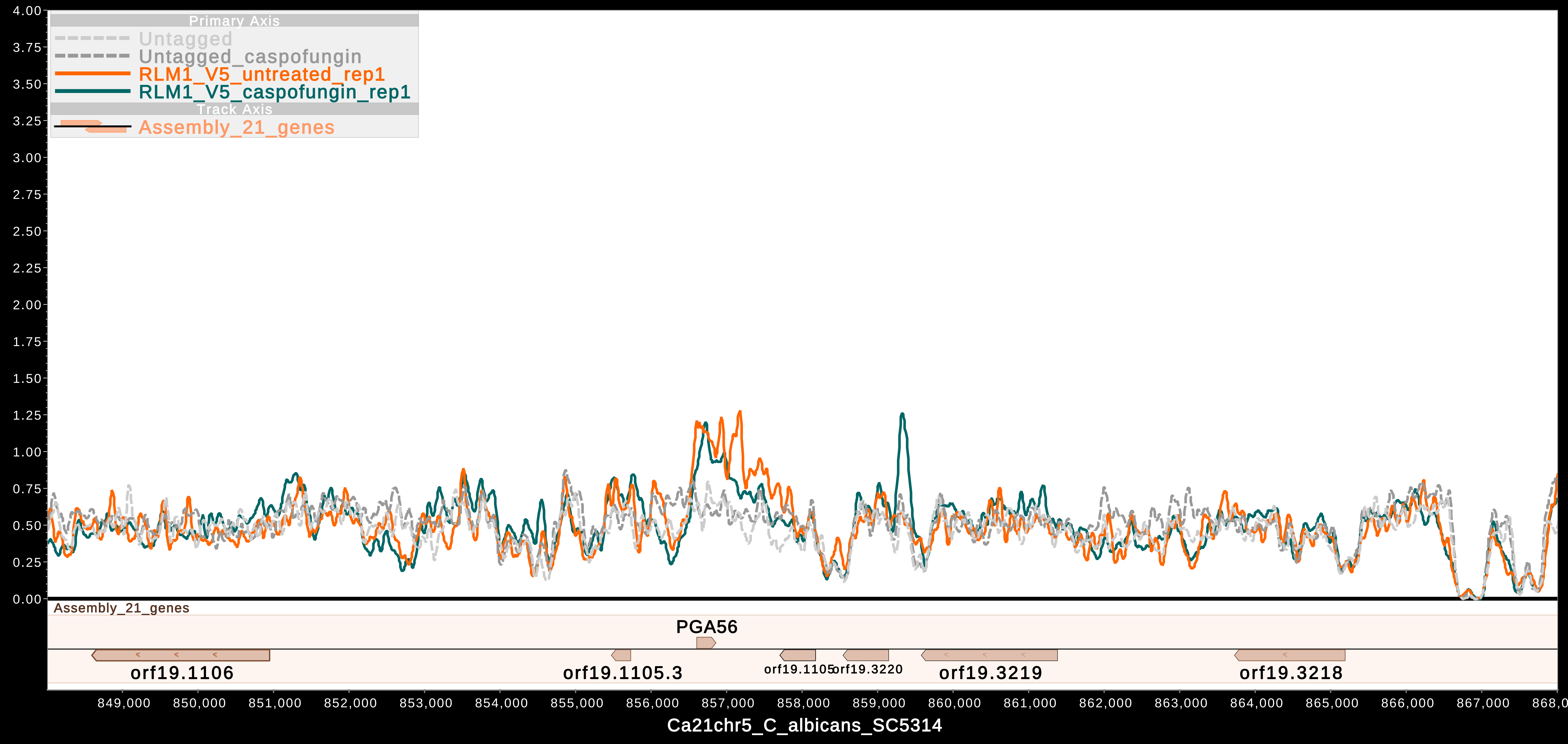

Primary Axis
Untagged
Untagged_caspofungin
RLM1_V5_untreated_rep1
RLM1_V5_caspofungin_rep1
Track Axis
Assembly_21_genes
4.00
3.75
3.50
3.25
3.00
2.75
2.50
2.25
2.00
1.75
1.50
1.25
1.00
0.75
0.50
0.25
0.00
Assembly_21_genes
PGA56
orf19.1106
orf19.1105.3
orf19.1105
orf19.3220
orf19.3219
orf19.3218
849,000
850,000
851,000
852,000
853,000
854,000
855,000
856,000
857,000
858,000
859,000
860,000
861,000
862,000
863,000
864,000
865,000
866,000
867,000
Ca21chr5_C_albicans_SC5314

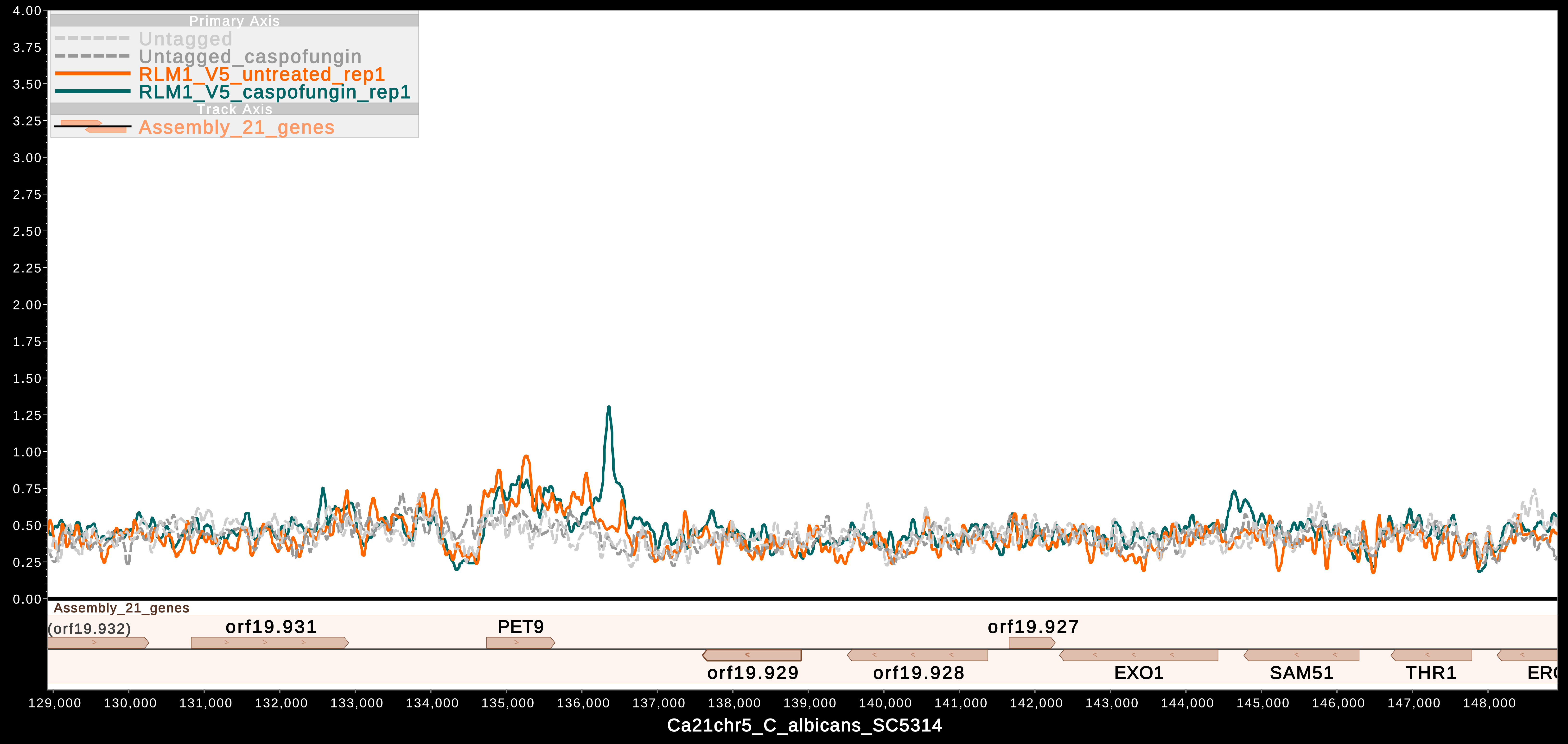

Primary Axis
Untagged
Untagged_caspofungin
RLM1_V5_untreated_rep1
RLM1_V5_caspofungin_rep1
Track Axis
Assembly_21_genes
4.00
3.75
3.50
3.25
3.00
2.75
2.50
2.25
2.00
1.75
1.50
1.25
1.00
0.75
0.50
0.25
0.00
Assembly_21_genes
(orf19.932)
orf19.931
PET9
orf19.927
orf19.929
orf19.928
EXO1
SAM51
THR1
129,000
130,000
131,000
132,000
133,000
134,000
135,000
136,000
137,000
138,000
139,000
140,000
141,000
142,000
143,000
144,000
145,000
146,000
147,000
148,000
Ca21chr5_C_albicans_SC5314

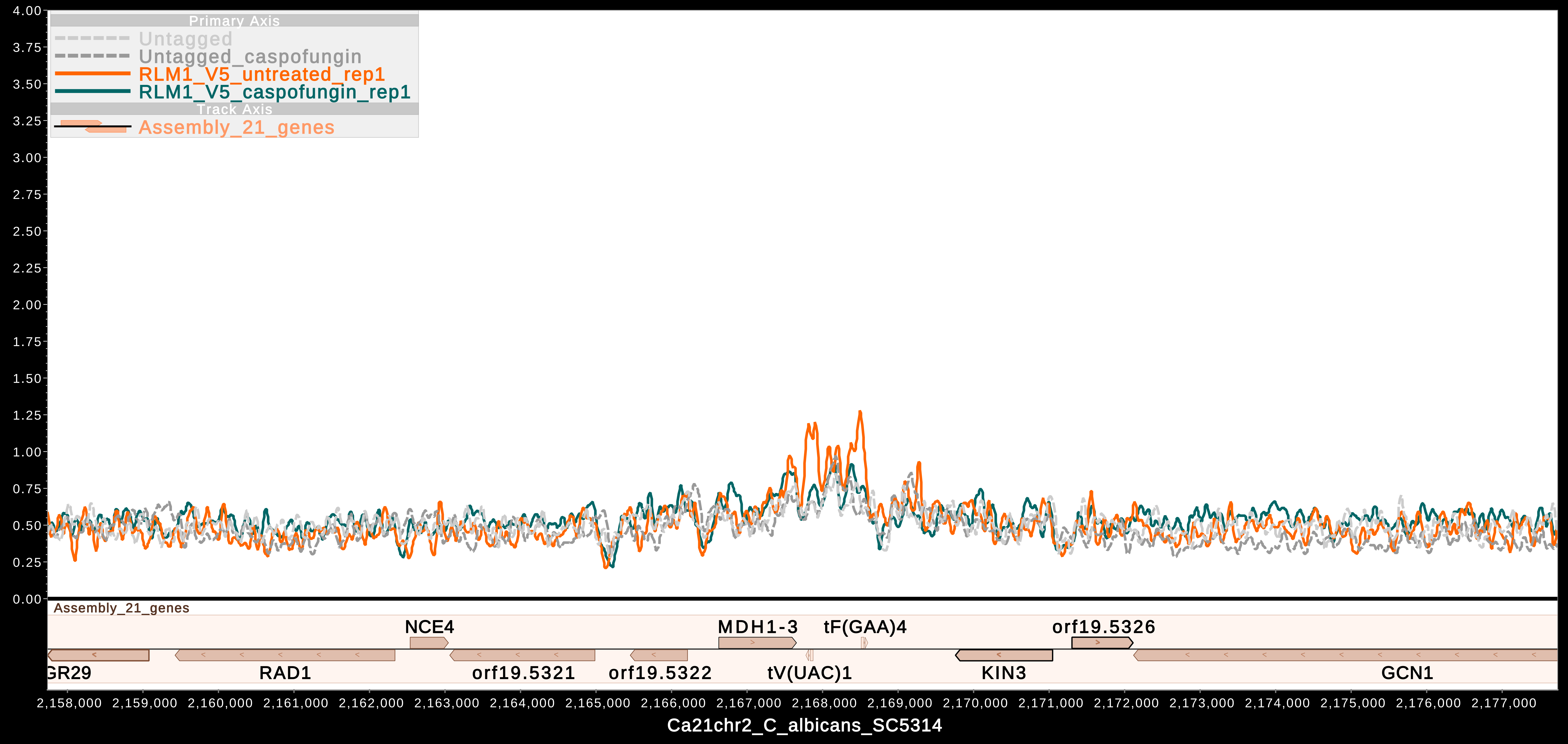
Primary Axis
Untagged
Untagged_caspofungin
RLM1_V5_untreated_rep1
RLM1_V5_caspofungin_rep1
Track Axis
Assembly_21_genes
4.00
3.75
3.50
3.25
3.00
2.75
2.50
2.25
2.00
1.75
1.50
1.25
1.00
0.75
0.50
0.25
0.00
Assembly_21_genes
NCE4
MDH1-3
tF(GAA)4
orf19.5326
GR29
RAD1
orf19.5321
orf19.5322
tV(UAC)1
KIN3
GCN1
2,158,000
2,159,000
2,160,000
2,161,000
2,162,000
2,163,000
2,164,000
2,165,000
2,166,000
2,167,000
2,168,000
2,169,000
2,170,000
2,171,000
2,172,000
2,173,000
2,174,000
2,175,000
2,176,000
2,177,000
Ca21chr2_C_albicans_SC5314

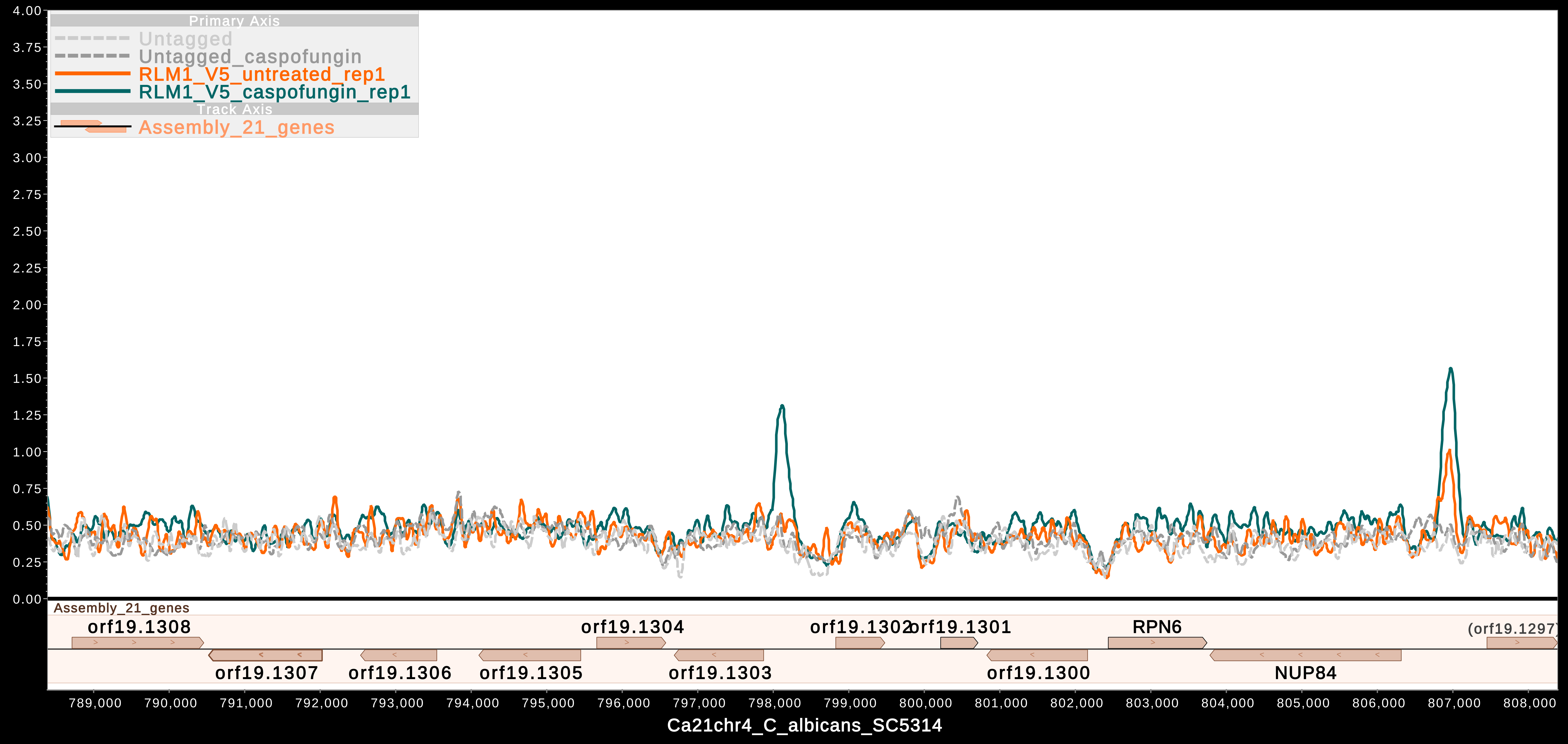
Primary Axis
Untagged
Untagged_caspofungin
RLM1_V5_untreated_rep1
RLM1_V5_caspofungin_rep1
Track Axis
Assembly_21_genes
4.00
3.75
3.50
3.25
3.00
2.75
2.50
2.25
2.00
1.75
1.50
1.25
1.00
0.75
0.50
0.25
0.00
Assembly_21_genes
orf19.1308
orf19.1304
orf19.1302
orf19.1301
RPN6
(orf19.1297)
orf19.1307
orf19.1306
orf19.1305
orf19.1303
orf19.1300
NUP84
789,000
790,000
791,000
792,000
793,000
794,000
795,000
796,000
797,000
798,000
799,000
800,000
801,000
802,000
803,000
804,000
805,000
806,000
807,000
808,000
Ca21chr4_C_albicans_SC5314

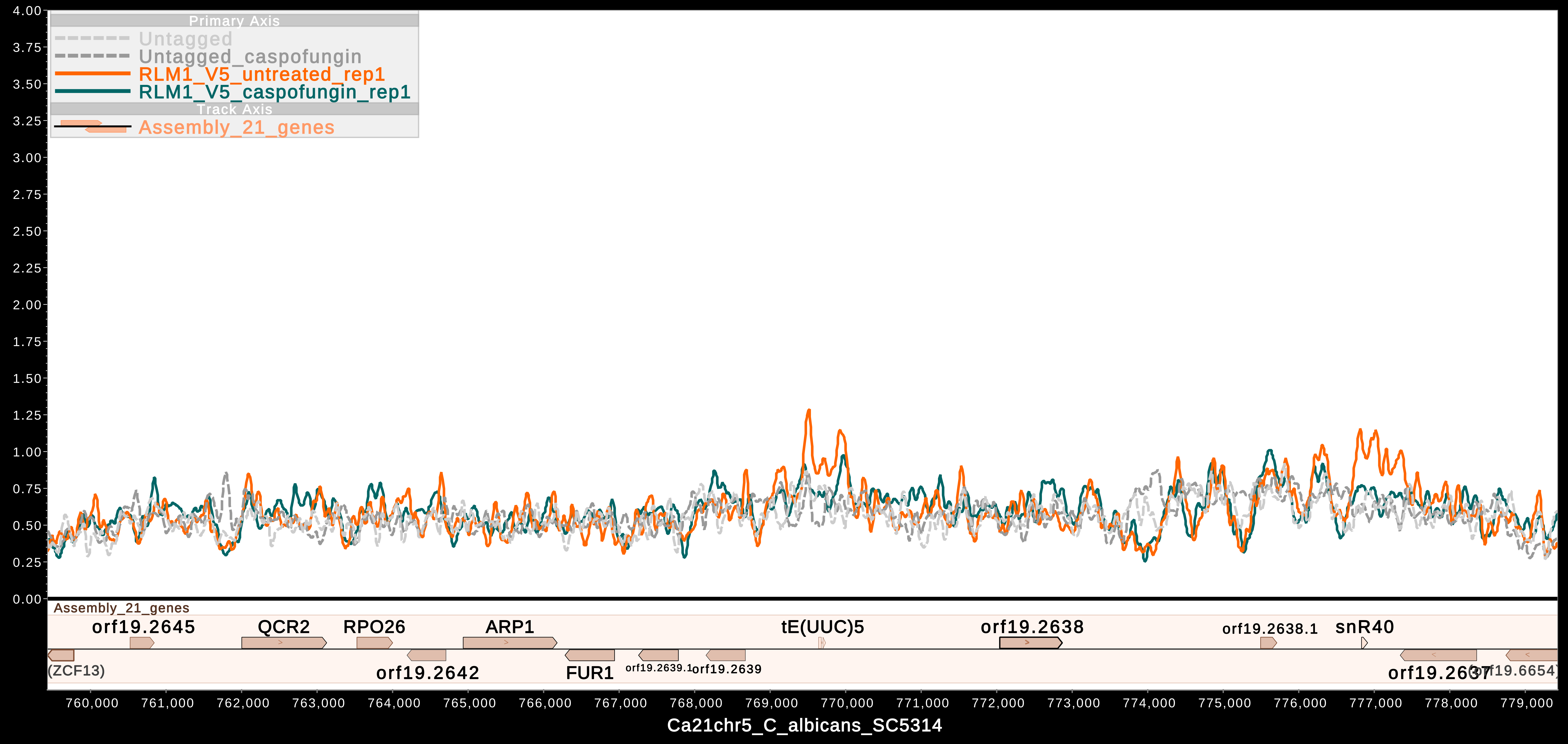
Primary Axis
Untagged
Untagged_caspofungin
RLM1_V5_untreated_rep1
RLM1_V5_caspofungin_rep1
Track Axis
Assembly_21_genes
4.00
3.75
3.50
3.25
3.00
2.75
2.50
2.25
2.00
1.75
1.50
1.25
1.00
0.75
0.50
0.25
0.00
Assembly_21_genes
orf19.2645
QCR2
RPO26
ARP1
tE(UUC)5
orf19.2638
orf19.2638.1
snR40
(ZCF13)
orf19.2642
FUR1
orf19.2639.1
orf19.2639
orf19.2637
760,000
761,000
762,000
763,000
764,000
765,000
766,000
767,000
768,000
769,000
770,000
771,000
772,000
773,000
774,000
775,000
776,000
777,000
778,000
779,000
Ca21chr5_C_albicans_SC5314

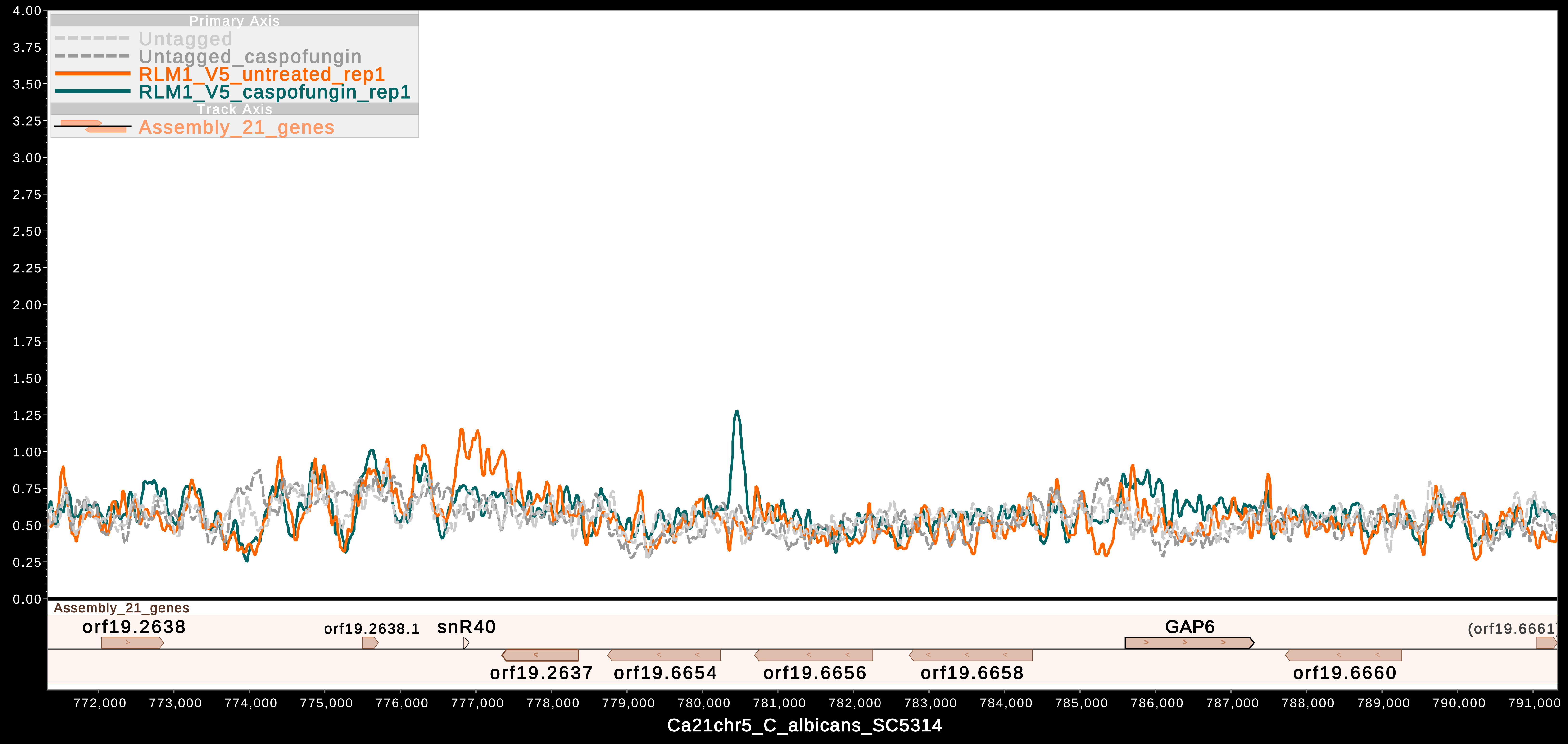

Primary Axis
Untagged
Untagged_caspofungin
RLM1_V5_untreated_rep1
RLM1_V5_caspofungin_rep1
Track Axis
Assembly_21_genes
4.00
3.75
3.50
3.25
3.00
2.75
2.50
2.25
2.00
1.75
1.50
1.25
1.00
0.75
0.50
0.25
0.00
Assembly_21_genes
orf19.2638
orf19.2638.1
snR40
GAP6
(orf19.6661)
orf19.2637
orf19.6654
orf19.6656
orf19.6658
orf19.6660
772,000
773,000
774,000
775,000
776,000
777,000
778,000
779,000
780,000
781,000
782,000
783,000
784,000
785,000
786,000
787,000
788,000
789,000
790,000
791,000
Ca21chr5_C_albicans_SC5314

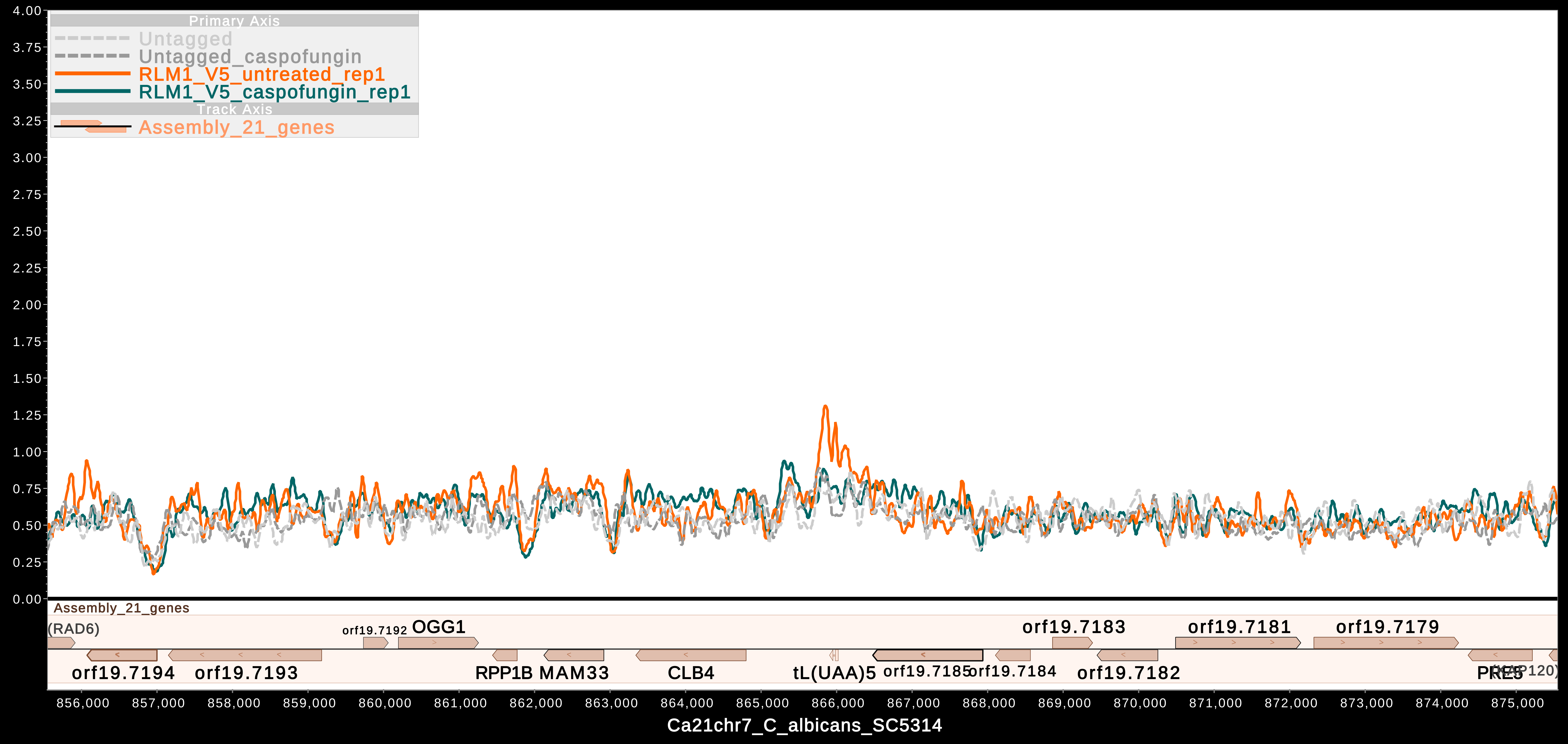

Primary Axis
Untagged
Untagged_caspofungin
RLM1_V5_untreated_rep1
RLM1_V5_caspofungin_rep1
Track Axis
Assembly_21_genes
4.00
3.75
3.50
3.25
3.00
2.75
2.50
2.25
2.00
1.75
1.50
1.25
1.00
0.75
0.50
0.25
0.00
Assembly_21_genes
(RAD6)
orf19.7192
OGG1
orf19.7183
orf19.7181
orf19.7179
orf19.7194
orf19.7193
RPP1B
MAM33
CLB4
tL(UAA)5
orf19.7185
orf19.7184
orf19.7182
PRE5
(MAP120)
856,000
857,000
858,000
859,000
860,000
861,000
862,000
863,000
864,000
865,000
866,000
867,000
868,000
869,000
870,000
871,000
872,000
873,000
874,000
875,000
Ca21chr7_C_albicans_SC5314

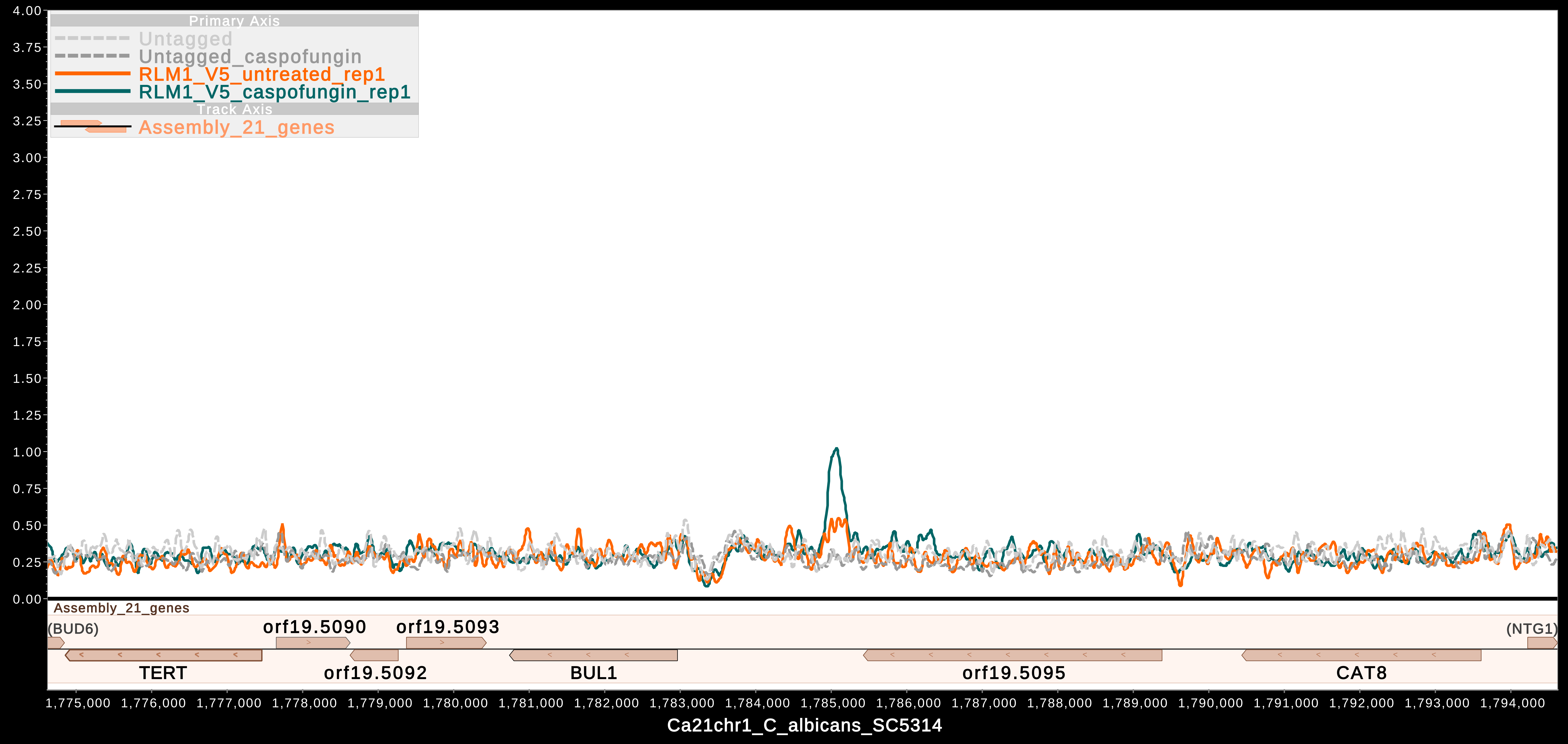
Primary Axis
Untagged
Untagged_caspofungin
RLM1_V5_untreated_rep1
RLM1_V5_caspofungin_rep1
Track Axis
Assembly_21_genes
4.00
3.75
3.50
3.25
3.00
2.75
2.50
2.25
2.00
1.75
1.50
1.25
1.00
0.75
0.50
0.25
0.00
Assembly_21_genes
(BUD6)
orf19.5090
orf19.5093
(NTG1)
TERT
orf19.5092
BUL1
orf19.5095
CAT8
1,775,000
1,776,000
1,777,000
1,778,000
1,779,000
1,780,000
1,781,000
1,782,000
1,783,000
1,784,000
1,785,000
1,786,000
1,787,000
1,788,000
1,789,000
1,790,000
1,791,000
1,792,000
1,793,000
1,794,000
Ca21chr1_C_albicans_SC5314

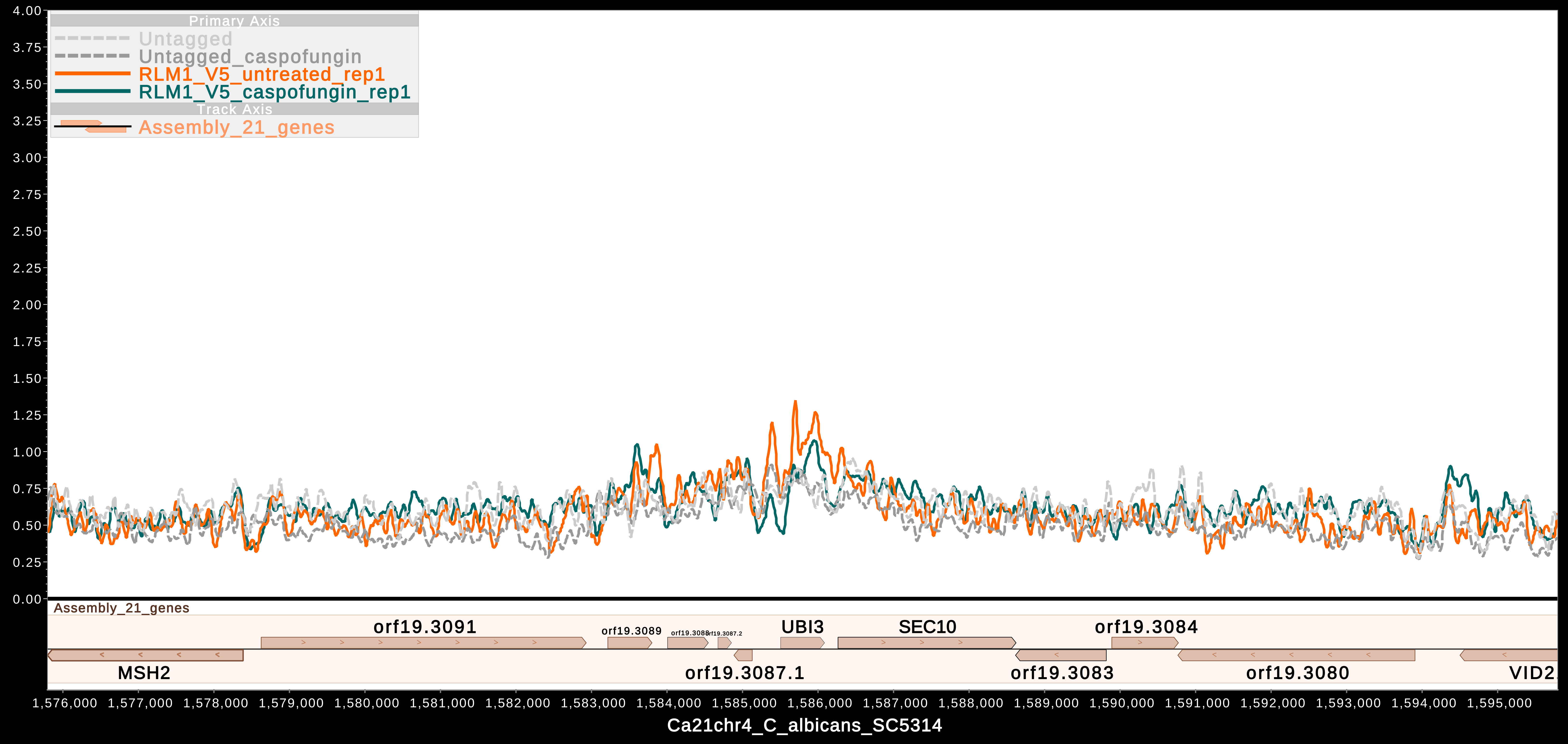
Primary Axis
Untagged
Untagged_caspofungin
RLM1_V5_untreated_rep1
RLM1_V5_caspofungin_rep1
Track Axis
Assembly_21_genes
4.00
3.75
3.50
3.25
3.00
2.75
2.50
2.25
2.00
1.75
1.50
1.25
1.00
0.75
0.50
0.25
0.00
Assembly_21_genes
orf19.3091
orf19.3089
orf19.3088
UBI3
SEC10
orf19.3084
MSH2
orf19.3087.1
orf19.3083
orf19.3080
VID2
1,576,000 1,577,000 1,578,000 1,579,000 1,580,000 1,581,000 1,582,000 1,583,000 1,584,000 1,585,000 1,586,000 1,587,000 1,588,000 1,589,000 1,590,000 1,591,000 1,592,000 1,593,000 1,594,000 1,595,000
Ca21chr4_C_albicans_SC5314

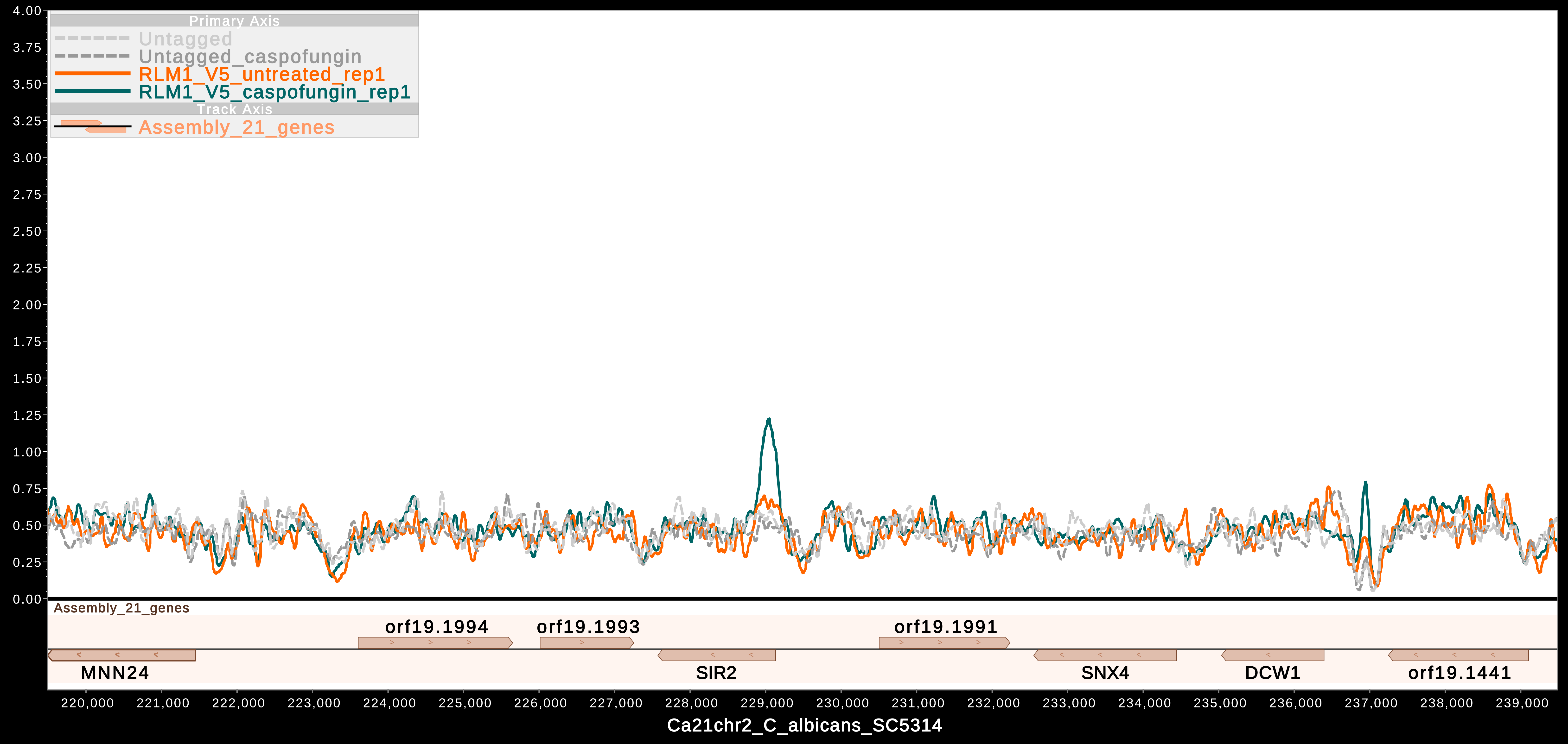
Primary Axis
Untagged
Untagged_caspofungin
RLM1_V5_untreated_rep1
RLM1_V5_caspofungin_rep1
Track Axis
Assembly_21_genes
4.00
3.75
3.50
3.25
3.00
2.75
2.50
2.25
2.00
1.75
1.50
1.25
1.00
0.75
0.50
0.25
0.00
Assembly_21_genes
orf19.1994
orf19.1993
orf19.1991
MNN24
SIR2
SNX4
DCW1
orf19.1441
220,000
221,000
222,000
223,000
224,000
225,000
226,000
227,000
228,000
229,000
230,000
231,000
232,000
233,000
234,000
235,000
236,000
237,000
238,000
239,000
Ca21chr2_C_albicans_SC5314
