# Supplementary material for: An expanded cell wall damage signaling network is comprised of the transcription factors Rlm1 and Sko1 in Candida albicans
Source: PLoS Genet. 2020 Jul 8;16(7):e1008908. doi: 10.1371/journal.pgen.1008908 (PMC7371209; doi:10.1371/journal.pgen.1008908)
Supplement: S2 Appendix — Genes are listed in descending order for Sko1 upstream intergenic region enrichment. (PDF) [file pgen.1008908.s007.pdf]

ORF Number	Gene	Page in S2 Appendix
orf19.6484	*ORF19.6484*	2
orf19.6486	*LDG3*	2
orf19.6487	*ORF19.6487*	2
orf19.1011	*MNN21*	3
orf19.5288.1	*ORF19.5288.1*	3
orf19.5741	*ALS1*	4
orf19.5045	*PTP2*	5
orf19.5798	*LIG4*	6
orf19.5274	*ORF19.5274*	7
orf19.450	*ORF19.450*	8
orf19.1065	*SSA2*	9
orf19.6547	*ORF19.6547*	10
orf19.6548	*ISU1*	10
orf19.1032	*SKO1*	11
orf19.1033	*STR2*	11
orf19.2896	*SOU1*	12
orf19.3971	*ORF19.3971*	13
orf19.5302	*PGA31*	14
orf19.5305	*RHD3*	14
orf19.5437	*RHR2*	15
orf19.5438	*ORF19.5438*	15
orf19.4076	*MET10*	16
orf19.4077	*MIT1*	16
orf19.2075	*DFG5*	17
orf19.5170	*ENA21*	18
orf19.5171	*PMT1*	18
orf19.7544	*TLO1*	19
orf19.5302	*PGA31*	20
orf19.5305	*RHD3*	20
snR52	*SNR52*	21
orf19.6736	*ORF19.6736*	21
orf19.2781	*ORF19.2781*	22
orf19.778	*PIL1*	23
orf19.4679	*AGP2*	24
orf19.6163	*CSE4*	25
orf19.6160	*ORF19.6160*	25
orf19.1959	*ORF19.1959*	26
orf19.5069	*ORF19.5069*	27
orf19.7150	*NRG1*	28
orf19.7152	*ORF19.7152*	28
orf19.515	*ORF19.515*	29
orf19.7186	*CLB4*	30
orf19.7183	*ORF19.7183*	30
orf19.7193	*ORF19.7193*	30
orf19.1440.2	*ORF19.1440.2*	31
orf19.7150	*NRG1*	32
orf19.2699	*ABP1*	33
orf19.2701	*ORF19.2701*	33
orf19.20	*RTS1*	34
orf19.610	*EFG1*	35
orf19.7592	*FAA4*	36
orf19.691	*GPD2*	37
orf19.5447	*HGT19*	38
orf19.5449	*ORF19.5449*	38
orf19.2726	*ORF19.2726*	39
orf19.4081	*ORF19.4081*	40
orf19.5322	*ORF19.5322*	41
orf19.7506	*ORF19.7506*	42
orf19.7077	*ORF19.7077*	43
orf19.4443	*YPD1*	44
orf19.3689	*ORF19.3689*	45
orf19.3690.2	*ORF19.3690.2*	45
orf19.3691	*TIM21*	45
orf19.2178.1	*ORF19.2178.1*	46
orf19.3997	*ADH1*	47
orf19.3999	*ORF19.3999*	47
orf19.954	*ORF19.954*	48
orf19.2943.5	*ORF19.2943.5*	49
orf19.2760	*ORF19.2760*	50
orf19.2765	*PGA62*	50
orf19.2766	*ORF19.2766*	50
orf19.2767	*PGA59*	50
orf19.3086	*SEC10*	51
orf19.3087	*UBI3*	51
orf19.7380	*ORF19.7380*	52
orf19.7381	*AHR1*	52
orf19.7186	*CLB4*	53
orf19.7183	*ORF19.7183*	53
orf19.7193	*ORF19.7193*	53
orf19.319	*ORF19.319*	54
orf19.1634	*ORF19.1634*	55
orf19.4246	*ORF19.4246*	56
orf19.4666	*ORF19.4666*	57
orf19.5284	*ORF19.5284*	58
orf19.2309	*PET127*	59
orf19.6863	*VPH1*	60
orf19.6864	*ORF19.6864*	60
orf19.3059	*SUA71*	61
orf19.3059.1	*ORF19.3059.1*	61
orf19.1906	*ORF19.1906*	62
orf19.837	*GNA1*	63
orf19.3661	*ORF19.3661*	64
orf19.4309	*GRP2*	65
orf19.1189	*ORF19.1189*	66
orf19.2529.1	*ORF19.2529.1*	67
orf19.2531	*CSP37*	67
orf19.2638.1	*ORF19.2638.1*	68
orf19.7115	*SAC7*	69
orf19.7222	*PAM16*	70
orf19.7223	*ORF19.7223*	70
orf19.1105.2	*PGA56*	71
orf19.1105.3	*ORF19.1105.3*	71
orf19.6081	*PHR2*	72
orf19.6082	*ORF19.6082*	72
orf19.6090	*ORF19.6090*	72
orf19.847	*YIM1*	73
orf19.3689	*ORF19.3689*	74
orf19.3690.2	*ORF19.3690.2*	74
orf19.3691	*TIM21*	74
orf19.6734	*TCC1*	75
orf19.24	*RTA2*	76
orf19.3335	*ORF19.3335*	77
orf19.6873	*RPS8A*	78
orf19.7013	*ORF19.7013*	79
orf19.7014	*ORF19.7014*	79
orf19.3869	*ORF19.3869*	80
orf19.7195	*RAD6*	81

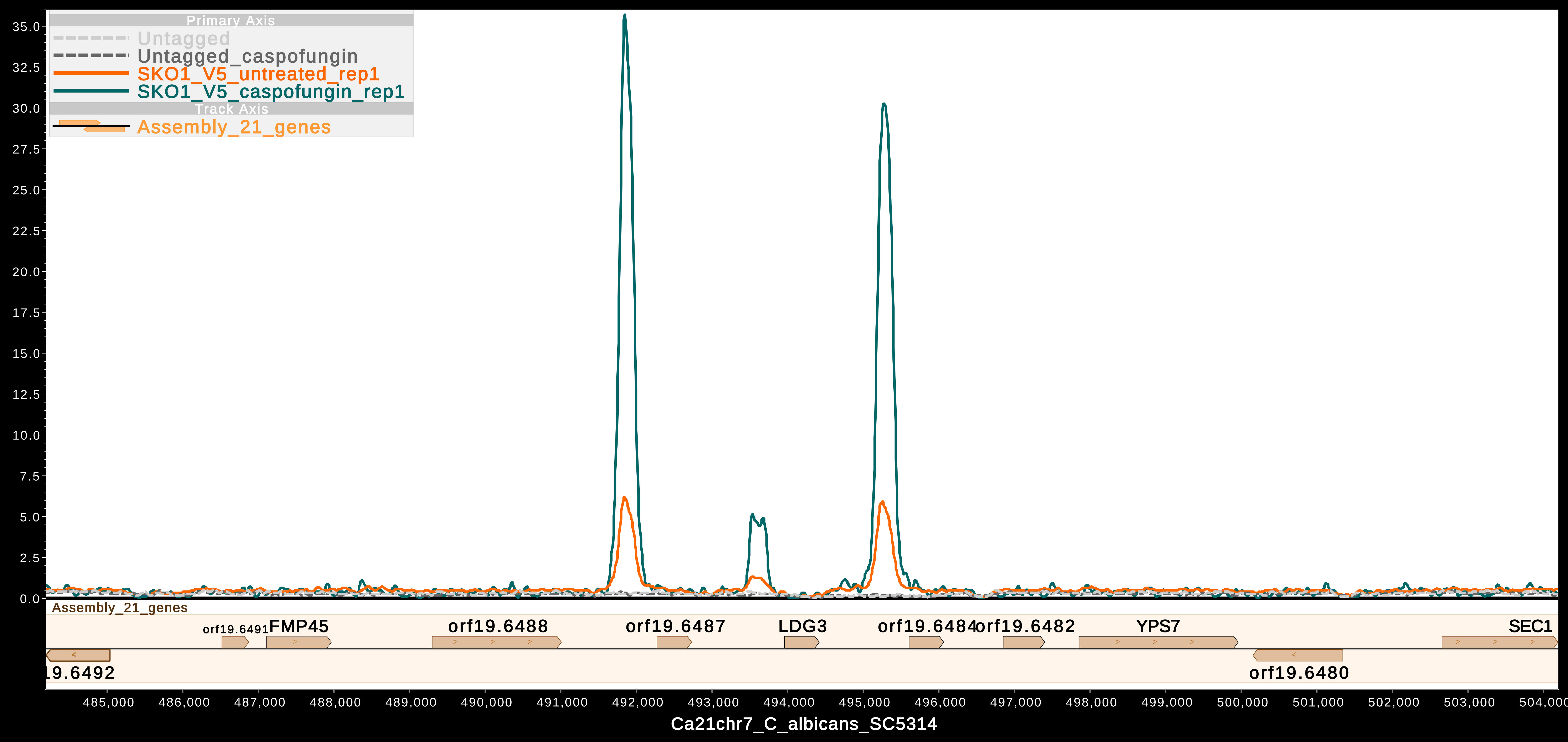

Primary Axis
Untagged
Untagged_caspofungin
SKO1_V5_untreated_rep1
SKO1_V5_caspofungin_rep1
Track Axis
Assembly_21_genes
35.0
32.5
30.0
27.5
25.0
22.5
20.0
17.5
15.0
12.5
10.0
7.5
5.0
2.5
0.0
Assembly_21_genes
orf19.6491
FMP45
orf19.6488
orf19.6487
LDG3
orf19.6484
orf19.6482
YPS7
SEC1
19.6492
orf19.6480
485,000
486,000
487,000
488,000
489,000
490,000
491,000
492,000
493,000
494,000
495,000
496,000
497,000
498,000
499,000
500,000
501,000
502,000
503,000
504,000
Ca21chr7_C_albicans_SC5314

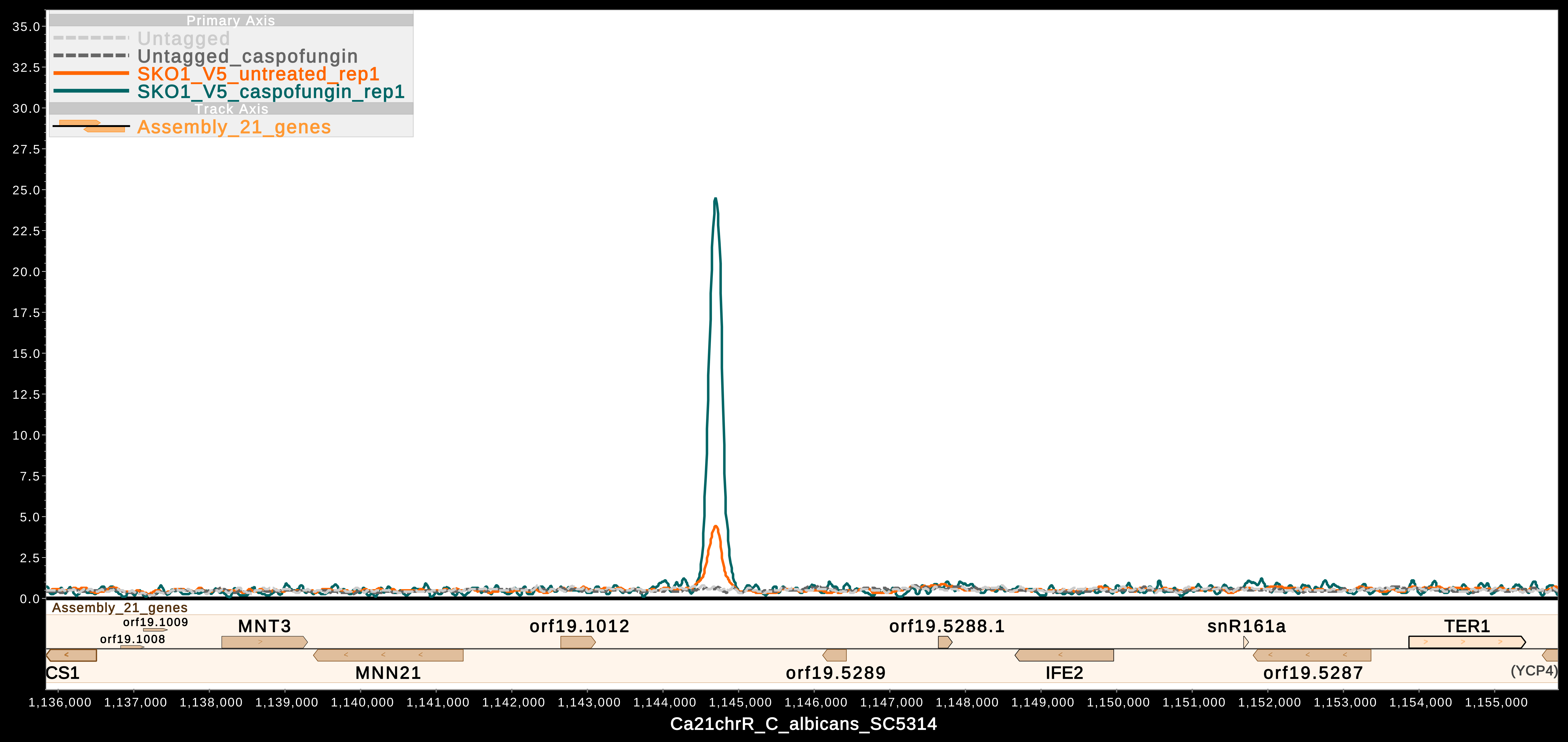
Primary Axis
Untagged
Untagged_caspofungin
SKO1_V5_untreated_rep1
SKO1_V5_caspofungin_rep1
Track Axis
Assembly_21_genes
35.0
32.5
30.0
27.5
25.0
22.5
20.0
17.5
15.0
12.5
10.0
7.5
5.0
2.5
0.0
Assembly_21_genes
orf19.1009
orf19.1008
MNT3
orf19.1012
orf19.5288.1
snR161a
TER1
CS1
MNN21
orf19.5289
IFE2
orf19.5287
(YCP4)
1,136,000
1,137,000
1,138,000
1,139,000
1,140,000
1,141,000
1,142,000
1,143,000
1,144,000
1,145,000
1,146,000
1,147,000
1,148,000
1,149,000
1,150,000
1,151,000
1,152,000
1,153,000
1,154,000
1,155,000
Ca21chrR_C_albicans_SC5314

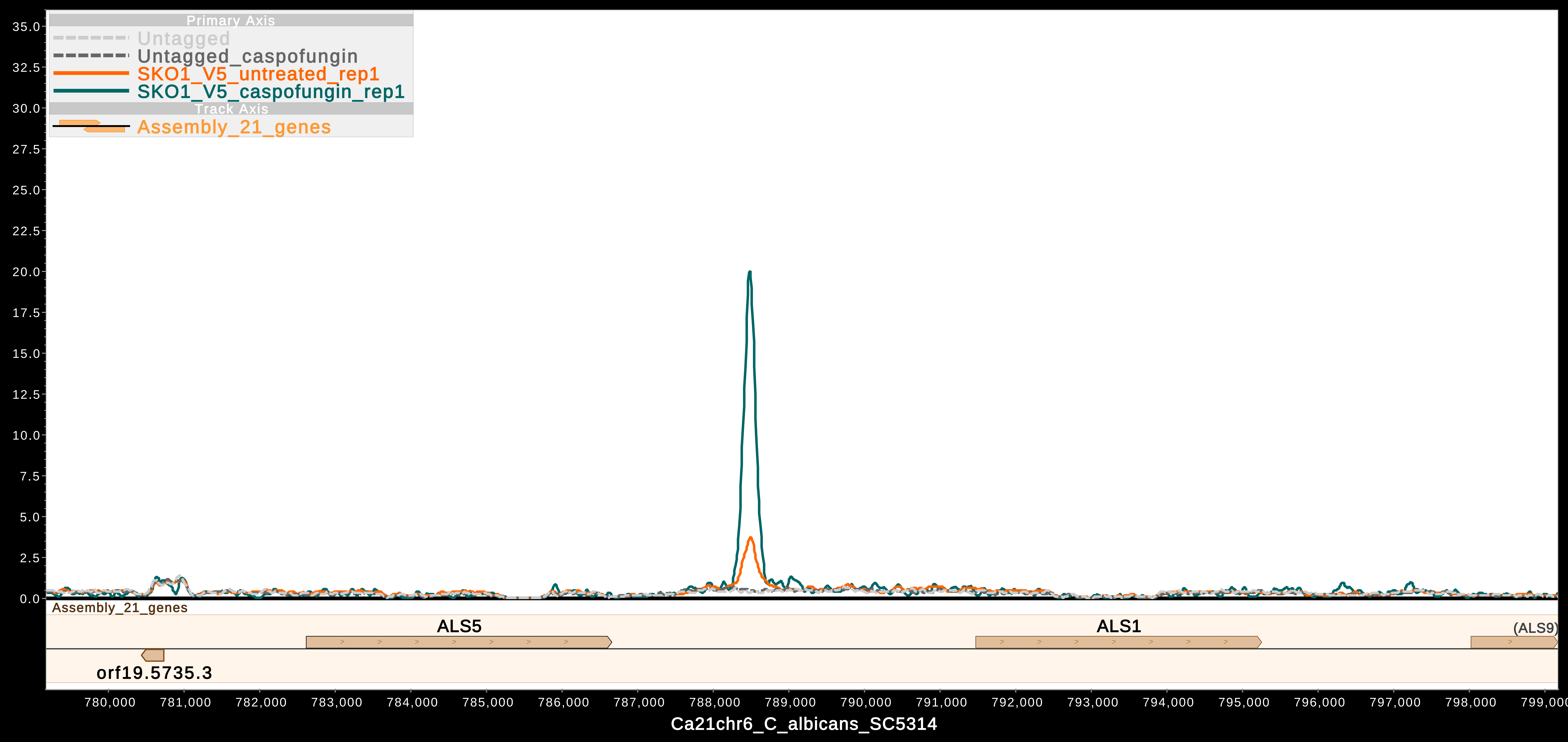

Primary Axis
Untagged
Untagged_caspofungin
SKO1_V5_untreated_rep1
SKO1_V5_caspofungin_rep1
Track Axis
Assembly_21_genes
35.0
32.5
30.0
27.5
25.0
22.5
20.0
17.5
15.0
12.5
10.0
7.5
5.0
2.5
0.0
Assembly_21_genes
ALS5
ALS1
(ALS9)
orf19.5735.3
780,000
781,000
782,000
783,000
784,000
785,000
786,000
787,000
788,000
789,000
790,000
791,000
792,000
793,000
794,000
795,000
796,000
797,000
798,000
Ca21chr6_C_albicans_SC5314

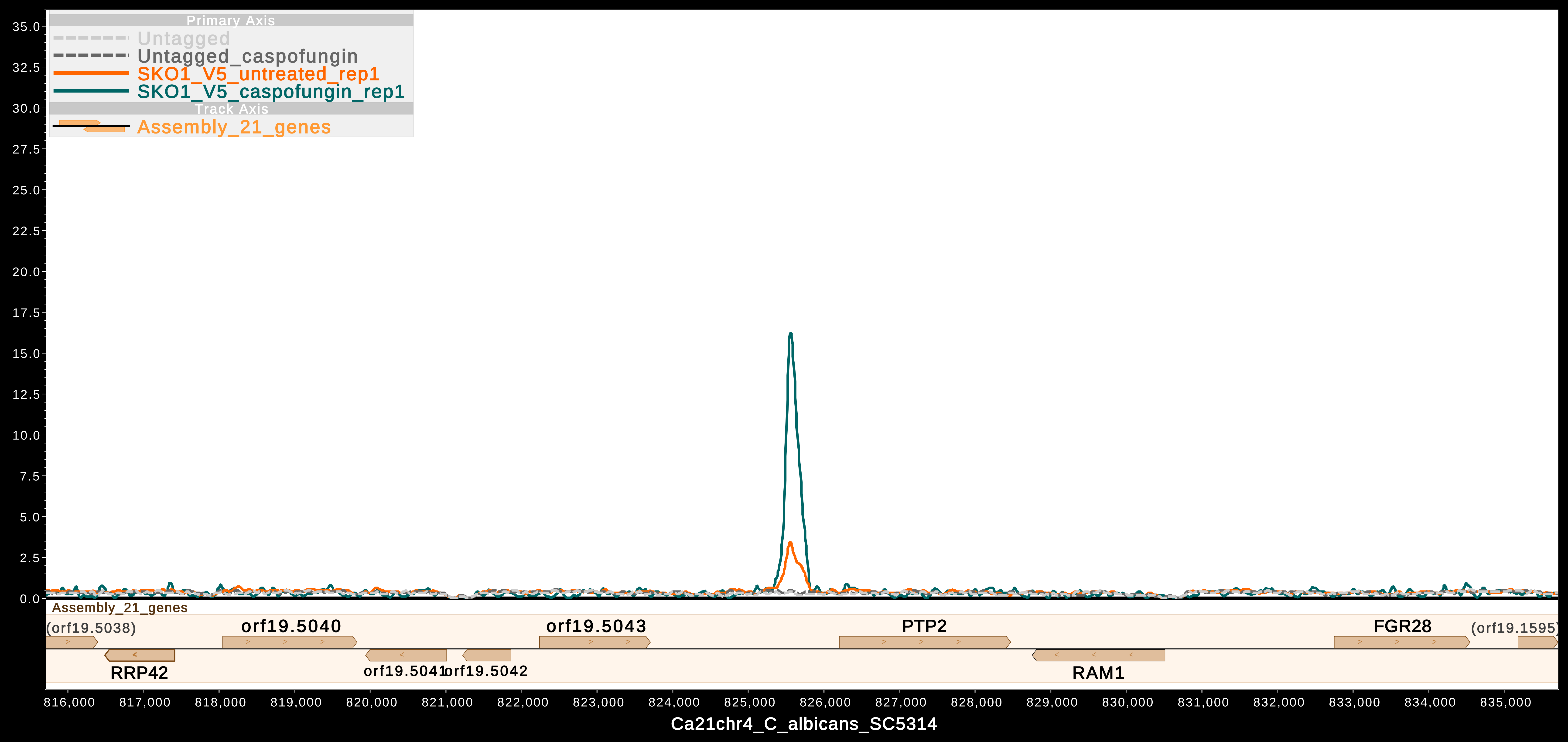

Primary Axis
Untagged
Untagged_caspofungin
SKO1_V5_untreated_rep1
SKO1_V5_caspofungin_rep1
Track Axis
Assembly_21_genes
35.0
32.5
30.0
27.5
25.0
22.5
20.0
17.5
15.0
12.5
10.0
7.5
5.0
2.5
0.0
Assembly_21_genes
(orf19.5038)
orf19.5040
orf19.5043
PTP2
FGR28
(orf19.1595)
RRP42
orf19.5041
orf19.5042
RAM1
816,000
817,000
818,000
819,000
820,000
821,000
822,000
823,000
824,000
825,000
826,000
827,000
828,000
829,000
830,000
831,000
832,000
833,000
834,000
835,000
Ca21chr4_C_albicans_SC5314

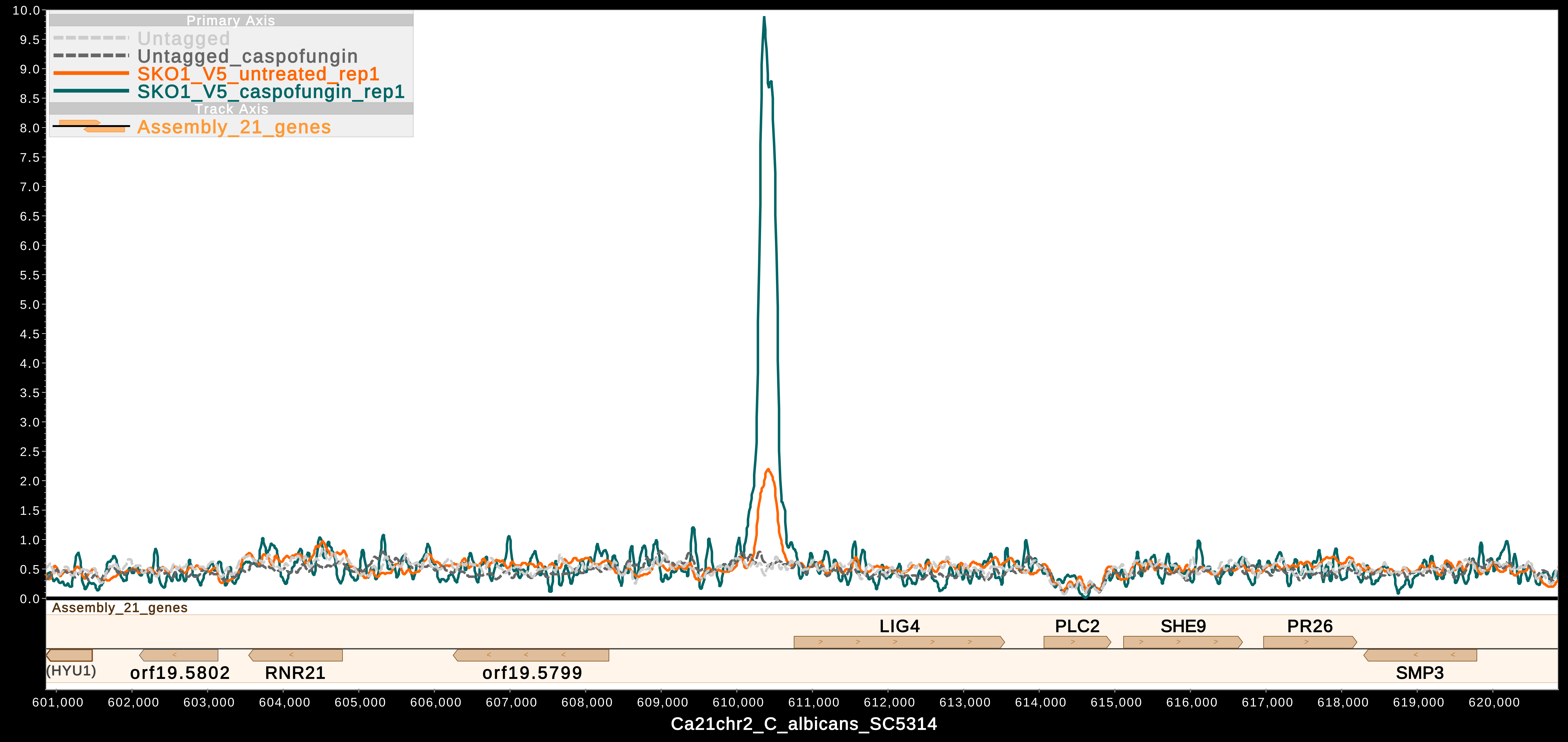

Primary Axis
Untagged
Untagged_caspofungin
SKO1_V5_untreated_rep1
SKO1_V5_caspofungin_rep1
Track Axis
Assembly_21_genes
10.0
9.5
9.0
8.5
8.0
7.5
7.0
6.5
6.0
5.5
5.0
4.5
4.0
3.5
3.0
2.5
2.0
1.5
1.0
0.5
0.0
Assembly_21_genes
LIG4
PLC2
SHE9
PR26
(HYU1)
orf19.5802
RNR21
orf19.5799
SMP3
601,000
602,000
603,000
604,000
605,000
606,000
607,000
608,000
609,000
610,000
611,000
612,000
613,000
614,000
615,000
616,000
617,000
618,000
619,000
620,000
Ca21chr2_C_albicans_SC5314

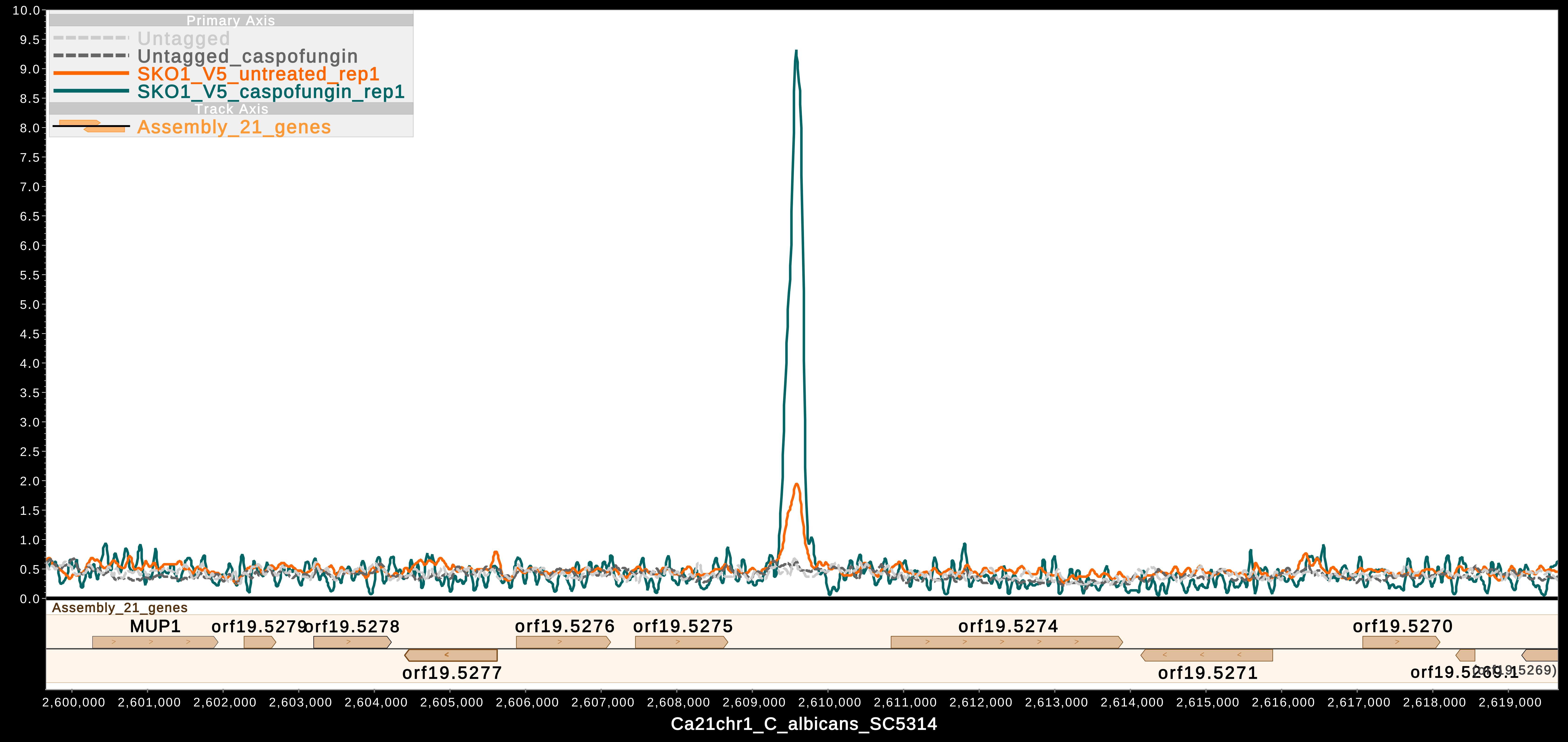

Primary Axis
Untagged
Untagged_caspofungin
SKO1_V5_untreated_rep1
SKO1_V5_caspofungin_rep1
Track Axis
Assembly_21_genes
10.0
9.5
9.0
8.5
8.0
7.5
7.0
6.5
6.0
5.5
5.0
4.5
4.0
3.5
3.0
2.5
2.0
1.5
1.0
0.5
0.0
Assembly_21_genes
MUP1
orf19.5279
orf19.5278
orf19.5276
orf19.5275
orf19.5274
orf19.5270
orf19.5277
orf19.5271
orf19.5269.1
(orf19.5269)
2,600,000
2,601,000
2,602,000
2,603,000
2,604,000
2,605,000
2,606,000
2,607,000
2,608,000
2,609,000
2,610,000
2,611,000
2,612,000
2,613,000
2,614,000
2,615,000
2,616,000
2,617,000
2,618,000
2,619,000
Ca21chr1_C_albicans_SC5314

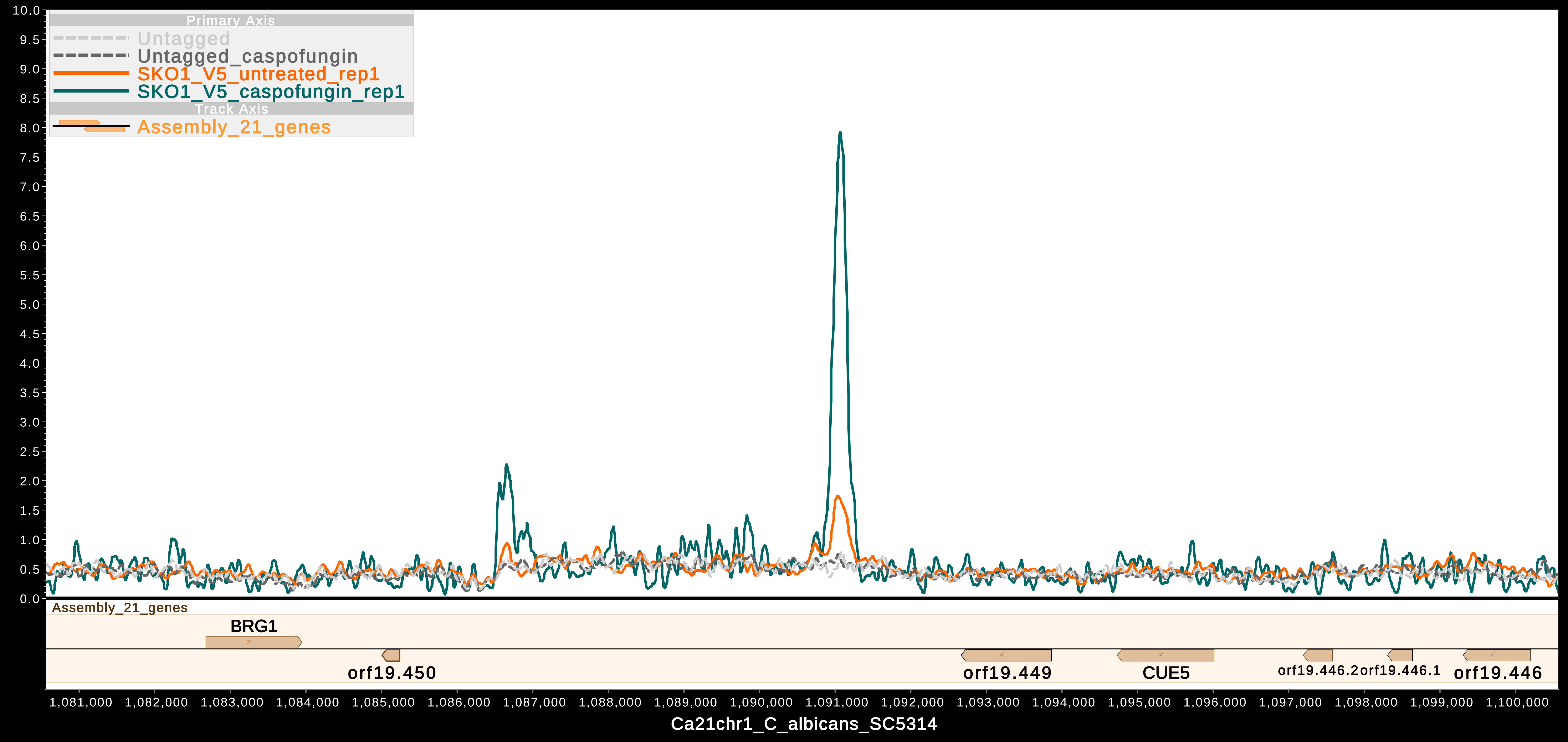

Primary Axis
Untagged
Untagged_caspofungin
SKO1_V5_untreated_rep1
SKO1_V5_caspofungin_rep1
Track Axis
Assembly_21_genes
10.0
9.5
9.0
8.5
8.0
7.5
7.0
6.5
6.0
5.5
5.0
4.5
4.0
3.5
3.0
2.5
2.0
1.5
1.0
0.5
0.0
Assembly_21_genes
BRG1
orf19.450
orf19.449
CUE5
orf19.446.2
orf19.446.1
orf19.446
1,081,000
1,082,000
1,083,000
1,084,000
1,085,000
1,086,000
1,087,000
1,088,000
1,089,000
1,090,000
1,091,000
1,092,000
1,093,000
1,094,000
1,095,000
1,096,000
1,097,000
1,098,000
1,099,000
1,100,000
Ca21chr1_C_albicans_SC5314

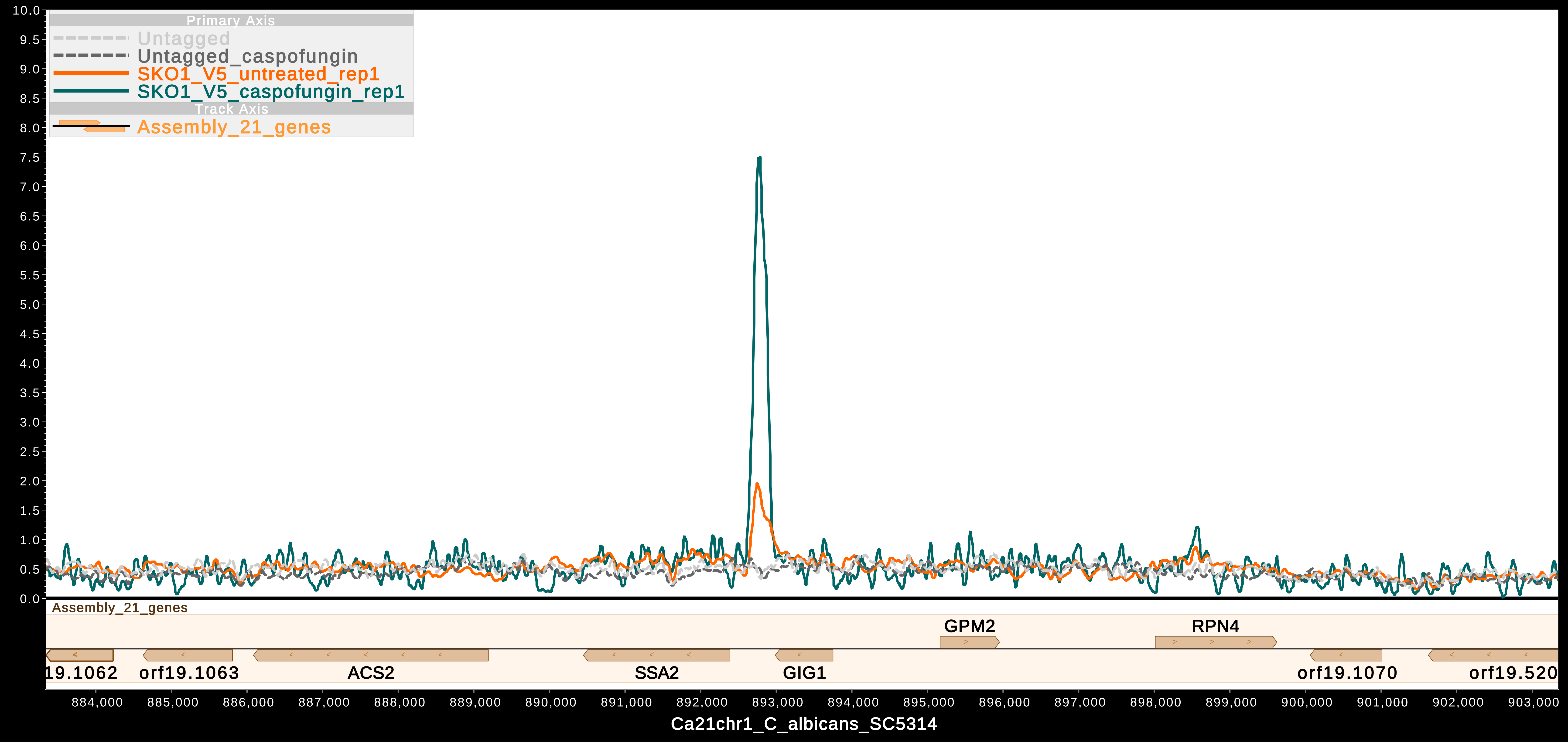

Primary Axis
Untagged
Untagged_caspofungin
SKO1_V5_untreated_rep1
SKO1_V5_caspofungin_rep1
Track Axis
Assembly_21_genes
10.0
9.5
9.0
8.5
8.0
7.5
7.0
6.5
6.0
5.5
5.0
4.5
4.0
3.5
3.0
2.5
2.0
1.5
1.0
0.5
0.0
Assembly_21_genes
GPM2
RPN4
19.1062
orf19.1063
ACS2
SSA2
GIG1
orf19.1070
orf19.520
884,000
885,000
886,000
887,000
888,000
889,000
890,000
891,000
892,000
893,000
894,000
895,000
896,000
897,000
898,000
899,000
900,000
901,000
902,000
903,000
Ca21chr1_C_albicans_SC5314

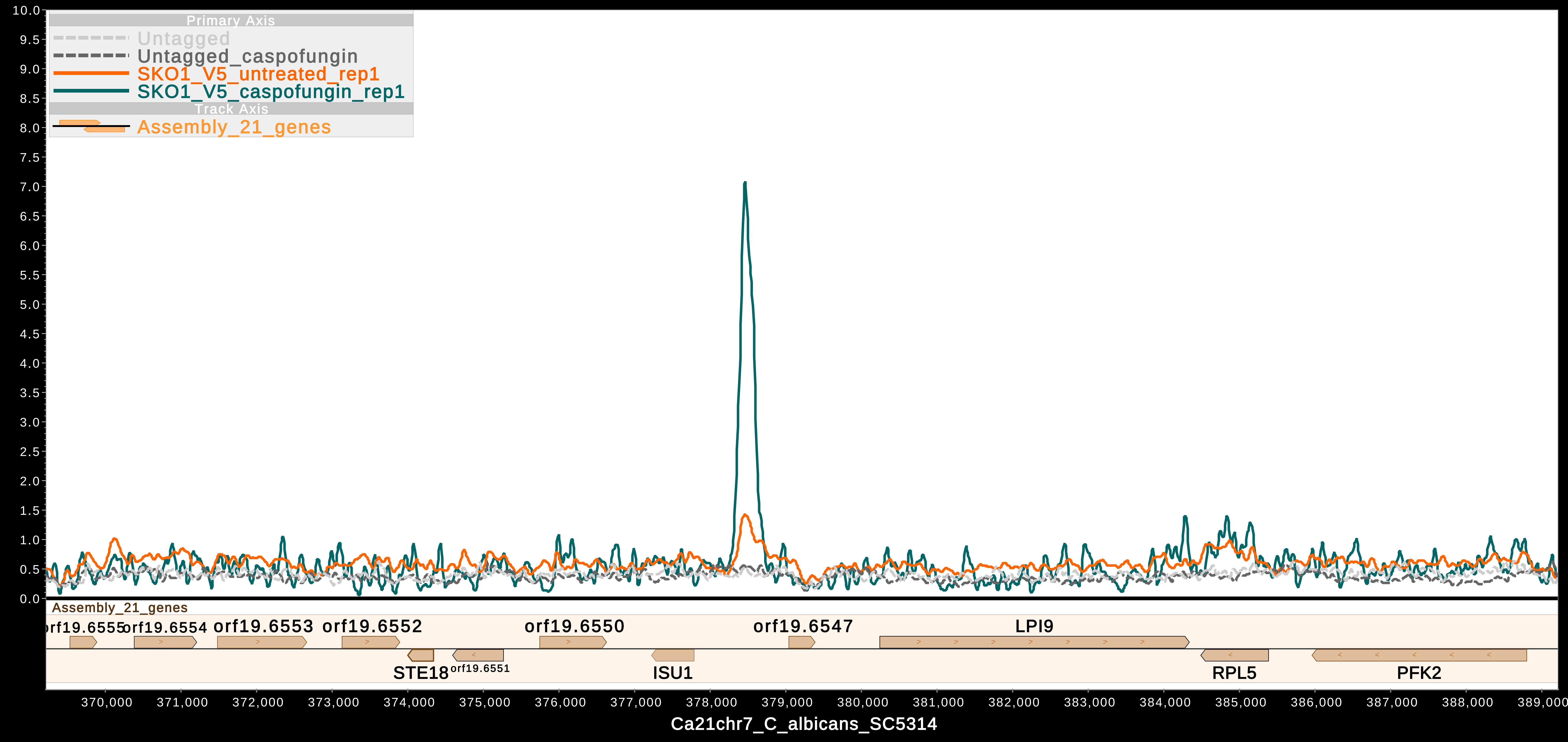

Primary Axis
Untagged
Untagged_caspofungin
SKO1_V5_untreated_rep1
SKO1_V5_caspofungin_rep1
Track Axis
Assembly_21_genes
10.0
9.5
9.0
8.5
8.0
7.5
7.0
6.5
6.0
5.5
5.0
4.5
4.0
3.5
3.0
2.5
2.0
1.5
1.0
0.5
0.0
Assembly_21_genes
orf19.6555
orf19.6554
orf19.6553
orf19.6552
orf19.6550
orf19.6547
LPI9
STE18
orf19.6551
ISU1
RPL5
PFK2
370,000
371,000
372,000
373,000
374,000
375,000
376,000
377,000
378,000
379,000
380,000
381,000
382,000
383,000
384,000
385,000
386,000
387,000
388,000
389,000
Ca21chr7_C_albicans_SC5314

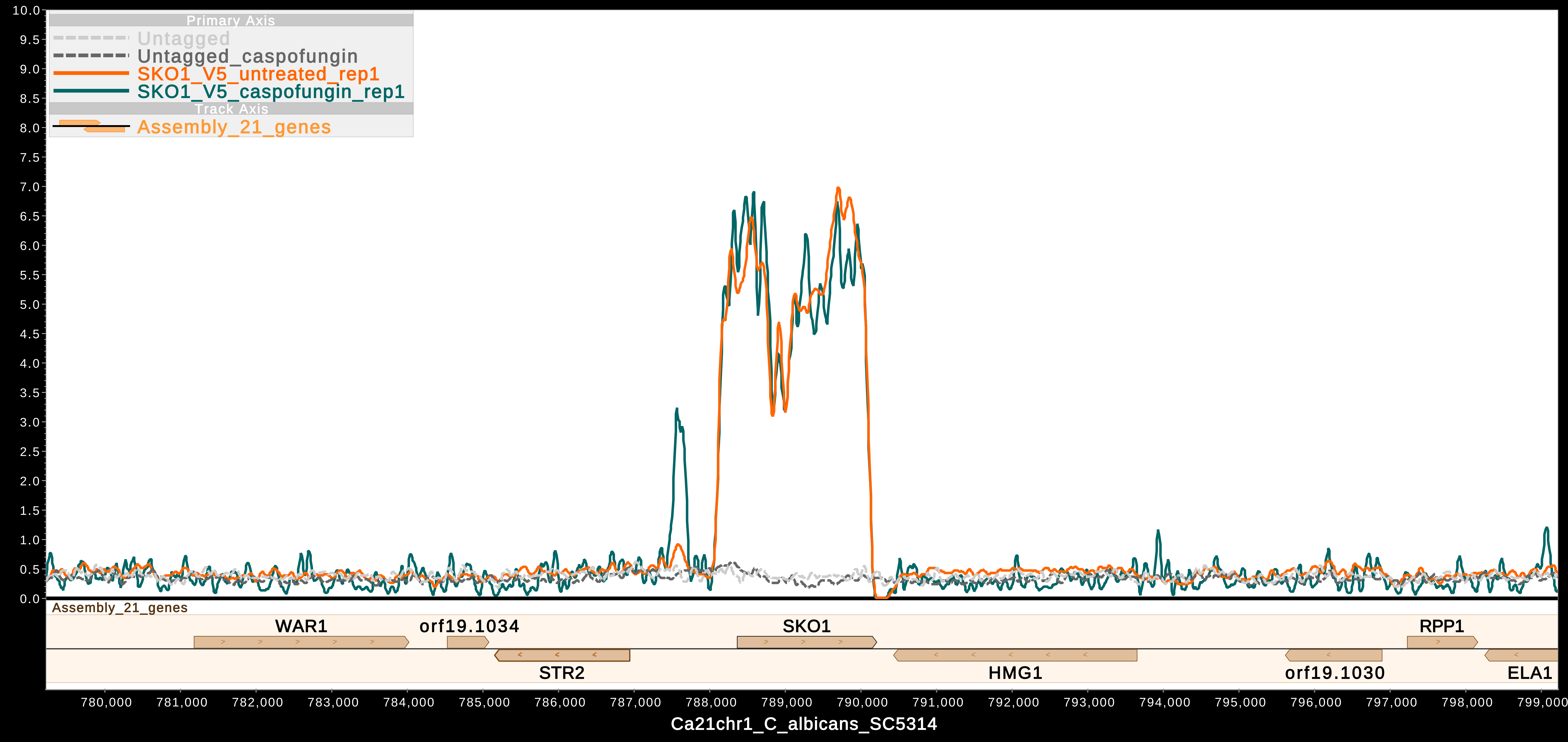

Primary Axis
Untagged
Untagged_caspofungin
SKO1_V5_untreated_rep1
SKO1_V5_caspofungin_rep1
Track Axis
Assembly_21_genes
10.0
9.5
9.0
8.5
8.0
7.5
7.0
6.5
6.0
5.5
5.0
4.5
4.0
3.5
3.0
2.5
2.0
1.5
1.0
0.5
0.0
Assembly_21_genes
WAR1
orf19.1034
SKO1
RPP1
STR2
HMG1
orf19.1030
ELA1
780,000
781,000
782,000
783,000
784,000
785,000
786,000
787,000
788,000
789,000
790,000
791,000
792,000
793,000
794,000
795,000
796,000
797,000
798,000
799,000
Ca21chr1_C_albicans_SC5314

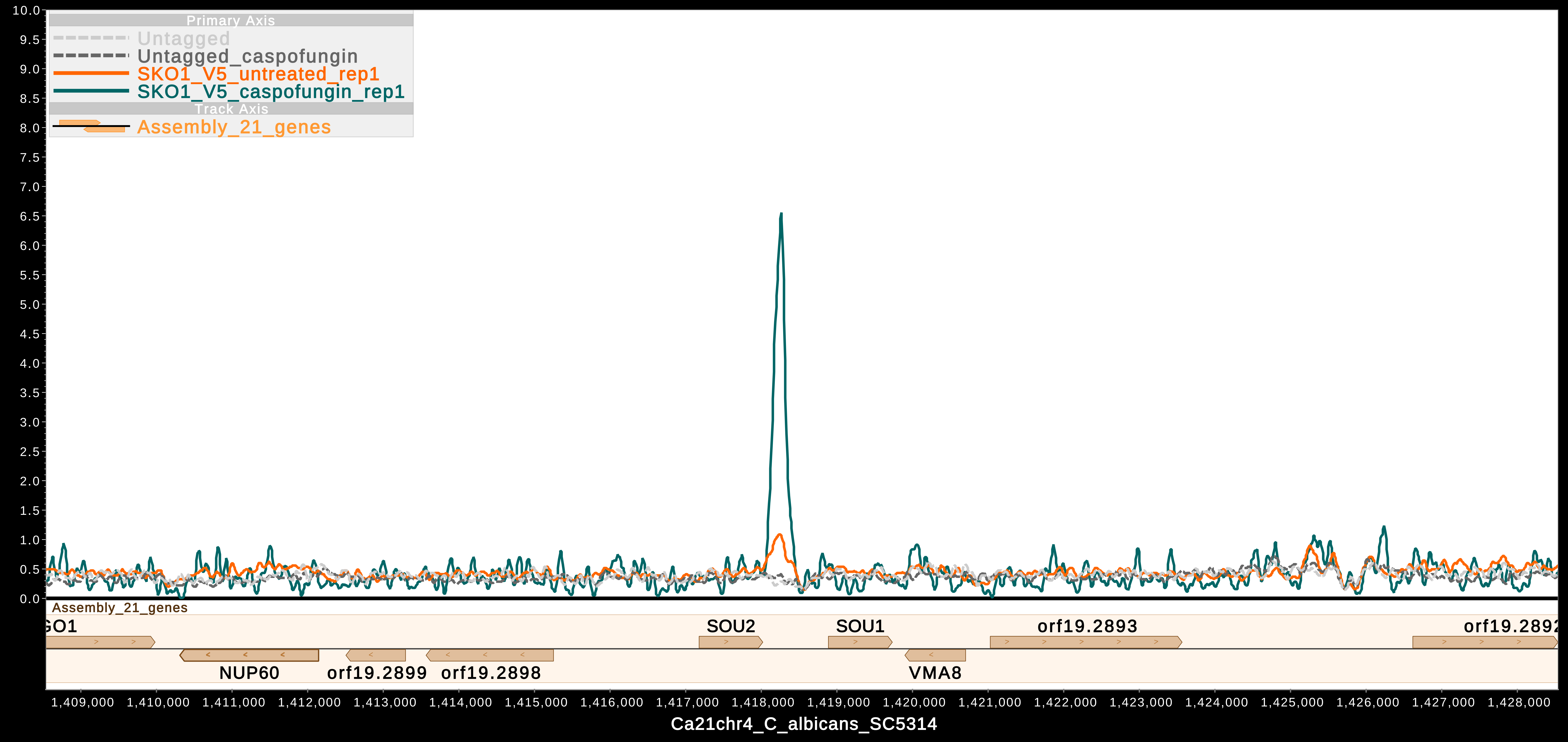

Primary Axis
Untagged
Untagged_caspofungin
SKO1_V5_untreated_rep1
SKO1_V5_caspofungin_rep1
Track Axis
Assembly_21_genes
10.0
9.5
9.0
8.5
8.0
7.5
7.0
6.5
6.0
5.5
5.0
4.5
4.0
3.5
3.0
2.5
2.0
1.5
1.0
0.5
0.0
Assembly_21_genes
SOU2
SOU1
orf19.2893
NUP60
orf19.2899
orf19.2898
VMA8
1,409,000
1,410,000
1,411,000
1,412,000
1,413,000
1,414,000
1,415,000
1,416,000
1,417,000
1,418,000
1,419,000
1,420,000
1,421,000
1,422,000
1,423,000
1,424,000
1,425,000
1,426,000
1,427,000
1,428,000
Ca21chr4_C_albicans_SC5314

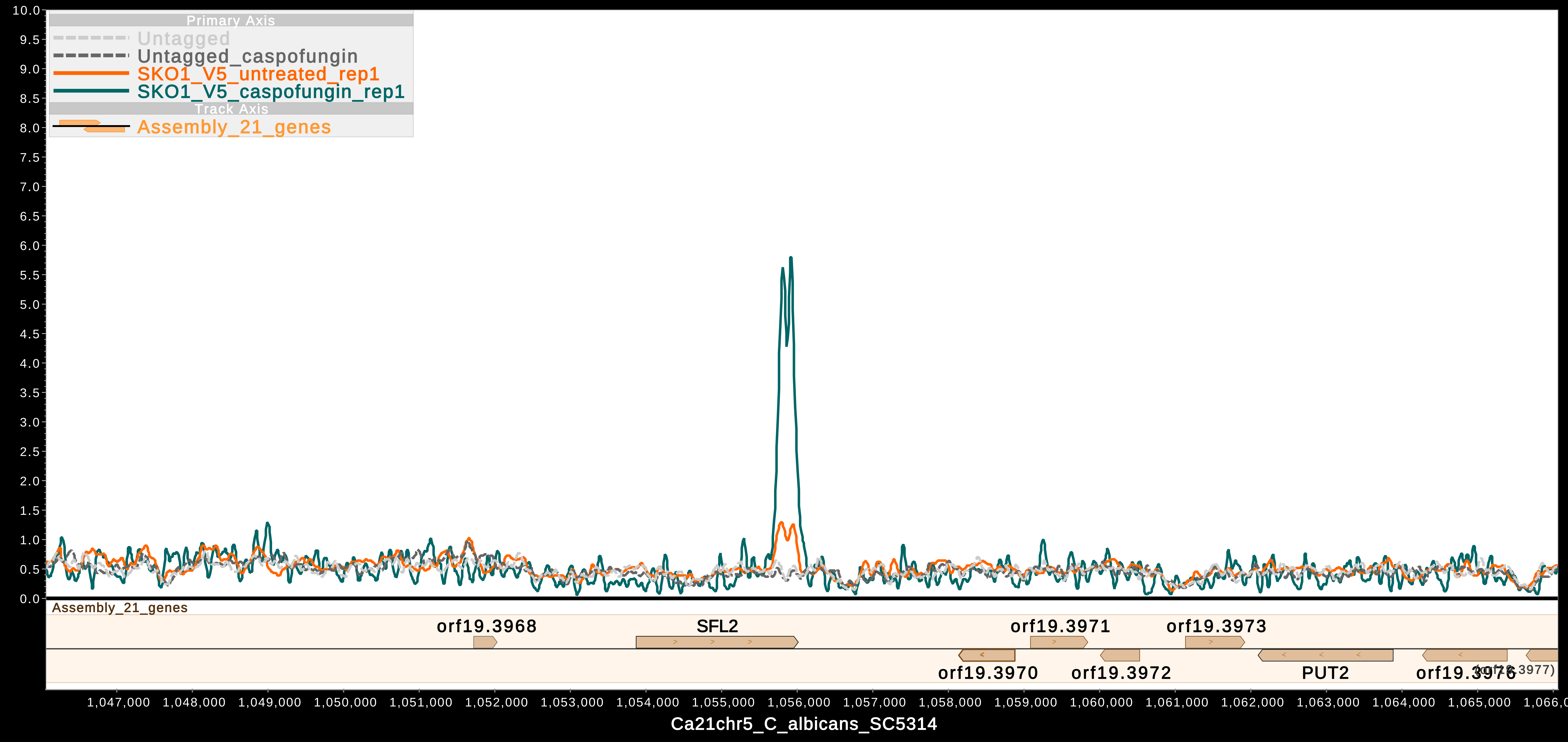

Primary Axis
Untagged
Untagged_caspofungin
SKO1_V5_untreated_rep1
SKO1_V5_caspofungin_rep1
Track Axis
Assembly_21_genes
10.0
9.5
9.0
8.5
8.0
7.5
7.0
6.5
6.0
5.5
5.0
4.5
4.0
3.5
3.0
2.5
2.0
1.5
1.0
0.5
0.0
Assembly_21_genes
orf19.3968
SFL2
orf19.3971
orf19.3973
orf19.3970
orf19.3972
PUT2
orf19.3976
1,047,000
1,048,000
1,049,000
1,050,000
1,051,000
1,052,000
1,053,000
1,054,000
1,055,000
1,056,000
1,057,000
1,058,000
1,059,000
1,060,000
1,061,000
1,062,000
1,063,000
1,064,000
1,065,000
Ca21chr5_C_albicans_SC5314

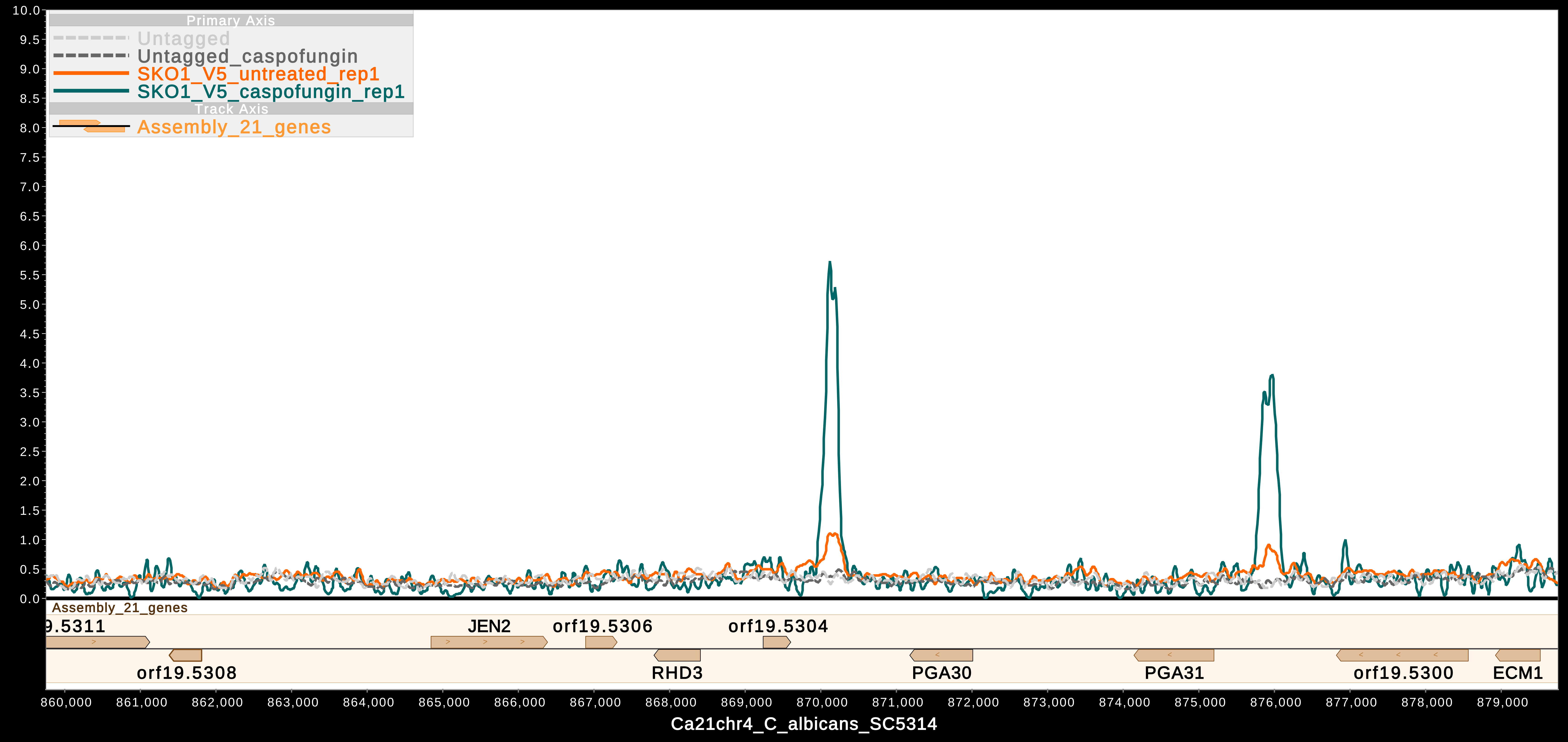

Primary Axis
Untagged
Untagged_caspofungin
SKO1_V5_untreated_rep1
SKO1_V5_caspofungin_rep1
Track Axis
Assembly_21_genes
10.0
9.5
9.0
8.5
8.0
7.5
7.0
6.5
6.0
5.5
5.0
4.5
4.0
3.5
3.0
2.5
2.0
1.5
1.0
0.5
0.0
Assembly_21_genes
9.5311
JEN2
orf19.5306
orf19.5304
orf19.5308
RHD3
PGA30
PGA31
orf19.5300
ECM1
860,000
861,000
862,000
863,000
864,000
865,000
866,000
867,000
868,000
869,000
870,000
871,000
872,000
873,000
874,000
875,000
876,000
877,000
878,000
879,000
Ca21chr4_C_albicans_SC5314

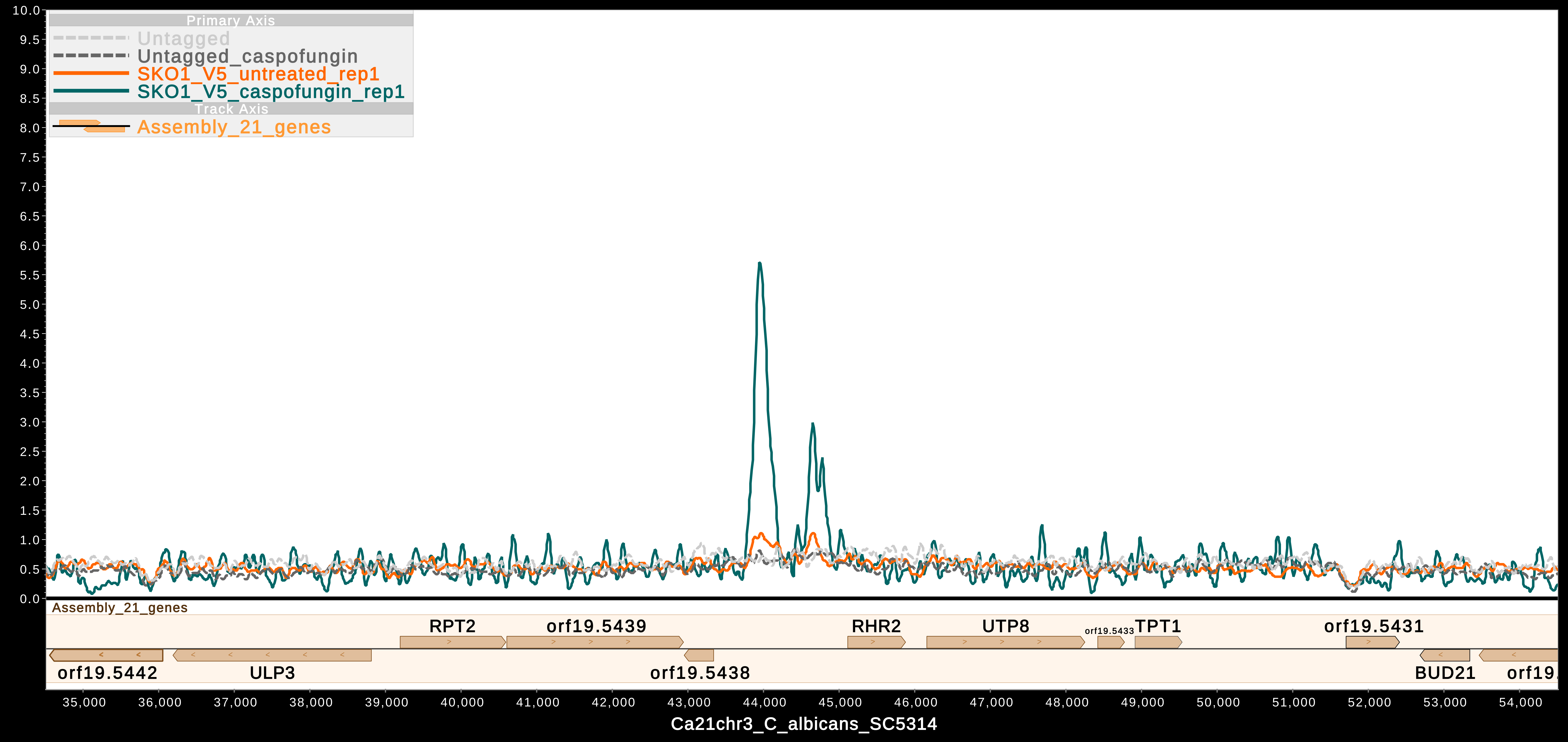
Primary Axis
Untagged
Untagged_caspofungin
SKO1_V5_untreated_rep1
SKO1_V5_caspofungin_rep1
Track Axis
Assembly_21_genes
10.0
9.5
9.0
8.5
8.0
7.5
7.0
6.5
6.0
5.5
5.0
4.5
4.0
3.5
3.0
2.5
2.0
1.5
1.0
0.5
0.0
Assembly_21_genes
RPT2
orf19.5439
RHR2
UTP8
orf19.5433
TPT1
orf19.5431
orf19.5442
ULP3
orf19.5438
BUD21
35,000
36,000
37,000
38,000
39,000
40,000
41,000
42,000
43,000
44,000
45,000
46,000
47,000
48,000
49,000
50,000
51,000
52,000
53,000
54,000
Ca21chr3_C_albicans_SC5314

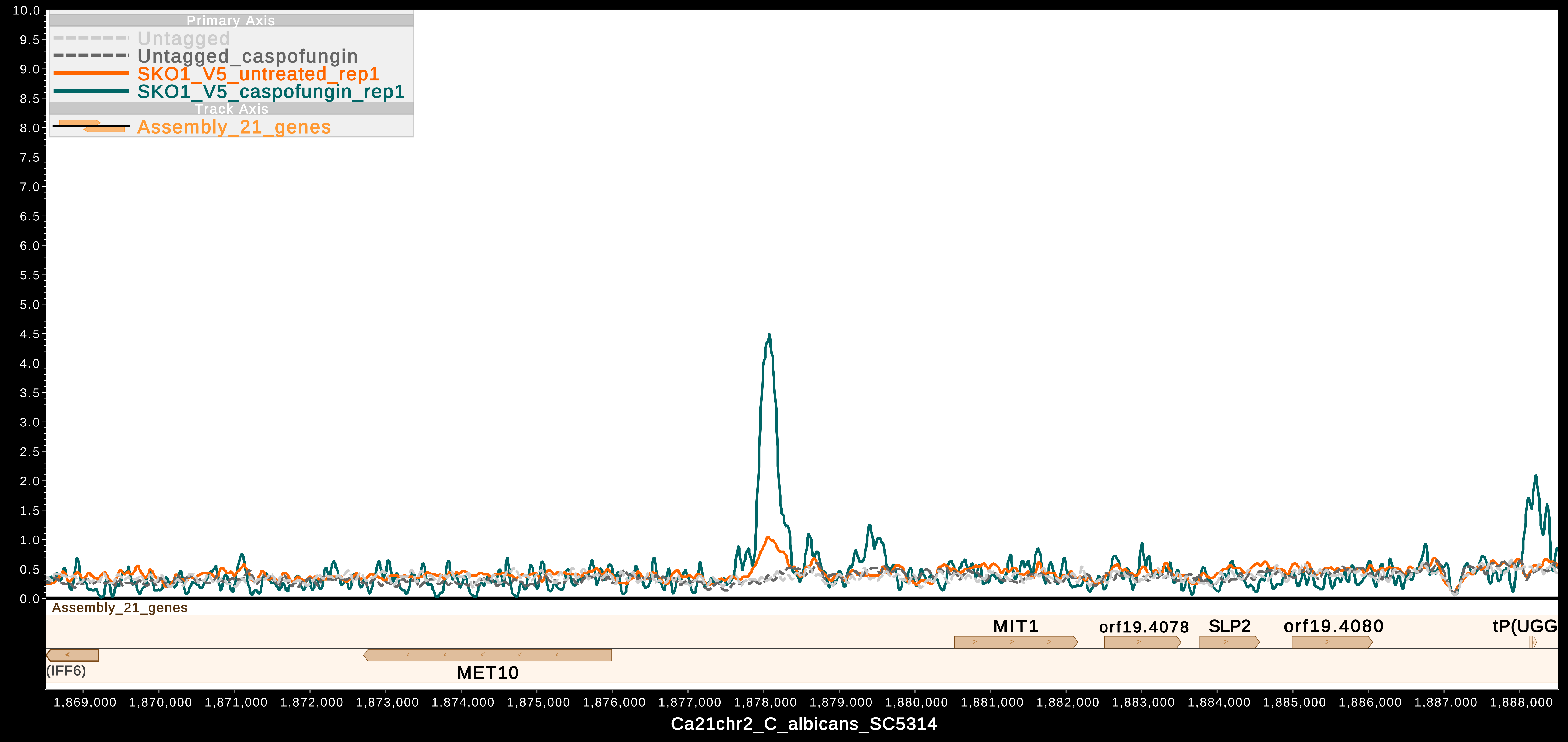

Primary Axis
Untagged
Untagged_caspofungin
SKO1_V5_untreated_rep1
SKO1_V5_caspofungin_rep1
Track Axis
Assembly_21_genes
10.0
9.5
9.0
8.5
8.0
7.5
7.0
6.5
6.0
5.5
5.0
4.5
4.0
3.5
3.0
2.5
2.0
1.5
1.0
0.5
0.0
Assembly_21_genes
MIT1
orf19.4078
SLP2
orf19.4080
tP(UGG
(IFF6)
MET10
1,869,000
1,870,000
1,871,000
1,872,000
1,873,000
1,874,000
1,875,000
1,876,000
1,877,000
1,878,000
1,879,000
1,880,000
1,881,000
1,882,000
1,883,000
1,884,000
1,885,000
1,886,000
1,887,000
1,888,000
Ca21chr2_C_albicans_SC5314

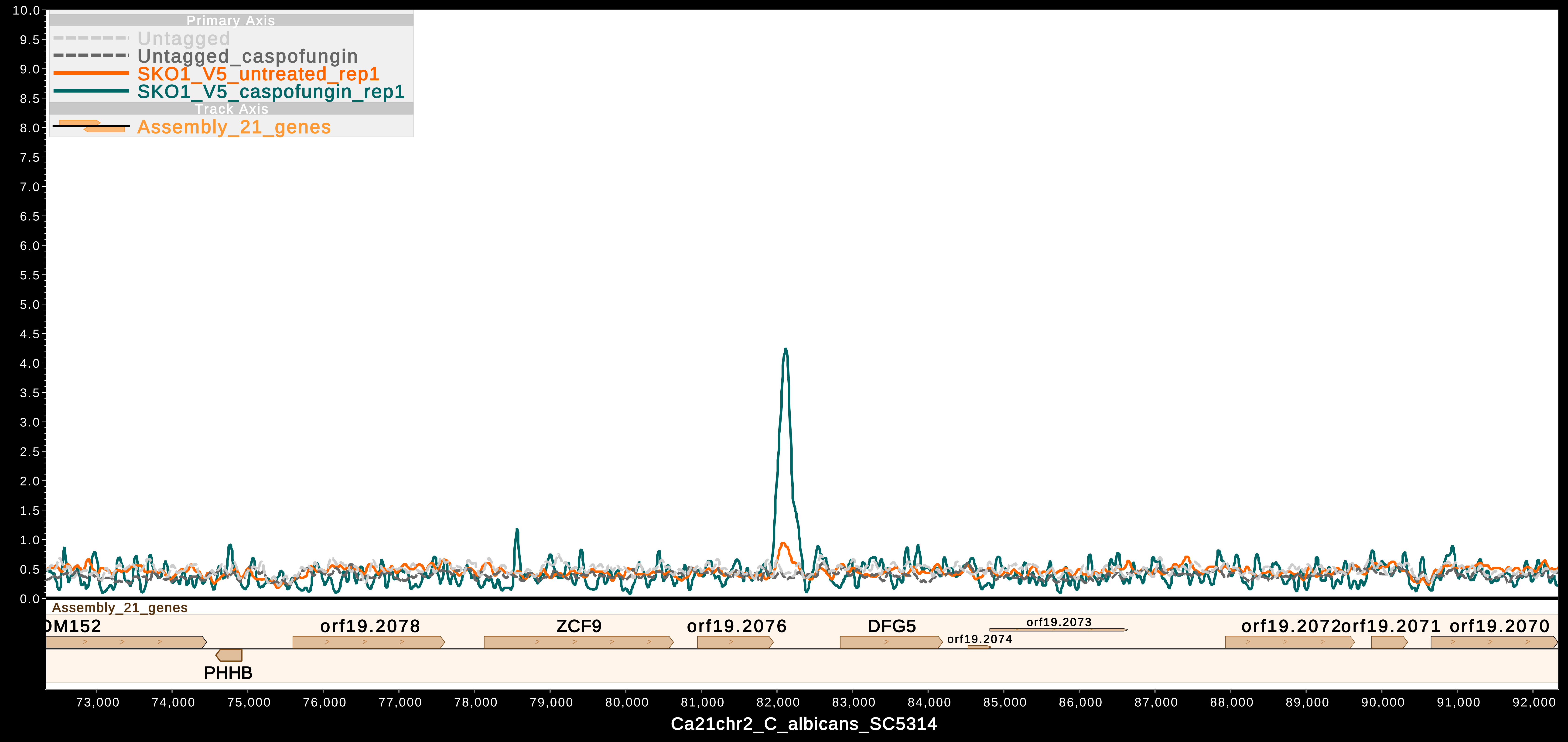

Primary Axis
Untagged
Untagged_caspofungin
SKO1_V5_untreated_rep1
SKO1_V5_caspofungin_rep1
Track Axis
Assembly_21_genes
Assembly_21_genes
DM152
orf19.2078
ZCF9
orf19.2076
DFG5
orf19.2073
orf19.2074
orf19.2072
orf19.2071
orf19.2070
PHHB
Ca21chr2_C_albicans_SC5314

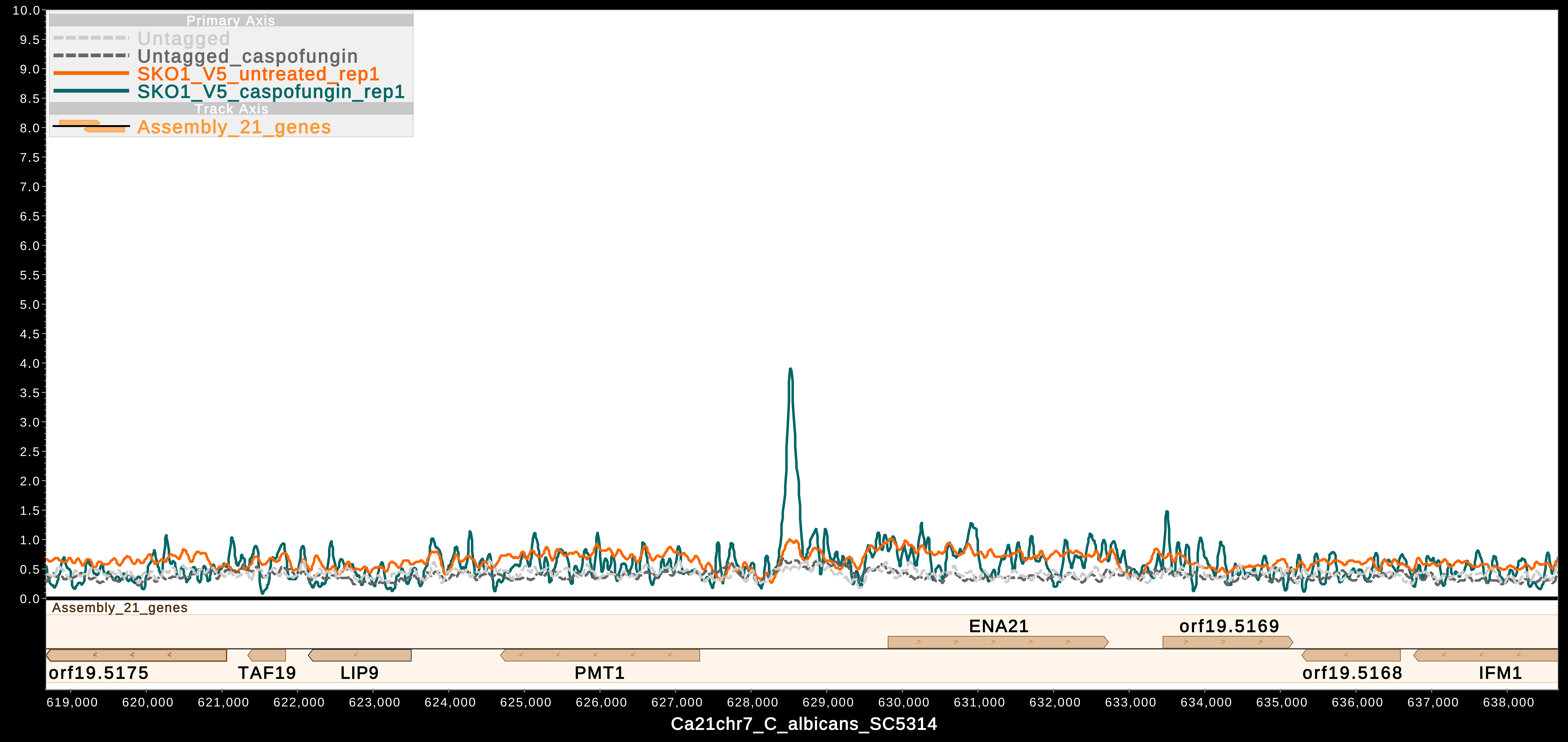

Primary Axis
Untagged
Untagged_caspofungin
SKO1_V5_untreated_rep1
SKO1_V5_caspofungin_rep1
Track Axis
Assembly_21_genes
0.0
0.5
1.0
1.5
2.0
2.5
3.0
3.5
4.0
4.5
5.0
5.5
6.0
6.5
7.0
7.5
8.0
8.5
9.0
9.5
10.0
Assembly_21_genes
ENA21
orf19.5169
orf19.5175
TAF19
LIP9
PMT1
orf19.5168
IFM1
619,000
620,000
621,000
622,000
623,000
624,000
625,000
626,000
627,000
628,000
629,000
630,000
631,000
632,000
633,000
634,000
635,000
636,000
637,000
638,000
Ca21chr7_C_albicans_SC5314

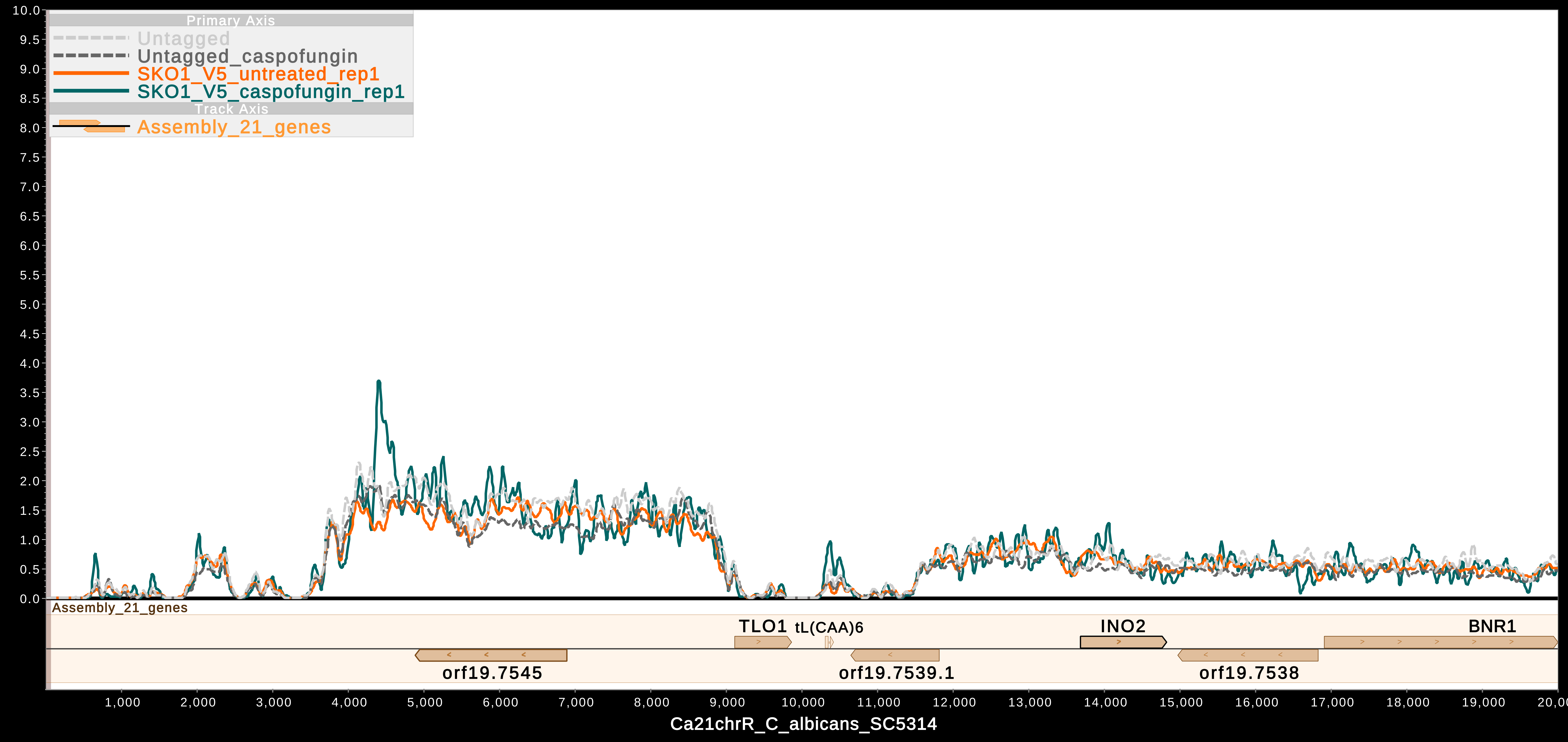

Primary Axis
Untagged
Untagged_caspofungin
SKO1_V5_untreated_rep1
SKO1_V5_caspofungin_rep1
Track Axis
Assembly_21_genes
10.0
9.5
9.0
8.5
8.0
7.5
7.0
6.5
6.0
5.5
5.0
4.5
4.0
3.5
3.0
2.5
2.0
1.5
1.0
0.5
0.0
Assembly_21_genes
TLO1
tL(CAA)6
INO2
BNR1
orf19.7545
orf19.7539.1
orf19.7538
1,000
2,000
3,000
4,000
5,000
6,000
7,000
8,000
9,000
10,000
11,000
12,000
13,000
14,000
15,000
16,000
17,000
18,000
19,000
Ca21chrR_C_albicans_SC5314

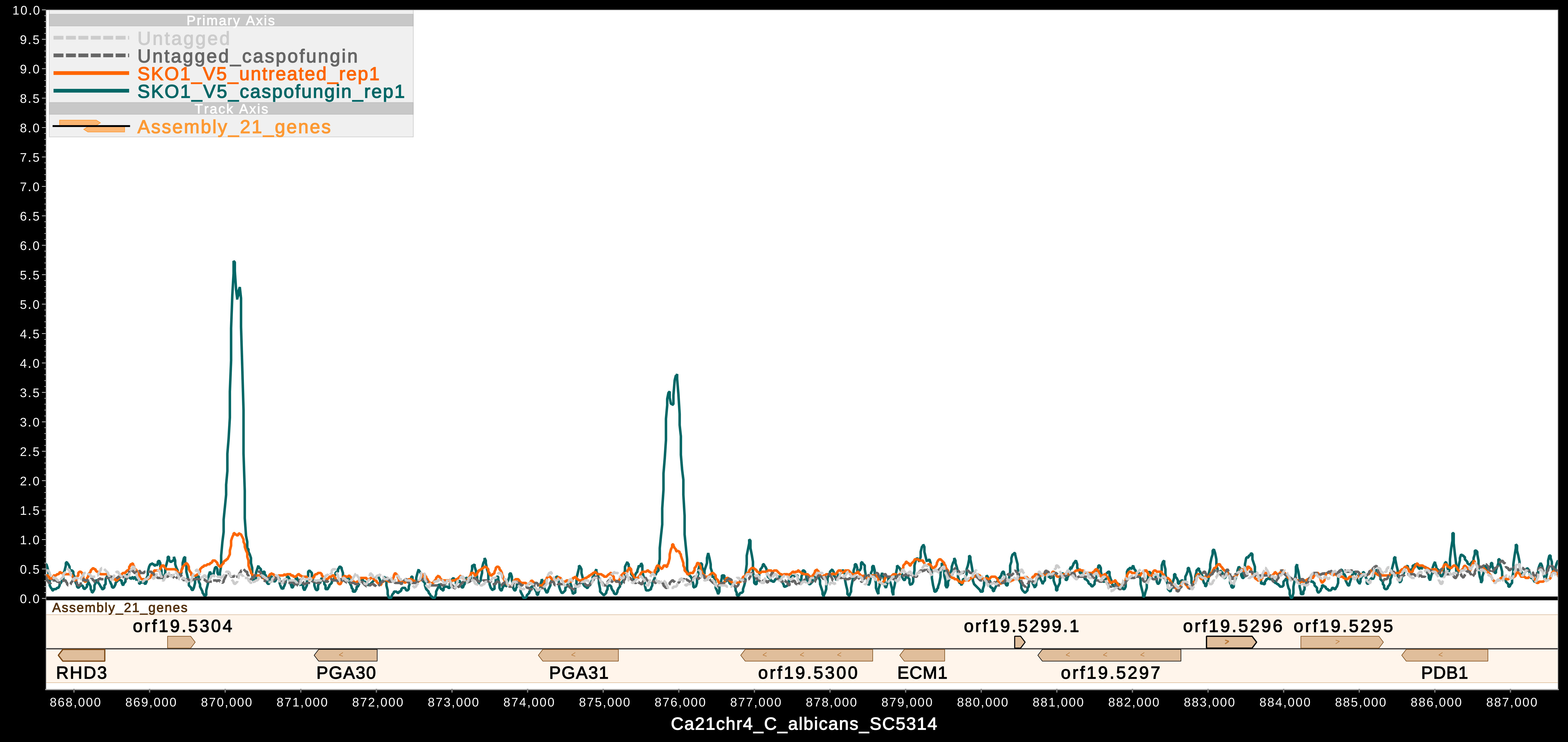

Primary Axis
Untagged
Untagged_caspofungin
SKO1_V5_untreated_rep1
SKO1_V5_caspofungin_rep1
Track Axis
Assembly_21_genes
10.0
9.5
9.0
8.5
8.0
7.5
7.0
6.5
6.0
5.5
5.0
4.5
4.0
3.5
3.0
2.5
2.0
1.5
1.0
0.5
0.0
Assembly_21_genes
orf19.5304
orf19.5299.1
orf19.5296
orf19.5295
RHD3
PGA30
PGA31
orf19.5300
ECM1
orf19.5297
PDB1
868,000
869,000
870,000
871,000
872,000
873,000
874,000
875,000
876,000
877,000
878,000
879,000
880,000
881,000
882,000
883,000
884,000
885,000
886,000
887,000
Ca21chr4_C_albicans_SC5314

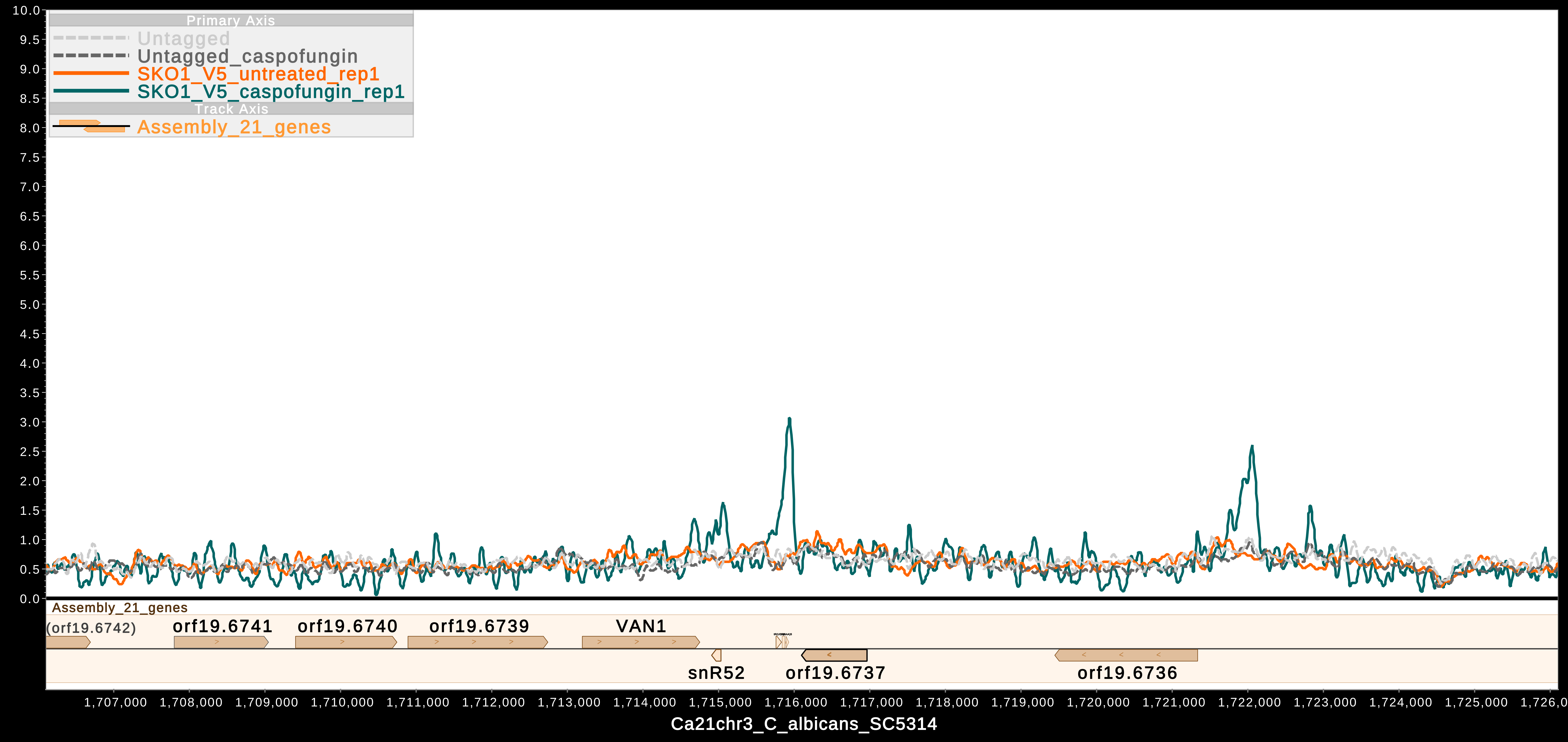
Primary Axis
Untagged
Untagged_caspofungin
SKO1_V5_untreated_rep1
SKO1_V5_caspofungin_rep1
Track Axis
Assembly_21_genes
10.0
9.5
9.0
8.5
8.0
7.5
7.0
6.5
6.0
5.5
5.0
4.5
4.0
3.5
3.0
2.5
2.0
1.5
1.0
0.5
0.0
Assembly_21_genes
(orf19.6742)
orf19.6741
orf19.6740
orf19.6739
VAN1
snR52
orf19.6737
orf19.6736
1,707,000
1,708,000
1,709,000
1,710,000
1,711,000
1,712,000
1,713,000
1,714,000
1,715,000
1,716,000
1,717,000
1,718,000
1,719,000
1,720,000
1,721,000
1,722,000
1,723,000
1,724,000
1,725,000
Ca21chr3_C_albicans_SC5314

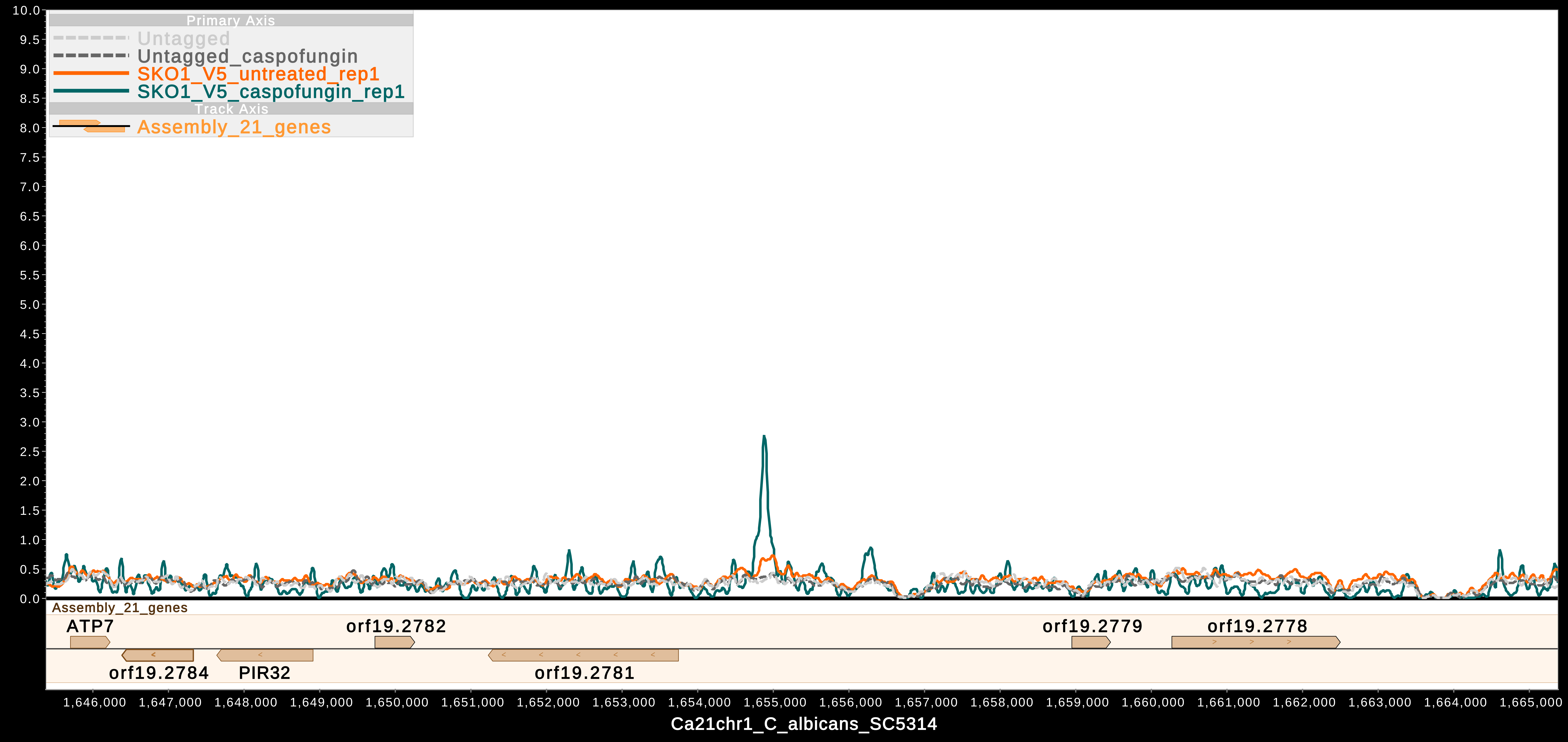

Primary Axis
Untagged
Untagged_caspofungin
SKO1_V5_untreated_rep1
SKO1_V5_caspofungin_rep1
Track Axis
Assembly_21_genes
10.0
9.5
9.0
8.5
8.0
7.5
7.0
6.5
6.0
5.5
5.0
4.5
4.0
3.5
3.0
2.5
2.0
1.5
1.0
0.5
0.0
Assembly_21_genes
ATP7
orf19.2782
orf19.2779
orf19.2778
orf19.2784
PIR32
orf19.2781
1,646,000
1,647,000
1,648,000
1,649,000
1,650,000
1,651,000
1,652,000
1,653,000
1,654,000
1,655,000
1,656,000
1,657,000
1,658,000
1,659,000
1,660,000
1,661,000
1,662,000
1,663,000
1,664,000
1,665,000
Ca21chr1_C_albicans_SC5314

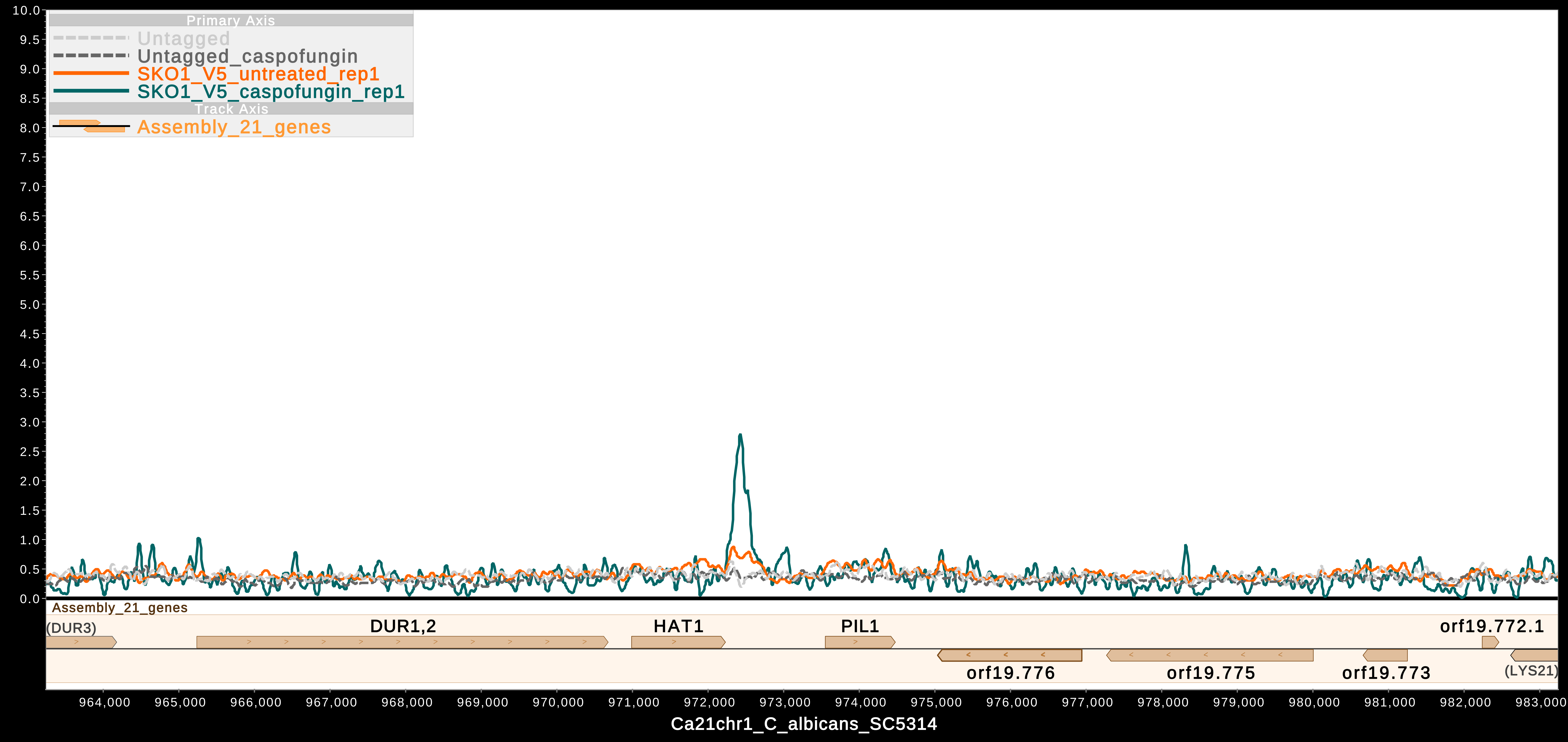

Primary Axis
Untagged
Untagged_caspofungin
SKO1_V5_untreated_rep1
SKO1_V5_caspofungin_rep1
Track Axis
Assembly_21_genes
10.0
9.5
9.0
8.5
8.0
7.5
7.0
6.5
6.0
5.5
5.0
4.5
4.0
3.5
3.0
2.5
2.0
1.5
1.0
0.5
0.0
Assembly_21_genes
(DUR3)
DUR1,2
HAT1
PIL1
orf19.772.1
orf19.776
orf19.775
orf19.773
(LYS21)
964,000
965,000
966,000
967,000
968,000
969,000
970,000
971,000
972,000
973,000
974,000
975,000
976,000
977,000
978,000
979,000
980,000
981,000
982,000
983,000
Ca21chr1_C_albicans_SC5314

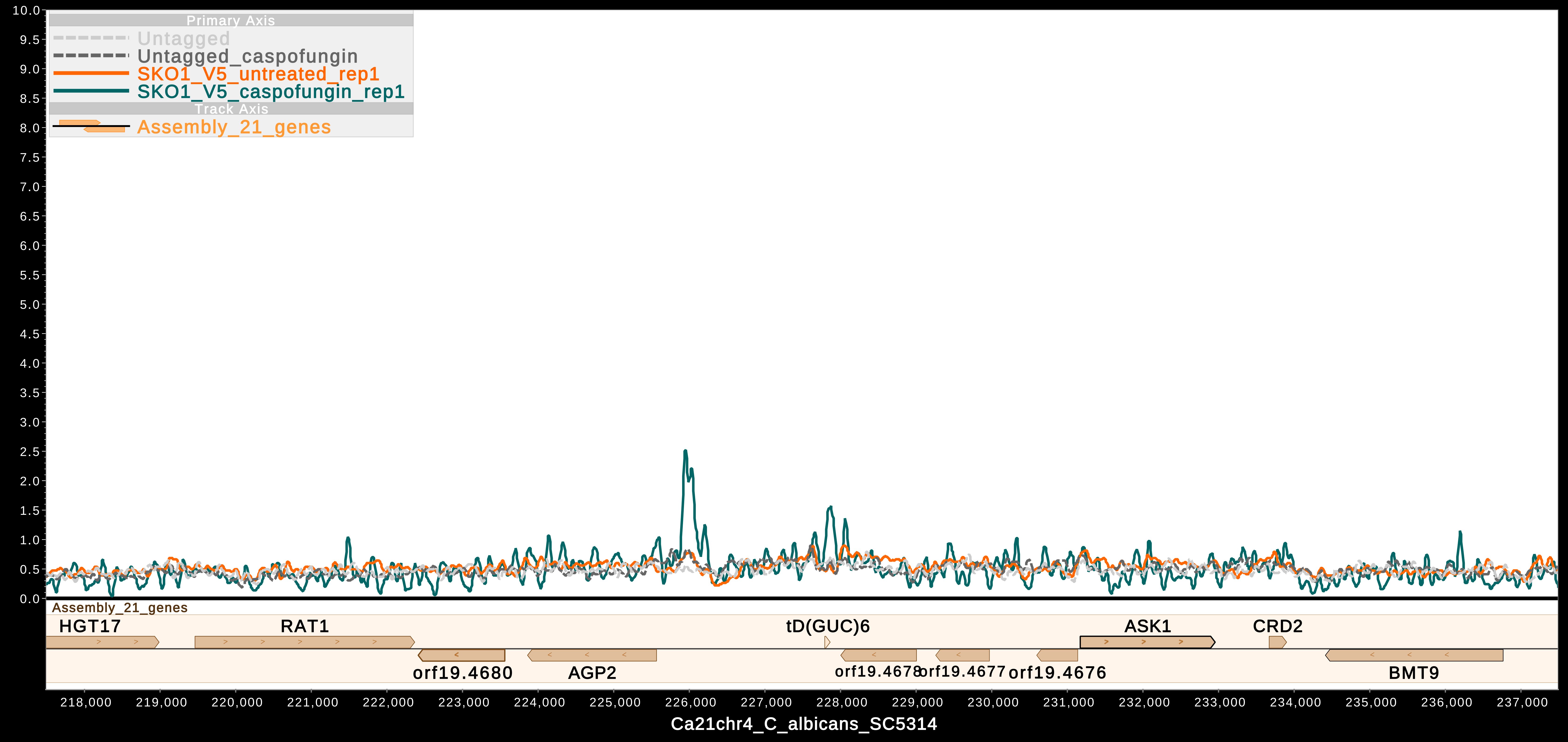

Primary Axis
Untagged
Untagged_caspofungin
SKO1_V5_untreated_rep1
SKO1_V5_caspofungin_rep1
Track Axis
Assembly_21_genes
10.0
9.5
9.0
8.5
8.0
7.5
7.0
6.5
6.0
5.5
5.0
4.5
4.0
3.5
3.0
2.5
2.0
1.5
1.0
0.5
0.0
Assembly_21_genes
HGT17
RAT1
tD(GUC)6
ASK1
CRD2
orf19.4680
AGP2
orf19.4678
orf19.4677
orf19.4676
BMT9
218,000
219,000
220,000
221,000
222,000
223,000
224,000
225,000
226,000
227,000
228,000
229,000
230,000
231,000
232,000
233,000
234,000
235,000
236,000
237,000
Ca21chr4_C_albicans_SC5314

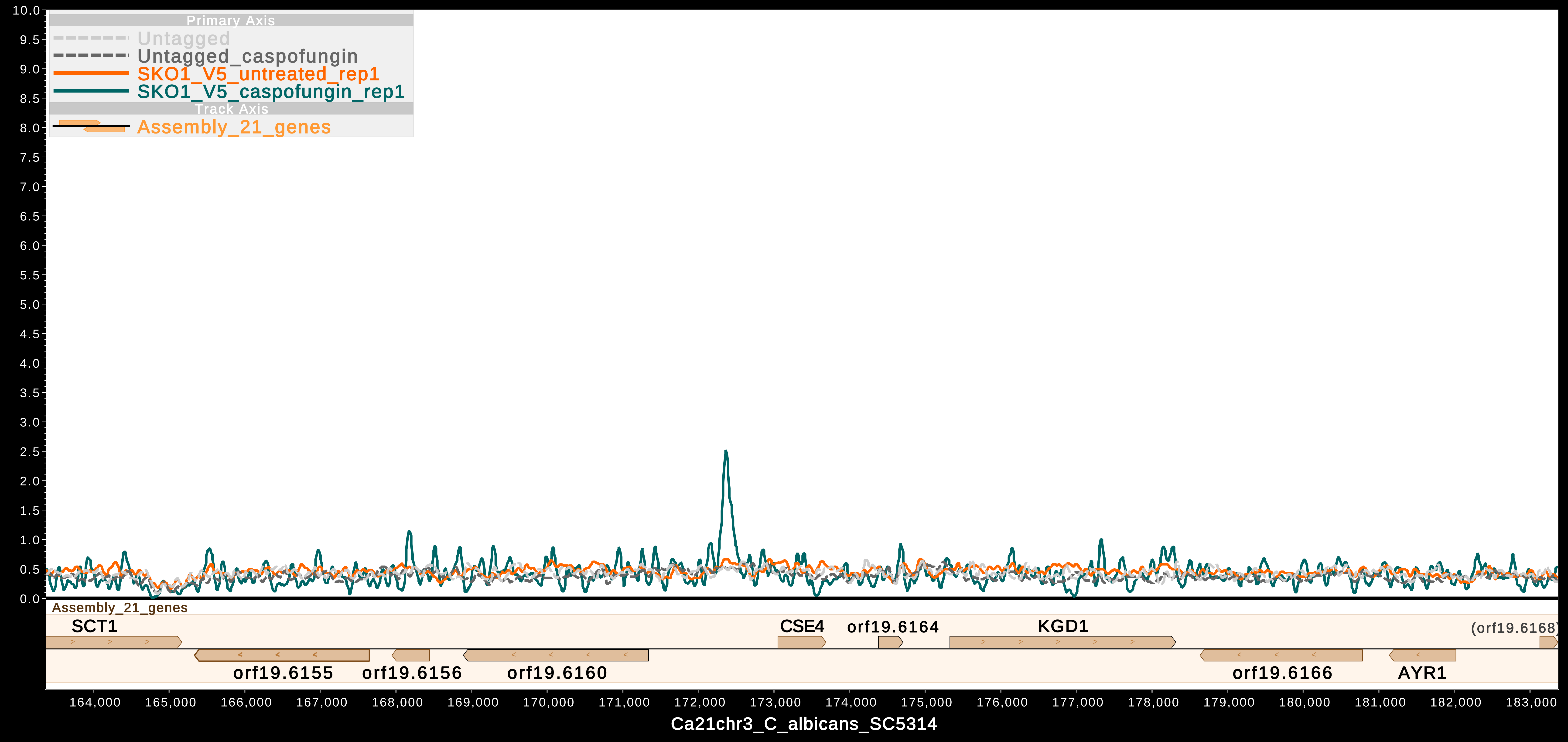
Primary Axis
Untagged
Untagged_caspofungin
SKO1_V5_untreated_rep1
SKO1_V5_caspofungin_rep1
Track Axis
Assembly_21_genes
10.0
9.5
9.0
8.5
8.0
7.5
7.0
6.5
6.0
5.5
5.0
4.5
4.0
3.5
3.0
2.5
2.0
1.5
1.0
0.5
0.0
Assembly_21_genes
SCT1
CSE4
orf19.6164
KGD1
(orf19.6168)
orf19.6155
orf19.6156
orf19.6160
orf19.6166
AYR1
164,000
165,000
166,000
167,000
168,000
169,000
170,000
171,000
172,000
173,000
174,000
175,000
176,000
177,000
178,000
179,000
180,000
181,000
182,000
183,000
Ca21chr3_C_albicans_SC5314

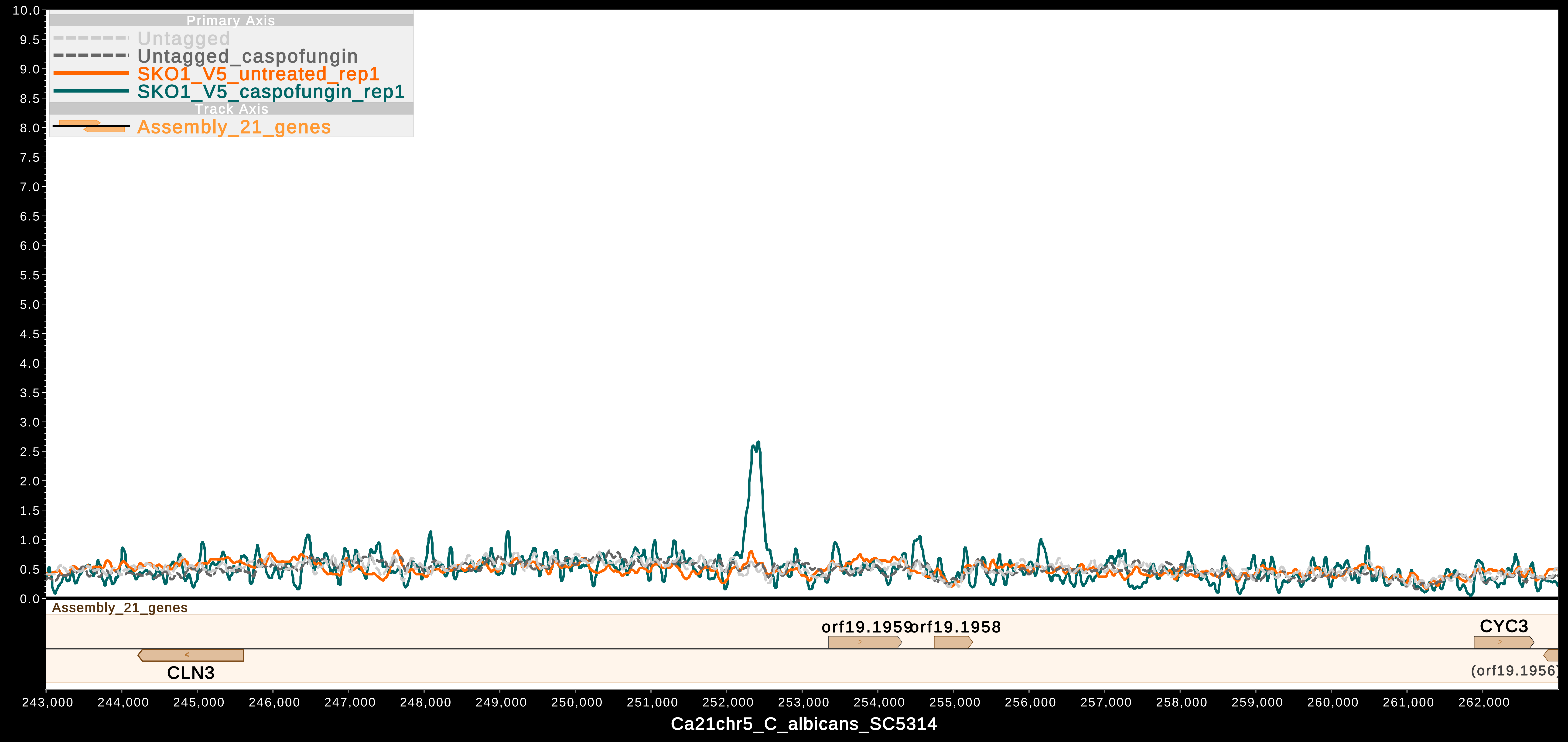
Primary Axis
Untagged
Untagged_caspofungin
SKO1_V5_untreated_rep1
SKO1_V5_caspofungin_rep1
Track Axis
Assembly_21_genes
10.0
9.5
9.0
8.5
8.0
7.5
7.0
6.5
6.0
5.5
5.0
4.5
4.0
3.5
3.0
2.5
2.0
1.5
1.0
0.5
0.0
Assembly_21_genes
orf19.1959
orf19.1958
CYC3
CLN3
(orf19.1956)
243,000
244,000
245,000
246,000
247,000
248,000
249,000
250,000
251,000
252,000
253,000
254,000
255,000
256,000
257,000
258,000
259,000
260,000
261,000
262,000
Ca21chr5_C_albicans_SC5314

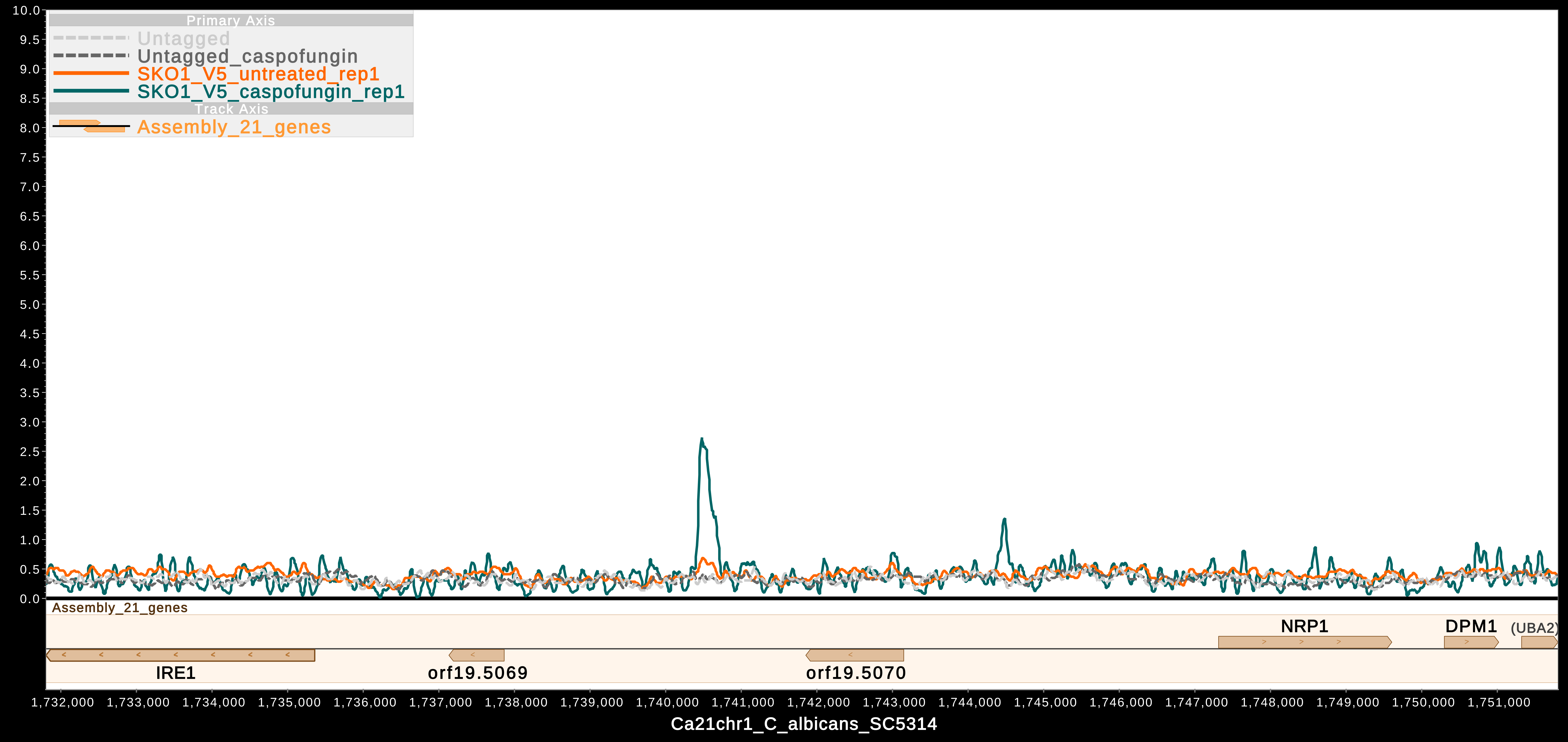

Primary Axis
Untagged
Untagged_caspofungin
SKO1_V5_untreated_rep1
SKO1_V5_caspofungin_rep1
Track Axis
Assembly_21_genes
10.0
9.5
9.0
8.5
8.0
7.5
7.0
6.5
6.0
5.5
5.0
4.5
4.0
3.5
3.0
2.5
2.0
1.5
1.0
0.5
0.0
Assembly_21_genes
NRP1
DPM1
(UBA2)
IRE1
orf19.5069
orf19.5070
1,732,000
1,733,000
1,734,000
1,735,000
1,736,000
1,737,000
1,738,000
1,739,000
1,740,000
1,741,000
1,742,000
1,743,000
1,744,000
1,745,000
1,746,000
1,747,000
1,748,000
1,749,000
1,750,000
1,751,000
Ca21chr1_C_albicans_SC5314

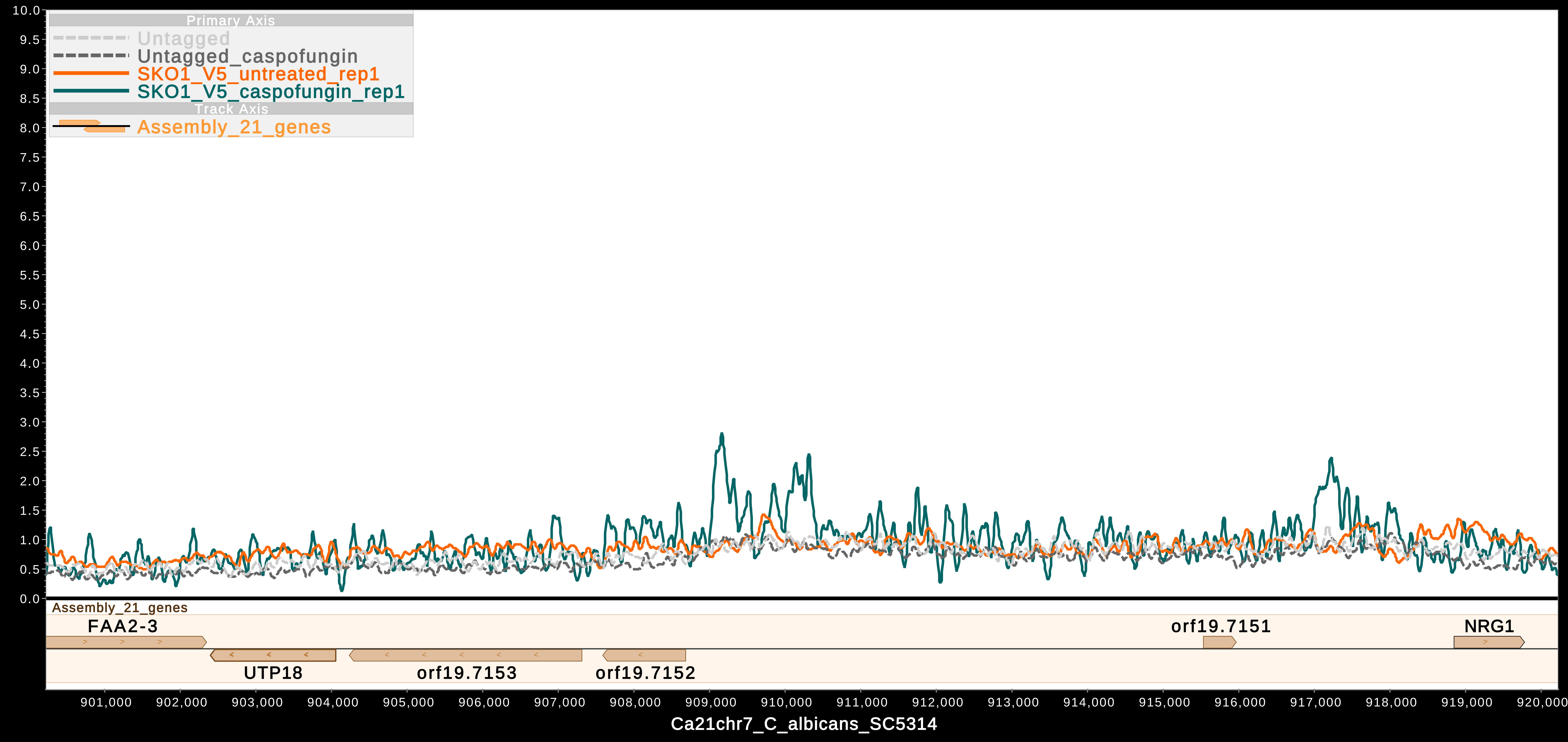

Primary Axis
Untagged
Untagged_caspofungin
SKO1_V5_untreated_rep1
SKO1_V5_caspofungin_rep1
Track Axis
Assembly_21_genes
10.0
9.5
9.0
8.5
8.0
7.5
7.0
6.5
6.0
5.5
5.0
4.5
4.0
3.5
3.0
2.5
2.0
1.5
1.0
0.5
0.0
Assembly_21_genes
FAA2-3
UTP18
orf19.7153
orf19.7152
orf19.7151
NRG1
901,000
902,000
903,000
904,000
905,000
906,000
907,000
908,000
909,000
910,000
911,000
912,000
913,000
914,000
915,000
916,000
917,000
918,000
919,000
920,000
Ca21chr7_C_albicans_SC5314

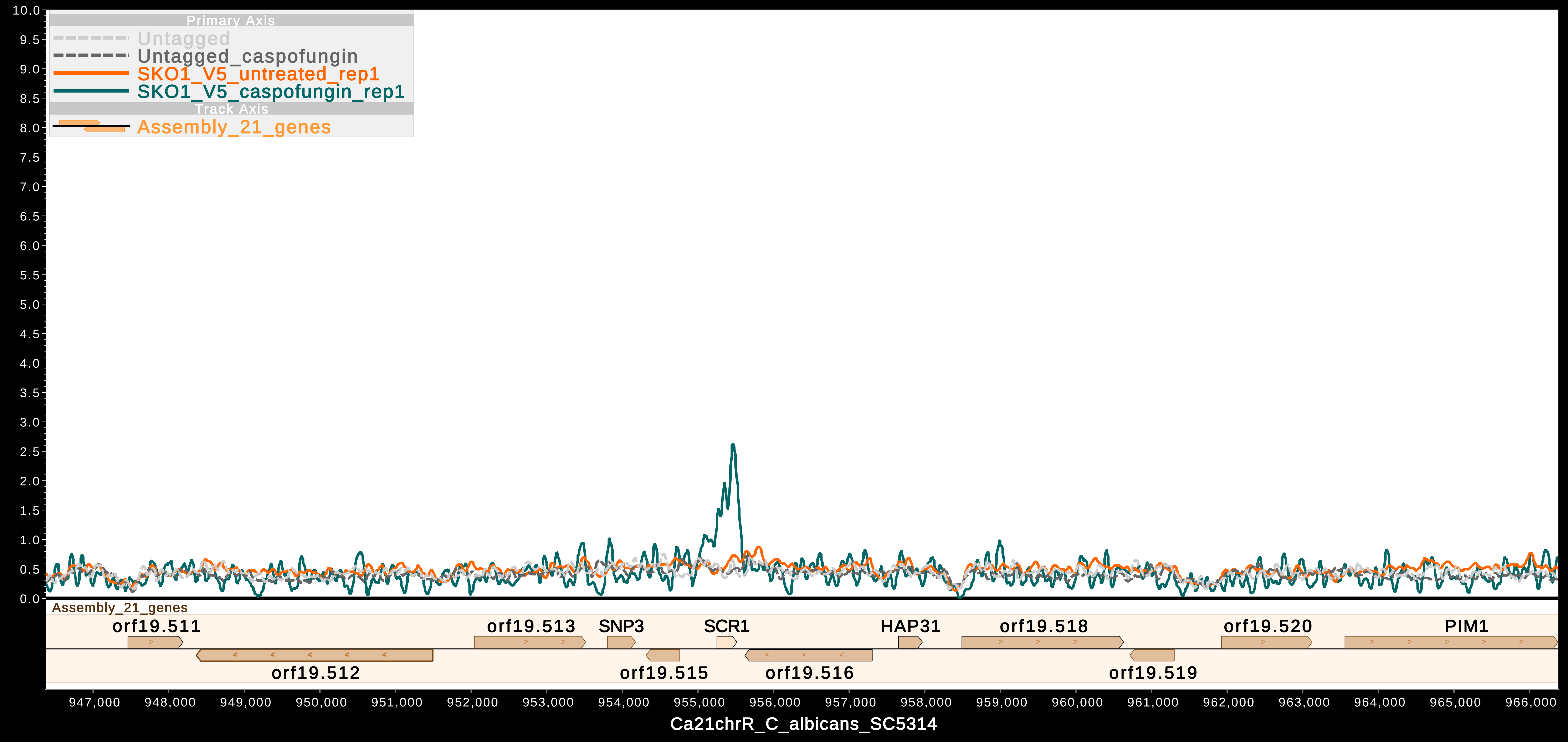

Primary Axis
Untagged
Untagged_caspofungin
SKO1_V5_untreated_rep1
SKO1_V5_caspofungin_rep1
Track Axis
Assembly_21_genes
10.0
9.5
9.0
8.5
8.0
7.5
7.0
6.5
6.0
5.5
5.0
4.5
4.0
3.5
3.0
2.5
2.0
1.5
1.0
0.5
0.0
Assembly_21_genes
orf19.511
orf19.513
SNP3
SCR1
HAP31
orf19.518
orf19.520
PIM1
orf19.512
orf19.515
orf19.516
orf19.519
947,000
948,000
949,000
950,000
951,000
952,000
953,000
954,000
955,000
956,000
957,000
958,000
959,000
960,000
961,000
962,000
963,000
964,000
965,000
966,000
Ca21chrR_C_albicans_SC5314

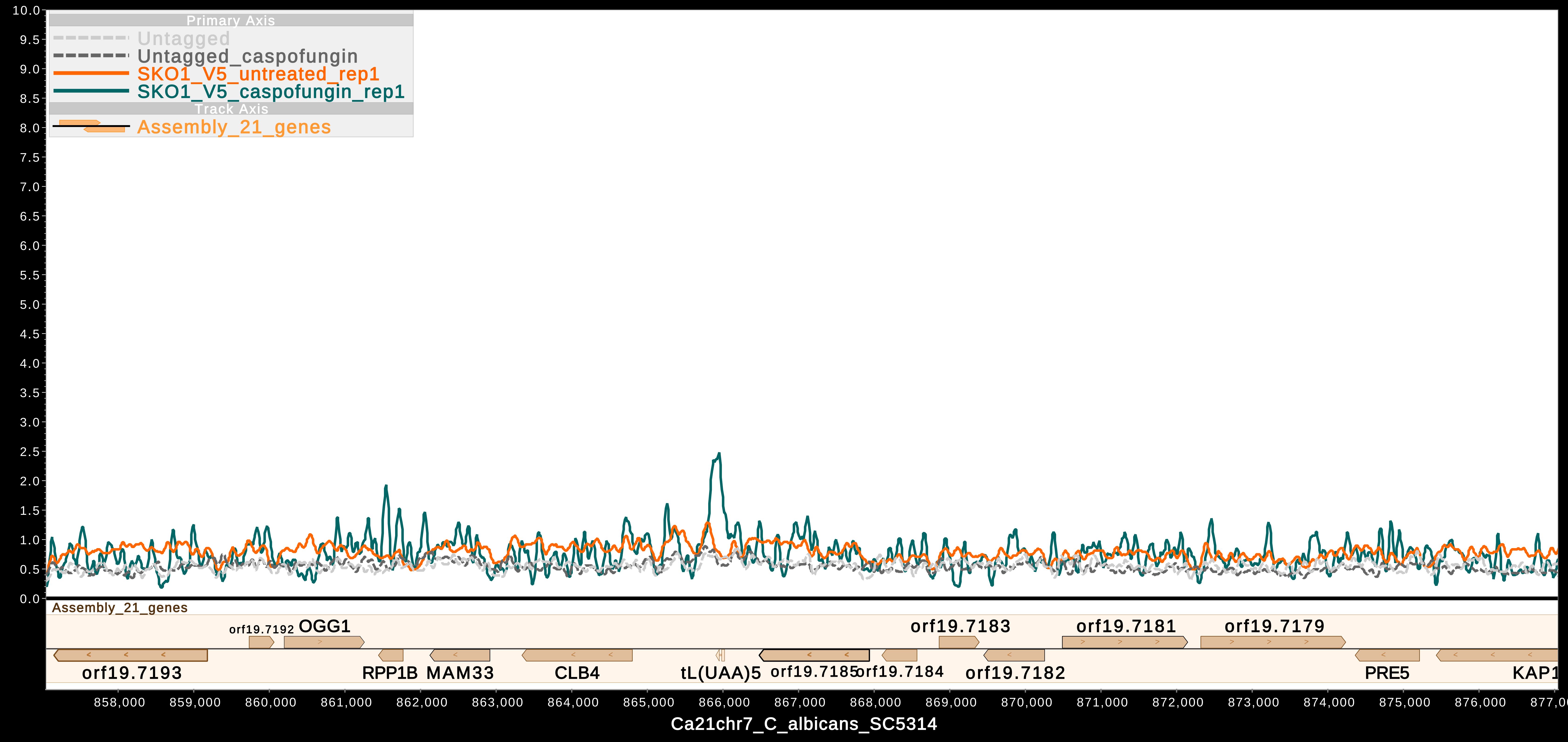

Primary Axis
Untagged
Untagged_caspofungin
SKO1_V5_untreated_rep1
SKO1_V5_caspofungin_rep1
Track Axis
Assembly_21_genes
10.0
9.5
9.0
8.5
8.0
7.5
7.0
6.5
6.0
5.5
5.0
4.5
4.0
3.5
3.0
2.5
2.0
1.5
1.0
0.5
0.0
Assembly_21_genes
orf19.7192
OGG1
orf19.7183
orf19.7181
orf19.7179
orf19.7193
RPP1B
MAM33
CLB4
tL(UAA)5
orf19.7185
orf19.7184
orf19.7182
PRE5
KAP1
858,000
859,000
860,000
861,000
862,000
863,000
864,000
865,000
866,000
867,000
868,000
869,000
870,000
871,000
872,000
873,000
874,000
875,000
876,000
Ca21chr7_C_albicans_SC5314

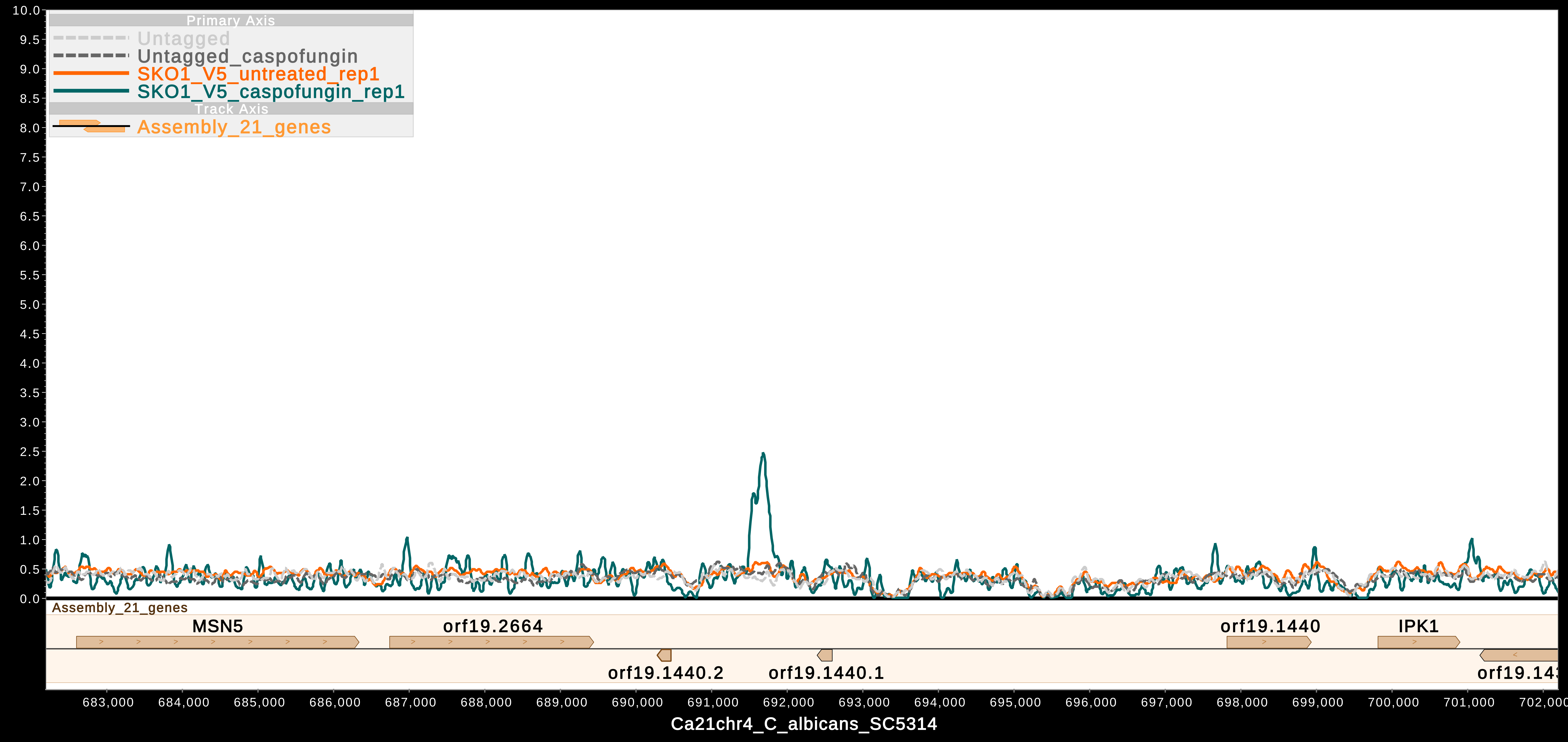

Primary Axis
Untagged
Untagged_caspofungin
SKO1_V5_untreated_rep1
SKO1_V5_caspofungin_rep1
Track Axis
Assembly_21_genes
10.0
9.5
9.0
8.5
8.0
7.5
7.0
6.5
6.0
5.5
5.0
4.5
4.0
3.5
3.0
2.5
2.0
1.5
1.0
0.5
0.0
Assembly_21_genes
MSN5
orf19.2664
orf19.1440
IPK1
orf19.1440.2
orf19.1440.1
683,000
684,000
685,000
686,000
687,000
688,000
689,000
690,000
691,000
692,000
693,000
694,000
695,000
696,000
697,000
698,000
699,000
700,000
701,000
Ca21chr4_C_albicans_SC5314

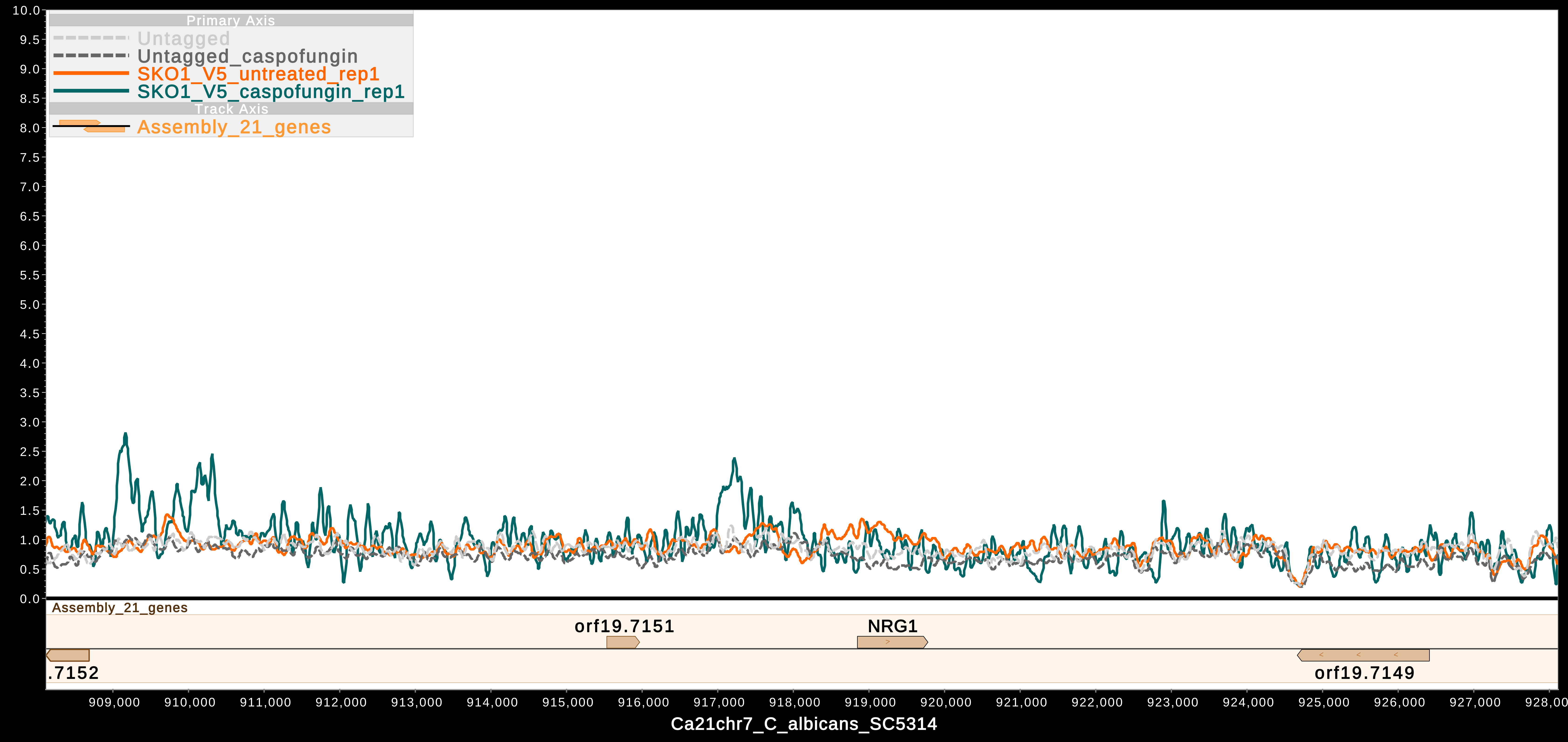
Primary Axis
Untagged
Untagged_caspofungin
SKO1_V5_untreated_rep1
SKO1_V5_caspofungin_rep1
Track Axis
Assembly_21_genes
Assembly_21_genes
orf19.7151
NRG1
.7152
orf19.7149
Ca21chr7_C_albicans_SC5314

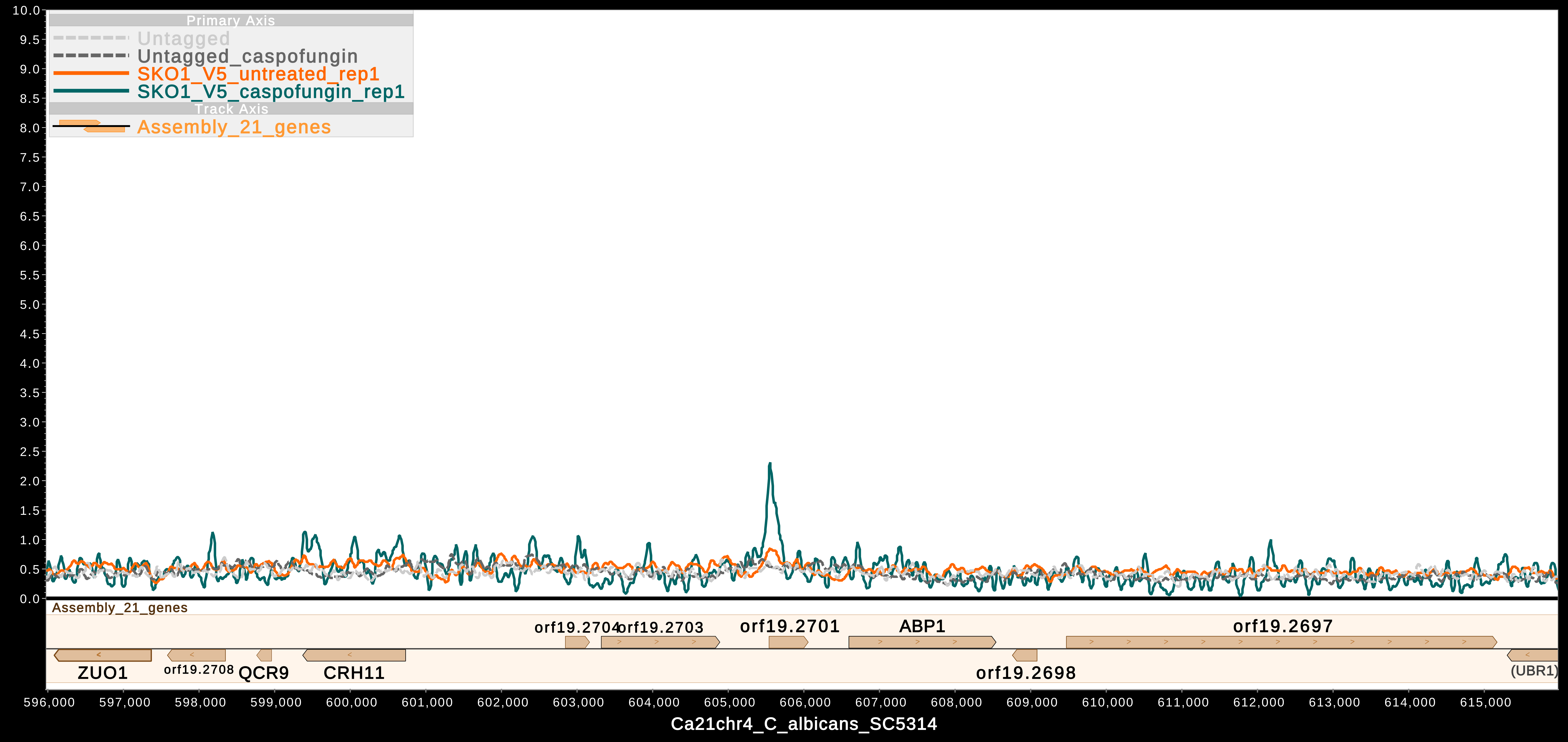
Primary Axis
Untagged
Untagged_caspofungin
SKO1_V5_untreated_rep1
SKO1_V5_caspofungin_rep1
Track Axis
Assembly_21_genes
10.0
9.5
9.0
8.5
8.0
7.5
7.0
6.5
6.0
5.5
5.0
4.5
4.0
3.5
3.0
2.5
2.0
1.5
1.0
0.5
0.0
Assembly_21_genes
orf19.2704
orf19.2703
orf19.2701
ABP1
orf19.2697
ZUO1
orf19.2708
QCR9
CRH11
orf19.2698
(UBR1)
596,000
597,000
598,000
599,000
600,000
601,000
602,000
603,000
604,000
605,000
606,000
607,000
608,000
609,000
610,000
611,000
612,000
613,000
614,000
615,000
Ca21chr4_C_albicans_SC5314

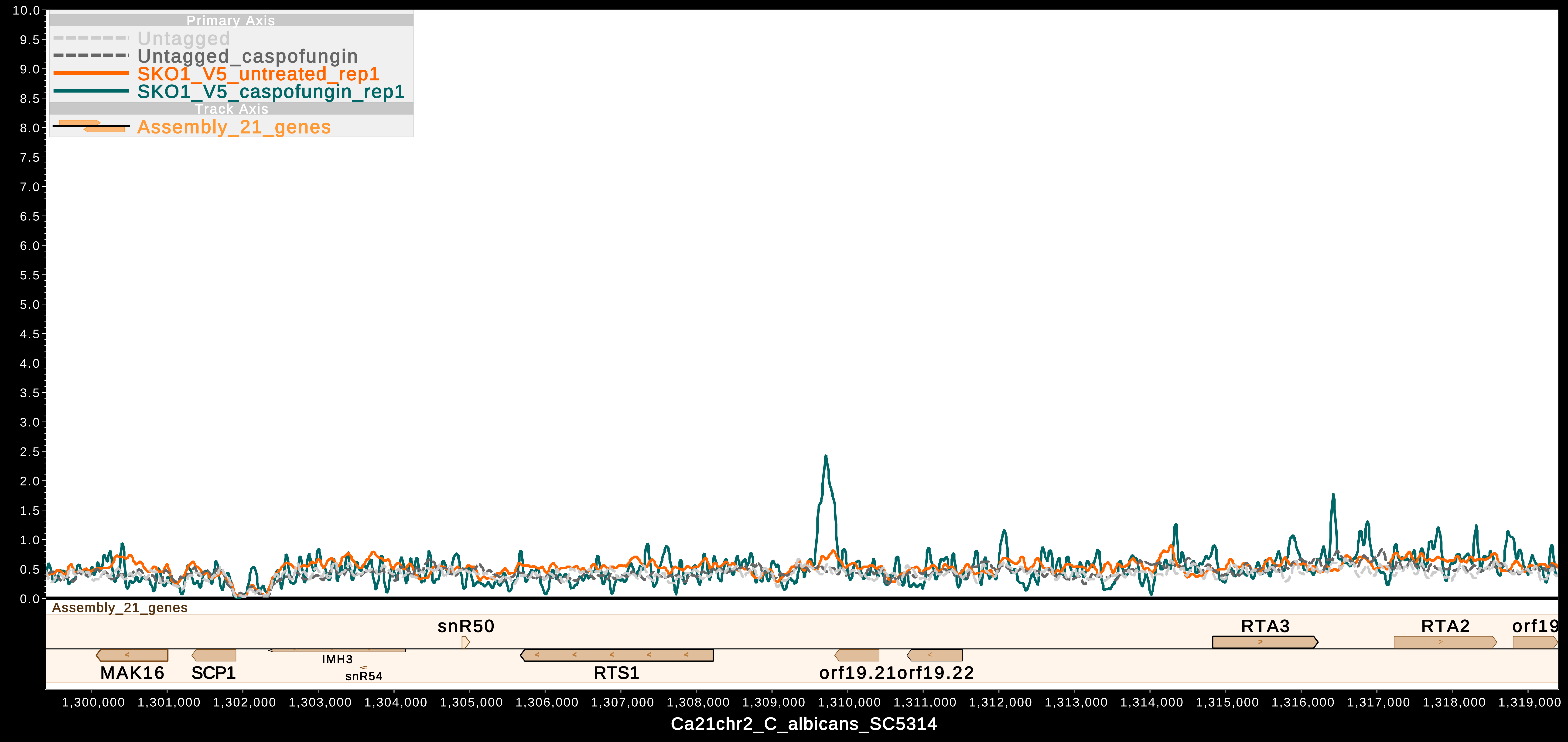

Primary Axis
Untagged
Untagged_caspofungin
SKO1_V5_untreated_rep1
SKO1_V5_caspofungin_rep1
Track Axis
Assembly_21_genes
10.0
9.5
9.0
8.5
8.0
7.5
7.0
6.5
6.0
5.5
5.0
4.5
4.0
3.5
3.0
2.5
2.0
1.5
1.0
0.5
0.0
Assembly_21_genes
snR50
RTA3
RTA2
orf19
MAK16
SCP1
IMH3
snR54
RTS1
orf19.21
orf19.22
1,300,000
1,301,000
1,302,000
1,303,000
1,304,000
1,305,000
1,306,000
1,307,000
1,308,000
1,309,000
1,310,000
1,311,000
1,312,000
1,313,000
1,314,000
1,315,000
1,316,000
1,317,000
1,318,000
1,319,000
Ca21chr2_C_albicans_SC5314

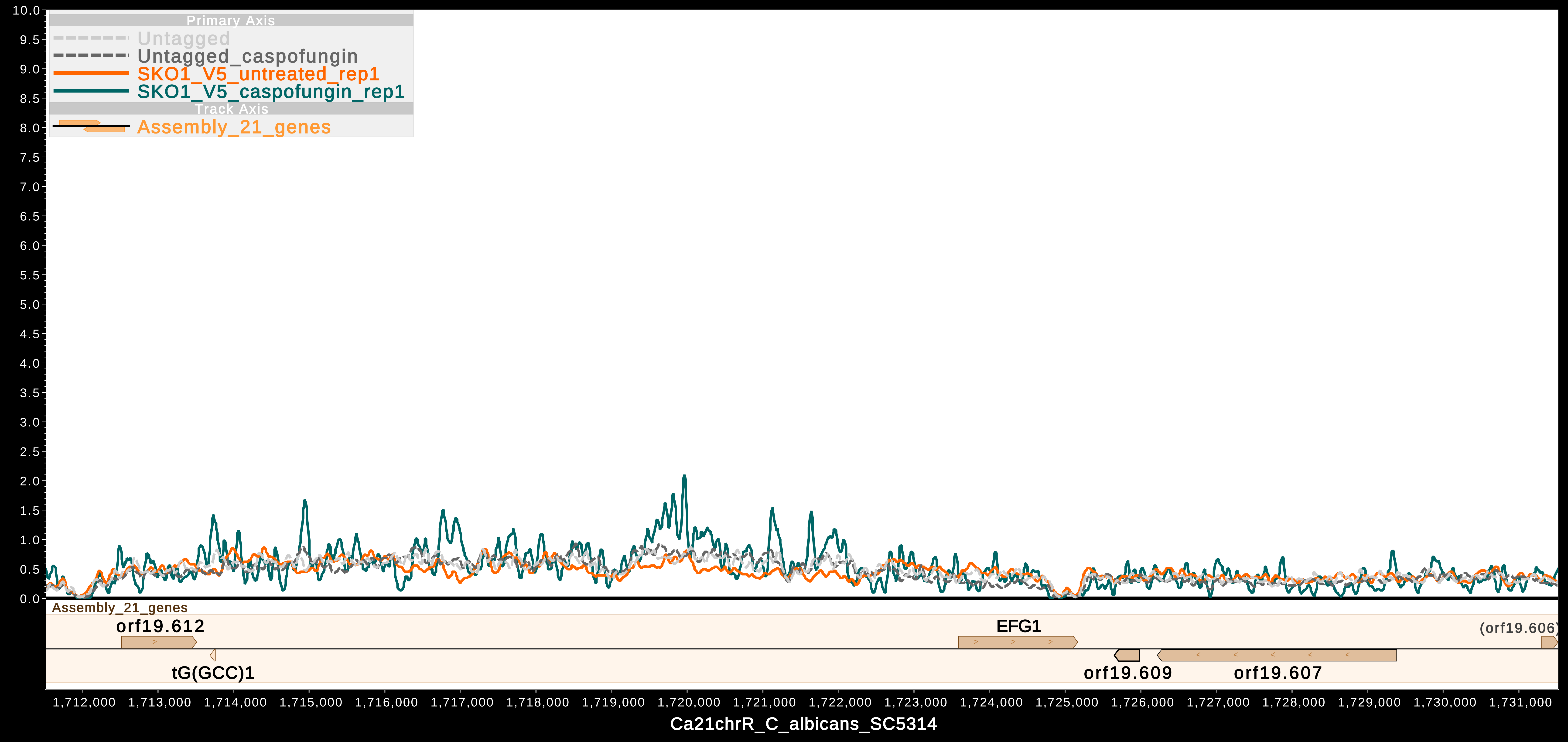

Primary Axis
Untagged
Untagged_caspofungin
SKO1_V5_untreated_rep1
SKO1_V5_caspofungin_rep1
Track Axis
Assembly_21_genes
10.0
9.5
9.0
8.5
8.0
7.5
7.0
6.5
6.0
5.5
5.0
4.5
4.0
3.5
3.0
2.5
2.0
1.5
1.0
0.5
0.0
Assembly_21_genes
orf19.612
EFG1
(orf19.606)
tG(GCC)1
orf19.609
orf19.607
1,712,000
1,713,000
1,714,000
1,715,000
1,716,000
1,717,000
1,718,000
1,719,000
1,720,000
1,721,000
1,722,000
1,723,000
1,724,000
1,725,000
1,726,000
1,727,000
1,728,000
1,729,000
1,730,000
1,731,000
Ca21chrR_C_albicans_SC5314

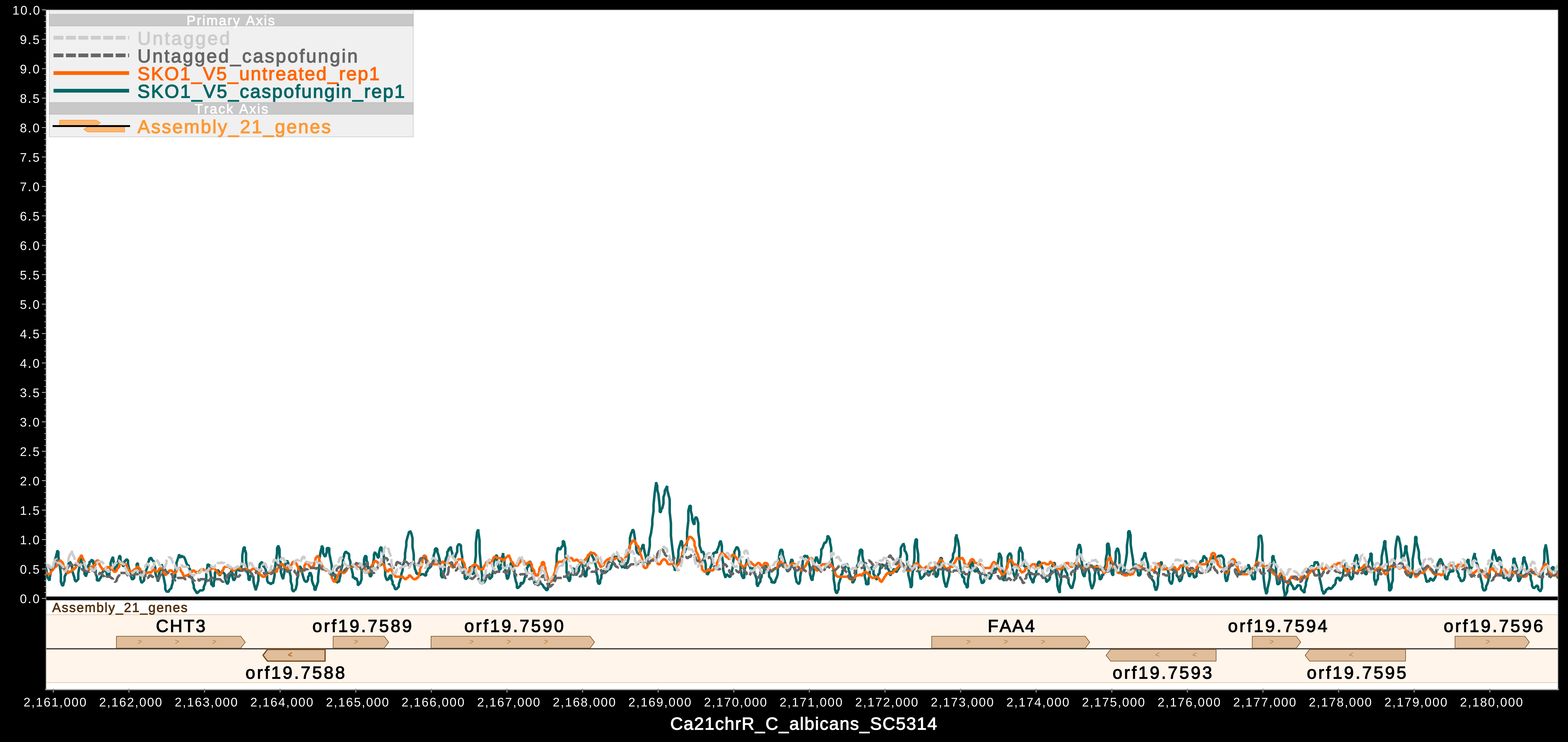

Primary Axis
Untagged
Untagged_caspofungin
SKO1_V5_untreated_rep1
SKO1_V5_caspofungin_rep1
Track Axis
Assembly_21_genes
10.0
9.5
9.0
8.5
8.0
7.5
7.0
6.5
6.0
5.5
5.0
4.5
4.0
3.5
3.0
2.5
2.0
1.5
1.0
0.5
0.0
Assembly_21_genes
CHT3
orf19.7588
orf19.7589
orf19.7590
FAA4
orf19.7593
orf19.7594
orf19.7595
orf19.7596
2,161,000
2,162,000
2,163,000
2,164,000
2,165,000
2,166,000
2,167,000
2,168,000
2,169,000
2,170,000
2,171,000
2,172,000
2,173,000
2,174,000
2,175,000
2,176,000
2,177,000
2,178,000
2,179,000
2,180,000
Ca21chrR_C_albicans_SC5314

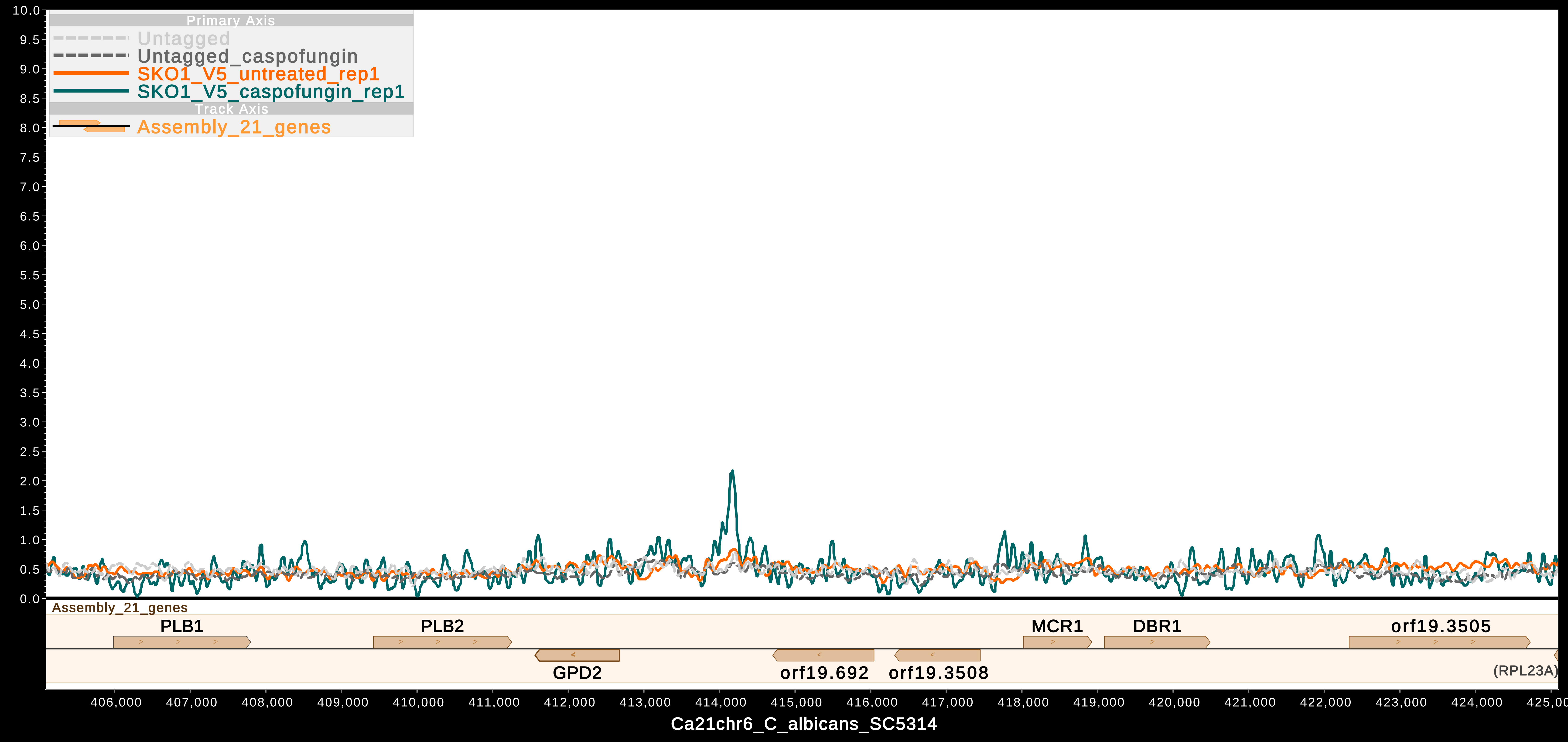
Primary Axis
Untagged
Untagged_caspofungin
SKO1_V5_untreated_rep1
SKO1_V5_caspofungin_rep1
Track Axis
Assembly_21_genes
10.0
9.5
9.0
8.5
8.0
7.5
7.0
6.5
6.0
5.5
5.0
4.5
4.0
3.5
3.0
2.5
2.0
1.5
1.0
0.5
0.0
Assembly_21_genes
PLB1
PLB2
GPD2
orf19.692
orf19.3508
MCR1
DBR1
orf19.3505
(RPL23A)
406,000
407,000
408,000
409,000
410,000
411,000
412,000
413,000
414,000
415,000
416,000
417,000
418,000
419,000
420,000
421,000
422,000
423,000
424,000
Ca21chr6_C_albicans_SC5314

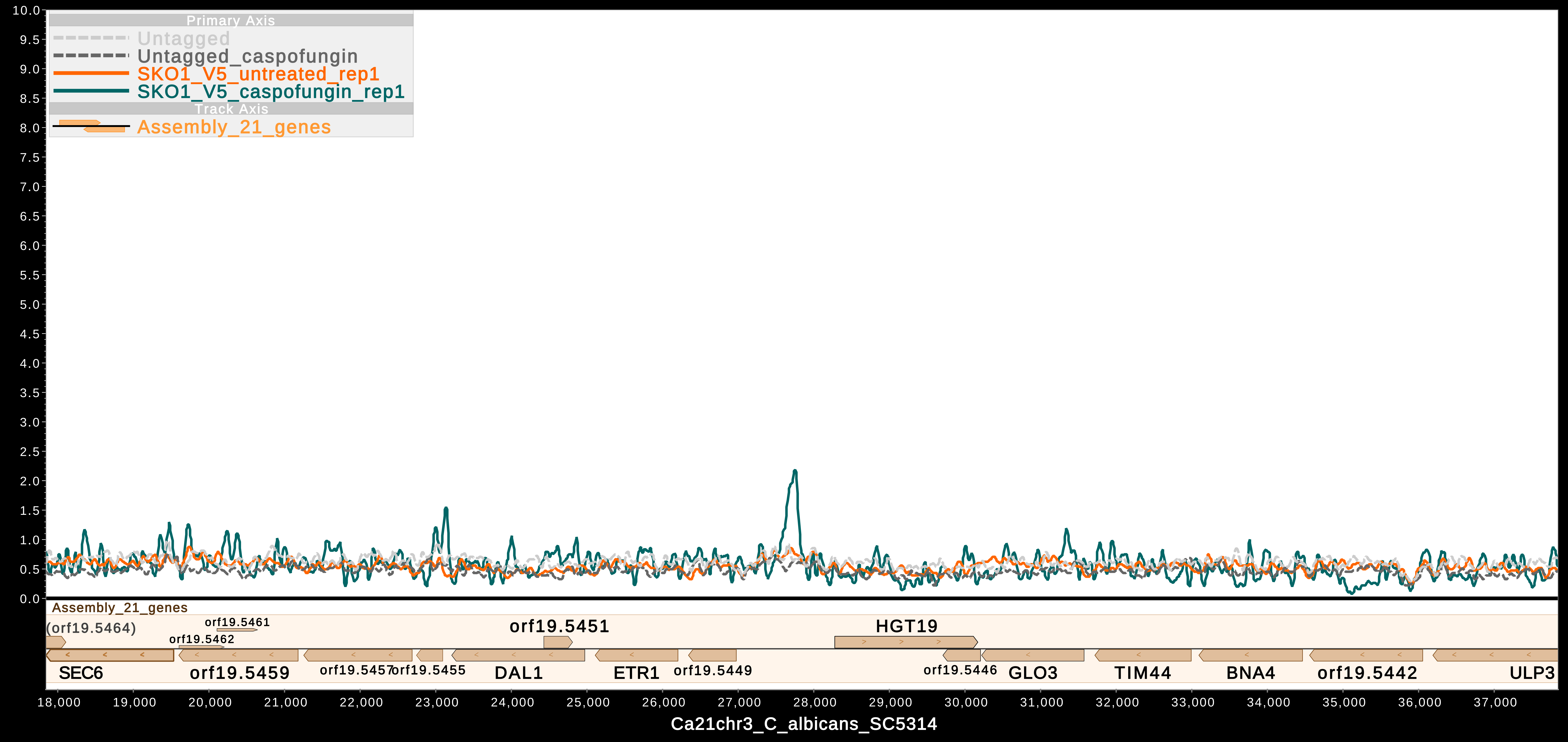

Primary Axis
Untagged
Untagged_caspofungin
SKO1_V5_untreated_rep1
SKO1_V5_caspofungin_rep1
Track Axis
Assembly_21_genes
10.0
9.5
9.0
8.5
8.0
7.5
7.0
6.5
6.0
5.5
5.0
4.5
4.0
3.5
3.0
2.5
2.0
1.5
1.0
0.5
0.0
Assembly_21_genes
(orf19.5464)
orf19.5461
orf19.5462
orf19.5451
HGT19
SEC6
orf19.5459
orf19.5457
orf19.5455
DAL1
ETR1
orf19.5449
orf19.5446
GLO3
TIM44
BNA4
orf19.5442
ULP3
18,000
19,000
20,000
21,000
22,000
23,000
24,000
25,000
26,000
27,000
28,000
29,000
30,000
31,000
32,000
33,000
34,000
35,000
36,000
37,000
Ca21chr3_C_albicans_SC5314

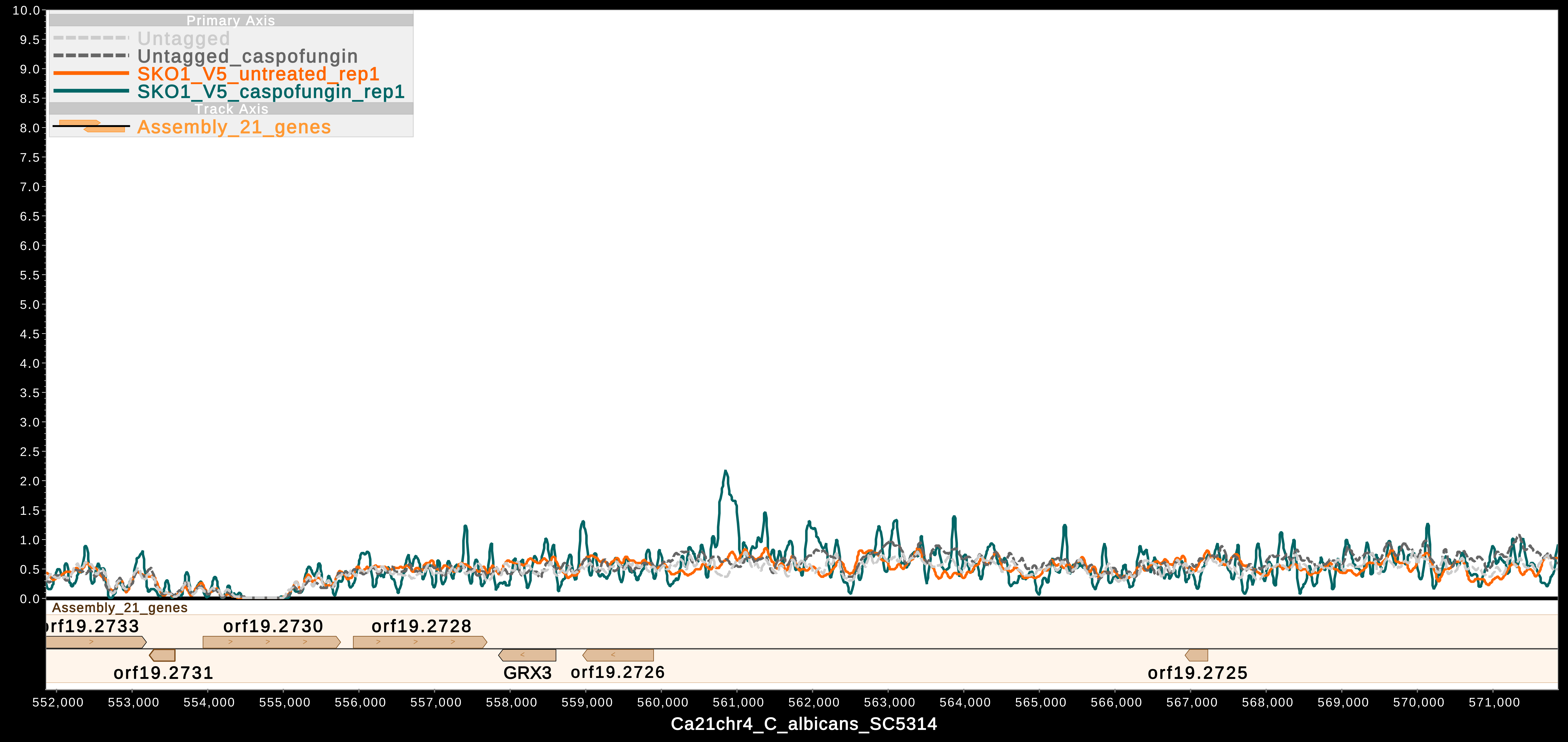

Primary Axis
Untagged
Untagged_caspofungin
SKO1_V5_untreated_rep1
SKO1_V5_caspofungin_rep1
Track Axis
Assembly_21_genes
10.0
9.5
9.0
8.5
8.0
7.5
7.0
6.5
6.0
5.5
5.0
4.5
4.0
3.5
3.0
2.5
2.0
1.5
1.0
0.5
0.0
Assembly_21_genes
orf19.2733
orf19.2730
orf19.2728
orf19.2731
GRX3
orf19.2726
orf19.2725
552,000
553,000
554,000
555,000
556,000
557,000
558,000
559,000
560,000
561,000
562,000
563,000
564,000
565,000
566,000
567,000
568,000
569,000
570,000
571,000
Ca21chr4_C_albicans_SC5314

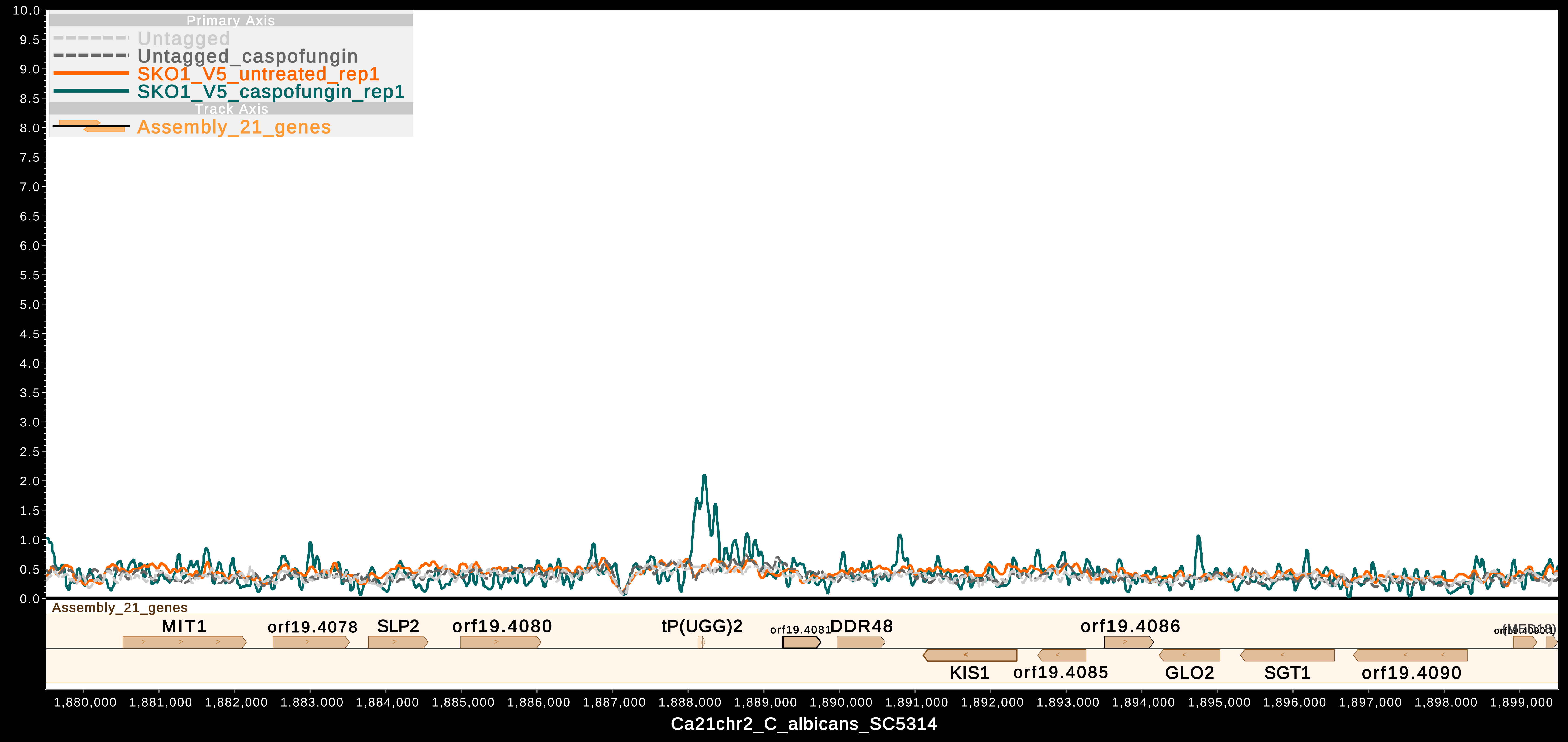
Primary Axis
Untagged
Untagged_caspofungin
SKO1_V5_untreated_rep1
SKO1_V5_caspofungin_rep1
Track Axis
Assembly_21_genes
10.0
9.5
9.0
8.5
8.0
7.5
7.0
6.5
6.0
5.5
5.0
4.5
4.0
3.5
3.0
2.5
2.0
1.5
1.0
0.5
0.0
Assembly_21_genes
MIT1
orf19.4078
SLP2
orf19.4080
tP(UGG)2
orf19.4081
DDR48
orf19.4086
KIS1
orf19.4085
GLO2
SGT1
orf19.4090
1,880,000
1,881,000
1,882,000
1,883,000
1,884,000
1,885,000
1,886,000
1,887,000
1,888,000
1,889,000
1,890,000
1,891,000
1,892,000
1,893,000
1,894,000
1,895,000
1,896,000
1,897,000
1,898,000
1,899,000
Ca21chr2_C_albicans_SC5314

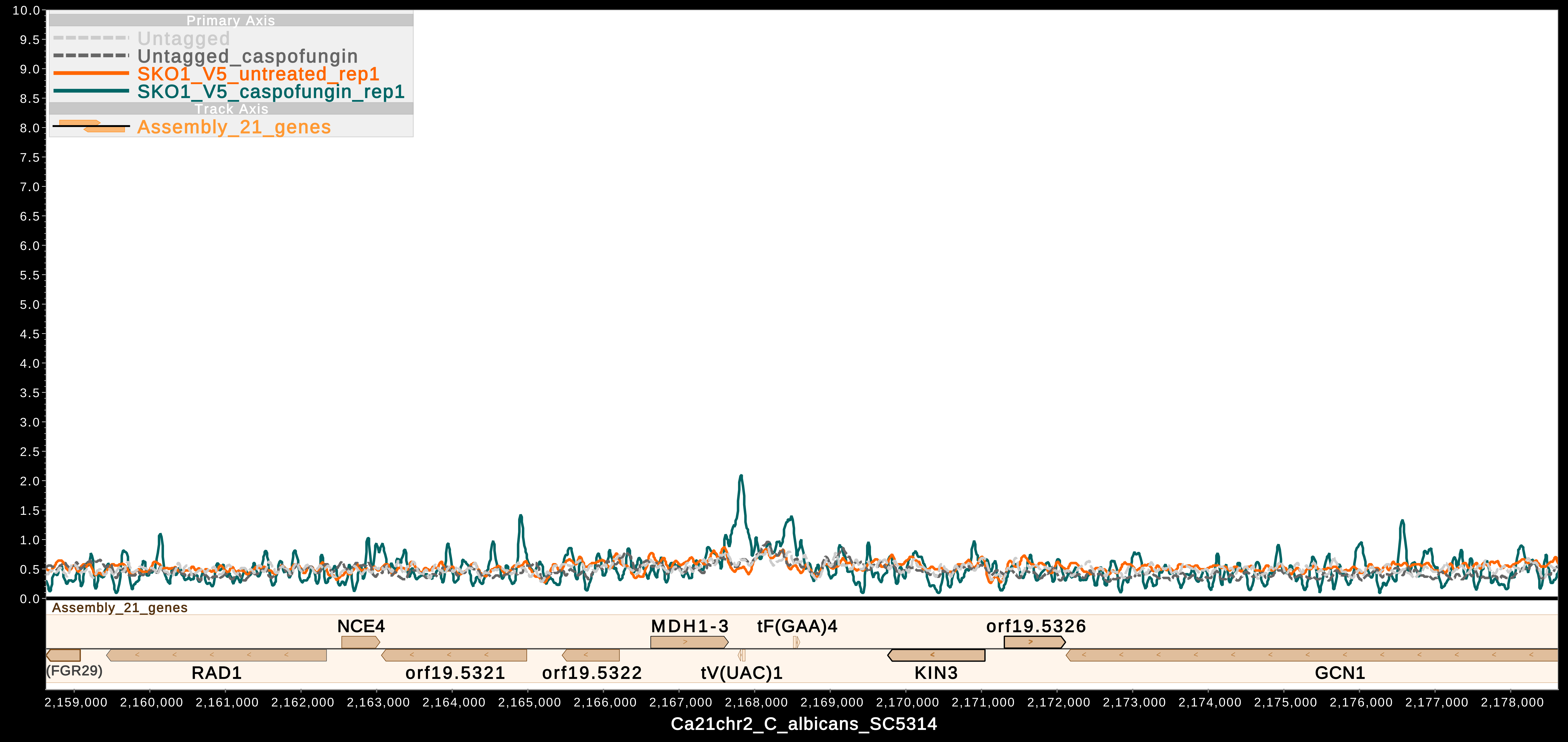
Primary Axis
Untagged
Untagged_caspofungin
SKO1_V5_untreated_rep1
SKO1_V5_caspofungin_rep1
Track Axis
Assembly_21_genes
10.0
9.5
9.0
8.5
8.0
7.5
7.0
6.5
6.0
5.5
5.0
4.5
4.0
3.5
3.0
2.5
2.0
1.5
1.0
0.5
0.0
Assembly_21_genes
NCE4
MDH1-3
tF(GAA)4
orf19.5326
(FGR29)
RAD1
orf19.5321
orf19.5322
tV(UAC)1
KIN3
GCN1
2,159,000
2,160,000
2,161,000
2,162,000
2,163,000
2,164,000
2,165,000
2,166,000
2,167,000
2,168,000
2,169,000
2,170,000
2,171,000
2,172,000
2,173,000
2,174,000
2,175,000
2,176,000
2,177,000
2,178,000
Ca21chr2_C_albicans_SC5314

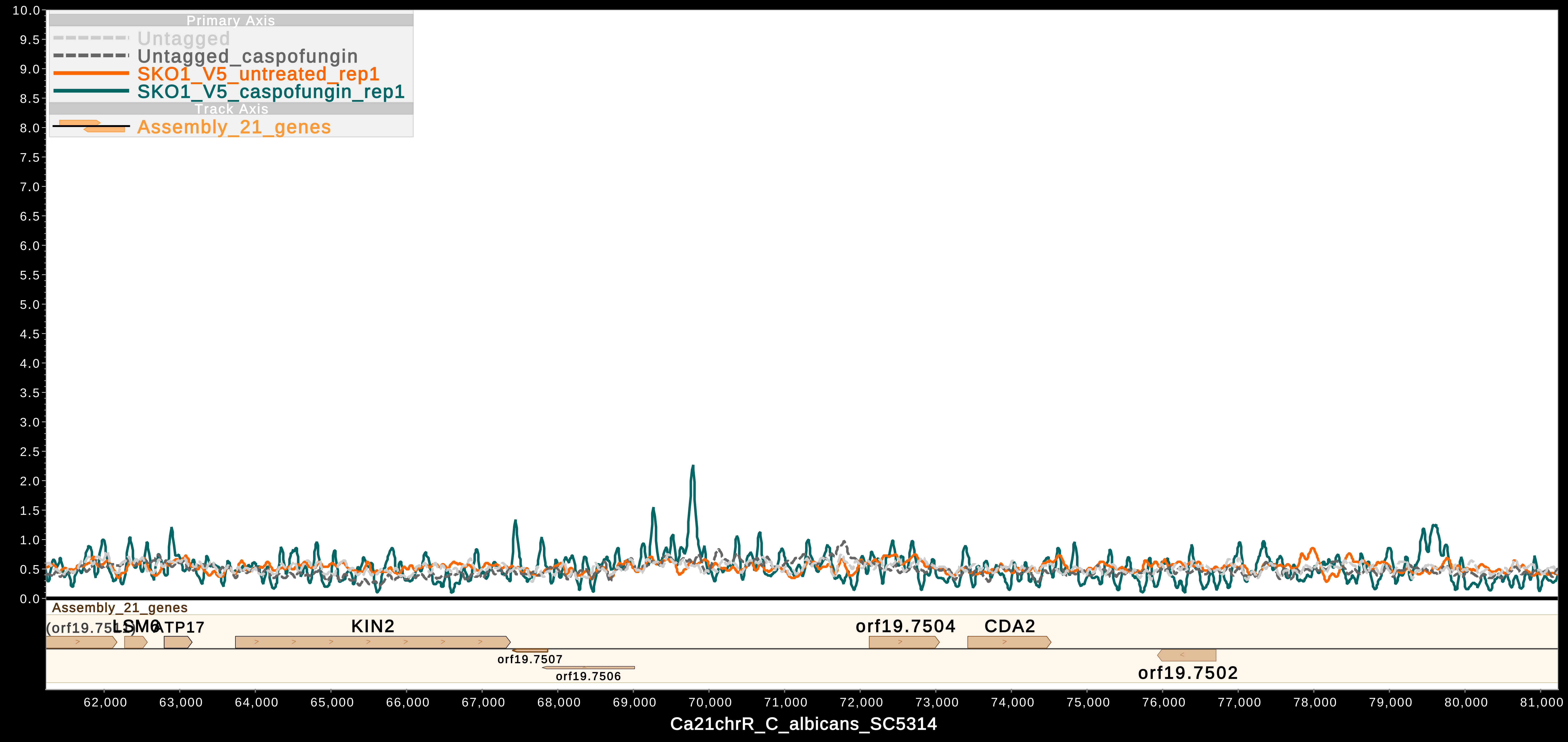

Primary Axis
Untagged
Untagged_caspofungin
SKO1_V5_untreated_rep1
SKO1_V5_caspofungin_rep1
Track Axis
Assembly_21_genes
10.0
9.5
9.0
8.5
8.0
7.5
7.0
6.5
6.0
5.5
5.0
4.5
4.0
3.5
3.0
2.5
2.0
1.5
1.0
0.5
0.0
Assembly_21_genes
LSM6
ATP17
KIN2
orf19.7504
CDA2
orf19.7507
orf19.7506
orf19.7502
62,000
63,000
64,000
65,000
66,000
67,000
68,000
69,000
70,000
71,000
72,000
73,000
74,000
75,000
76,000
77,000
78,000
79,000
80,000
81,000
Ca21chrR_C_albicans_SC5314

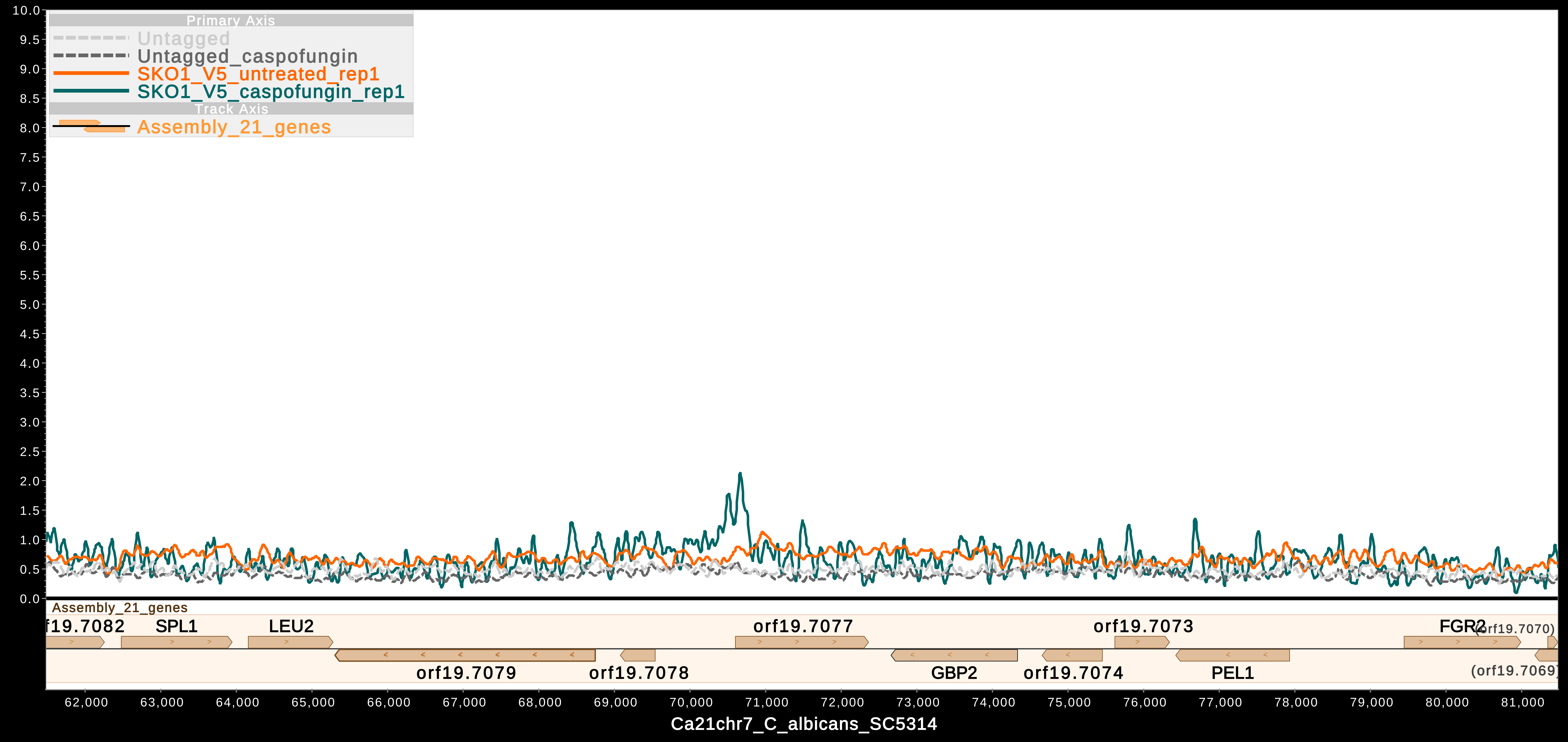

Primary Axis
Untagged
Untagged_caspofungin
SKO1_V5_untreated_rep1
SKO1_V5_caspofungin_rep1
Track Axis
Assembly_21_genes
10.0
9.5
9.0
8.5
8.0
7.5
7.0
6.5
6.0
5.5
5.0
4.5
4.0
3.5
3.0
2.5
2.0
1.5
1.0
0.5
0.0
Assembly_21_genes
f19.7082
SPL1
LEU2
orf19.7077
orf19.7073
FGR2
(orf19.7070)
orf19.7079
orf19.7078
GBP2
orf19.7074
PEL1
(orf19.7069)
62,000
63,000
64,000
65,000
66,000
67,000
68,000
69,000
70,000
71,000
72,000
73,000
74,000
75,000
76,000
77,000
78,000
79,000
80,000
81,000
Ca21chr7_C_albicans_SC5314

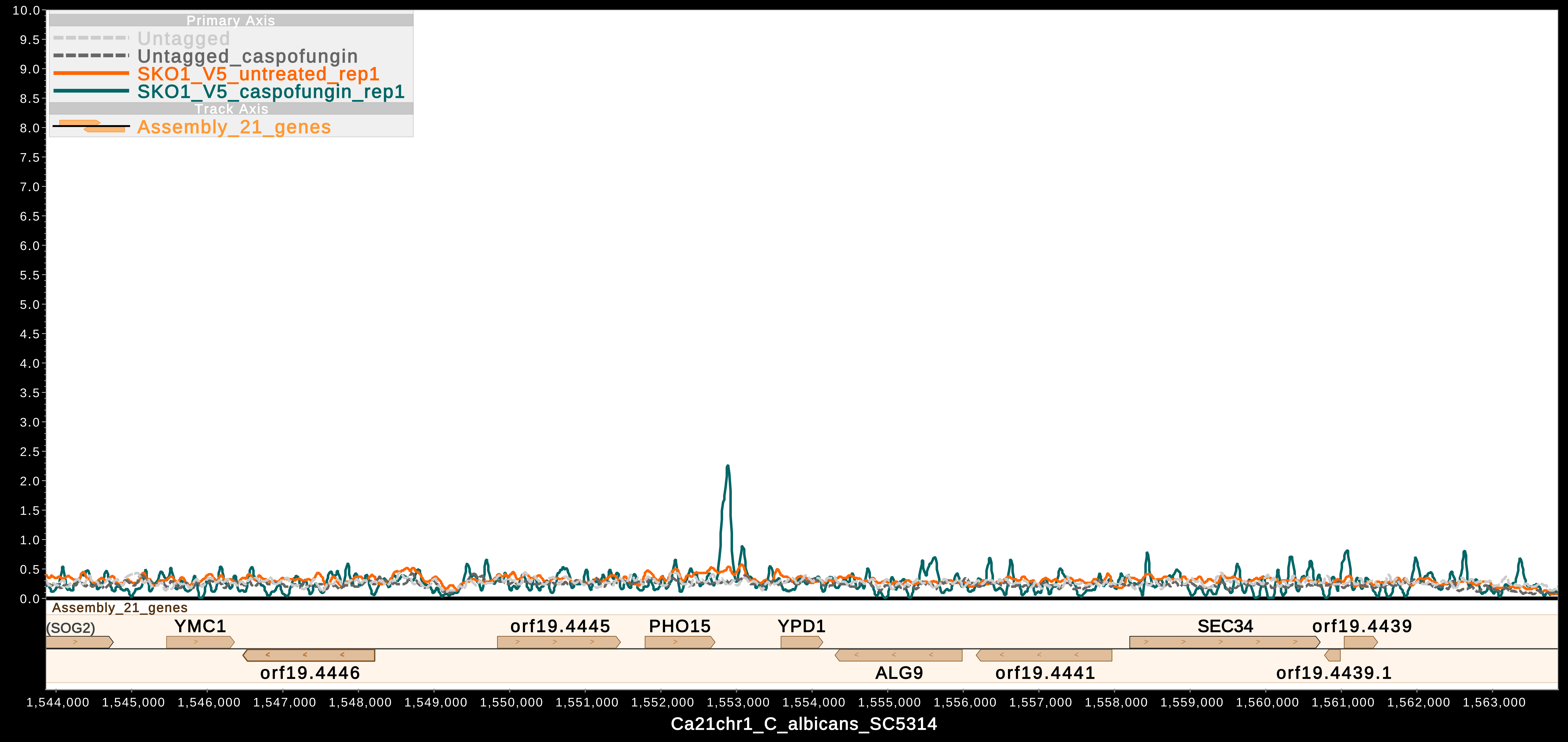

Primary Axis
Untagged
Untagged_caspofungin
SKO1_V5_untreated_rep1
SKO1_V5_caspofungin_rep1
Track Axis
Assembly_21_genes
10.0
9.5
9.0
8.5
8.0
7.5
7.0
6.5
6.0
5.5
5.0
4.5
4.0
3.5
3.0
2.5
2.0
1.5
1.0
0.5
0.0
Assembly_21_genes
(SOG2)
YMC1
orf19.4446
orf19.4445
PHO15
YPD1
ALG9
orf19.4441
SEC34
orf19.4439
orf19.4439.1
1,544,000
1,545,000
1,546,000
1,547,000
1,548,000
1,549,000
1,550,000
1,551,000
1,552,000
1,553,000
1,554,000
1,555,000
1,556,000
1,557,000
1,558,000
1,559,000
1,560,000
1,561,000
1,562,000
1,563,000
Ca21chr1_C_albicans_SC5314

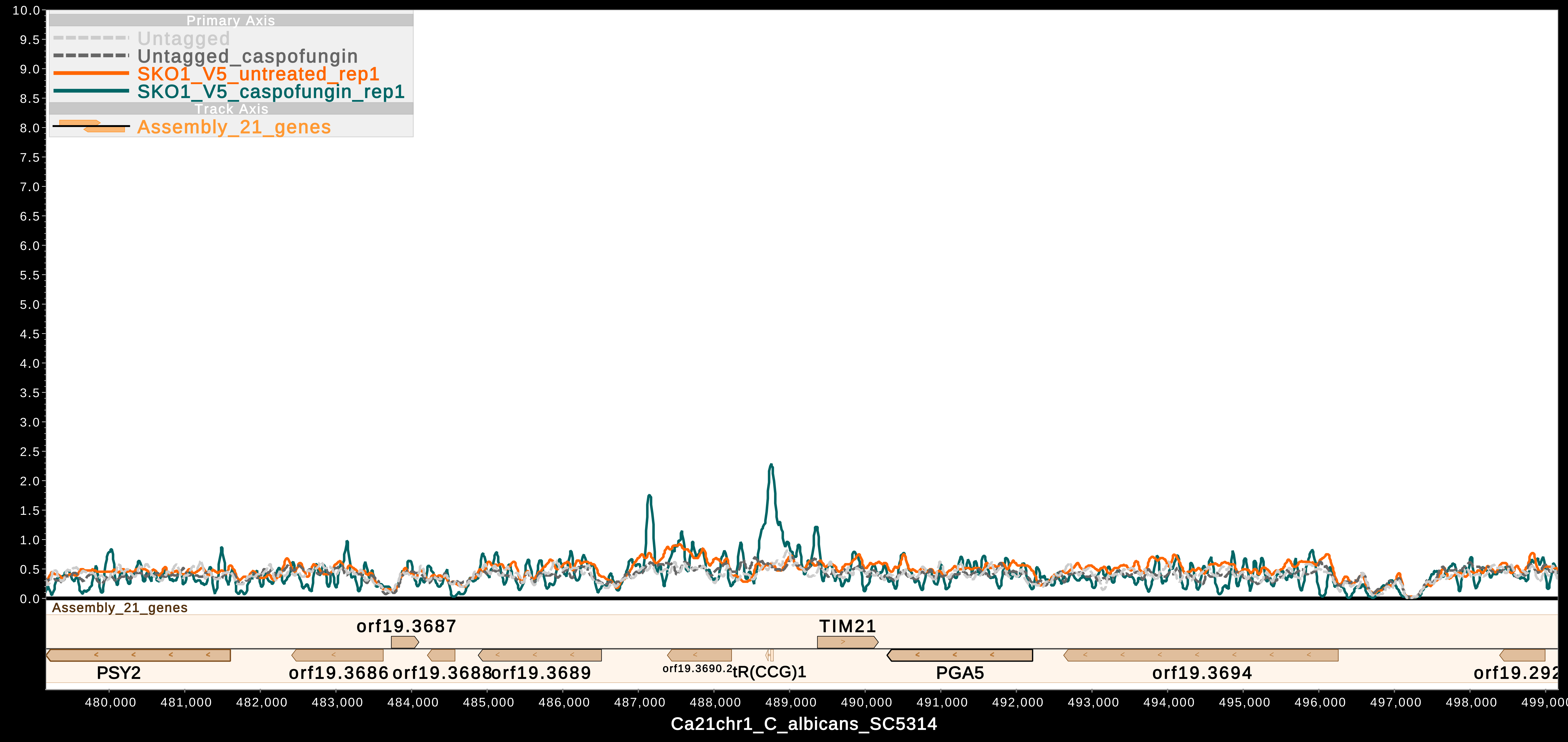

Primary Axis
Untagged
Untagged_caspofungin
SKO1_V5_untreated_rep1
SKO1_V5_caspofungin_rep1
Track Axis
Assembly_21_genes
10.0
9.5
9.0
8.5
8.0
7.5
7.0
6.5
6.0
5.5
5.0
4.5
4.0
3.5
3.0
2.5
2.0
1.5
1.0
0.5
0.0
Assembly_21_genes
orf19.3687
TIM21
PSY2
orf19.3686
orf19.3688
orf19.3689
orf19.3690.2
tR(CCG)1
PGA5
orf19.3694
480,000
481,000
482,000
483,000
484,000
485,000
486,000
487,000
488,000
489,000
490,000
491,000
492,000
493,000
494,000
495,000
496,000
497,000
498,000
Ca21chr1_C_albicans_SC5314

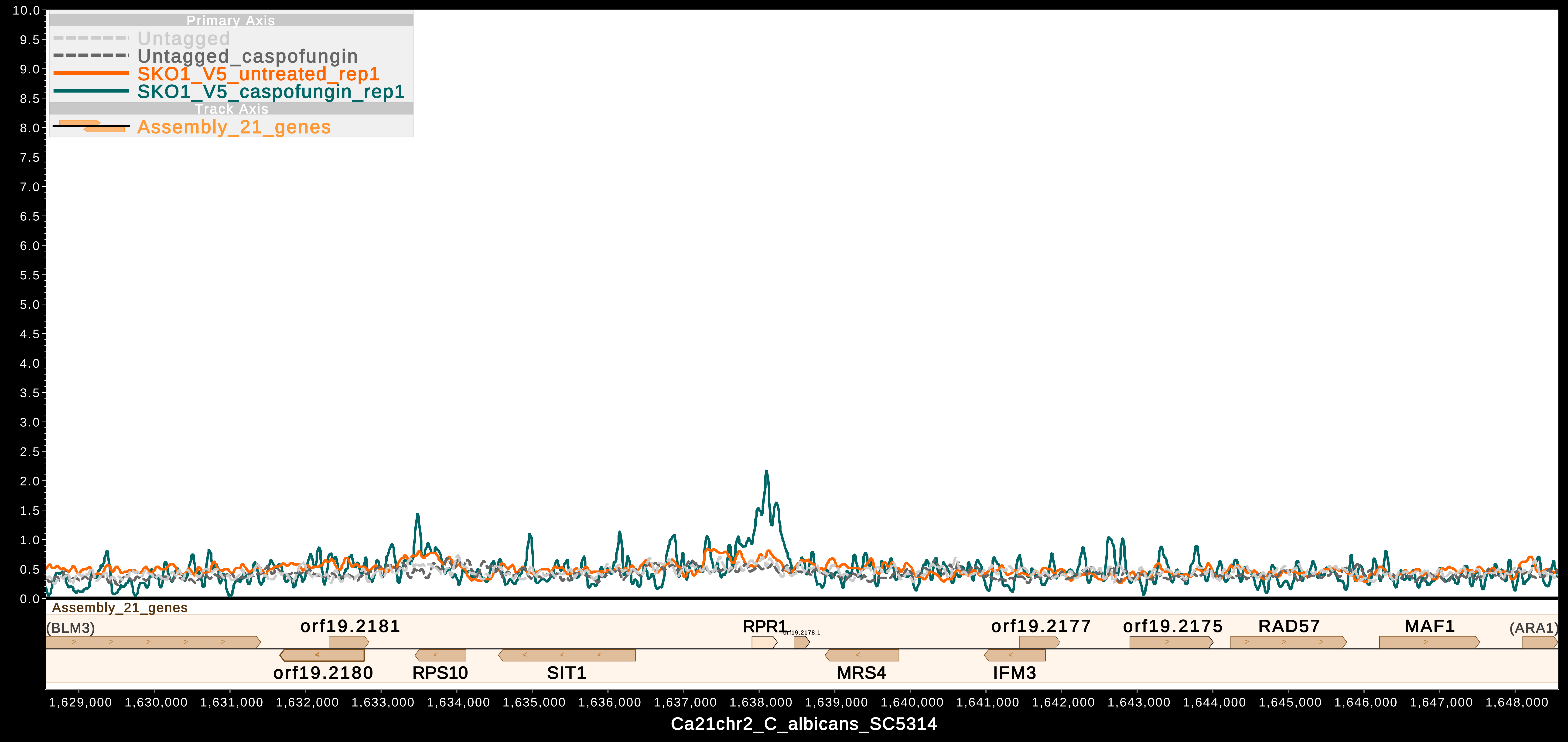
Primary Axis
Untagged
Untagged_caspofungin
SKO1_V5_untreated_rep1
SKO1_V5_caspofungin_rep1
Track Axis
Assembly_21_genes
10.0
9.5
9.0
8.5
8.0
7.5
7.0
6.5
6.0
5.5
5.0
4.5
4.0
3.5
3.0
2.5
2.0
1.5
1.0
0.5
0.0
Assembly_21_genes
(BLM3)
orf19.2181
RPR1
orf19.2178.1
orf19.2177
orf19.2175
RAD57
MAF1
(ARA1)
orf19.2180
RPS10
SIT1
MRS4
IFM3
1,629,000
1,630,000
1,631,000
1,632,000
1,633,000
1,634,000
1,635,000
1,636,000
1,637,000
1,638,000
1,639,000
1,640,000
1,641,000
1,642,000
1,643,000
1,644,000
1,645,000
1,646,000
1,647,000
1,648,000
Ca21chr2_C_albicans_SC5314

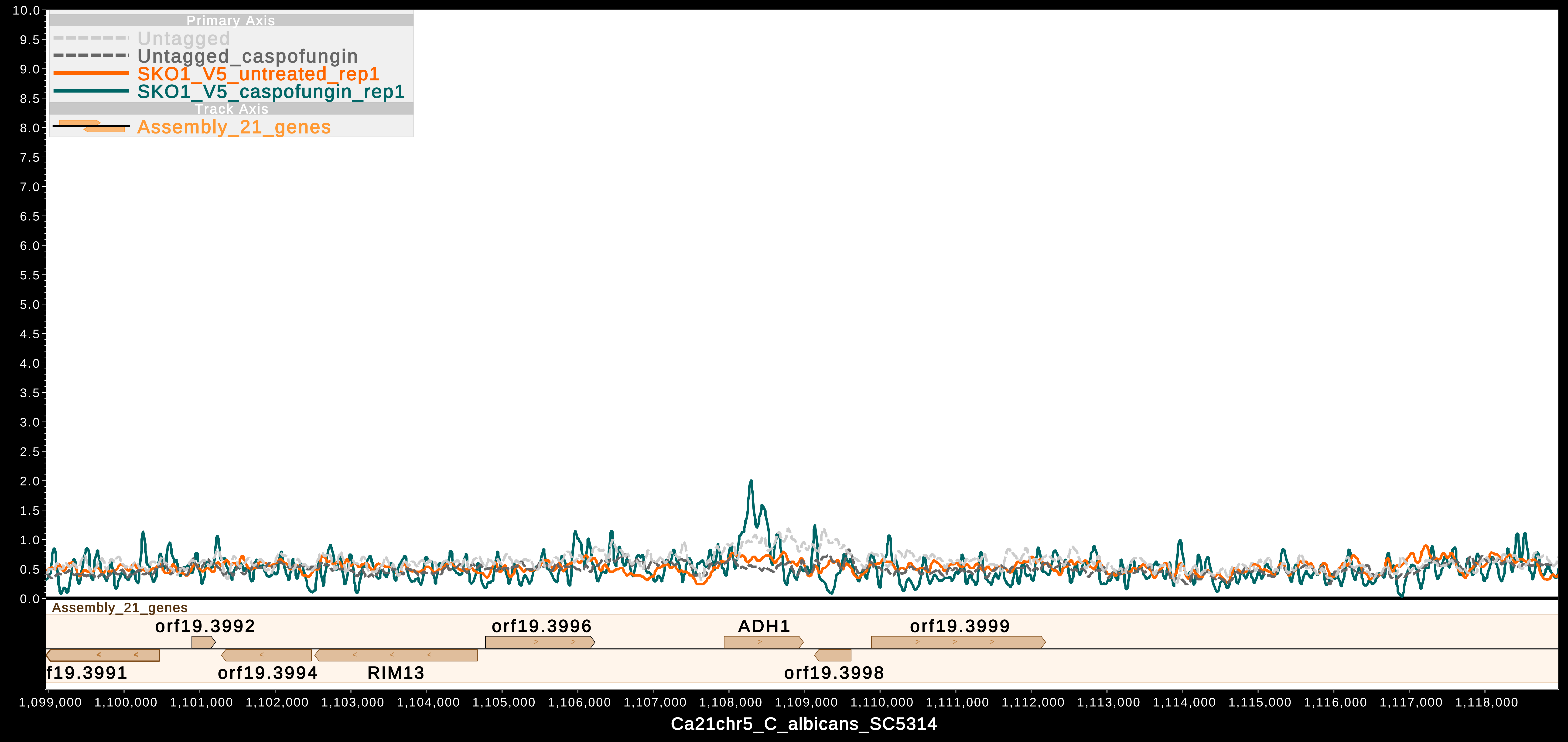
Primary Axis
Untagged
Untagged_caspofungin
SKO1_V5_untreated_rep1
SKO1_V5_caspofungin_rep1
Track Axis
Assembly_21_genes
10.0
9.5
9.0
8.5
8.0
7.5
7.0
6.5
6.0
5.5
5.0
4.5
4.0
3.5
3.0
2.5
2.0
1.5
1.0
0.5
0.0
Assembly_21_genes
orf19.3992
orf19.3996
ADH1
orf19.3999
f19.3991
orf19.3994
RIM13
orf19.3998
1,099,000
1,100,000
1,101,000
1,102,000
1,103,000
1,104,000
1,105,000
1,106,000
1,107,000
1,108,000
1,109,000
1,110,000
1,111,000
1,112,000
1,113,000
1,114,000
1,115,000
1,116,000
1,117,000
1,118,000
Ca21chr5_C_albicans_SC5314

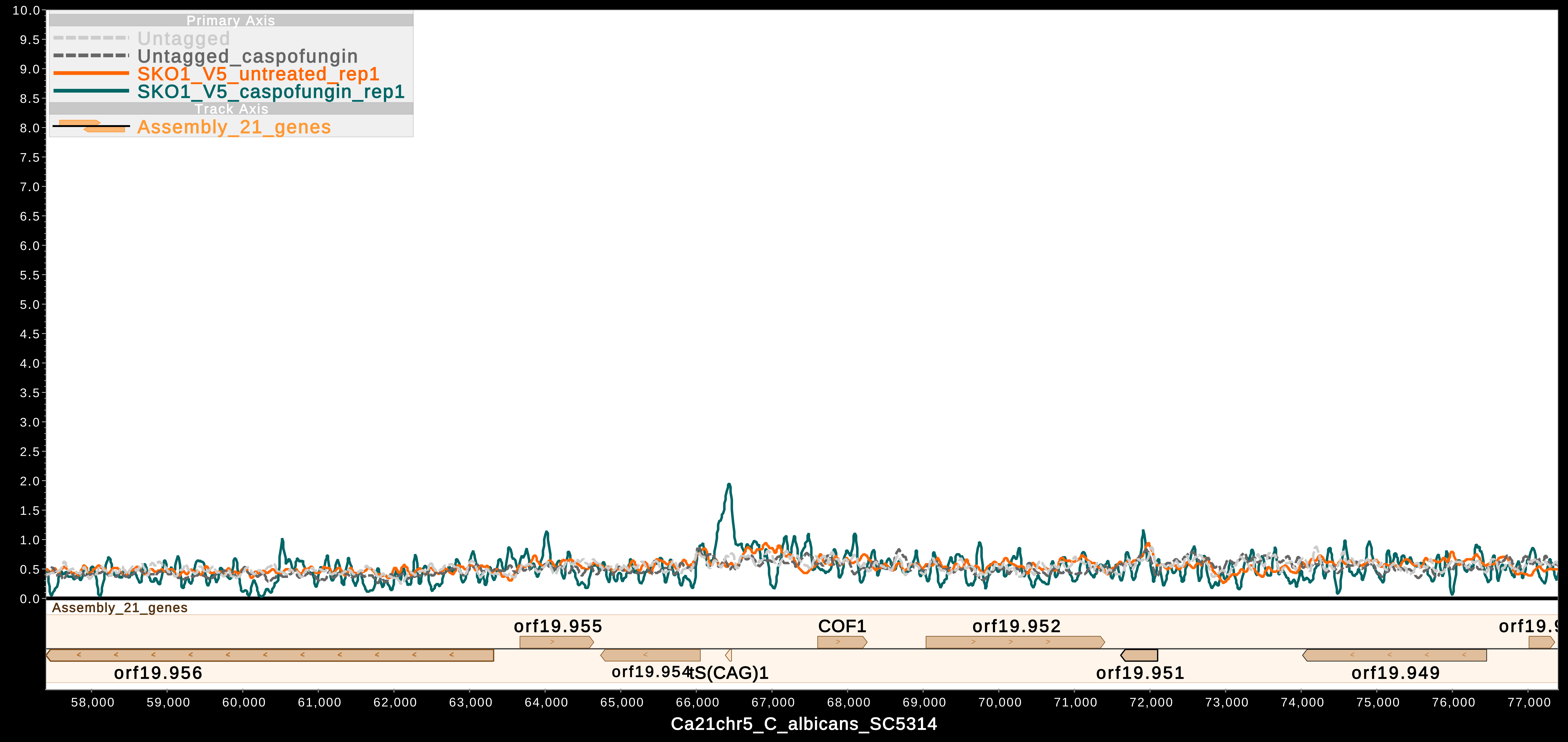

Primary Axis
Untagged
Untagged_caspofungin
SKO1_V5_untreated_rep1
SKO1_V5_caspofungin_rep1
Track Axis
Assembly_21_genes
10.0
9.5
9.0
8.5
8.0
7.5
7.0
6.5
6.0
5.5
5.0
4.5
4.0
3.5
3.0
2.5
2.0
1.5
1.0
0.5
0.0
Assembly_21_genes
orf19.955
COF1
orf19.952
orf19.956
orf19.954
tS(CAG)1
orf19.951
orf19.949
58,000
59,000
60,000
61,000
62,000
63,000
64,000
65,000
66,000
67,000
68,000
69,000
70,000
71,000
72,000
73,000
74,000
75,000
76,000
77,000
Ca21chr5_C_albicans_SC5314

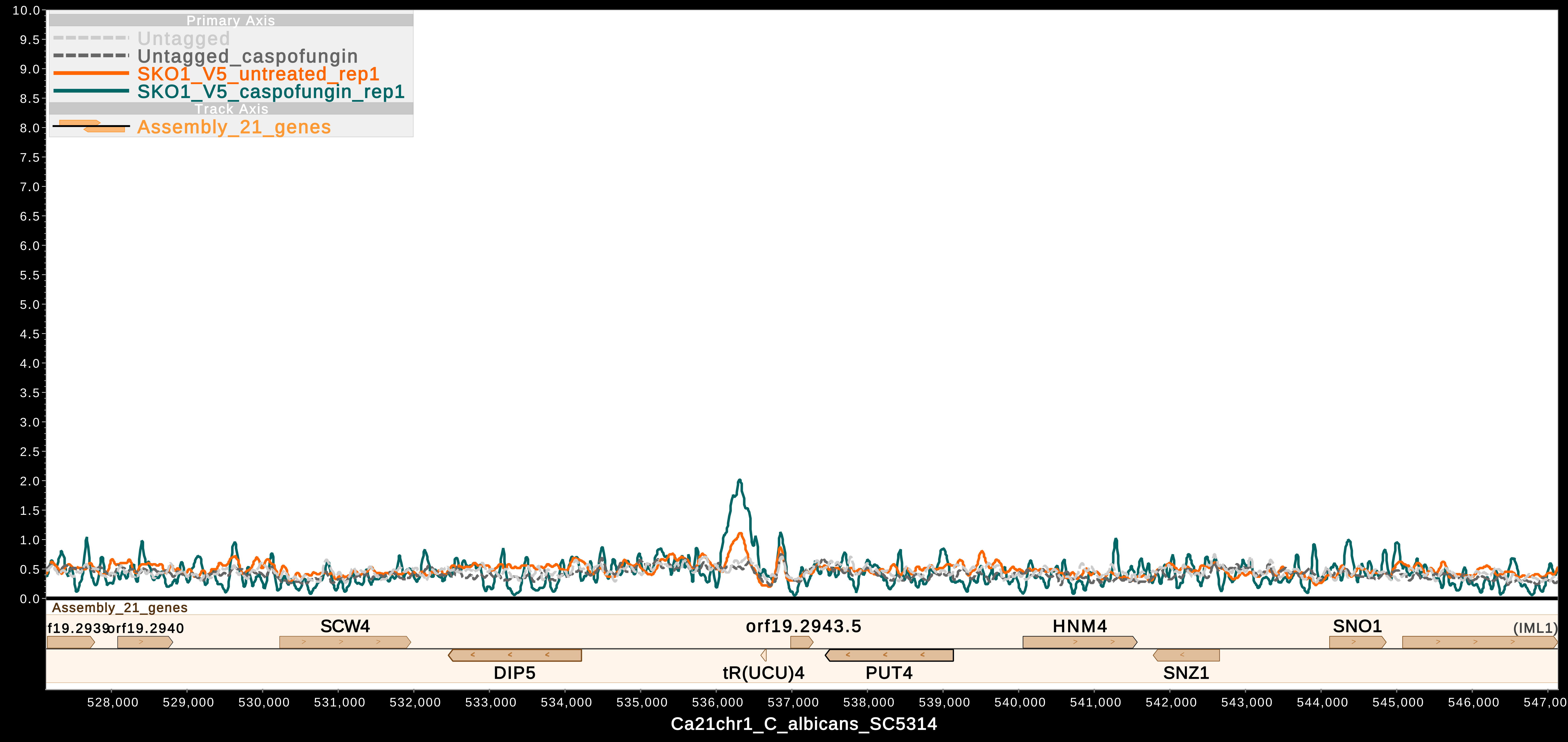
Primary Axis
Untagged
Untagged_caspofungin
SKO1_V5_untreated_rep1
SKO1_V5_caspofungin_rep1
Track Axis
Assembly_21_genes
10.0
9.5
9.0
8.5
8.0
7.5
7.0
6.5
6.0
5.5
5.0
4.5
4.0
3.5
3.0
2.5
2.0
1.5
1.0
0.5
0.0
Assembly_21_genes
f19.2939
orf19.2940
SCW4
orf19.2943.5
HNM4
SNO1
(IML1)
DIP5
tR(UCU)4
PUT4
SNZ1
528,000
529,000
530,000
531,000
532,000
533,000
534,000
535,000
536,000
537,000
538,000
539,000
540,000
541,000
542,000
543,000
544,000
545,000
546,000
547,00
Ca21chr1_C_albicans_SC5314

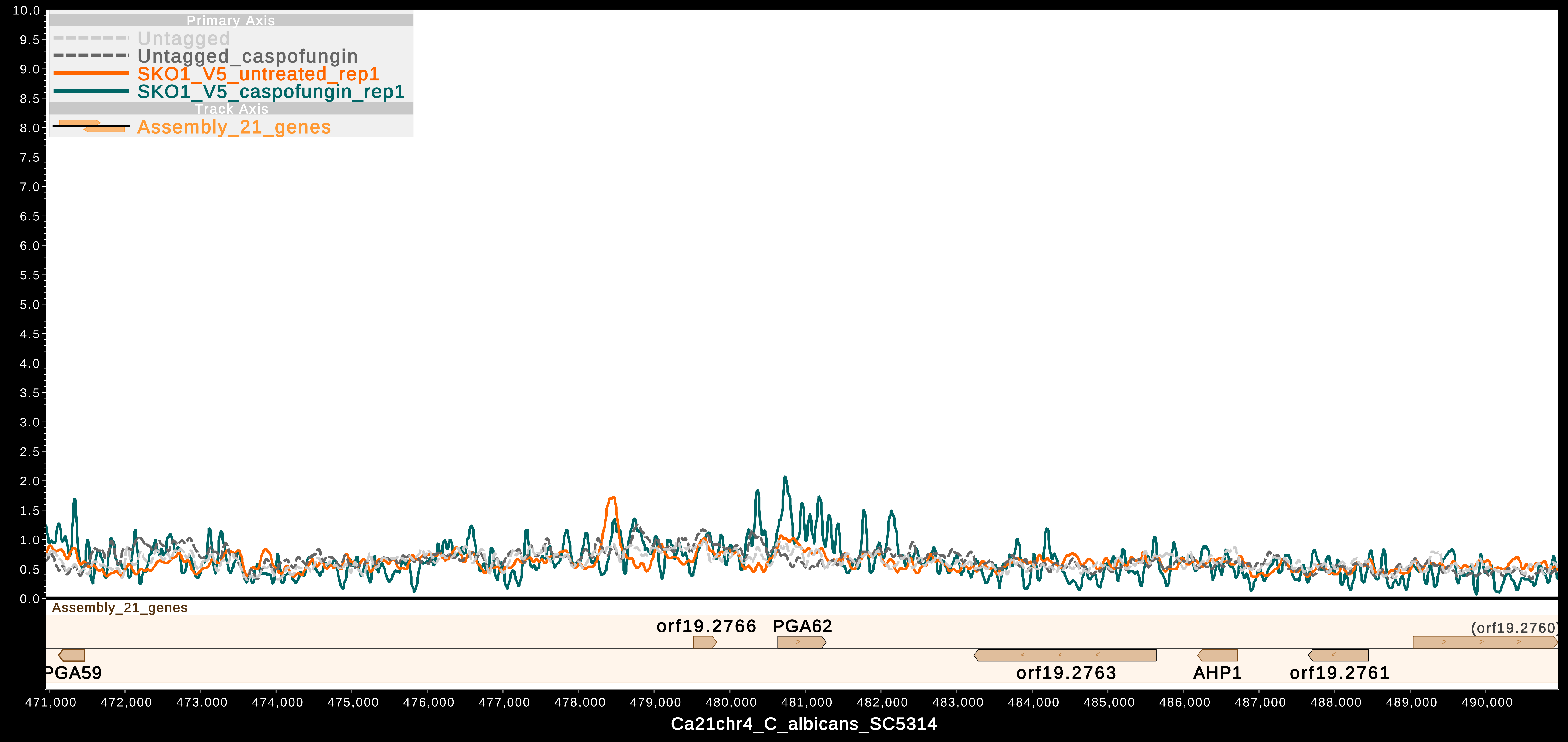

Primary Axis
Untagged
Untagged_caspofungin
SKO1_V5_untreated_rep1
SKO1_V5_caspofungin_rep1
Track Axis
Assembly_21_genes
10.0
9.5
9.0
8.5
8.0
7.5
7.0
6.5
6.0
5.5
5.0
4.5
4.0
3.5
3.0
2.5
2.0
1.5
1.0
0.5
0.0
Assembly_21_genes
orf19.2766
PGA62
(orf19.2760)
PGA59
orf19.2763
AHP1
orf19.2761
471,000
472,000
473,000
474,000
475,000
476,000
477,000
478,000
479,000
480,000
481,000
482,000
483,000
484,000
485,000
486,000
487,000
488,000
489,000
490,000
Ca21chr4_C_albicans_SC5314

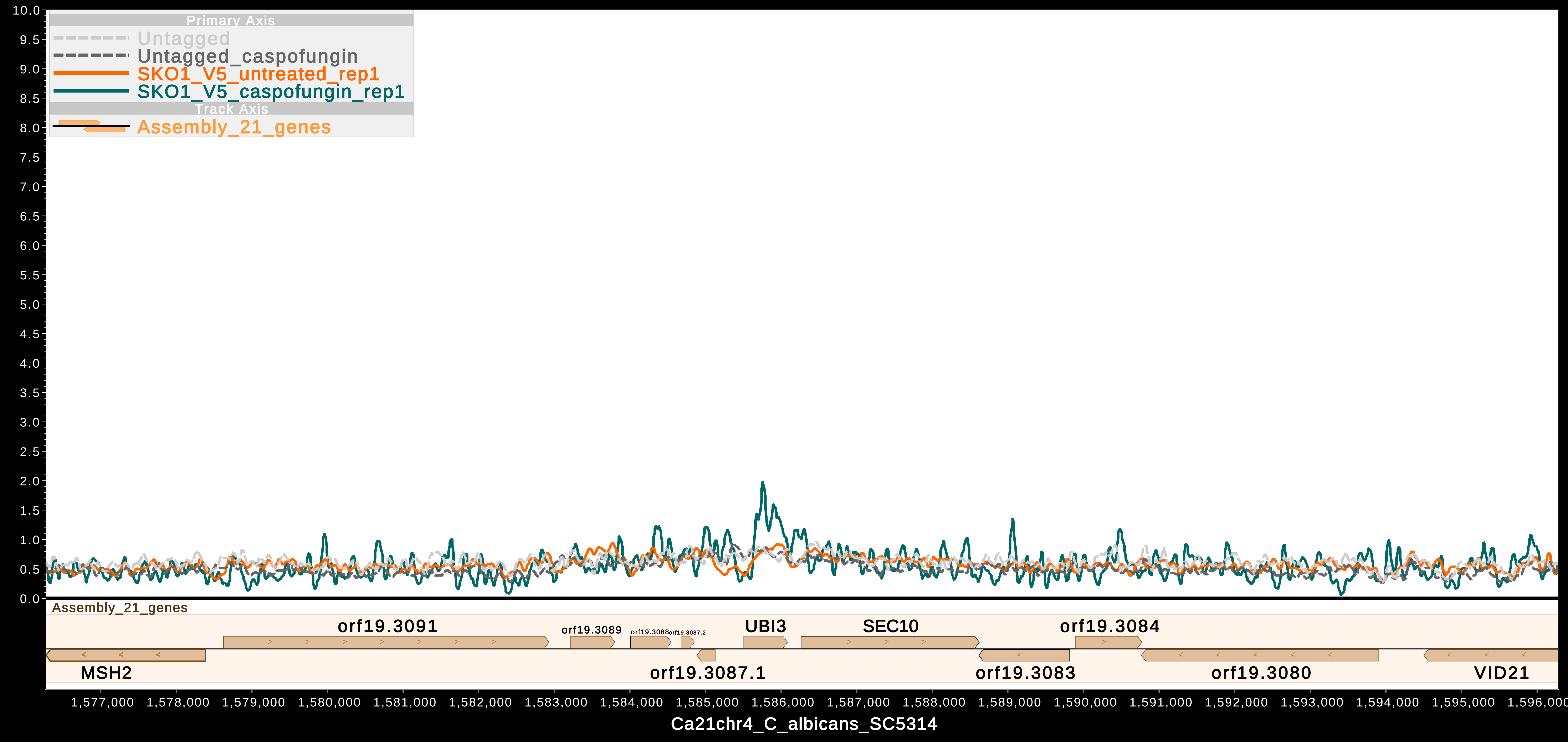

Primary Axis
Untagged
Untagged_caspofungin
SKO1_V5_untreated_rep1
SKO1_V5_caspofungin_rep1
Track Axis
Assembly_21_genes
10.0
9.5
9.0
8.5
8.0
7.5
7.0
6.5
6.0
5.5
5.0
4.5
4.0
3.5
3.0
2.5
2.0
1.5
1.0
0.5
0.0
Assembly_21_genes
orf19.3091
orf19.3089
orf19.3088
orf19.3087.2
UBI3
SEC10
orf19.3084
MSH2
orf19.3087.1
orf19.3083
orf19.3080
VID21
1,577,000
1,578,000
1,579,000
1,580,000
1,581,000
1,582,000
1,583,000
1,584,000
1,585,000
1,586,000
1,587,000
1,588,000
1,589,000
1,590,000
1,591,000
1,592,000
1,593,000
1,594,000
1,595,000
1,596,00
Ca21chr4_C_albicans_SC5314

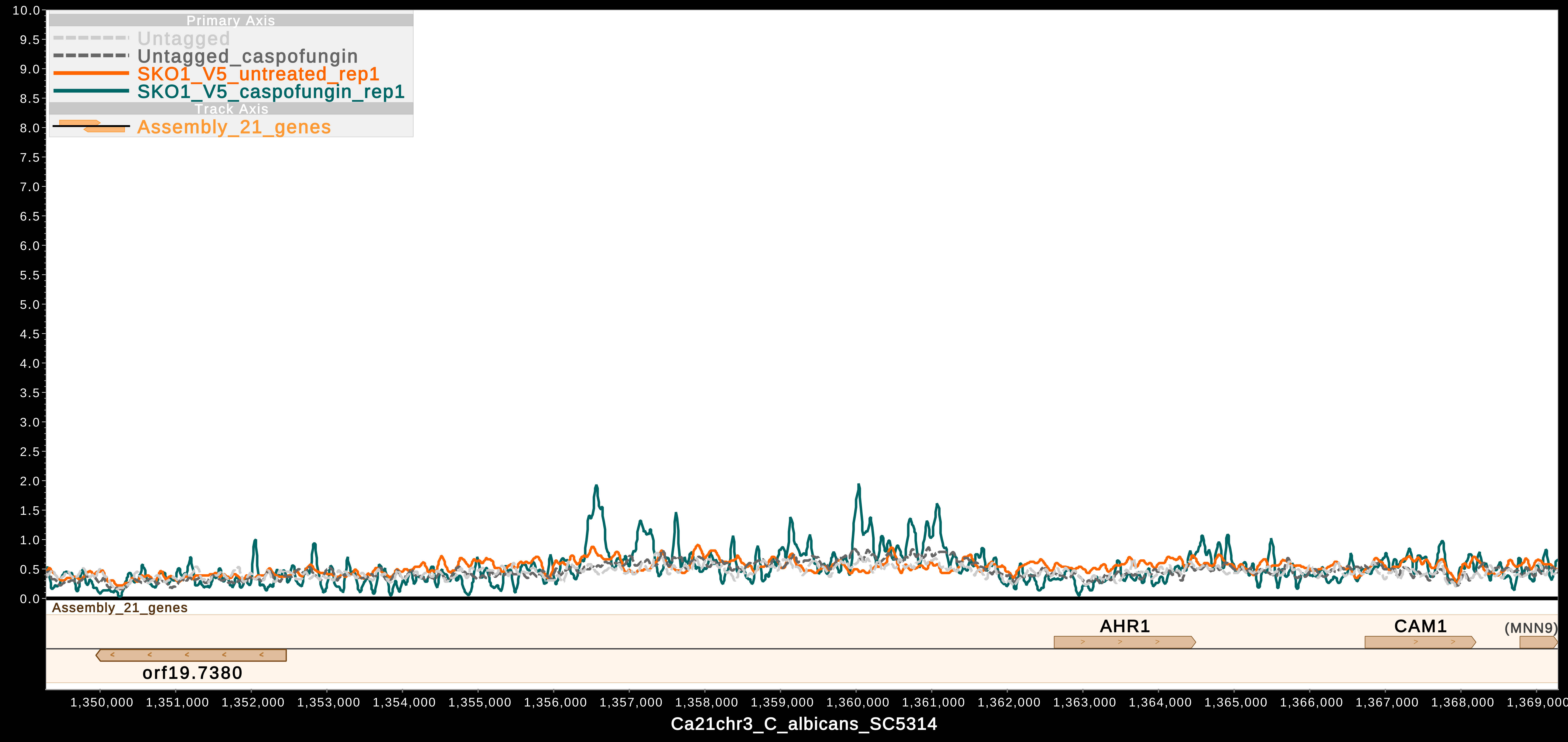

Primary Axis
Untagged
Untagged_caspofungin
SKO1_V5_untreated_rep1
SKO1_V5_caspofungin_rep1
Track Axis
Assembly_21_genes
10.0
9.5
9.0
8.5
8.0
7.5
7.0
6.5
6.0
5.5
5.0
4.5
4.0
3.5
3.0
2.5
2.0
1.5
1.0
0.5
0.0
Assembly_21_genes
AHR1
CAM1
(MNN9)
orf19.7380
1,350,000
1,351,000
1,352,000
1,353,000
1,354,000
1,355,000
1,356,000
1,357,000
1,358,000
1,359,000
1,360,000
1,361,000
1,362,000
1,363,000
1,364,000
1,365,000
1,366,000
1,367,000
1,368,000
1,369,000
Ca21chr3_C_albicans_SC5314

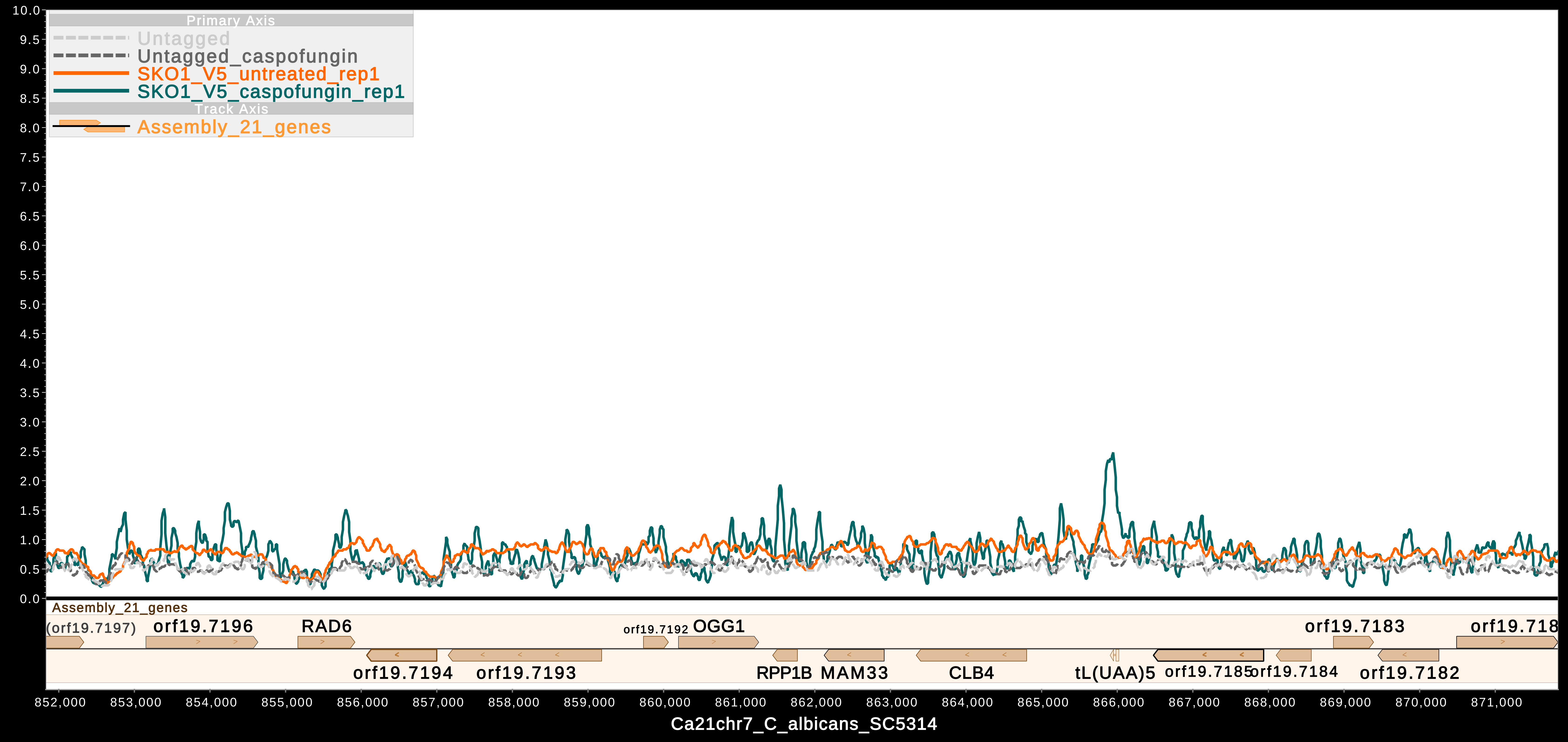

Primary Axis
Untagged
Untagged_caspofungin
SKO1_V5_untreated_rep1
SKO1_V5_caspofungin_rep1
Track Axis
Assembly_21_genes
10.0
9.5
9.0
8.5
8.0
7.5
7.0
6.5
6.0
5.5
5.0
4.5
4.0
3.5
3.0
2.5
2.0
1.5
1.0
0.5
0.0
Assembly_21_genes
(orf19.7197)
orf19.7196
RAD6
orf19.7192
OGG1
orf19.7183
orf19.718
orf19.7194
orf19.7193
RPP1B
MAM33
CLB4
tL(UAA)5
orf19.7185
orf19.7184
orf19.7182
852,000
853,000
854,000
855,000
856,000
857,000
858,000
859,000
860,000
861,000
862,000
863,000
864,000
865,000
866,000
867,000
868,000
869,000
870,000
871,000
Ca21chr7_C_albicans_SC5314

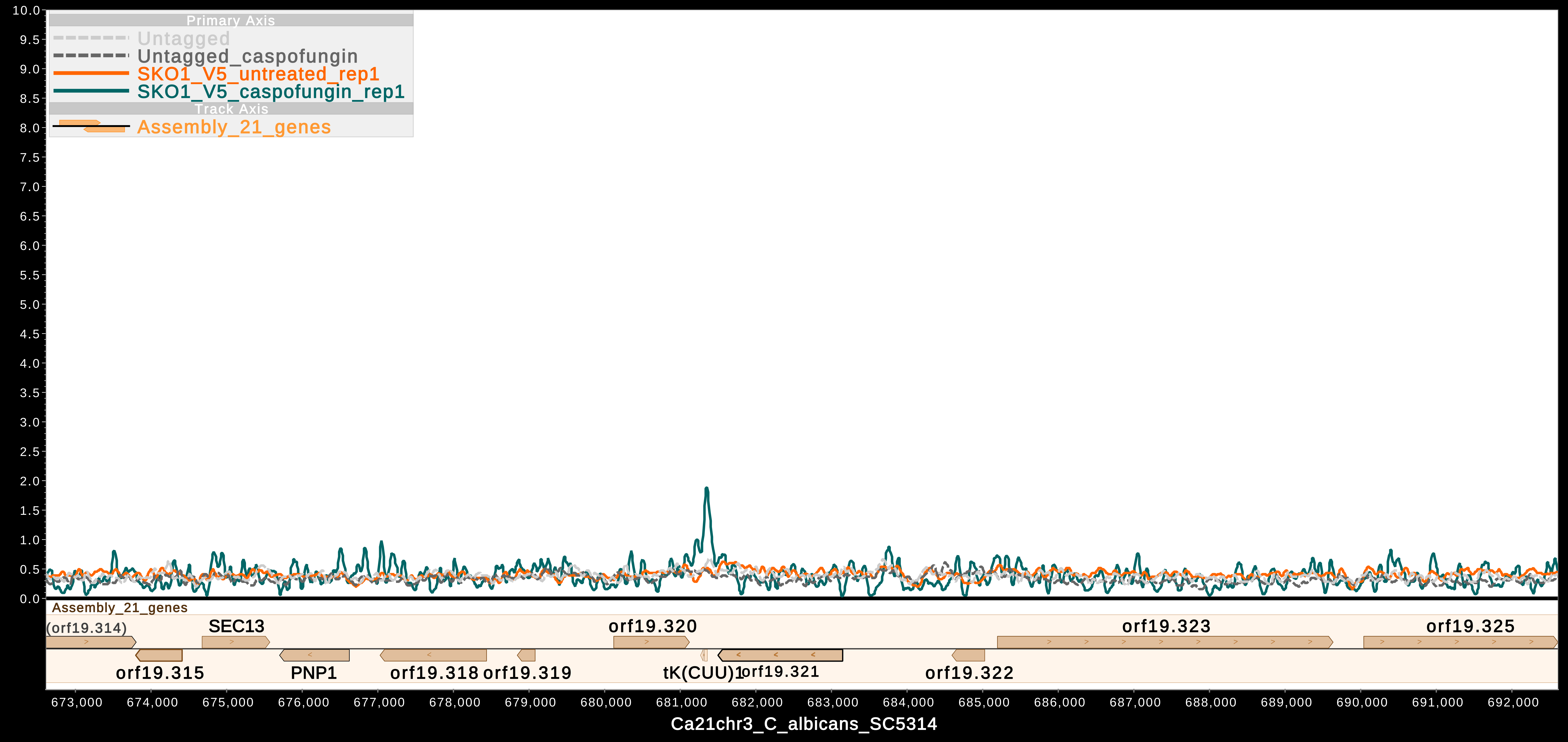

Primary Axis
Untagged
Untagged_caspofungin
SKO1_V5_untreated_rep1
SKO1_V5_caspofungin_rep1
Track Axis
Assembly_21_genes
10.0
9.5
9.0
8.5
8.0
7.5
7.0
6.5
6.0
5.5
5.0
4.5
4.0
3.5
3.0
2.5
2.0
1.5
1.0
0.5
0.0
Assembly_21_genes
(orf19.314)
SEC13
orf19.320
orf19.323
orf19.325
orf19.315
PNP1
orf19.318
orf19.319
tK(CUU)1
orf19.321
orf19.322
673,000
674,000
675,000
676,000
677,000
678,000
679,000
680,000
681,000
682,000
683,000
684,000
685,000
686,000
687,000
688,000
689,000
690,000
691,000
692,000
Ca21chr3_C_albicans_SC5314

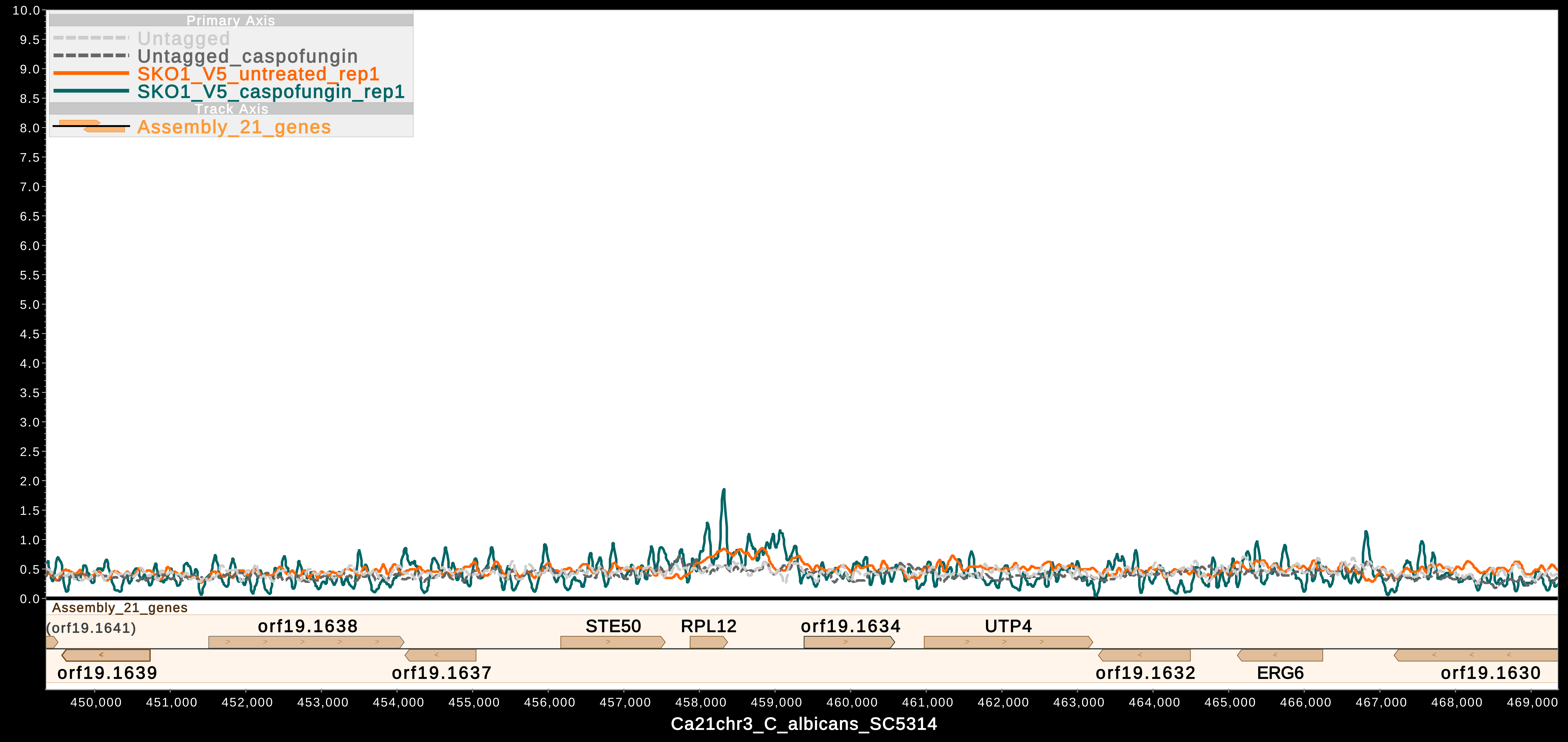

Primary Axis
Untagged
Untagged_caspofungin
SKO1_V5_untreated_rep1
SKO1_V5_caspofungin_rep1
Track Axis
Assembly_21_genes
10.0
9.5
9.0
8.5
8.0
7.5
7.0
6.5
6.0
5.5
5.0
4.5
4.0
3.5
3.0
2.5
2.0
1.5
1.0
0.5
0.0
Assembly_21_genes
(orf19.1641)
orf19.1638
STE50
RPL12
orf19.1634
UTP4
orf19.1639
orf19.1637
orf19.1632
ERG6
orf19.1630
450,000
451,000
452,000
453,000
454,000
455,000
456,000
457,000
458,000
459,000
460,000
461,000
462,000
463,000
464,000
465,000
466,000
467,000
468,000
469,000
Ca21chr3_C_albicans_SC5314

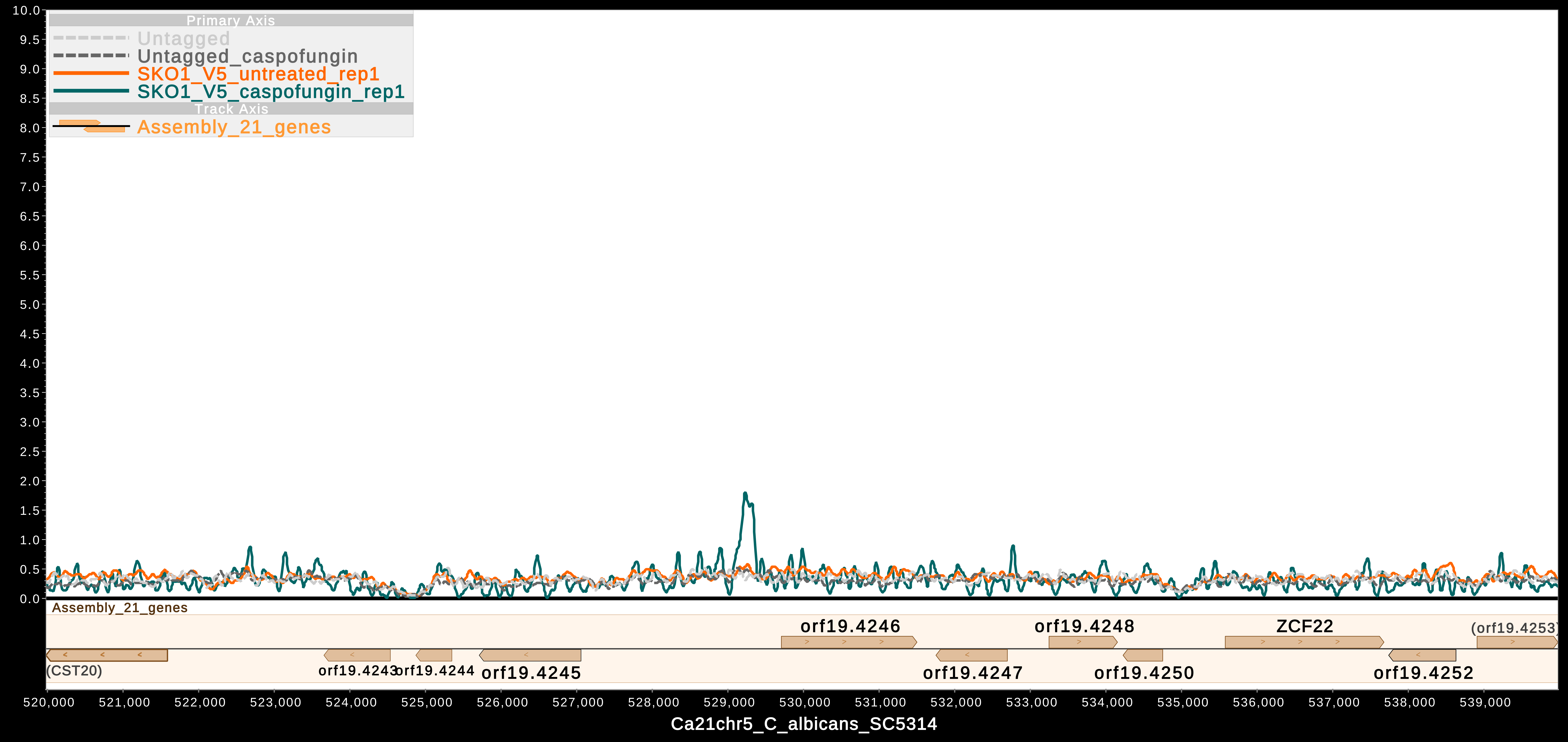

Primary Axis
Untagged
Untagged_caspofungin
SKO1_V5_untreated_rep1
SKO1_V5_caspofungin_rep1
Track Axis
Assembly_21_genes
10.0
9.5
9.0
8.5
8.0
7.5
7.0
6.5
6.0
5.5
5.0
4.5
4.0
3.5
3.0
2.5
2.0
1.5
1.0
0.5
0.0
Assembly_21_genes
orf19.4246
orf19.4248
ZCF22
(orf19.4253)
(CST20)
orf19.4243
orf19.4244
orf19.4245
orf19.4247
orf19.4250
orf19.4252
520,000
521,000
522,000
523,000
524,000
525,000
526,000
527,000
528,000
529,000
530,000
531,000
532,000
533,000
534,000
535,000
536,000
537,000
538,000
539,000
Ca21chr5_C_albicans_SC5314

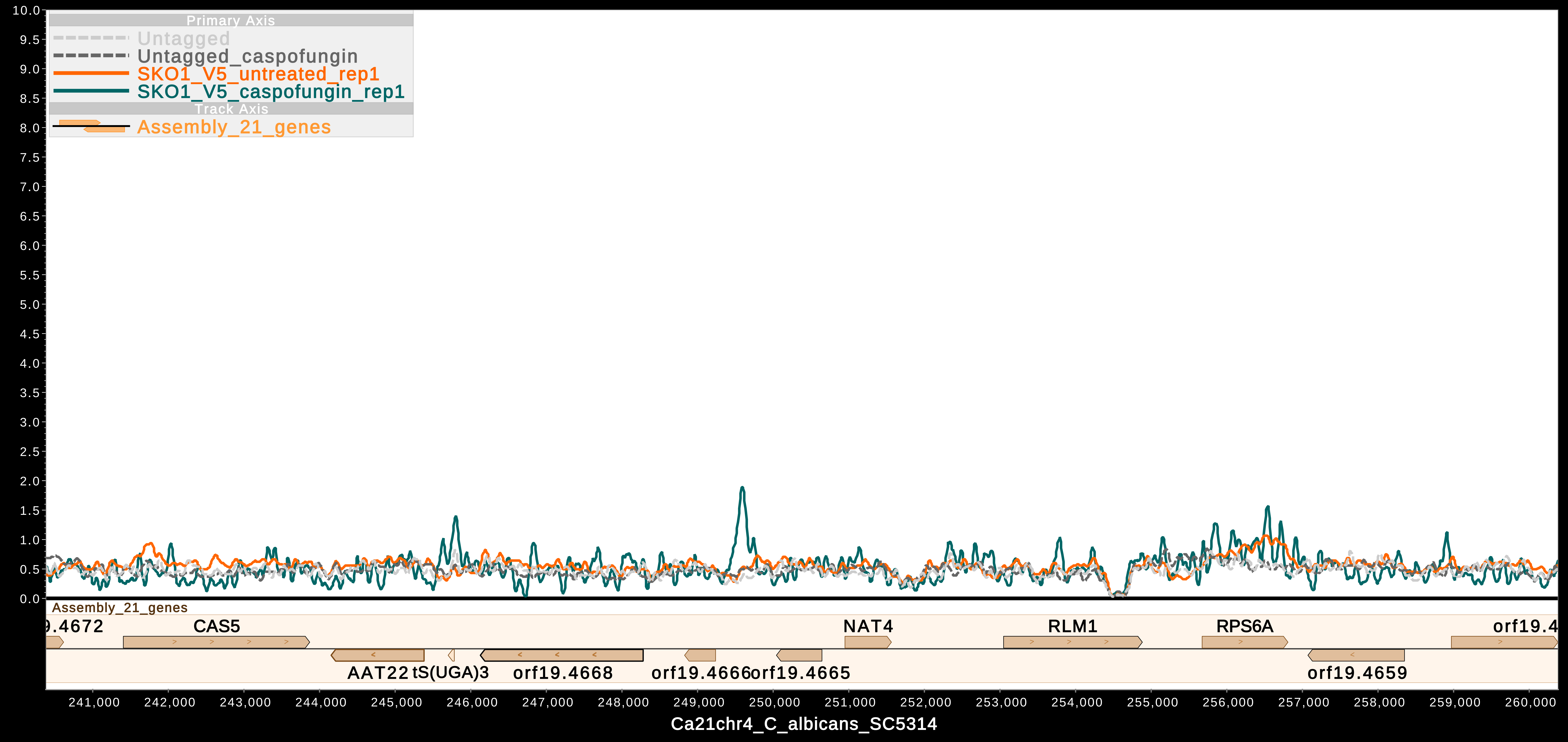
Primary Axis
Untagged
Untagged_caspofungin
SKO1_V5_untreated_rep1
SKO1_V5_caspofungin_rep1
Track Axis
Assembly_21_genes
10.0
9.5
9.0
8.5
8.0
7.5
7.0
6.5
6.0
5.5
5.0
4.5
4.0
3.5
3.0
2.5
2.0
1.5
1.0
0.5
0.0
Assembly_21_genes
.4672
CAS5
NAT4
RLM1
RPS6A
orf19.4
AAT22
tS(UGA)3
orf19.4668
orf19.4666
orf19.4665
orf19.4659
241,000
242,000
243,000
244,000
245,000
246,000
247,000
248,000
249,000
250,000
251,000
252,000
253,000
254,000
255,000
256,000
257,000
258,000
259,000
260,000
Ca21chr4_C_albicans_SC5314

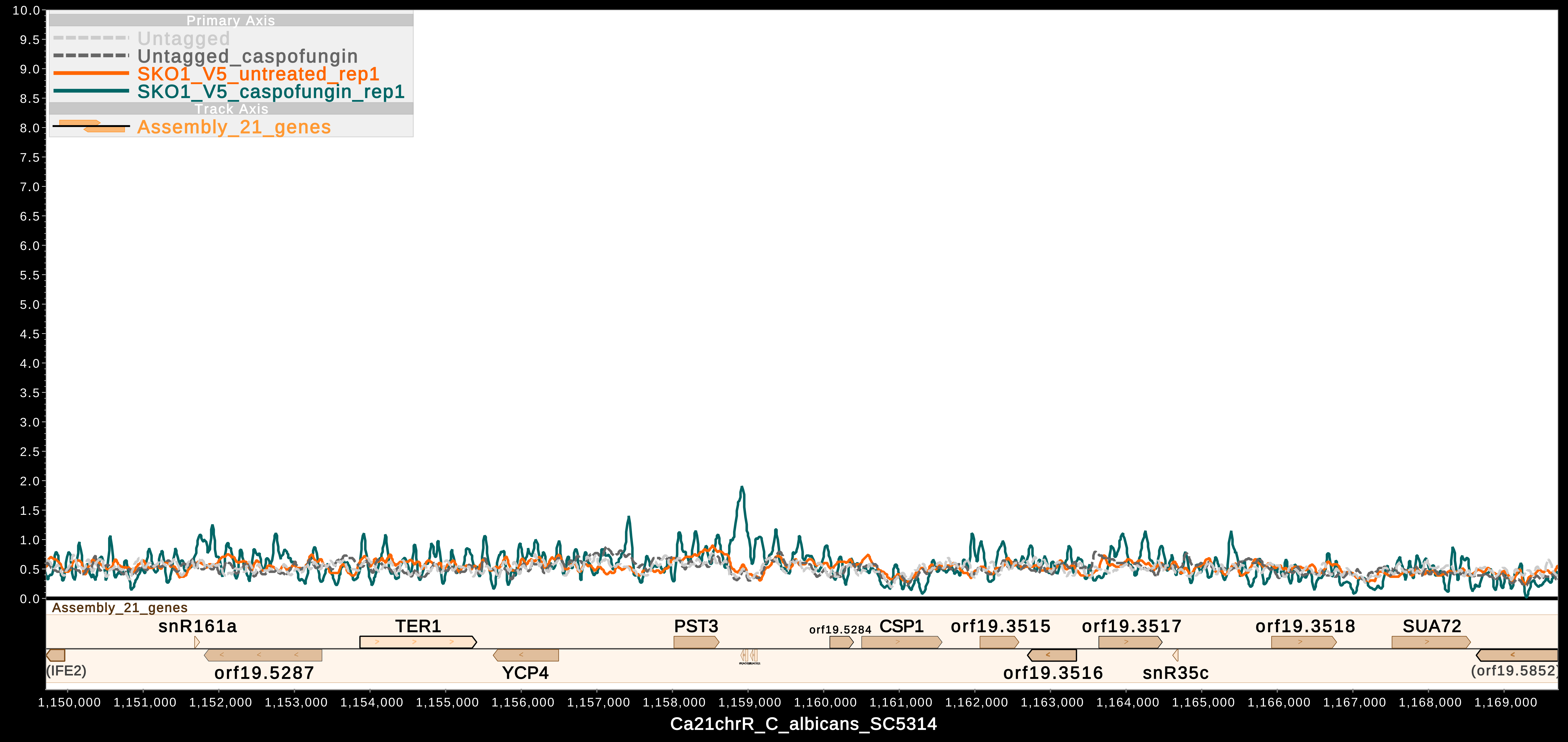
Primary Axis
Untagged
Untagged_caspofungin
SKO1_V5_untreated_rep1
SKO1_V5_caspofungin_rep1
Track Axis
Assembly_21_genes
10.0
9.5
9.0
8.5
8.0
7.5
7.0
6.5
6.0
5.5
5.0
4.5
4.0
3.5
3.0
2.5
2.0
1.5
1.0
0.5
0.0
Assembly_21_genes
snR161a
TER1
PST3
orf19.5284
CSP1
orf19.3515
orf19.3517
orf19.3518
SUA72
(IFE2)
orf19.5287
YCP4
orf19.3516
snR35c
(orf19.5852)
1,150,000
1,151,000
1,152,000
1,153,000
1,154,000
1,155,000
1,156,000
1,157,000
1,158,000
1,159,000
1,160,000
1,161,000
1,162,000
1,163,000
1,164,000
1,165,000
1,166,000
1,167,000
1,168,000
1,169,000
Ca21chrR_C_albicans_SC5314

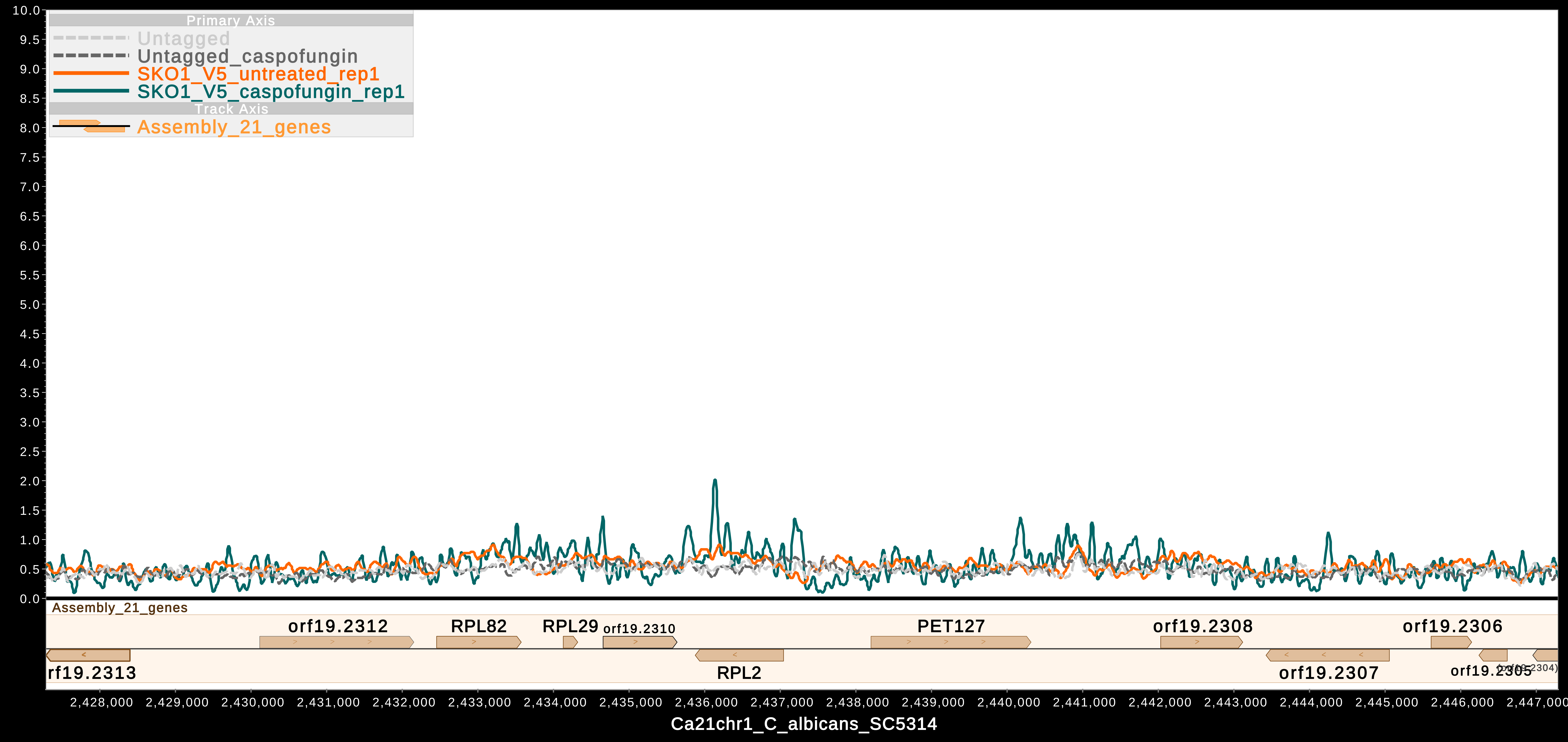

Primary Axis
Untagged
Untagged_caspofungin
SKO1_V5_untreated_rep1
SKO1_V5_caspofungin_rep1
Track Axis
Assembly_21_genes
10.0
9.5
9.0
8.5
8.0
7.5
7.0
6.5
6.0
5.5
5.0
4.5
4.0
3.5
3.0
2.5
2.0
1.5
1.0
0.5
0.0
Assembly_21_genes
orf19.2312
RPL82
RPL29
orf19.2310
PET127
orf19.2308
orf19.2306
rf19.2313
RPL2
orf19.2307
orf19.2305
2,428,000
2,429,000
2,430,000
2,431,000
2,432,000
2,433,000
2,434,000
2,435,000
2,436,000
2,437,000
2,438,000
2,439,000
2,440,000
2,441,000
2,442,000
2,443,000
2,444,000
2,445,000
2,446,000
2,447,000
Ca21chr1_C_albicans_SC5314

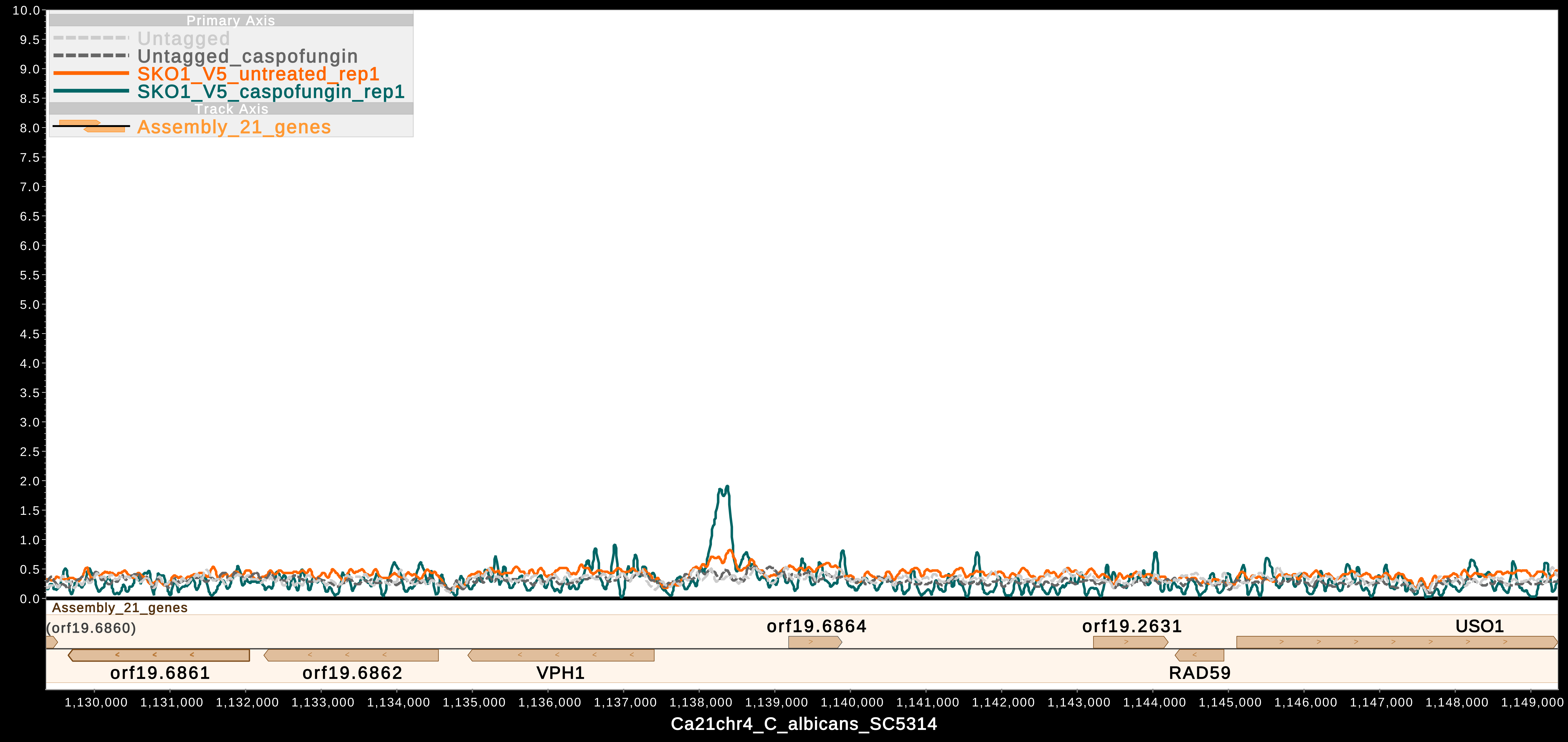

Primary Axis
Untagged
Untagged_caspofungin
SKO1_V5_untreated_rep1
SKO1_V5_caspofungin_rep1
Track Axis
Assembly_21_genes
10.0
9.5
9.0
8.5
8.0
7.5
7.0
6.5
6.0
5.5
5.0
4.5
4.0
3.5
3.0
2.5
2.0
1.5
1.0
0.5
0.0
Assembly_21_genes
(orf19.6860)
orf19.6864
orf19.2631
USO1
orf19.6861
orf19.6862
VPH1
RAD59
1,130,000
1,131,000
1,132,000
1,133,000
1,134,000
1,135,000
1,136,000
1,137,000
1,138,000
1,139,000
1,140,000
1,141,000
1,142,000
1,143,000
1,144,000
1,145,000
1,146,000
1,147,000
1,148,000
1,149,000
Ca21chr4_C_albicans_SC5314

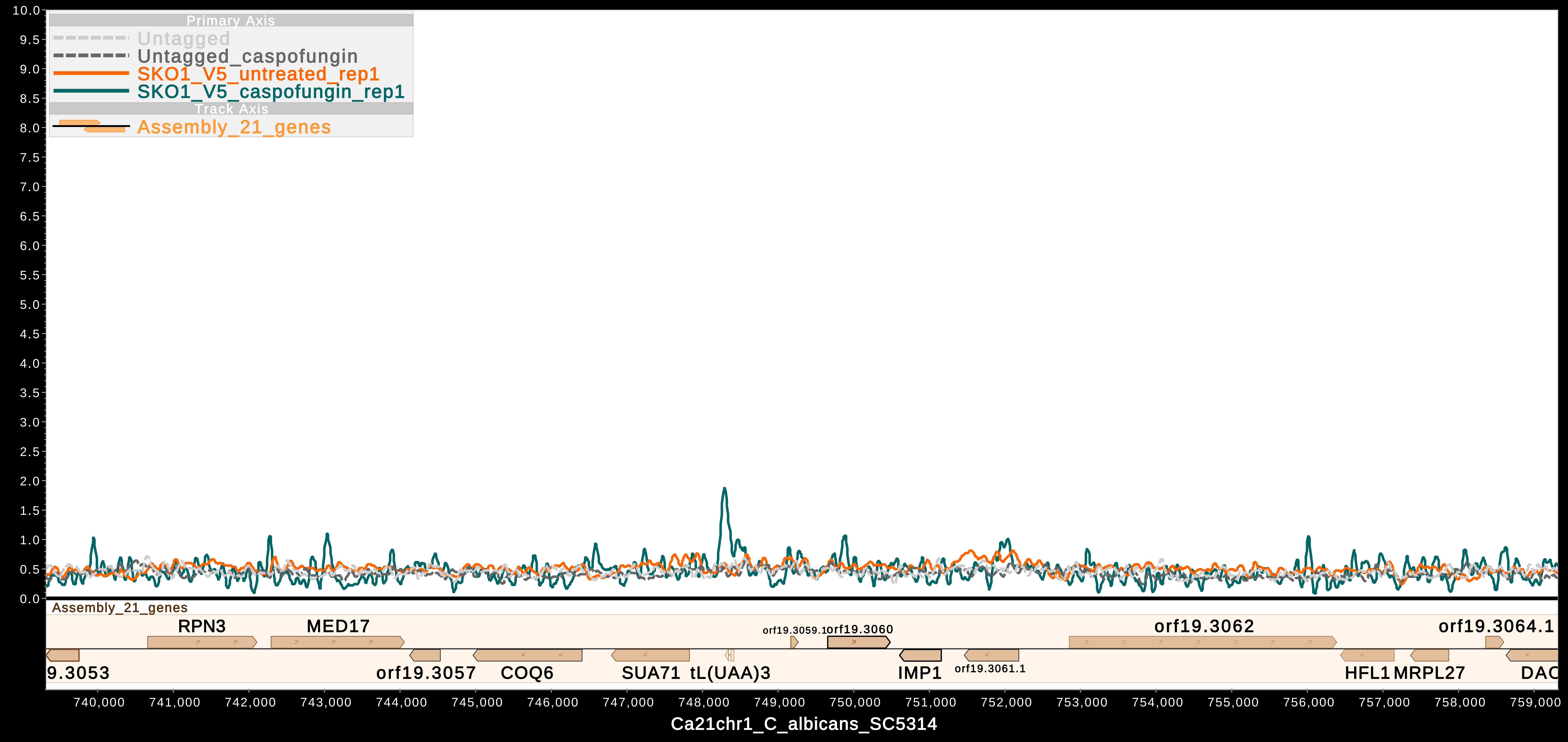

Primary Axis
Untagged
Untagged_caspofungin
SKO1_V5_untreated_rep1
SKO1_V5_caspofungin_rep1
Track Axis
Assembly_21_genes
10.0
9.5
9.0
8.5
8.0
7.5
7.0
6.5
6.0
5.5
5.0
4.5
4.0
3.5
3.0
2.5
2.0
1.5
1.0
0.5
0.0
Assembly_21_genes
RPN3
MED17
orf19.3059.1
orf19.3060
orf19.3062
orf19.3064.1
9.3053
orf19.3057
COQ6
SUA71
tL(UAA)3
IMP1
orf19.3061.1
HFL1
MRPL27
DAC
740,000
741,000
742,000
743,000
744,000
745,000
746,000
747,000
748,000
749,000
750,000
751,000
752,000
753,000
754,000
755,000
756,000
757,000
758,000
759,000
Ca21chr1_C_albicans_SC5314

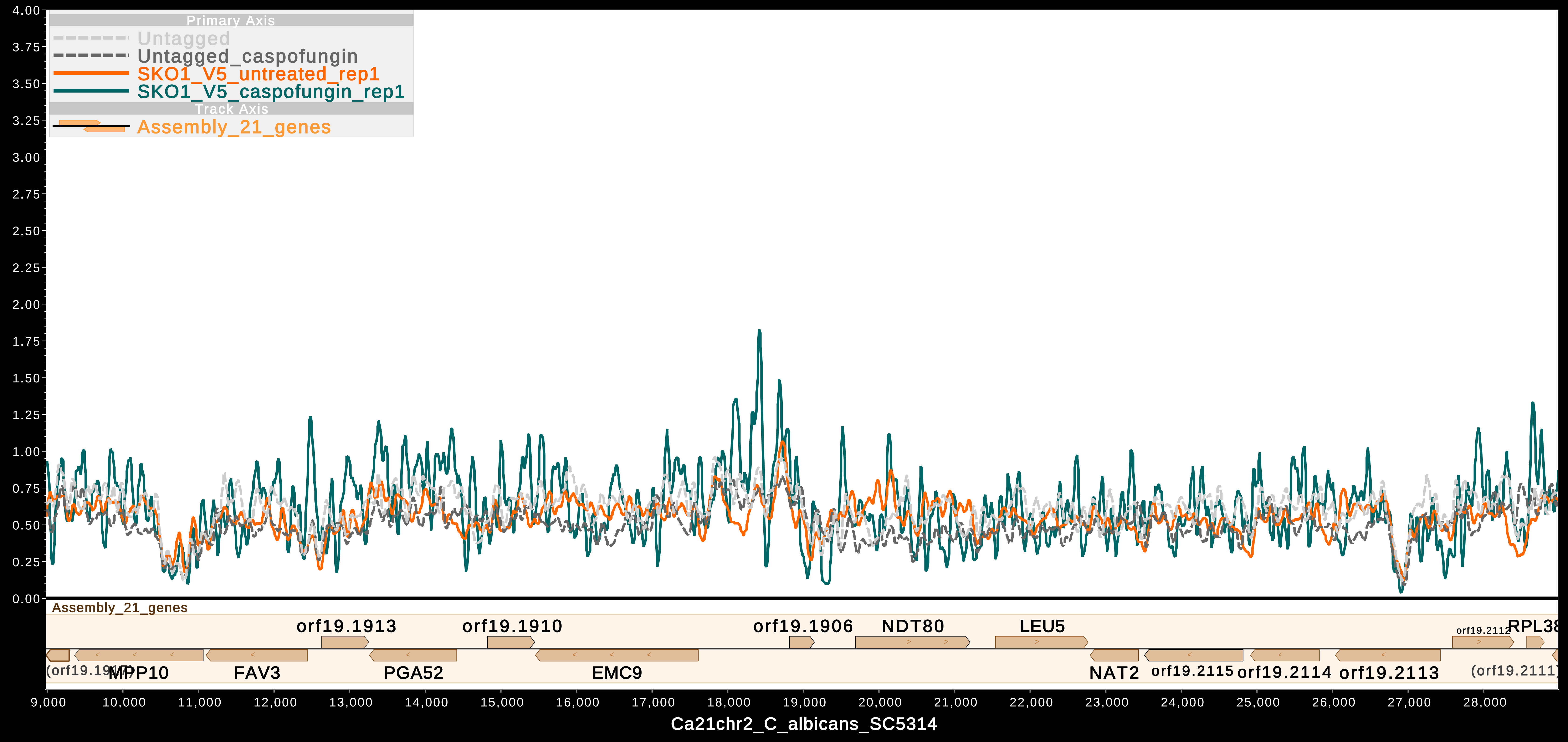

Primary Axis
Untagged
Untagged_caspofungin
SKO1_V5_untreated_rep1
SKO1_V5_caspofungin_rep1
Track Axis
Assembly_21_genes
4.00
3.75
3.50
3.25
3.00
2.75
2.50
2.25
2.00
1.75
1.50
1.25
1.00
0.75
0.50
0.25
0.00
Assembly_21_genes
orf19.1913
orf19.1910
orf19.1906
NDT80
LEU5
orf19.2112
RPL38
MPP10
FAV3
PGA52
EMC9
NAT2
orf19.2115
orf19.2114
orf19.2113
(orf19.2111)
9,000
10,000
11,000
12,000
13,000
14,000
15,000
16,000
17,000
18,000
19,000
20,000
21,000
22,000
23,000
24,000
25,000
26,000
27,000
28,000
Ca21chr2_C_albicans_SC5314

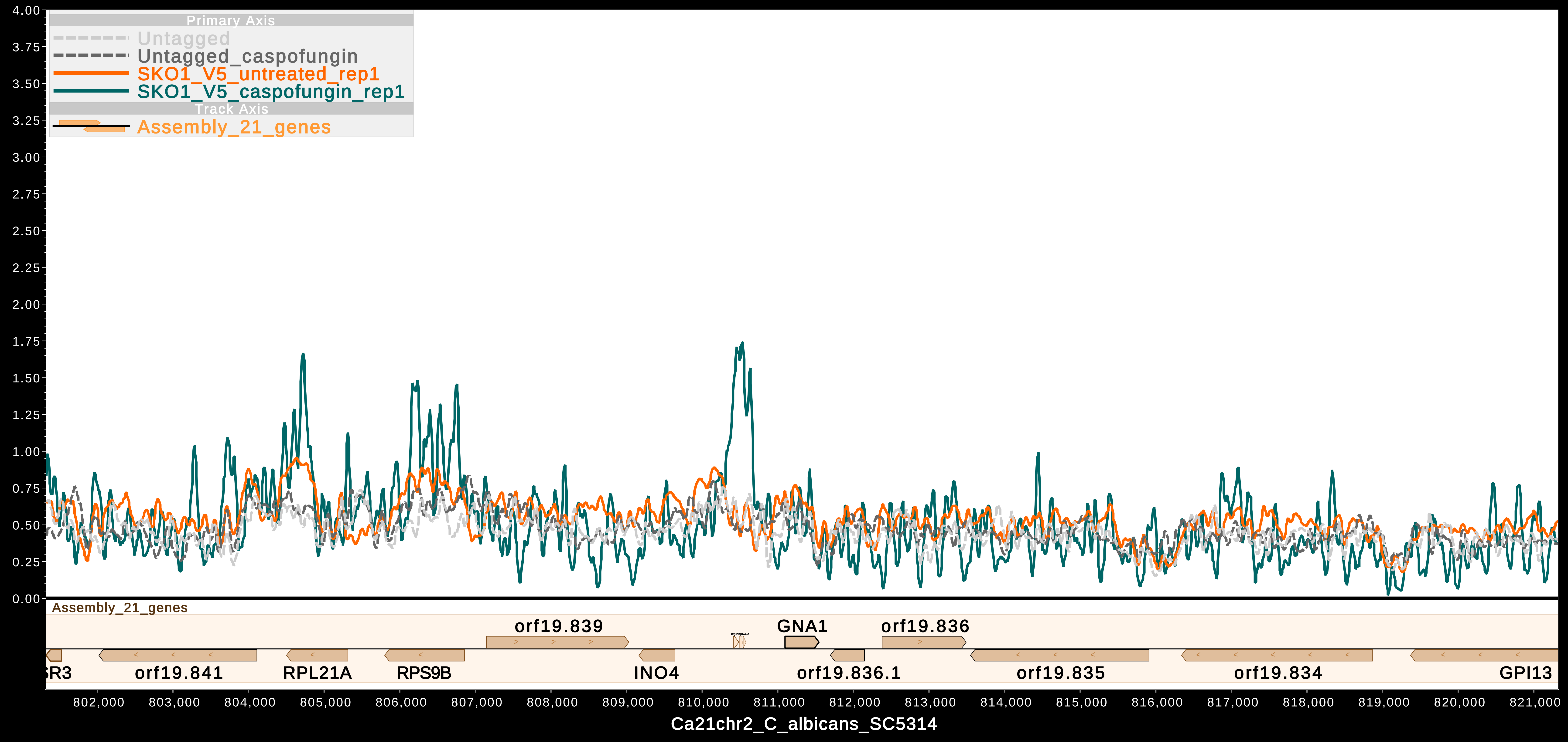

Primary Axis
Untagged
Untagged_caspofungin
SKO1_V5_untreated_rep1
SKO1_V5_caspofungin_rep1
Track Axis
Assembly_21_genes
4.00
3.75
3.50
3.25
3.00
2.75
2.50
2.25
2.00
1.75
1.50
1.25
1.00
0.75
0.50
0.25
0.00
Assembly_21_genes
orf19.839
GNA1
orf19.836
R3
orf19.841
RPL21A
RPS9B
INO4
orf19.836.1
orf19.835
orf19.834
GPI13
802,000
803,000
804,000
805,000
806,000
807,000
808,000
809,000
810,000
811,000
812,000
813,000
814,000
815,000
816,000
817,000
818,000
819,000
820,000
821,000
Ca21chr2_C_albicans_SC5314

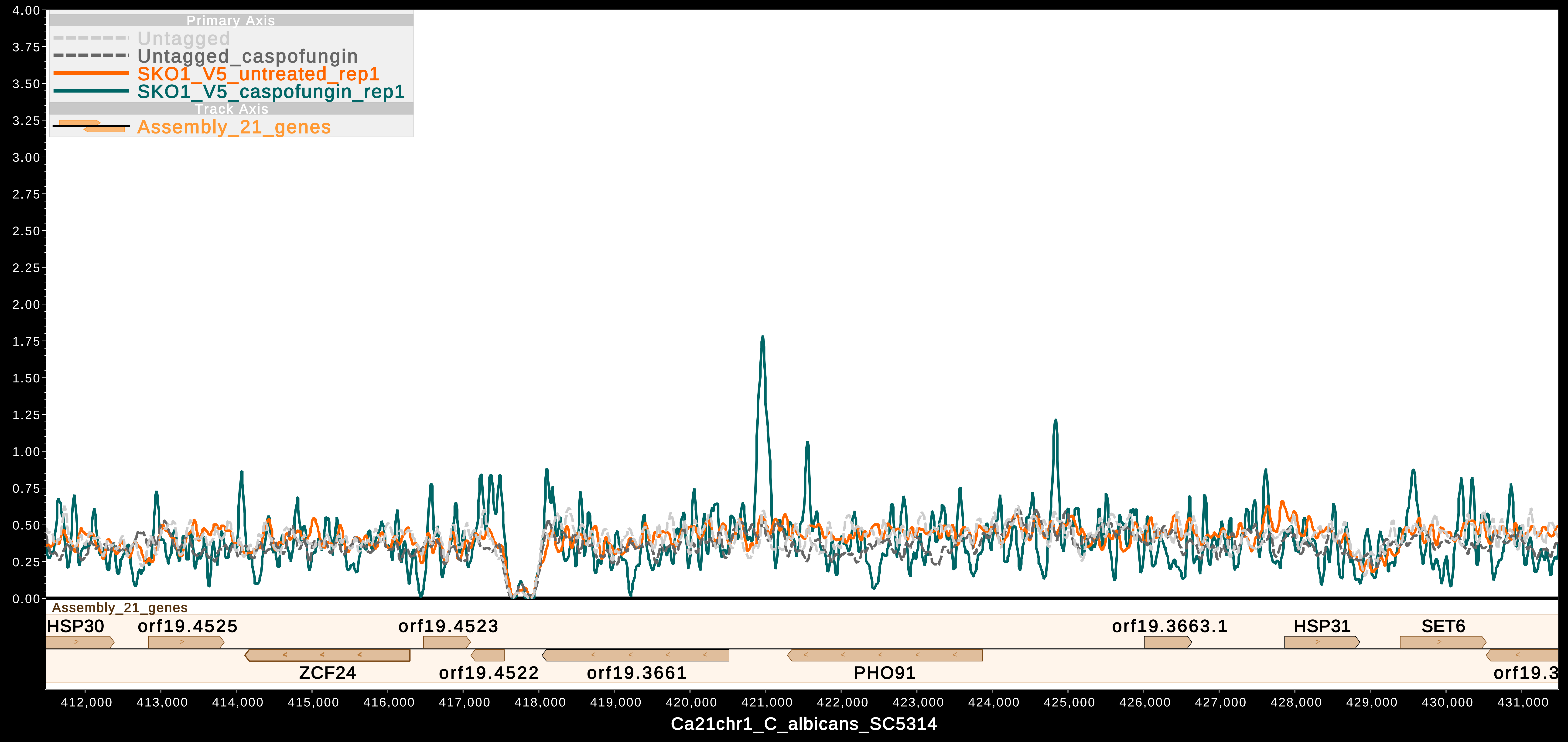

Primary Axis
Untagged
Untagged_caspofungin
SKO1_V5_untreated_rep1
SKO1_V5_caspofungin_rep1
Track Axis
Assembly_21_genes
4.00
3.75
3.50
3.25
3.00
2.75
2.50
2.25
2.00
1.75
1.50
1.25
1.00
0.75
0.50
0.25
0.00
Assembly_21_genes
HSP30
orf19.4525
orf19.4523
orf19.3663.1
HSP31
SET6
ZCF24
orf19.4522
orf19.3661
PHO91
orf19.3
412,000
413,000
414,000
415,000
416,000
417,000
418,000
419,000
420,000
421,000
422,000
423,000
424,000
425,000
426,000
427,000
428,000
429,000
430,000
431,000
Ca21chr1_C_albicans_SC5314

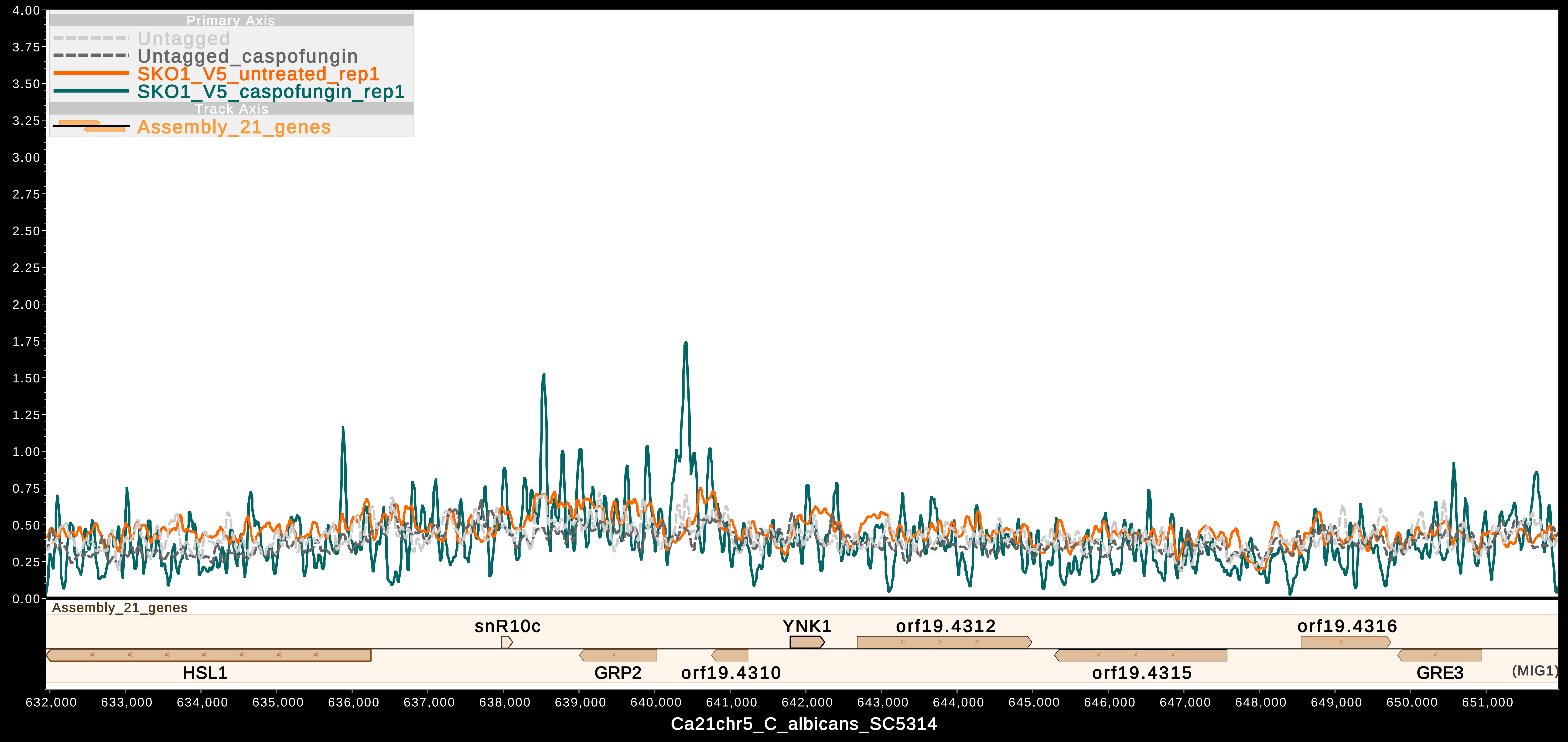

Primary Axis
Untagged
Untagged_caspofungin
SKO1_V5_untreated_rep1
SKO1_V5_caspofungin_rep1
Track Axis
Assembly_21_genes
4.00
3.75
3.50
3.25
3.00
2.75
2.50
2.25
2.00
1.75
1.50
1.25
1.00
0.75
0.50
0.25
0.00
Assembly_21_genes
snR10c
YNK1
orf19.4312
orf19.4316
HSL1
GRP2
orf19.4310
orf19.4315
GRE3
(MIG1)
632,000
633,000
634,000
635,000
636,000
637,000
638,000
639,000
640,000
641,000
642,000
643,000
644,000
645,000
646,000
647,000
648,000
649,000
650,000
651,000
Ca21chr5_C_albicans_SC5314

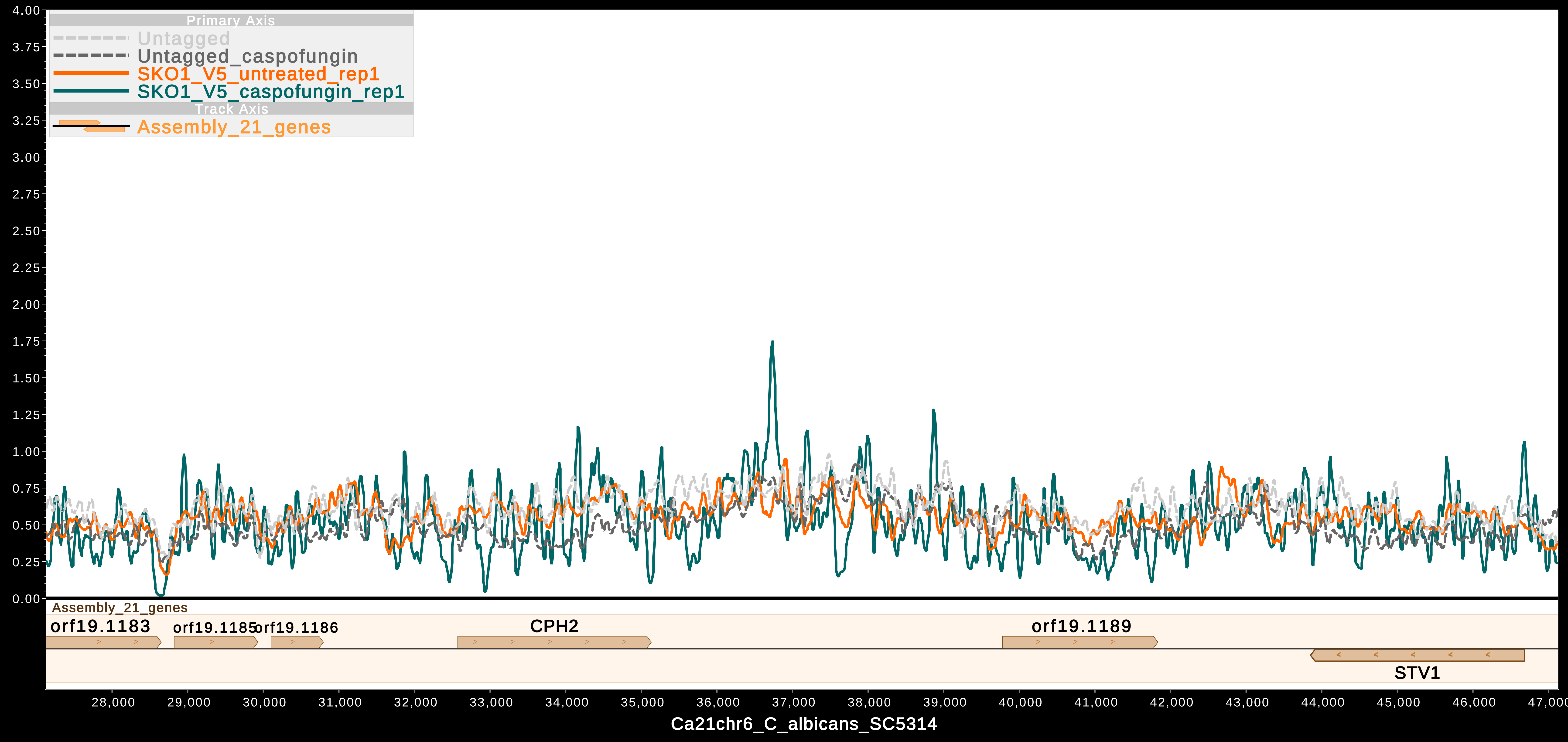

Primary Axis
Untagged
Untagged_caspofungin
SKO1_V5_untreated_rep1
SKO1_V5_caspofungin_rep1
Track Axis
Assembly_21_genes
4.00
3.75
3.50
3.25
3.00
2.75
2.50
2.25
2.00
1.75
1.50
1.25
1.00
0.75
0.50
0.25
0.00
Assembly_21_genes
orf19.1183
orf19.1185
orf19.1186
CPH2
orf19.1189
STV1
28,000
29,000
30,000
31,000
32,000
33,000
34,000
35,000
36,000
37,000
38,000
39,000
40,000
41,000
42,000
43,000
44,000
45,000
46,000
Ca21chr6_C_albicans_SC5314

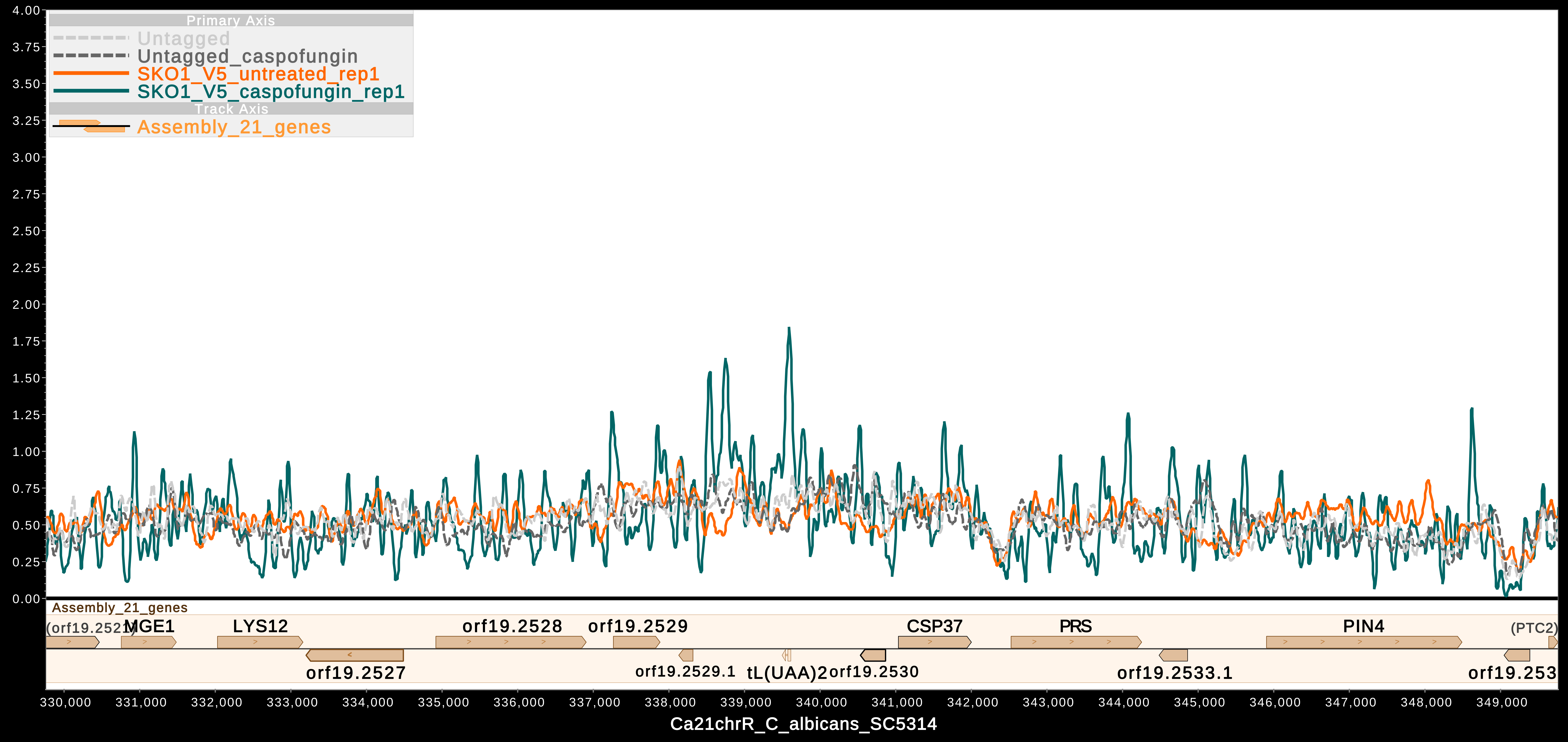

4.00
3.75
3.50
3.25
3.00
2.75
2.50
2.25
2.00
1.75
1.50
1.25
1.00
0.75
0.50
0.25
0.00
Primary Axis
Untagged
Untagged_caspofungin
SKO1_V5_untreated_rep1
SKO1_V5_caspofungin_rep1
Track Axis
Assembly_21_genes
Assembly_21_genes
(orf19.2521)
MGE1
LYS12
orf19.2528
orf19.2529
CSP37
PRS
PIN4
(PTC2)
orf19.2527
orf19.2529.1
tL(UAA)2
orf19.2530
orf19.2533.1
orf19.253
330,000
331,000
332,000
333,000
334,000
335,000
336,000
337,000
338,000
339,000
340,000
341,000
342,000
343,000
344,000
345,000
346,000
347,000
348,000
349,000
Ca21chrR_C_albicans_SC5314

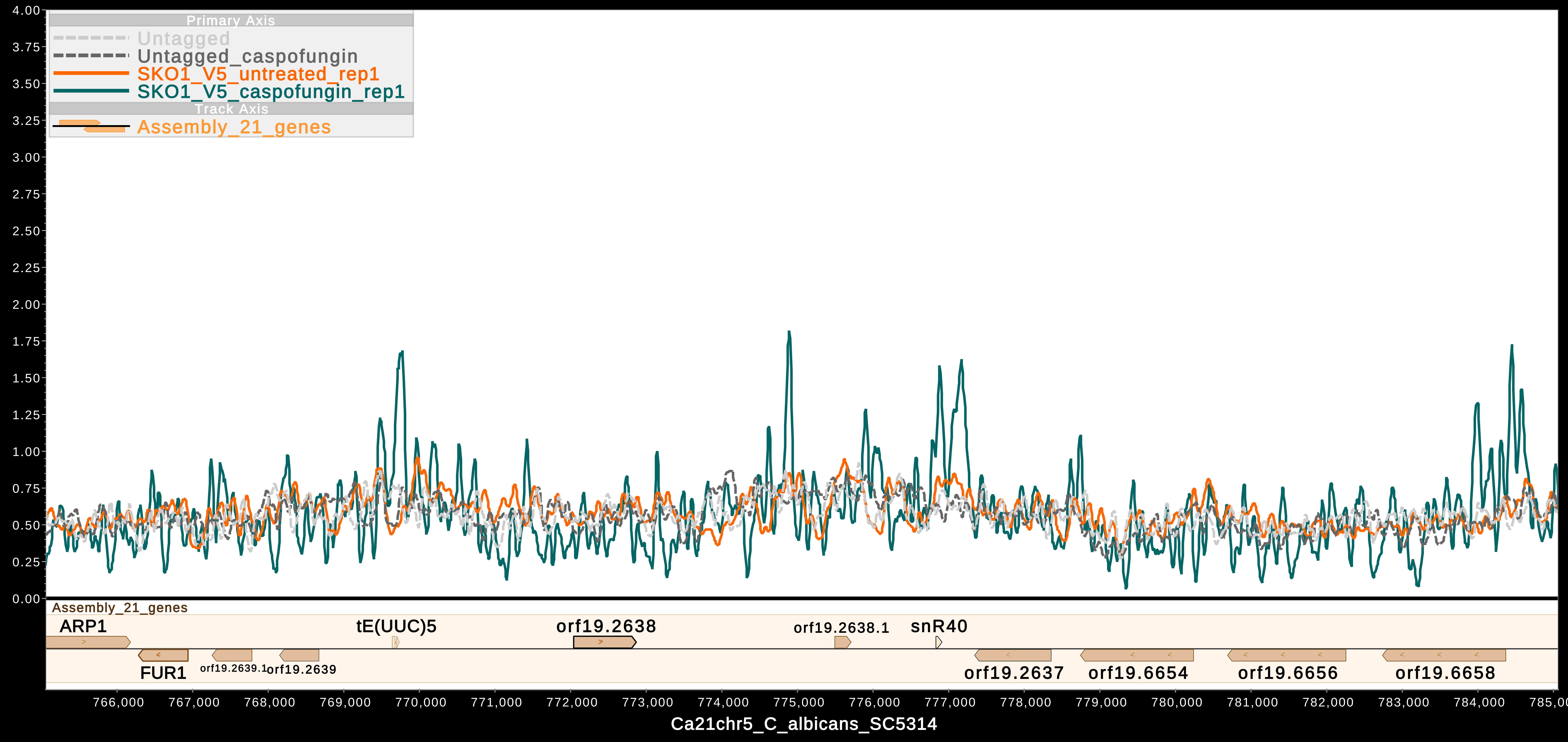

Primary Axis
Untagged
Untagged_caspofungin
SKO1_V5_untreated_rep1
SKO1_V5_caspofungin_rep1
Track Axis
Assembly_21_genes
4.00
3.75
3.50
3.25
3.00
2.75
2.50
2.25
2.00
1.75
1.50
1.25
1.00
0.75
0.50
0.25
0.00
Assembly_21_genes
ARP1
tE(UUC)5
orf19.2638
orf19.2638.1
snR40
FUR1
orf19.2639.1
orf19.2639
orf19.2637
orf19.6654
orf19.6656
orf19.6658
766,000
767,000
768,000
769,000
770,000
771,000
772,000
773,000
774,000
775,000
776,000
777,000
778,000
779,000
780,000
781,000
782,000
783,000
784,000
Ca21chr5_C_albicans_SC5314

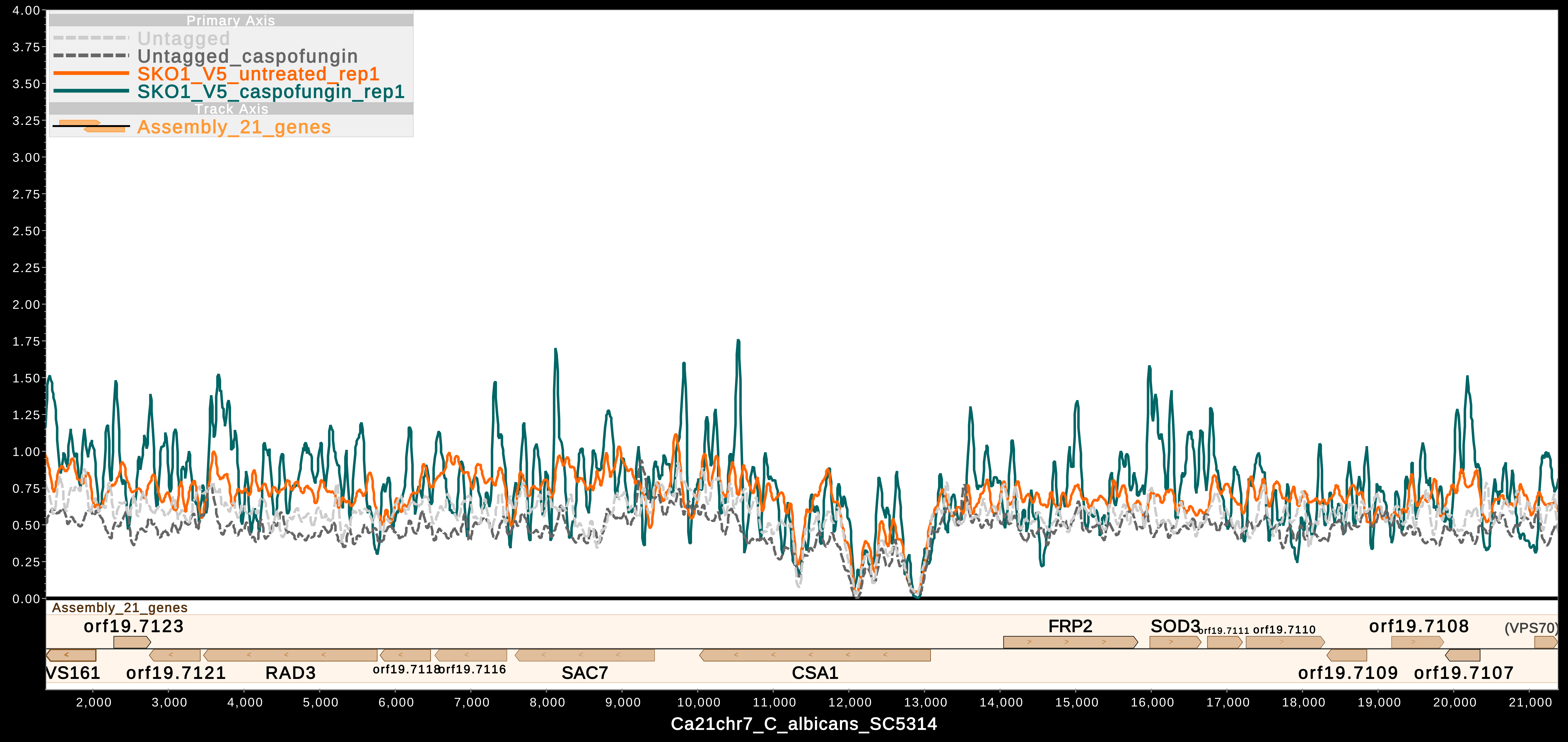
Primary Axis
Untagged
Untagged_caspofungin
SKO1_V5_untreated_rep1
SKO1_V5_caspofungin_rep1
Track Axis
Assembly_21_genes
4.00
3.75
3.50
3.25
3.00
2.75
2.50
2.25
2.00
1.75
1.50
1.25
1.00
0.75
0.50
0.25
0.00
Assembly_21_genes
orf19.7123
FRP2
SOD3
orf19.7111
orf19.7110
orf19.7108
(VPS70)
VS161
orf19.7121
RAD3
orf19.7118
orf19.7116
SAC7
CSA1
orf19.7109
orf19.7107
2,000
3,000
4,000
5,000
6,000
7,000
8,000
9,000
10,000
11,000
12,000
13,000
14,000
15,000
16,000
17,000
18,000
19,000
20,000
21,000
Ca21chr7_C_albicans_SC5314

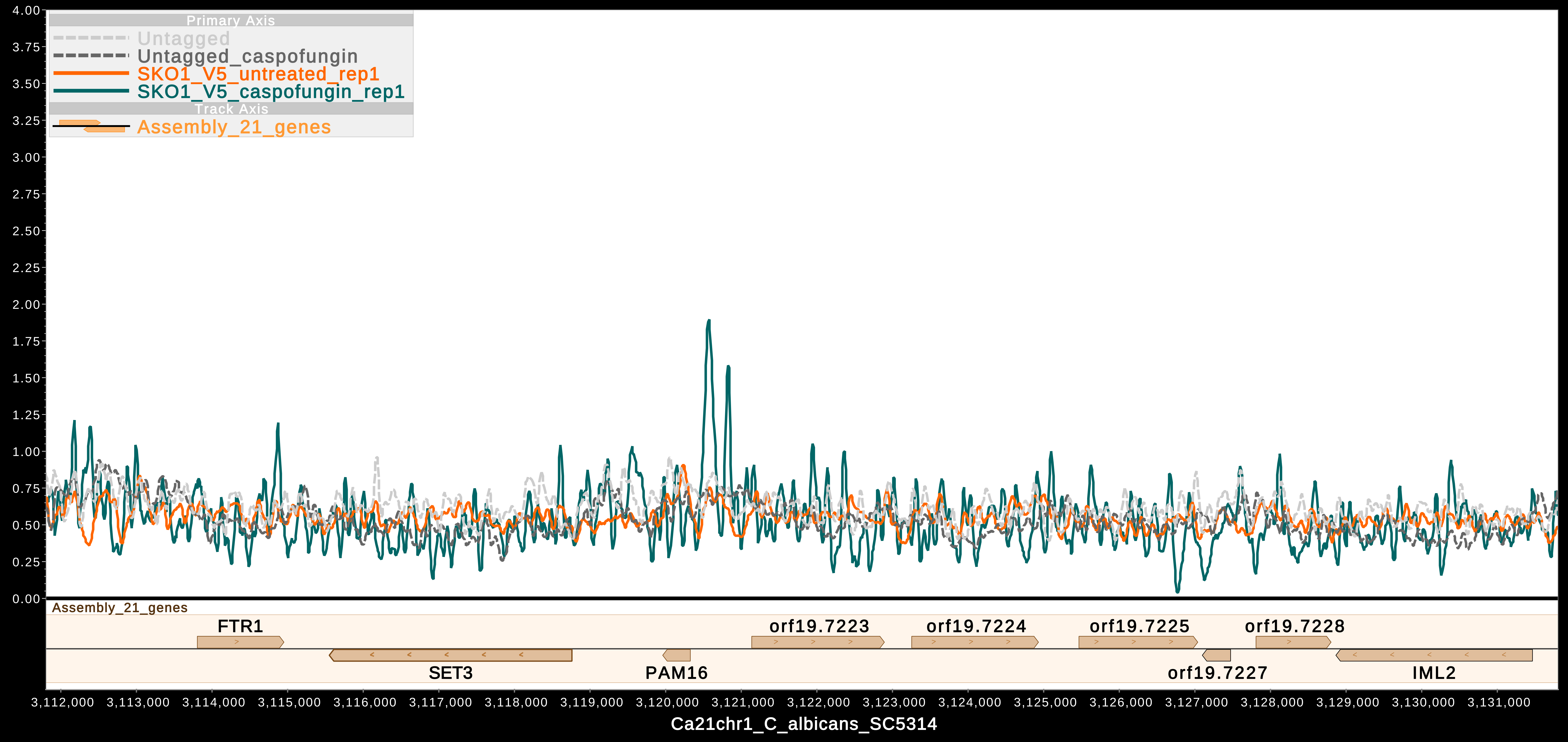

Primary Axis
Untagged
Untagged_caspofungin
SKO1_V5_untreated_rep1
SKO1_V5_caspofungin_rep1
Track Axis
Assembly_21_genes
4.00
3.75
3.50
3.25
3.00
2.75
2.50
2.25
2.00
1.75
1.50
1.25
1.00
0.75
0.50
0.25
0.00
Assembly_21_genes
FTR1
orf19.7223
orf19.7224
orf19.7225
orf19.7228
SET3
PAM16
orf19.7227
IML2
3,112,000
3,113,000
3,114,000
3,115,000
3,116,000
3,117,000
3,118,000
3,119,000
3,120,000
3,121,000
3,122,000
3,123,000
3,124,000
3,125,000
3,126,000
3,127,000
3,128,000
3,129,000
3,130,000
3,131,000
Ca21chr1_C_albicans_SC5314

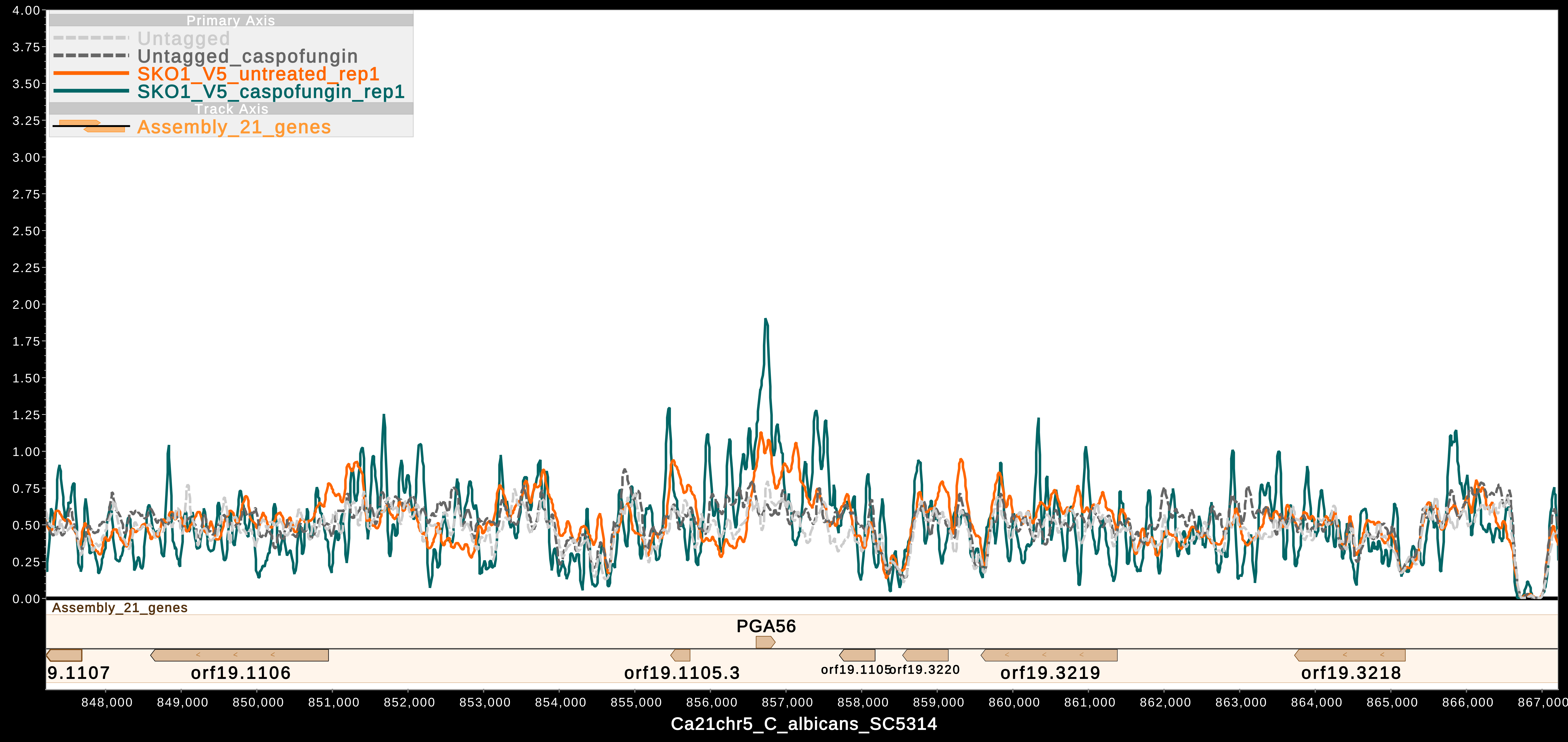

Primary Axis
Untagged
Untagged_caspofungin
SKO1_V5_untreated_rep1
SKO1_V5_caspofungin_rep1
Track Axis
Assembly_21_genes
4.00
3.75
3.50
3.25
3.00
2.75
2.50
2.25
2.00
1.75
1.50
1.25
1.00
0.75
0.50
0.25
0.00
Assembly_21_genes
PGA56
9.1107
orf19.1106
orf19.1105.3
orf19.1105
orf19.3220
orf19.3219
orf19.3218
848,000
849,000
850,000
851,000
852,000
853,000
854,000
855,000
856,000
857,000
858,000
859,000
860,000
861,000
862,000
863,000
864,000
865,000
866,000
867,000
Ca21chr5_C_albicans_SC5314

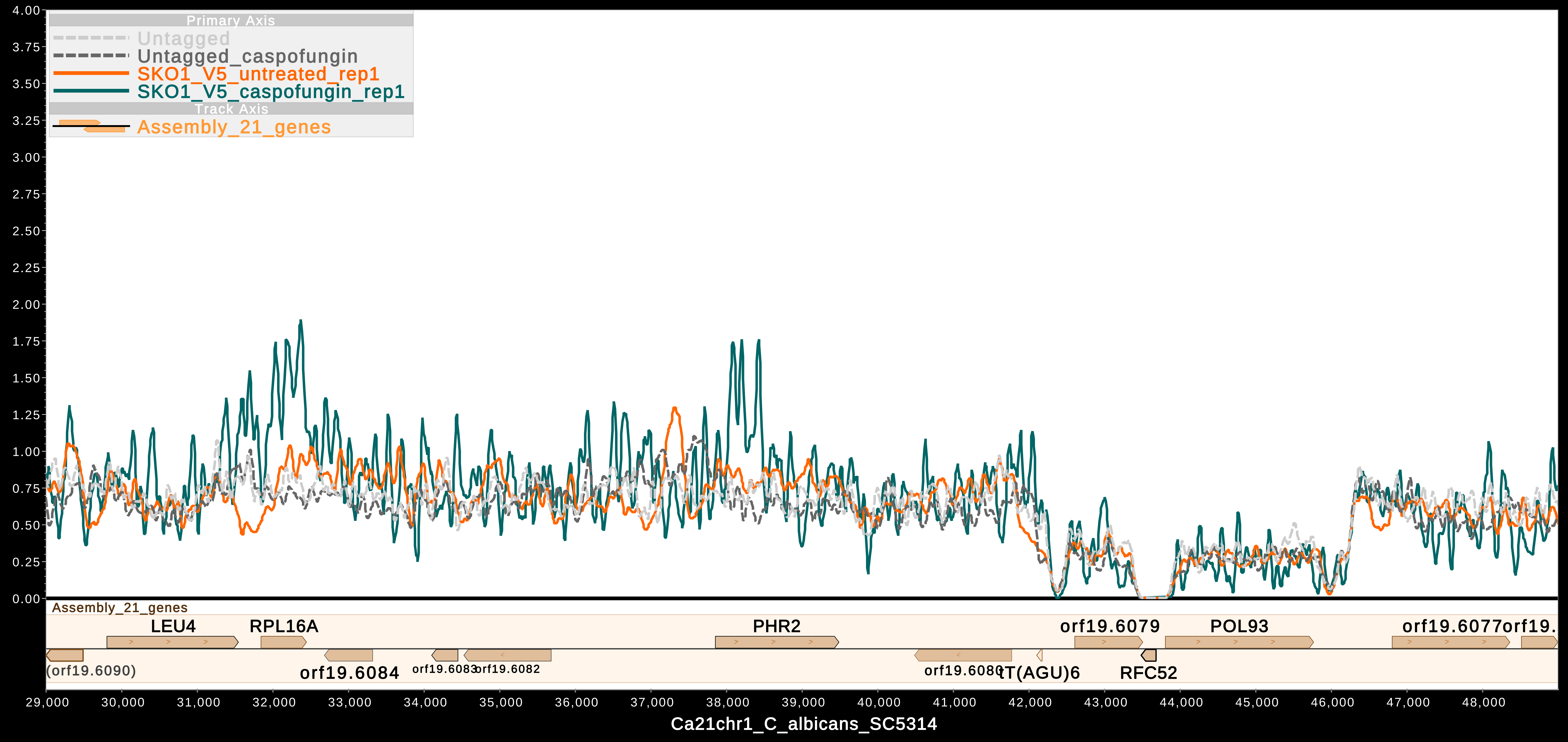

Primary Axis
Untagged
Untagged_caspofungin
SKO1_V5_untreated_rep1
SKO1_V5_caspofungin_rep1
Track Axis
Assembly_21_genes
4.00
3.75
3.50
3.25
3.00
2.75
2.50
2.25
2.00
1.75
1.50
1.25
1.00
0.75
0.50
0.25
0.00
Assembly_21_genes
LEU4
RPL16A
PHR2
orf19.6079
POL93
orf19.6077orf19.
(orf19.6090)
orf19.6084
orf19.6083
orf19.6082
orf19.6080
tT(AGU)6
RFC52
29,000
30,000
31,000
32,000
33,000
34,000
35,000
36,000
37,000
38,000
39,000
40,000
41,000
42,000
43,000
44,000
45,000
46,000
47,000
48,000
Ca21chr1_C_albicans_SC5314

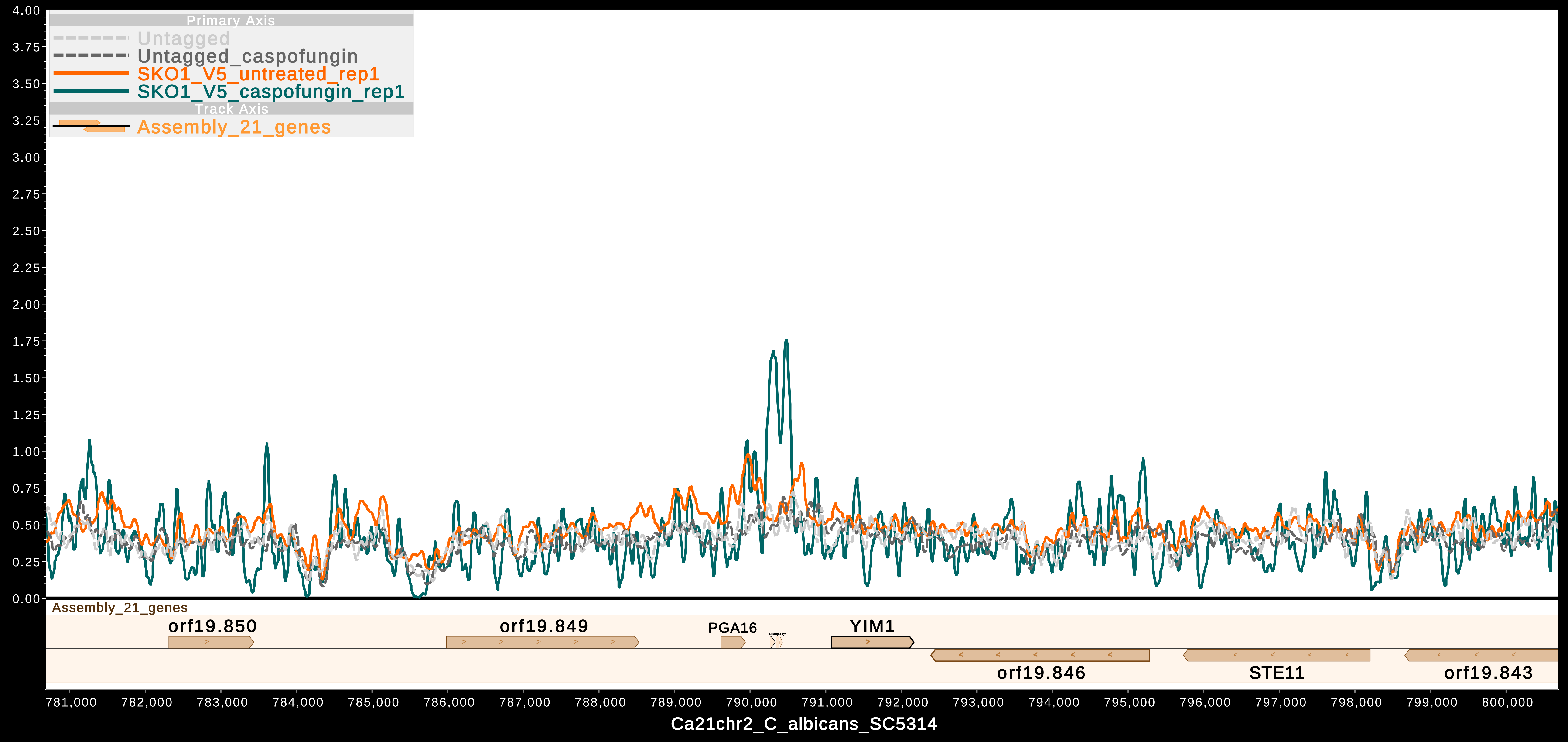

Primary Axis
Untagged
Untagged_caspofungin
SKO1_V5_untreated_rep1
SKO1_V5_caspofungin_rep1
Track Axis
Assembly_21_genes
4.00
3.75
3.50
3.25
3.00
2.75
2.50
2.25
2.00
1.75
1.50
1.25
1.00
0.75
0.50
0.25
0.00
Assembly_21_genes
orf19.850
orf19.849
PGA16
YIM1
orf19.846
STE11
orf19.843
781,000
782,000
783,000
784,000
785,000
786,000
787,000
788,000
789,000
790,000
791,000
792,000
793,000
794,000
795,000
796,000
797,000
798,000
799,000
800,000
Ca21chr2_C_albicans_SC5314

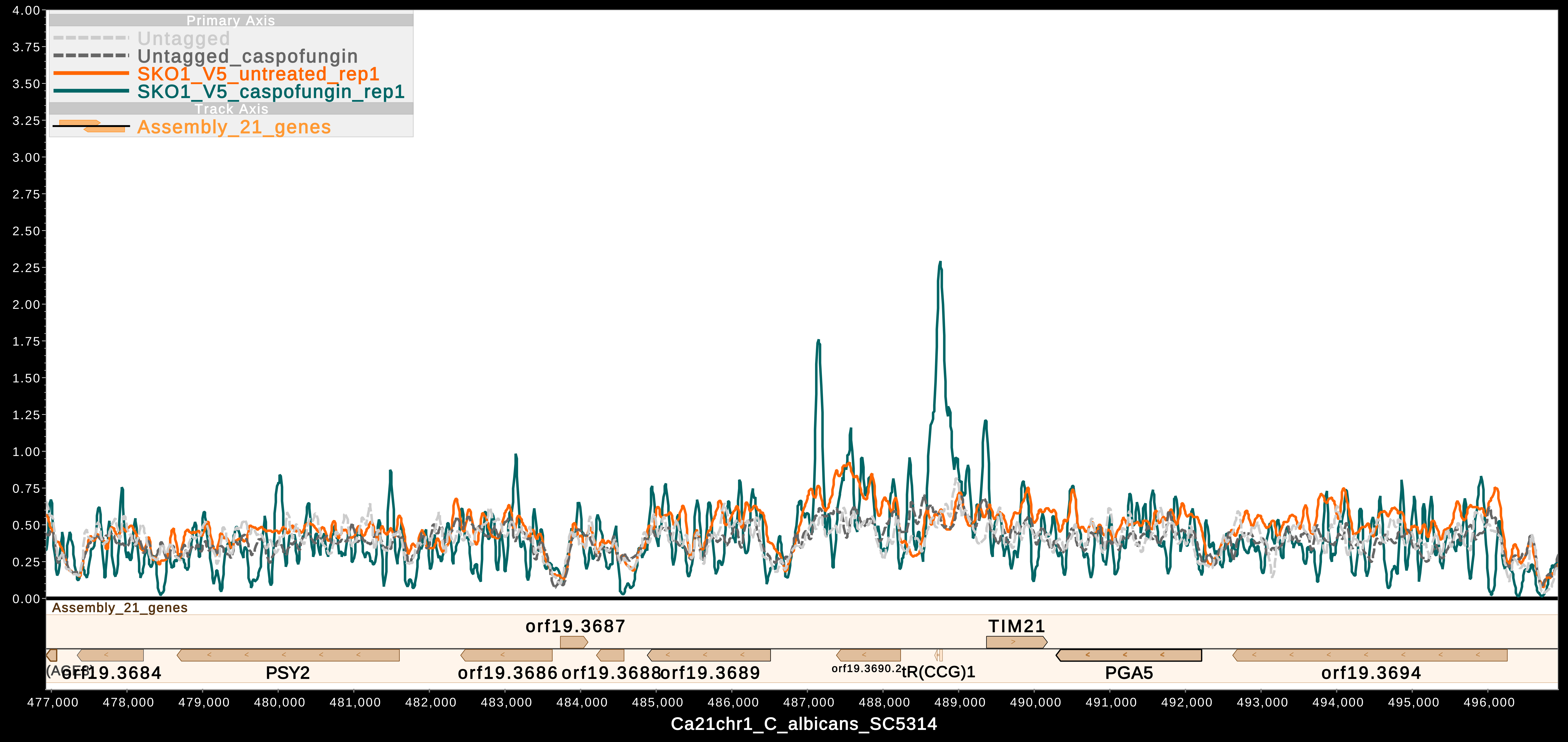

Primary Axis
Untagged
Untagged_caspofungin
SKO1_V5_untreated_rep1
SKO1_V5_caspofungin_rep1
Track Axis
Assembly_21_genes
4.00
3.75
3.50
3.25
3.00
2.75
2.50
2.25
2.00
1.75
1.50
1.25
1.00
0.75
0.50
0.25
0.00
Assembly_21_genes
orf19.3687
TIM21
orf19.3684
PSY2
orf19.3686
orf19.3688
orf19.3689
orf19.3690.2
tR(CCG)1
PGA5
orf19.3694
477,000
478,000
479,000
480,000
481,000
482,000
483,000
484,000
485,000
486,000
487,000
488,000
489,000
490,000
491,000
492,000
493,000
494,000
495,000
496,000
Ca21chr1_C_albicans_SC5314

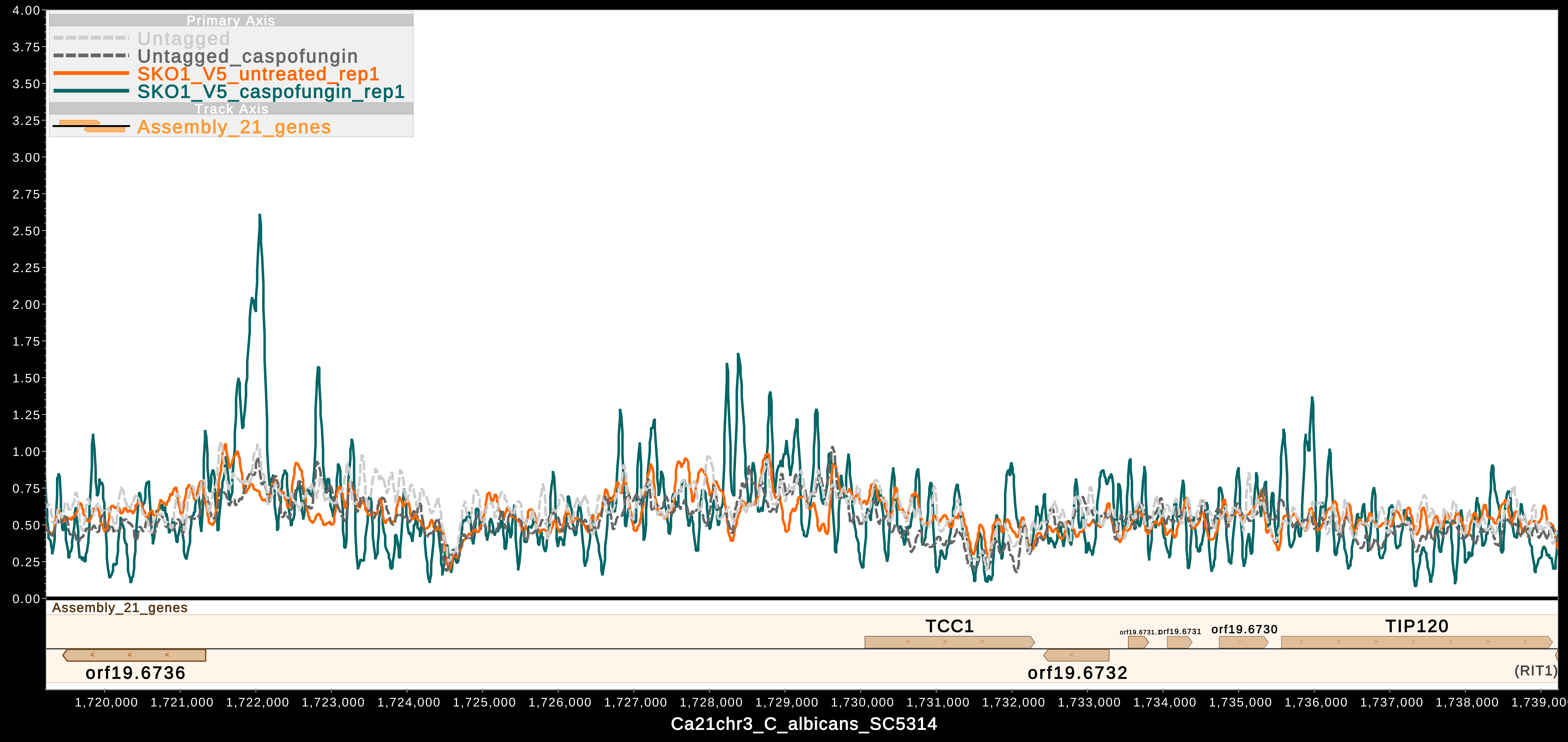

Primary Axis
Untagged
Untagged_caspofungin
SKO1_V5_untreated_rep1
SKO1_V5_caspofungin_rep1
Track Axis
Assembly_21_genes
4.00
3.75
3.50
3.25
3.00
2.75
2.50
2.25
2.00
1.75
1.50
1.25
1.00
0.75
0.50
0.25
0.00
Assembly_21_genes
TCC1
orf19.6731.1
orf19.6731
orf19.6730
TIP120
orf19.6736
orf19.6732
(RIT1)
1,720,000
1,721,000
1,722,000
1,723,000
1,724,000
1,725,000
1,726,000
1,727,000
1,728,000
1,729,000
1,730,000
1,731,000
1,732,000
1,733,000
1,734,000
1,735,000
1,736,000
1,737,000
1,738,000
1,739,000
Ca21chr3_C_albicans_SC5314

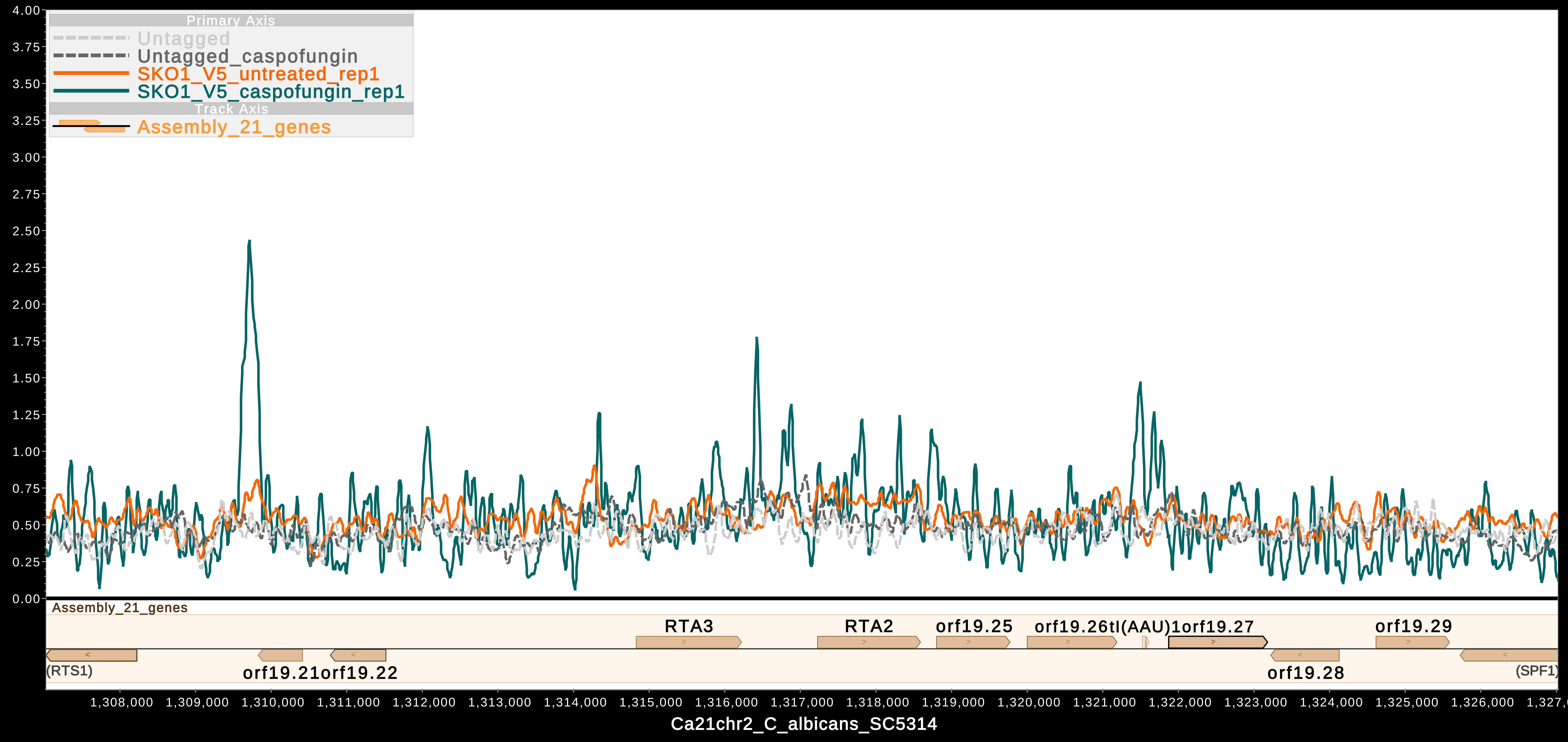

Primary Axis
Untagged
Untagged_caspofungin
SKO1_V5_untreated_rep1
SKO1_V5_caspofungin_rep1
Track Axis
Assembly_21_genes
4.00
3.75
3.50
3.25
3.00
2.75
2.50
2.25
2.00
1.75
1.50
1.25
1.00
0.75
0.50
0.25
0.00
Assembly_21_genes
RTA3
RTA2
orf19.25
orf19.26
tI(AAU)1
orf19.27
orf19.29
(RTS1)
orf19.21
orf19.22
orf19.28
(SPF1)
1,308,000
1,309,000
1,310,000
1,311,000
1,312,000
1,313,000
1,314,000
1,315,000
1,316,000
1,317,000
1,318,000
1,319,000
1,320,000
1,321,000
1,322,000
1,323,000
1,324,000
1,325,000
1,326,000
Ca21chr2_C_albicans_SC5314

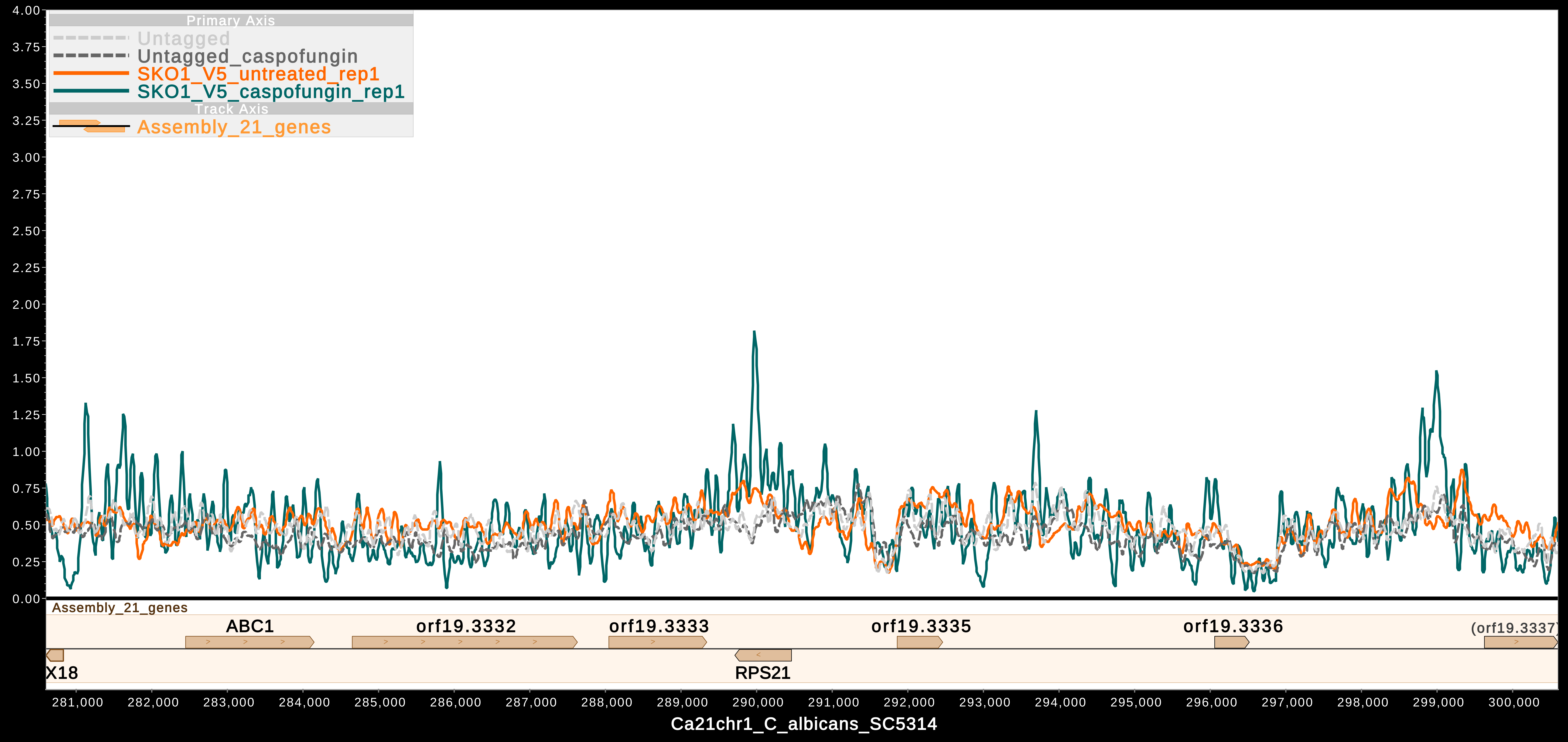

Primary Axis
Untagged
Untagged_caspofungin
SKO1_V5_untreated_rep1
SKO1_V5_caspofungin_rep1
Track Axis
Assembly_21_genes
4.00
3.75
3.50
3.25
3.00
2.75
2.50
2.25
2.00
1.75
1.50
1.25
1.00
0.75
0.50
0.25
0.00
Assembly_21_genes
ABC1
orf19.3332
orf19.3333
orf19.3335
orf19.3336
(orf19.3337)
X18
RPS21
281,000
282,000
283,000
284,000
285,000
286,000
287,000
288,000
289,000
290,000
291,000
292,000
293,000
294,000
295,000
296,000
297,000
298,000
299,000
300,000
Ca21chr1_C_albicans_SC5314

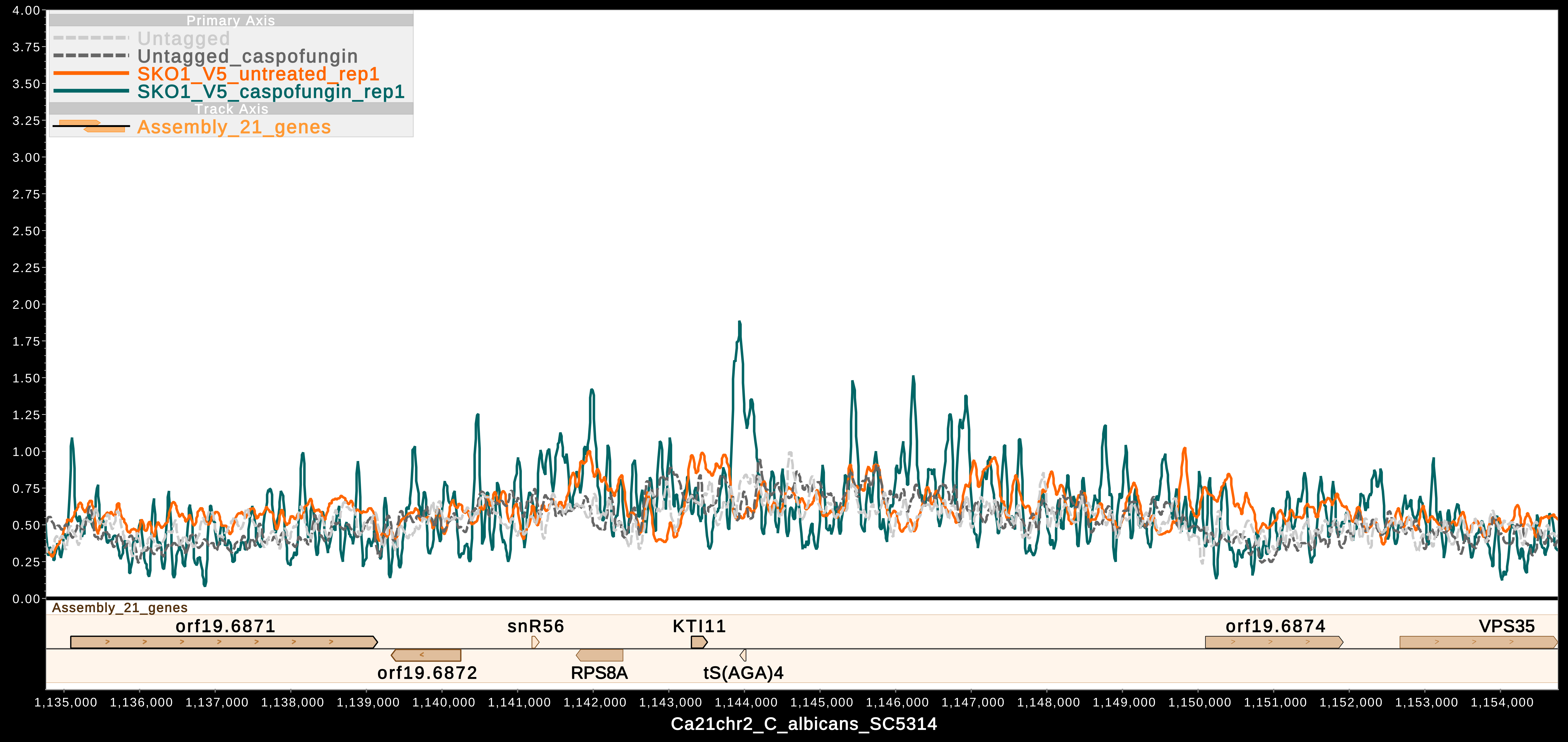

Primary Axis
Untagged
Untagged_caspofungin
SKO1_V5_untreated_rep1
SKO1_V5_caspofungin_rep1
Track Axis
Assembly_21_genes
4.00
3.75
3.50
3.25
3.00
2.75
2.50
2.25
2.00
1.75
1.50
1.25
1.00
0.75
0.50
0.25
0.00
Assembly_21_genes
orf19.6871
snR56
KTI11
orf19.6874
VPS35
orf19.6872
RPS8A
tS(AGA)4
1,135,000
1,136,000
1,137,000
1,138,000
1,139,000
1,140,000
1,141,000
1,142,000
1,143,000
1,144,000
1,145,000
1,146,000
1,147,000
1,148,000
1,149,000
1,150,000
1,151,000
1,152,000
1,153,000
1,154,000
Ca21chr2_C_albicans_SC5314

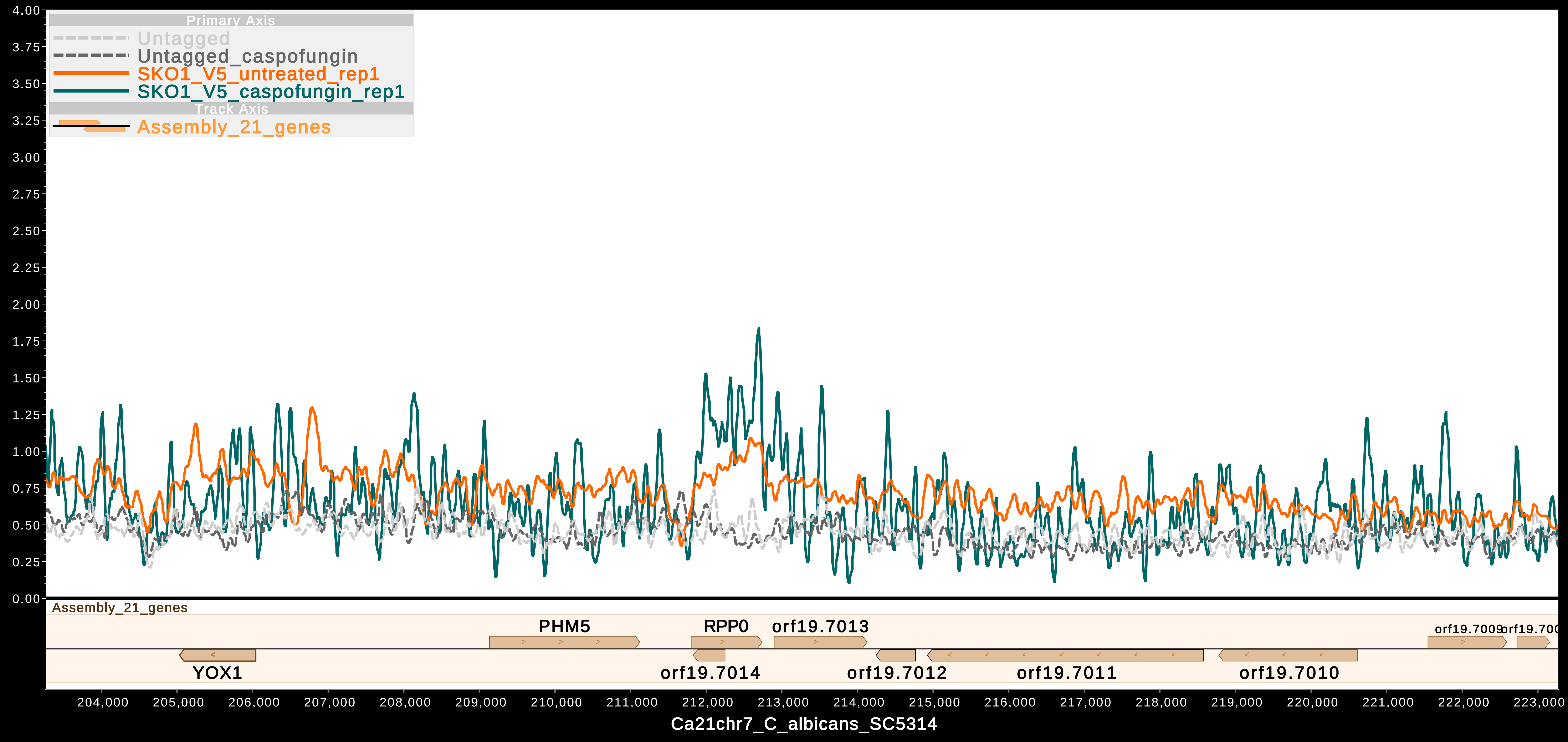

Primary Axis
Untagged
Untagged_caspofungin
SKO1_V5_untreated_rep1
SKO1_V5_caspofungin_rep1
Track Axis
Assembly_21_genes
4.00
3.75
3.50
3.25
3.00
2.75
2.50
2.25
2.00
1.75
1.50
1.25
1.00
0.75
0.50
0.25
0.00
Assembly_21_genes
PHM5
RPP0
orf19.7013
orf19.7009
YOX1
orf19.7014
orf19.7012
orf19.7011
orf19.7010
204,000
205,000
206,000
207,000
208,000
209,000
210,000
211,000
212,000
213,000
214,000
215,000
216,000
217,000
218,000
219,000
220,000
221,000
222,000
223,000
Ca21chr7_C_albicans_SC5314

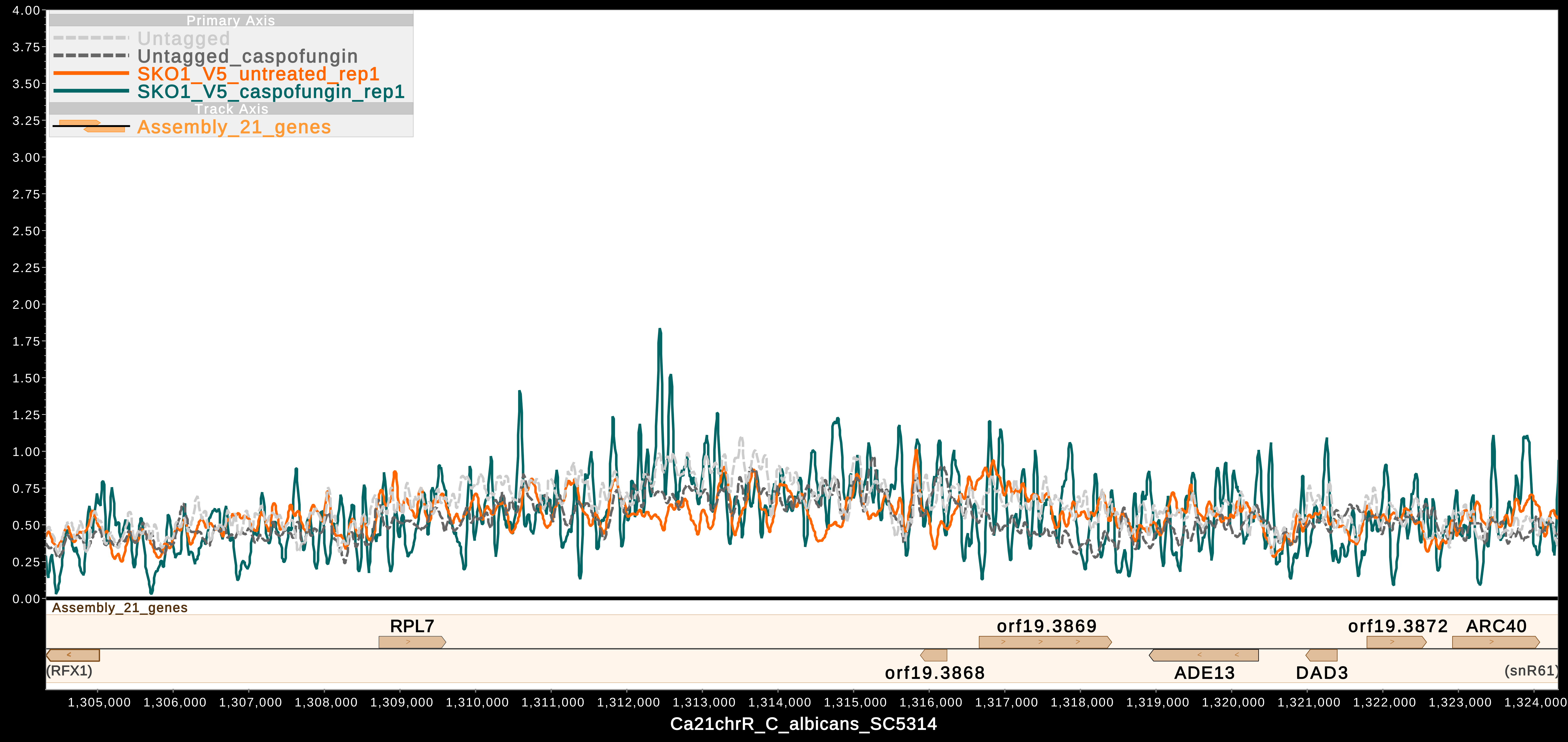

Primary Axis
Untagged
Untagged_caspofungin
SKO1_V5_untreated_rep1
SKO1_V5_caspofungin_rep1
Track Axis
Assembly_21_genes
4.00
3.75
3.50
3.25
3.00
2.75
2.50
2.25
2.00
1.75
1.50
1.25
1.00
0.75
0.50
0.25
0.00
Assembly_21_genes
RPL7
orf19.3869
orf19.3872
ARC40
(RFX1)
orf19.3868
ADE13
DAD3
(snR61)
1,305,000
1,306,000
1,307,000
1,308,000
1,309,000
1,310,000
1,311,000
1,312,000
1,313,000
1,314,000
1,315,000
1,316,000
1,317,000
1,318,000
1,319,000
1,320,000
1,321,000
1,322,000
1,323,000
1,324,000
Ca21chrR_C_albicans_SC5314

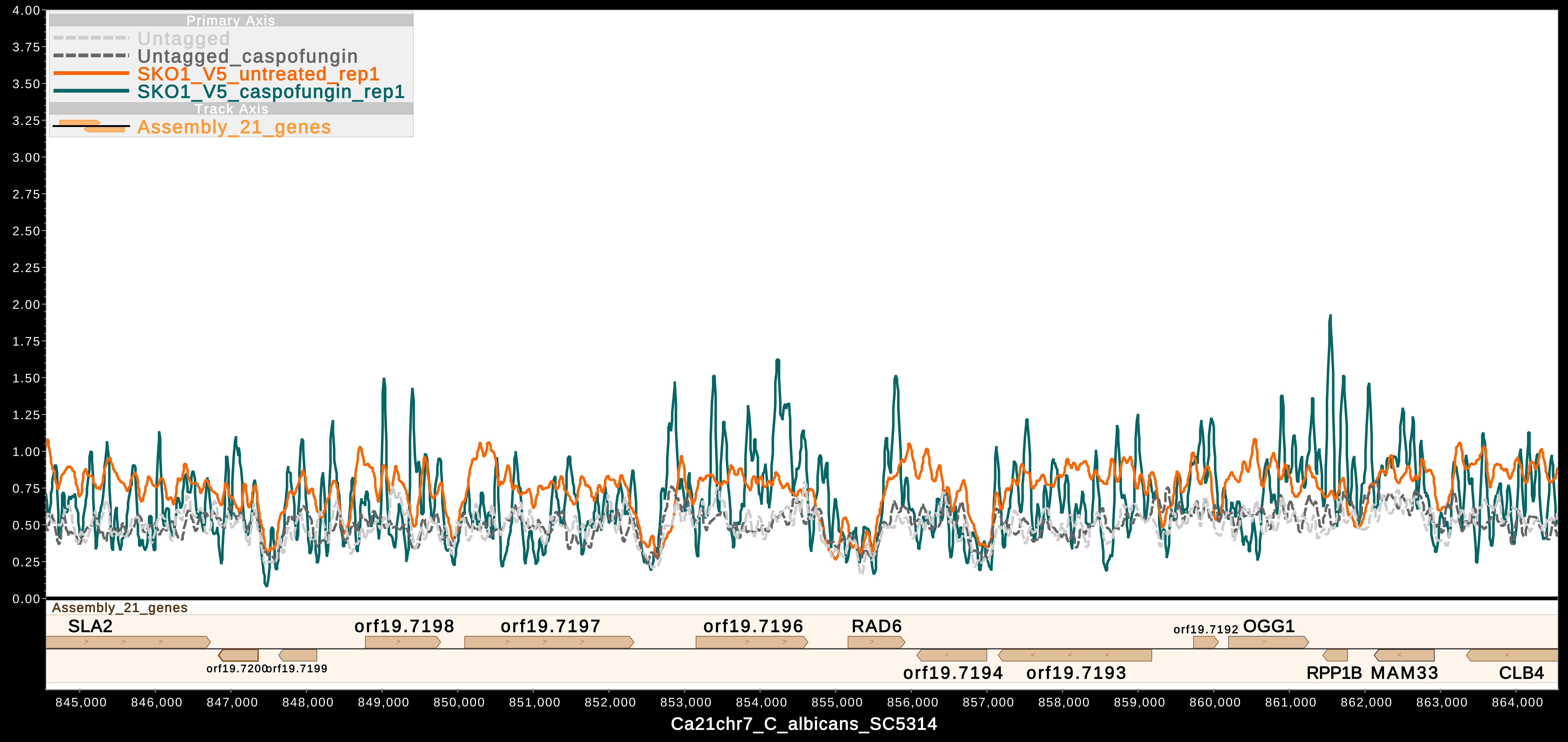

Primary Axis
Untagged
Untagged_caspofungin
SKO1_V5_untreated_rep1
SKO1_V5_caspofungin_rep1
Track Axis
Assembly_21_genes
4.00
3.75
3.50
3.25
3.00
2.75
2.50
2.25
2.00
1.75
1.50
1.25
1.00
0.75
0.50
0.25
0.00
Assembly_21_genes
SLA2
orf19.7198
orf19.7197
orf19.7196
RAD6
orf19.7192
OGG1
orf19.7200
orf19.7199
orf19.7194
orf19.7193
RPP1B
MAM33
CLB4
845,000
846,000
847,000
848,000
849,000
850,000
851,000
852,000
853,000
854,000
855,000
856,000
857,000
858,000
859,000
860,000
861,000
862,000
863,000
864,000
Ca21chr7_C_albicans_SC5314
