# Supplementary material for: An expanded cell wall damage signaling network is comprised of the transcription factors Rlm1 and Sko1 in Candida albicans
Source: PLoS Genet. 2020 Jul 8;16(7):e1008908. doi: 10.1371/journal.pgen.1008908 (PMC7371209; doi:10.1371/journal.pgen.1008908)
Supplement: Attachment — Submitted filename: Response_to_reviewers.pdf [file pgen.1008908.s008.pdf]

Jason Rauceo
Associate Professor of Biology
Department of Science
524 west 59th Street, Room 05.61.07NB
New York City, NY 10019
T. 646-557-4893
F. 212-621-3739
jrauceo@jjay.cuny.edu

Response to reviewers

Dear Dr. Butler and Dr. Copenhaver,

Thank you and the two reviewers for comments regarding our manuscript. We addressed all critiques and revised the manuscript accordingly. Our point-by-point response is presented below.

The reviewer's comments are shown in *italics*. The author's responses are in **bold**.

Reviewer #1

I am highly enthusiastic about the work presented and do not have significant comments.

Personally, I am interested in the cellular morphology and cell wall composition of the strains used in these studies and think some imaging would be beneficial to the reader and would aid in the understanding of the effect of these pathways on the overall cell structure and response. Additionally, it would be of interest to investigate the role of these pathways in the biofilm lifestyle of C. albicans and in non-albicans species. I also understand that due to the current situation, it is not plausible to acquire new data. This is just a suggestion and does not need to be incorporated unless the authors already have the data.

- **We have not investigated Sko1 or Rlm1 function in biofilm experiments for *C. albicans* or non-albicans species, although we can assure the reviewer that this is an area of interest for future studies.**

Reviewer #2

Key points

Fig 2, L247: The authors demonstrate a fairly constant (~40%) reduction in expression of Sko1 target genes in the rlm1 mutant. It would have been very useful in the figure to present the expression level of these genes in a sko1 mutant. This would have demonstrated the relative importance of Rlm1 and Sko1 in their regulation, and the interplay of these regulators in their control.

- **We previously published that the expression of these genes is significantly reduced in a *sko1Δ/Δ* mutant strain using DNA microarray and RT-qPCR analyses. We now address this in the body of the revised text (lines 137-139) and include the reference of those published results. The revised sentence is below:**

"Also, previous microarray and RT-qPCR results showed significantly reduced transcription of these genes in the *sko1Δ/Δ* mutant strain following caspofungin treatment

[6]".

The results of ChIP-seq for Rlm1 only identified a very small set of potential target genes, as such the use of GO enrichment approaches is somewhat questionable. However, this analysis did potentially link Rlm1 function with the spindle pole and trafficking. This result could be discussed in relation to the known role of Slt2 in regulating polarised growth in budding yeast.

- **Slt2 controls polarized growth in *S. cerevisiae*. However, it is unknown whether this function requires Rlm1, and we would rather not speculate on this unknown. The revised sentence discussing this point with accompanying references (lines 390-392) is below:**

"In *S. cerevisiae*, the Rlm1 regulator Slt2 is required for polarized cell growth [54, 55]; however, it is unknown whether Slt2 mediates cell growth via Rlm1. Our findings reveal a potential new role for Rlm1 function in *S. cerevisiae*".

In Fig 3 the results presented for Pga59 and Sec10 are unconvincing, with no clear peak.

- **While the enrichment peaks for *PGA59* and *SEC10* are not as striking relative to those of other Rlm1 targets, based on our statistical analyses of three independent ChIP samples for each experimental condition compared to three untagged control samples (see methods), Rlm1 does bind with significant enrichment to the upstream intergenic regions of *PGA59* and *SEC10.* Nevertheless, we agree with the reviewer that even though these peaks are significant, they are not striking, and therefore we have removed the MochiView plots for *PGA59* and *SEC10* in the revised Figure 3. In the revised narrative, we direct the reader to the S1 Appendix for the MochiView plots of *PGA59* (line 159) and *SEC10* (line 189).**

In the discussion of the results it is also important to differentiate between genes that are potentially regulated in response to caspofungin or those that are bound irrespective of its addition. For example, the authors discuss Pga56 alongside Pga59 & Pga62, however, in the case of Pga56 its promoter appears to be bound equally well in the presence or absence of the drug.

- **We have addressed this comment in the revised text (lines 482-484) as follows:**

  "Our ChIP-seq results identified *PGA59*, *PGA62*, and *PHR2* as both Rlm1 and Sko1 direct target genes, although we note that *PGA59* is bound by Rlm1 in both the presence and absence of caspofungin."

Finally, the authors identify Pga59 & Pga62 as potential targets, however, the binding site identified is identical in both cases plus appears to be more directly associated with orf19.2766.

- **orf19.2766 is annotated as a dubious ORF according to the Candida Genome Database (CGD). The reason for this designation is that it has not been found to be transcribed in any RNA-seq dataset to date. Give this information, we still list it as a potential binding target in case it is found to be transcribed in a condition that has not yet been explored by current RNA-seq datasets, however, it is much more likely that both of the *PGA* genes are being bound and possibly coregulated and that orf19.2766 is not transcribed. For this reason and to be inclusive of all potential binding events, we list both *PGA* genes as well as *orf19.2766* as potential targets.**

The authors state they screened for the presence of the baker's yeast Rlm1 binding motif in the promoter of C. albicans target genes (L257), however, no result of this is presented.

- **This was a typographical error. We intended to provide background information regarding Rlm1 target gene promoter analyses from a prior genomic study. We did not search our *C. albicans* Rlm1 ChIP-seq dataset for the presence of the *S. cerevisiae* Rlm1 DNA-binding consensus motif. The revised sentence with accompanying references (lines 250-253) is below:**

"For Rlm1, in silico promoter analysis has not been performed on microarray datasets, however Rlm1 target genes were previously screened for the presence of the *S. cerevisiae* Rlm1 binding motif CTAWWWWTAG [12, 42]".

Following on from this a highly diverged motif for Rlm1 binding in these two organisms is proposed, the authors could comment on how this fits with the conserved function of Rlm1 between these organisms and the likelihood of conserved targets.

- **We address this in the body of the revised text (lines 265-268). The revised sentence is below:**

"Although the function of Rlm1 in cell wall signaling is conserved in both species, this finding exemplifies the prevalence of transcriptional rewiring in *C. albicans*".

Minor points

Please provide a reference or further information on the pHAS01 vector.

- **We address this in the body of the revised text (lines 518-521). pHAS01 was incorrectly referenced, and the plasmid here should actually be pCJN613. The revised sentences are below:**

'We created plasmid pCJN613 as a template to produce an amplicon containing V5-specific sequences. This plasmid contains the V5 epitope tag sequence from the pYES2.1/V5-His-TOPO vector (Invitrogen) and the nourseothricin resistance gene (*NAT*) sequence from plasmid pCJN542 [22]".

L125: should be 'Sko1-specific sequences'

- **We address this in the body of the revised text (line 527).**

L:132 should be 'upstream of the RHR2 start codon'.

- **We address this in the body of the revised text (line 534).**

Fig 1A, L229: Is the increase of Sko1 expression in the cas5 mutant significant?

- **The increase of *SKO1* expression in the *cas5Δ/Δ* mutant strain is not significant. The revised sentences (lines 117-121) are below:**

"*SKO1* expression was relatively unchanged in the *cas5Δ/Δ* mutant strain under basal conditions and was slightly greater following caspofungin treatment compared to the wild-type strain; however, this increase was not significant."

Fig 1B, L238: Why was elevated expression of Sko1 seen in the complemented rlm1 mutant?

- ***SKO1* was integrated at the *HIS1* locus in the *rlm1Δ/Δ /RLM1* complemented strain, which is a standard approach for complementation in the field. Therefore, the expression of *RLM1* in the complemented strain that was integrated at the *HIS1* locus may not necessarily match the expression level at its native locus, and in this case appears to be slightly elevated. We note, however, that the *SKO1* expression levels in the complemented strain following caspofungin treatment was not significant (*P* > 0.05) when compared to the wild-type strain.**

L303: 'Highlighted a new biological role' is an overstatement, without further evidence please refer to as 'potential role'.

- **We address this in the body of the revised text (line 193-194).**

L307-325: What is the overlap between the 85 genes bound by Sko1 and the 26 originally described as caspofungin-inducible?

- **We address this in the body of the revised text. The revised sentences (lines 219-222) are below:**

"Specifically, in response to caspofungin, we identified 6 known Sko1 caspofungin-induced genes (out of the 26 caspofungin-induced gene targets identified in previous microarrays), including *PGA31* (Fig 4D), and 13 known Sko1 caspofungin-repressed genes (S2 Table)".

L318: Sko1 binds upstream of its own ORF in response to caspofungin. In addition, it also appears to bind across the ORF irrespective of the presence of caspofungin, the authors could comment on this.

- **We agree that it is interesting that Sko1 binds to its own promoter region and also appears to bind across its own ORF. It is difficult to explain binding over an ORF. This could be real and it could be a means of regulating gene expression. For example, Sko1 could be involved in activating transcription elongation. On the other hand, it could be an artifact of the ChIP-seq procedure, where it has been observed that transcription factors appear to show increased binding to the ORFs of genes that are more actively transcribed (see https://www.ncbi.nlm.nih.gov/pmc/articles/PMC3857294/).**

- **We now address this in the revised text (lines 207-212) as follows:**

  "In addition, Sko1 appears to bind across its own ORF irrespective of caspofungin treatment (Fig 4G and S2 Appendix). Although the basis of this binding is unclear, one possibility is that Sko1 is involved in activating transcription elongation. Alternatively, Sko1 ORF binding could be due to an artifact of the ChIP-seq procedure, which has been observed to occur for genes that are highly actively transcribed [41]."

L377 Does the motif for Sko1 identified relate to that previously reported for genes activated or repressed by Sko1?

- **The identified motif is similar to a previously identified motif for Sko1-activated genes. We address this in the body of the revised text (line 274-276).**

Fis S2: Please provide stats analysis for Pga13 & Pga31, the same as that presented for Crh11.

- **S2 figure was revised accordingly.**

Suppl. Table 2, Wksheet 2: For Rlm1 the top 50%, not 25%, enriched proteins were analysed according to the methods section.

- **S2 Table was revised accordingly.**

Fig S4 L357: PGA13 is only one of multiple genes that could play a role, as such looking at only one potential gene did not really add to the manuscript.

- **S4 figure and corresponding discussion points were deleted from the manuscript.**
